# Developmental Neurotoxicity and Behavioral Screening in Larval Zebrafish with a Comparison to Other Published Results

**DOI:** 10.3390/toxics10050256

**Published:** 2022-05-17

**Authors:** Kimberly A. Jarema, Deborah L. Hunter, Bridgett N. Hill, Jeanene K. Olin, Katy N. Britton, Matthew R. Waalkes, Stephanie Padilla

**Affiliations:** 1 Center for Public Health and Environmental Assessment, Immediate Office, Program Operations Staff, U.S. Environmental Protection Agency, Research Triangle Park, NC 27711, USA; 2 Center for Computational Toxicology and Exposure, Biomolecular and Computational Toxicology Division, Rapid Assay Development Branch, U.S. Environmental Protection Agency, Research Triangle Park, NC 27711, USA; hunter.deborah@epa.gov (D.L.H.); olin.jeanene@epa.gov (J.K.O.); 3 ORISE Research Participation Program Hosted by EPA, Center for Computational Toxicology and Exposure, Biomolecular and Computational Toxicology Division, Rapid Assay Development Branch, U.S. Environmental Protection Agency, Research Triangle Park, NC 27711, USA; hill.bridgett@epa.gov; 4 ORAU Research Participation Program Hosted by EPA, Center for Computational Toxicology and Exposure, Biomolecular and Computational Toxicology Division, Rapid Assay Development Branch, U.S. Environmental Protection Agency, Research Triangle Park, NC 27711, USA; britton.katy@epa.gov; 5 ORISE Research Participation Program Hosted by EPA, National Health and Environmental Effects Research Laboratory, Integrated Systems Toxicology Division, Genetic and Cellular Toxicology Branch, U.S. Environmental Protection Agency, Research Triangle Park, NC 27711, USA; mrw0051@mix.wvu.edu

**Keywords:** behavior, chemical screening, literature comparison, developmental toxicity, developmental neurotoxicity, negative control, positive control, rapid testing, zebrafish

## Abstract

With the abundance of chemicals in the environment that could potentially cause neurodevelopmental deficits, there is a need for rapid testing and chemical screening assays. This study evaluated the developmental toxicity and behavioral effects of 61 chemicals in zebrafish (*Danio rerio*) larvae using a behavioral Light/Dark assay. Larvae (n = 16–24 per concentration) were exposed to each chemical (0.0001–120 μM) during development and locomotor activity was assessed. Approximately half of the chemicals (n = 30) did not show any gross developmental toxicity (i.e., mortality, dysmorphology or non-hatching) at the highest concentration tested. Twelve of the 31 chemicals that did elicit developmental toxicity were toxic at the highest concentration only, and thirteen chemicals were developmentally toxic at concentrations of 10 µM or lower. Eleven chemicals caused behavioral effects; four chemicals (6-aminonicotinamide, cyclophosphamide, paraquat, phenobarbital) altered behavior in the absence of developmental toxicity. In addition to screening a library of chemicals for developmental neurotoxicity, we also compared our findings with previously published results for those chemicals. Our comparison revealed a general lack of standardized reporting of experimental details, and it also helped identify some chemicals that appear to be consistent positives and negatives across multiple laboratories.

## 1. Introduction

The incidence of neurodevelopmental deficits in children is steadily increasing (reviewed in [1,2]), accompanied by warnings from many scientific fronts regarding the possible adverse effects of environmental chemicals on nervous system development [3,4,5]. The evidence that chemicals may alter the trajectory of brain development has led to heightened awareness of the need for rapid testing of environmental chemicals for developmental neurotoxicity potential. An experimental model that appears to hold promise is a small, hardy, aquarium fish: zebrafish (*Danio rerio*). Elegant work in zebrafish has been published on the development of the nervous system, neuronal pathfinding, myelination, and the genetic or structural basis of nervous system function (e.g., [6,7]). Because of the concordance between zebrafish and human developmental and neurodevelopmental pathways, zebrafish are now used to discover mechanisms, and possibly treatments, of neurological diseases [8,9,10,11]. Many in vitro tests have been developed to assess specific aspects of brain development, but because brain development is complicated, with pre-described windows of migration and connectivity orchestrated by endocrine crosstalk and feedback, a whole animal model is often part of many developmental neurotoxicity screening batteries. 

Behavioral assessment, regarded as a functional endpoint, is an integrative signal representing nervous system status or fitness [12,13,14]. Not only are larval zebrafish able to exhibit many different behaviors [15,16] but, analogous to mammalian neurodevelopment, the development of the zebrafish nervous system is guided and influenced by the interplay among brain development and endocrine systems such as the hypothalamus-pituitary-thyroid (HPT) axis [17,18,19] and the hypothalamo-pituitary-adrenal (inter-renal in zebrafish) (HPA/HPI) axis (reviewed in [20]). Moreover, zebrafish at all developmental stages metabolize toxic chemicals using pathways similar to mammals [21,22]. Because of these attributes, zebrafish are often proffered as a model for developmental neurotoxicity screening (reviewed in [23,24,25]). 

Using the zebrafish model, we had two main goals for this study: to screen a library of chemicals for developmental neurotoxicity, and to compare our findings with previously published results for those chemicals. The specific chemical library was chosen because (1) some of the chemicals have been associated with developmental neurotoxicity in mammals [26]; (2) many of the chemicals were tested by other investigators within the U.S. Environmental Protection Agency (EPA) using in vitro assays for developmental neurotoxicity potential [27,28,29]; and/or (3) some of the chemicals have been tested by investigators external to EPA using zebrafish assays [30,31,32,33,34,35,36,37,38,39,40,41,42,43,44,45,46,47,48,49,50,51,52,53,54,55,56,57,58,59,60,61,62,63]. The first aspect of this study was to screen the library of 61 chemicals in zebrafish embryos/larvae to determine if the chemical (maximum nominal concentration = 120 µM) produced developmental toxicity (lethality, non-hatching or malformations) and/or neurotoxicity (changes in larval locomotor activity). The second aspect of this study was to compare our results with the results from other laboratories performing similar behavioral assays with larval zebrafish treated with the same chemicals during development. 

## 2. Materials and Methods

### 2.1. Chemicals

Table 1 lists information about the chemicals used in this study. The chemical name, CAS number, DTXSID, molecular weight, and solvent (vehicles) are included. Also included are the predicted median and range of water solubility, as well as the predicted median and range of the octanol/water partition coefficient, all of which were obtained from the EPA’s Chemicals Dashboard (https://comptox.epa.gov/dashboard/; last accessed on 31 January 2022). For the creation of stock plates, stock solutions of each chemical were prepared in their respective vehicles, either dimethyl sulfoxide (DMSO; Anhydrous (>99.9% pure) from Sigma-Aldrich] or deionized water, which were then used for subsequent serial dilutions for dosing of the experimental plates. Chlorpyrifos [ethyl; CAS# 39475-55-3) served as the positive control for behavioral alterations [64]. The highest nominal concentration tested of any chemical was 120 µM because human plasma rarely exceeds micromolar levels of most environmental chemicals. 

The primary medium for rearing the embryos was 10% Hanks’ Balanced Salt Solution (13.7 mM NaCl, 0.54 mM KCl, 25 μM Na2HPO4, 44 μM KH2PO4, 130 μM CaCl2, 100 μM MgSO4, and 420 μM NaHCO3; pH = 7.6 ± 0.2; all salts obtained from Sigma-Aldrich, St. Louis, MO; hereafter referred to as 10% Hanks’). The lead (Pb) exposed larvae were not exposed in 10% Hanks’ solution because of concerns about possible precipitation of the lead in that solution. Rather, larvae exposed to lead were reared in 1X EPA Moderately Hard Reconstituted Water (MHW: 54 µM KCl, 0.5 mM MgSO4·7H2O, 1.1 mM NaHCO3, 350 µM CaSO4; hereafter referred to as MHW). We have previously shown that control animals reared in either Hanks’ solution or MHW do not differ in their locomotor activity [65].

### 2.2. DMSO Evaluation

Some publications [66,67,68] have noted that exposure to DMSO at very low concentrations can affect larval zebrafish behavior. Therefore, we determined if the vehicle concentration (0.4% DMSO) in our developmental exposure regimen caused any behavioral changes in 6 days post fertilization (dpf) larvae tested using our behavioral protocol. The experiment was conducted under the same experimental conditions described below with both DMSO exposed and non-DMSO exposed animals on the same microtiter plate. For non-DMSO exposed animals, water was added in place of the DMSO. The results presented in Supplemental Figure S1 show no effect of DMSO exposure during development on the behavior of the zebrafish larvae. 

### 2.3. Experimental Animals

All studies were carried out in accordance with the guidelines of, and approved by, the Office of Research and Development’s Institutional Animal Care and Use Committee (IACUC) at the U.S. Environmental Protection Agency (EPA) in Research Triangle Park, NC, USA.

In-house, wild type adult zebrafish (*Danio rerio*) descended from undefined, outbred stock originally obtained from Aquatic Research Organisms (Hampton, NH, USA) and EkkWill Waterlife Resources (Ruskin, FL, USA) were used. Each year, as replacement breeders are reared, embryos of a new strain are mixed with the in-house strain to maintain the outbred status of the colony. Animals were housed in an American Association for Accreditation of Laboratory Animal Care (AAALAC) approved animal facility with a 14:10 h light/dark cycle (lights on at 0700 h). Adult fish were kept in flow-through colony tanks (Tecniplast USA, West Chester, PA or Aquaneering Inc., San Diego, CA, USA) with a water temperature of 28 °C. The system water is composed of Durham, NC city tap water that is purified via reverse osmosis and buffered with sea salt (Instant Ocean, Spectrum Brands, Blacksburg, VA, USA) and sodium bicarbonate (Church & Dwight Co., Ewing, NJ, USA). This water is maintained at pH 7.4, conductivity of 1000 µS/cm, with negligible ammonia and nitrate/nitrite present. For egg collection, adults from colony tanks were placed in a 2-L (static) breeding tank (Aquatic Habitats, Apopka, FL, USA) the night prior to embryo collection. At 0730 h the following morning, approximately 30 min after the light illumination, eggs were collected.

### 2.4. Experimental Procedure

**Figure 1:**
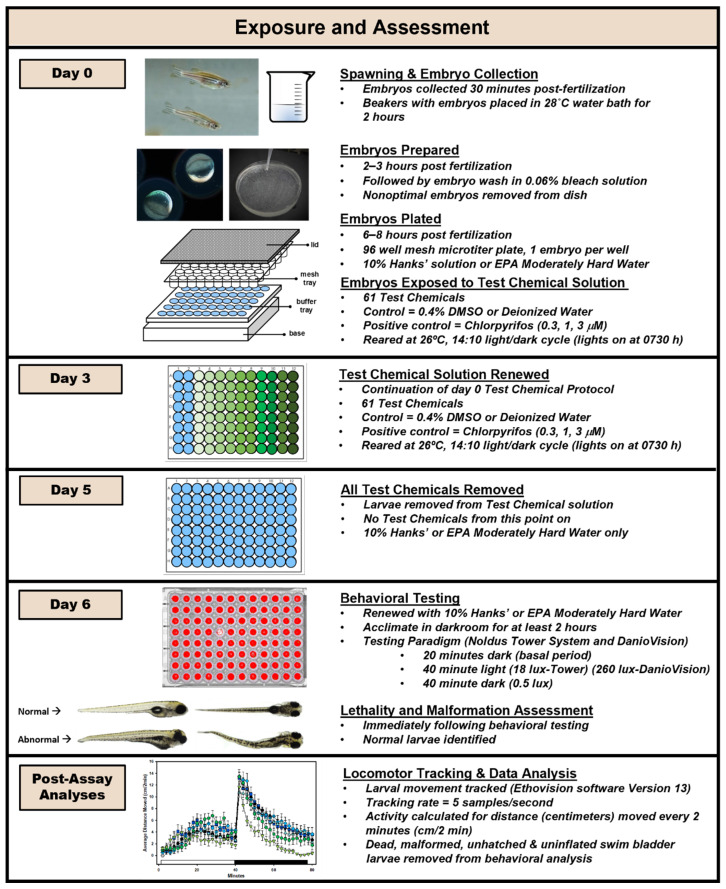
**Experimental Design**. Detailed timeline of the experimental process from spawning to analyses. The diagram is divided into sections for each critical time period, then further subdivided for event.

The Experimental Procedure is outlined in Figure 1 and explained in detail below. In the conduct and analysis of our behavioral assay, it was important that developmental neurotoxicity rather than the pharmacological effects of each chemical was assessed. To accomplish this, our experimental procedure included removal of the chemical from the dosing solution 24 h before testing and replacing the test chemical with a vehicle solution. We have previously shown that this removal of the test chemical markedly alters the behavioral profile, separating neuroactive from neurodevelopmental effects [64], although it is possible that this depuration time interval may not be long enough for all chemicals. We also wanted to limit the possibility that morphological changes alter the swimming behavior of the larvae, as this would seriously confound the interpretation of behavioral changes. We are assuming that any changes in swimming activity during the behavioral assessment is due to nervous system function and not changes in physical locomotor ability precipitated by teratological changes. To accomplish this, each animal was carefully assessed for any morphological changes, including swim bladder inflation as swim bladder inflation status has been shown to affect behavioral endpoints [69,70]. 

### 2.5. General Embryo Rearing

Newly collected embryos were washed with a dilute bleach solution shortly after collection. This process consisted of submerging the embryos in 0.06% bleach (*v*/*v*) in 10% Hanks’ two times, for five minutes each, and then briefly rinsing in 10% Hanks’ 3 times after each bleach wash [71]. Healthy, normal appearing embryos were individually placed, with their intact chorion (i.e., embryos were not dechorionated), into the upper mesh insert of a 96-well microtiter plate (Multiscreen™, Millipore Sigma, Burlington, MA, USA), which was submerged in a receiver plate containing 10% Hanks’ solution.

### 2.6. Chemical Exposure

After plating (6–8 h post fertilization (hpf)), embryos were immersed in the appropriate chemical solution. To accomplish this, the upper mesh insert containing the embryos was blotted on glass fiber filter paper (Whatman GF/B paper (fired) Brandel, Gaithersburg, MD) and placed in the new 96-well receiving plate, which contained the appropriate chemical concentration. To dilute the chemicals, 1 µL from the stock plate was added per well to the receiving plate containing 150 µL of 10% Hanks’, followed by an additional 100 µL of 10% Hanks’ solution after the transfer of mesh insert. All concentrations of each chemical, along with vehicle controls, were included on every plate. Each plate was sealed with a non-adhesive material (Microseal^®^ A, BioRad, Hercules, CA, USA), covered with a lid, and wrapped in Parafilm™ to secure the lid to the plate. The treated embryo plates were placed in a secondary container in the incubator (Lab-Line Imperial III, Barnstead International, Dubuque, IA, USA) and reared for 6 days at 26 °C under a 14:10 light:dark cycle (lights on at 0730 h). In addition to day 0, the 250 µL of 10% Hanks’ solution, along with the appropriate chemical and concentration, in each well was completely renewed on 3 dpf (as described above). On 5 dpf, larvae were transferred to 10% Hanks’ solution only (i.e., did not contain experimental chemical). On the morning of 6 dpf, the larvae were transferred again to 10% Hanks’ without chemical and placed in the pre-warmed behavioral testing darkroom. Zebrafish larvae at 6 dpf, reared at 26 °C (5 dpf if reared at 28.5 °C), are at an optimal age for behavioral testing since their locomotor activity and response to visual stimuli are well developed in preparation for independent feeding behaviors that begin at 7 dpf. 

The chlorpyrifos (0.3, 1.0 or 3 µM) positive control plates followed the same chemical exposure procedure described above. These positive control plates were tested throughout the study at intervals of about 60 days to ensure that the system was working properly.

### 2.7. Behavioral Testing Systems

These experiments utilized two larval zebrafish behavior systems for recording fish locomotion: a Noldus Tower System and a Noldus DanioVision System (model DVOC-0030), both manufactured by Noldus Information Technology, Leesburg, VA, USA. These systems are hereafter referred to as “Tower” or “DanioVision”. Each system was equipped with a light box that provided both infrared and visible light. The luminance of the Light portion of the testing paradigm was 260 lux (DanioVision) or 18 lux (Tower), and that of the Dark portion was 0.5 lux on both systems. Luminance measures were taken at the level of the recording platform using a photometer (Sper Scientific, model # 840022, Scottsdale, AZ, USA). 

**Figure 2:**
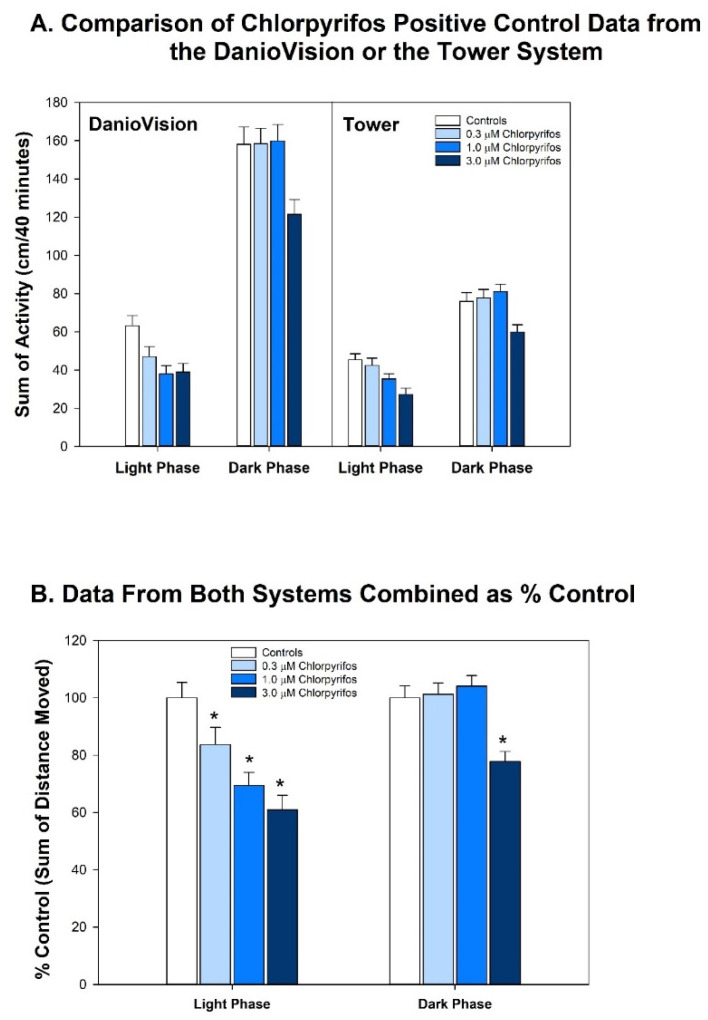
**Comparison of the Developmental Chlorpyrifos Effect When Tested on Either the DanioVision or Tower System.** Upper (**A**) shows the results when larvae treated with chlorpyrifos during development were tested on either the DanioVision system (**left** panel) or Tower system (**right** panel). Using an ANOVA with chlorpyrifos treatment and the system tested as independent variables, and locomotor activity as the dependent variable, it was found that there was an overall effect of chlorpyrifos (*p* < 0.0001) and of the system used (*p* < 0.0001), but that there was no interaction between those two variables (*p* = 0.22). Because the effect of the chlorpyrifos did not depend on the system that was used for testing, the data from both systems were combined, expressed as a percent of control and analyzed to delineate the effect of chlorpyrifos (**B**). In this case the data were analyzed using an ANOVA (chlorpyrifos or Light/Dark period were independent variables and locomotor activity was the dependent variable). This analysis showed that there was an overall effect of chlorpyrifos (*p* < 0.0001), Light/Dark period (*p* < 0.0001), and that there was an interaction between the two (*p* = 0.0003), meaning that the effect of chlorpyrifos was different depending on whether the animals were tested in the Light or Dark period. Using an ANOVA and testing each period separately, it was first determined whether there was an overall effect of chlorpyrifos concentration (*p* < 0.0001 in either the Light or Dark) and then a Fisher’s PLSD *post hoc* test was conducted to determine which chlorpyrifos concentration was different from control in either the Light or Dark period. Those concentrations that were different from control are indicated by an asterisk. In the Light period the 0.3 µM (*p* = 0.03), 1.0 µM (*p* < 0.0001) and the 3.0 µM (*p* < 0.0001) chlorpyrifos were all different from control, while in the Dark period, only the highest concentration 3.0 µM (*p* < 0.0001) was different from control. For the DanioVision system testing, the sample sizes were 63 controls, 65 at 0.3 µM, 63 at 1.0 µM, and 61 at 3.0 µM, and for the Tower system, the sample sizes were 69 controls, 62 at 0.3 µM, 69 at 1.0 µM and 54 at 3.0 µM. The sample sizes for (**B**) were a combination of each of those sample sizes for each system at each concentration.

Due to unavoidable circumstances, it was necessary to switch recording systems while the experiments were underway. Using two different systems for behavioral assessment is not ideal; however, data from each system indicated they were comparable. For this comparison, larval zebrafish (6 dpf) treated with the positive control, chlorpyrifos (0.3, 1.0 or 3 µM), following identical exposure configuration and behavior testing protocols, were tested on each system. This comparison (Figure 2) shows that the animals tested on the two systems exhibited different levels of baseline activity, but when animals exposed to chlorpyrifos during development (our positive control) were tested on both systems, the results did not differ. Panel A in Figure 2 shows the activity of the larvae in the Light or Dark period in either the DanioVision (left panel) system or the Tower (right panel) system. Note that the animals appear to be more active in the DanioVision system (overall effect of system: *p* < 0.0001): about 40% more activity in the Light and about twice as much activity in the Dark period. There is also an overall effect of chlorpyrifos (*p* < 0.0001), but there is no interaction of the chlorpyrifos effect with system used, meaning that the pattern of the chlorpyrifos effect is not dependent on whether the DanioVision or Tower system was used. Figure 2 (Panel B) shows the effect of chlorpyrifos when the data from both systems are combined as percent of control to correct for the differences in baseline activity. In the Light period chlorpyrifos exposure during development depressed locomotor activity at all three concentrations, while in the Dark only the animals exposed to the highest concentration (3 µM) of chlorpyrifos during development showed hypoactivity.

### 2.8. Behavioral Testing

All testing was performed on 6 dpf larvae in the same 96-well mesh plate in which they had been exposed and reared. On the morning of testing (6 dpf) the rearing solution was totally renewed, and the plates were moved to a light-tight drawer in the behavioral testing darkroom where the ambient temperature was the same as the rearing incubator (26 °C). For all experiments, testing occurred between 1200 and 1630 h. After acclimating in the behavioral testing room for at least 2 h, the plates were transferred to either the Tower or DanioVision recording platform light box to begin behavioral testing. The testing paradigm consisted of a 20-min acclimation period in the dark (Basal period), followed by 40 min of light (Light) followed by 40 min of dark (Dark). Prior research in this laboratory, and several others [72,73,74,75,76], have demonstrated that zebrafish larvae exposed to light drastically increase locomotor activity when transitioned to darkness. The Basal period serves to minimize any behavioral disruption due to transfer of the plate and larvae to the recording platform. Data were collected during this acclimation period but were not analyzed further because of a lack of specification and stimulus control. 

For both the Tower and DanioVision systems, fish movement (locomotion) was recorded using Media Recorder software (Noldus Information Technology, Leesburg, VA, USA) and saved as MPEG2 files, a process initially described by MacPhail [77]. 

### 2.9. Lethality and Malformation Assessment and Inclusion Criteria

Immediately following behavioral testing, larvae were assessed by observers (blinded to treatment conditions) for death and malformations using an Olympus SZH10 stereo microscope. Morphological assessments focused on the following: craniofacial (abnormal eyes or head), spinal (stunted, curved, or kinked tail), abdominal region (edema or emaciation), thoracic region (distention or heart malformations), swim bladder inflation, and position in the water column (floating or lying on side). All dead, unhatched, malformed larvae, and those with uninflated swim bladders, were eliminated from any behavioral analysis; malformed 6 dpf zebrafish larvae, as well as normal appearing larvae with uninflated swim bladders, do not behave normally in our behavioral paradigm [65,69]. Following the assessments, larvae were anaesthetized using cold shock and then euthanized with 20% (*v*/*v*) bleach solution. 

There were multiple levels of embryo quality acceptance for inclusion in the behavioral data. First, at the plate level, if more than 15% of the control larvae were abnormal, then no data from that plate were used; the plate was discarded and repeated. Next, at the concentration level, if more than 25% of the larvae from any concentration group were abnormal, then that entire concentration was removed from further behavior analyses, though the data were still used for developmental toxicity evaluation. The 75% concentration group threshold was established because it was thought that if any more than 25% of the animals were abnormal at a given concentration, then the developmental toxicity of that chemical concentration outweighed the neurodevelopmental toxicity. Lastly, each individual embryo included in the behavioral analyses must have appeared normal (i.e., no obvious malformations). 

**Figure 3:**
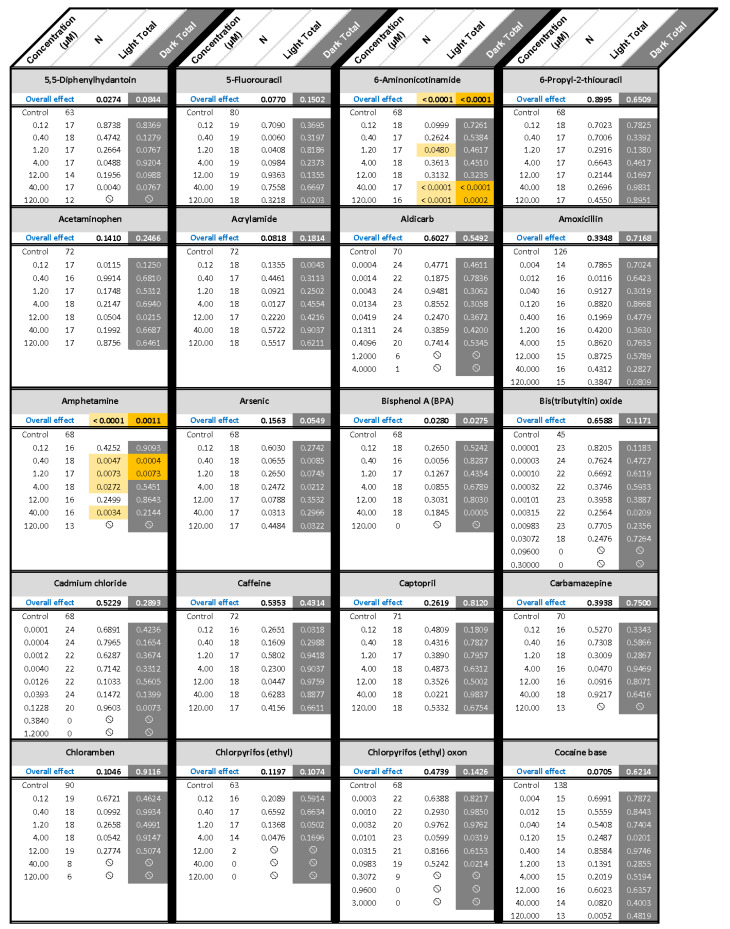

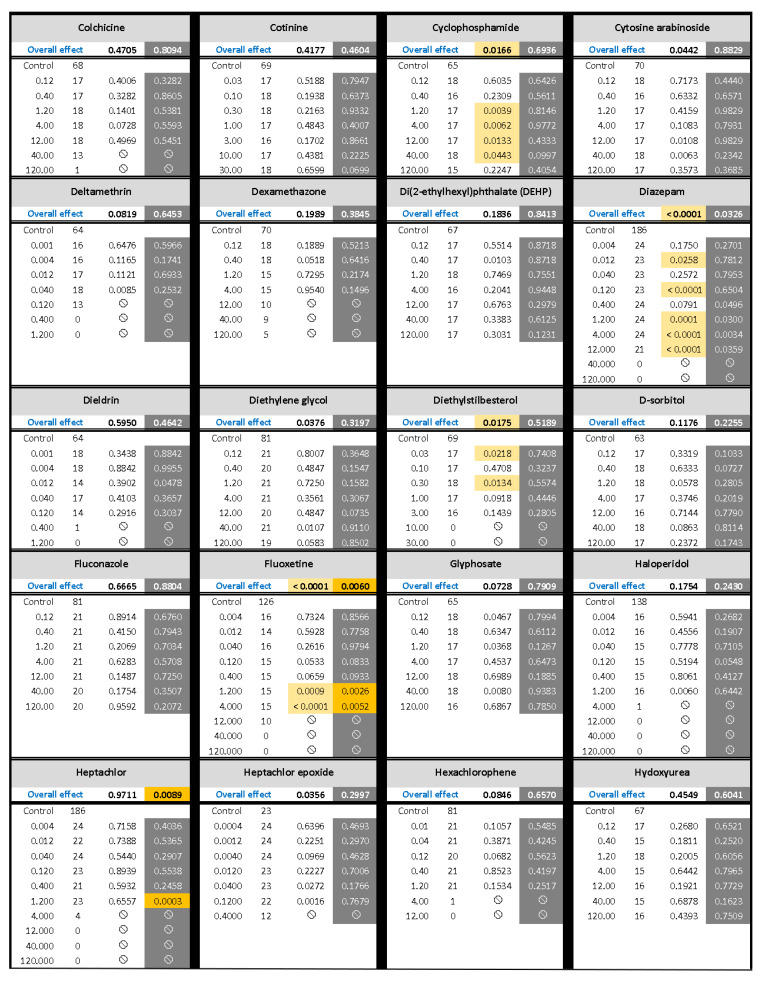

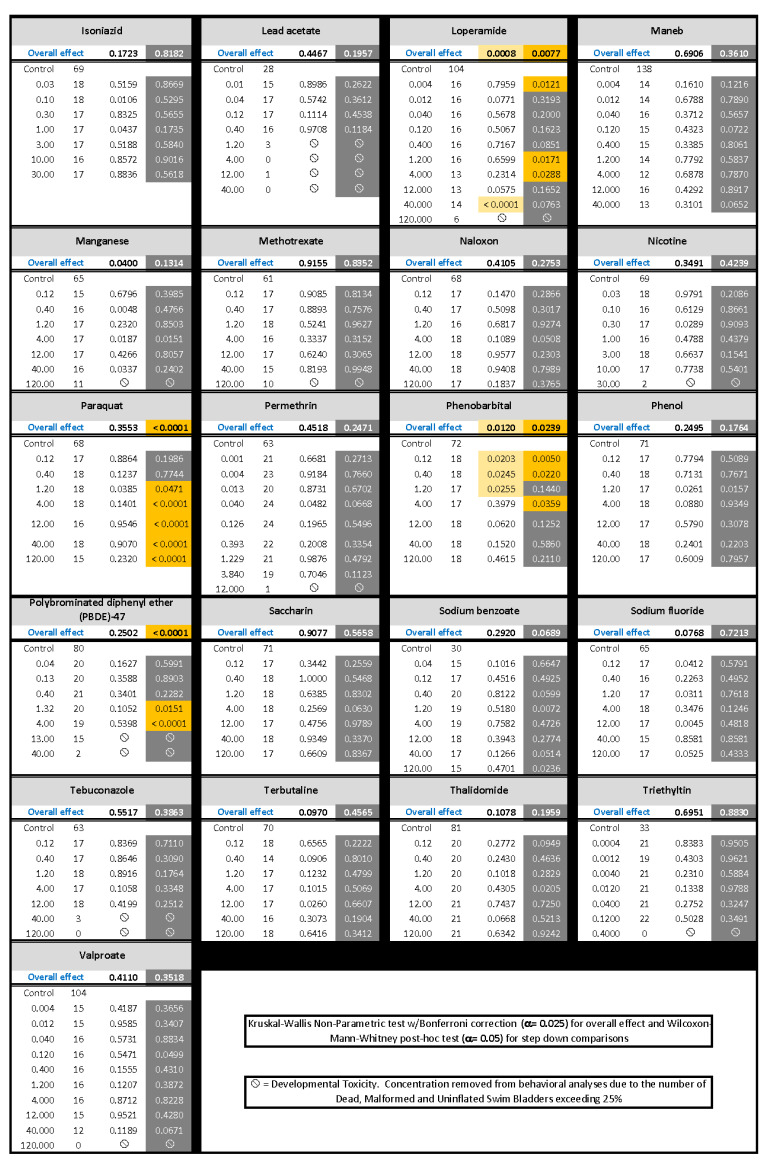
Behavioral Nonparametric Statistics Results. Results of the Kruskal-Wallis Nonparametric test for each chemical. A Bonferroni correction was applied to the overall effect to account for the Light and Dark periods, resulting in α = 0.025. The Wilcoxon-Mann-Whitney post-hoc test (α = 0.05) compared each concentration to the control for that chemical. The circle with the slash symbol (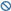) indicates developmental toxicity: that concentration was not included in behavioral analyses due to the number of dead, malformed and uninflated swim bladders exceeding 25%. Overall effect is listed under the chemical name followed by the sample size and results for each concentration, with the Dark period shaded gray. Statistically significant results are highlighted with the light-yellow shading in the Light period and dark-yellow shading in the Dark period.

The statistical results and number of larvae in every concentration group are noted in Figure 3, which also notes the concentration groups for each chemical that were excluded from behavioral analyses.

### 2.10. Analysis of Fish Movement

The videos recorded during the behavioral testing session were later analyzed using Ethovision XT (Noldus Information Technology) software Version 13 to quantify the distance moved by each larva. Tracking rate was 5 samples/sec (i.e., an image was captured every 200 ms). A dynamic subtraction method was used to detect objects that were darker than the background, with a minimum object size of 10 pixels. Tracks were analyzed for total distance moved (cm). An input filter of 0.135 cm (minimum distance moved) was used to remove system noise. All locomotion data is expressed as distance moved per segment of testing, from which total activity was calculated for each larva in both Light and Dark periods.

### 2.11. Data Analysis and Statistics

**Figure 4:**
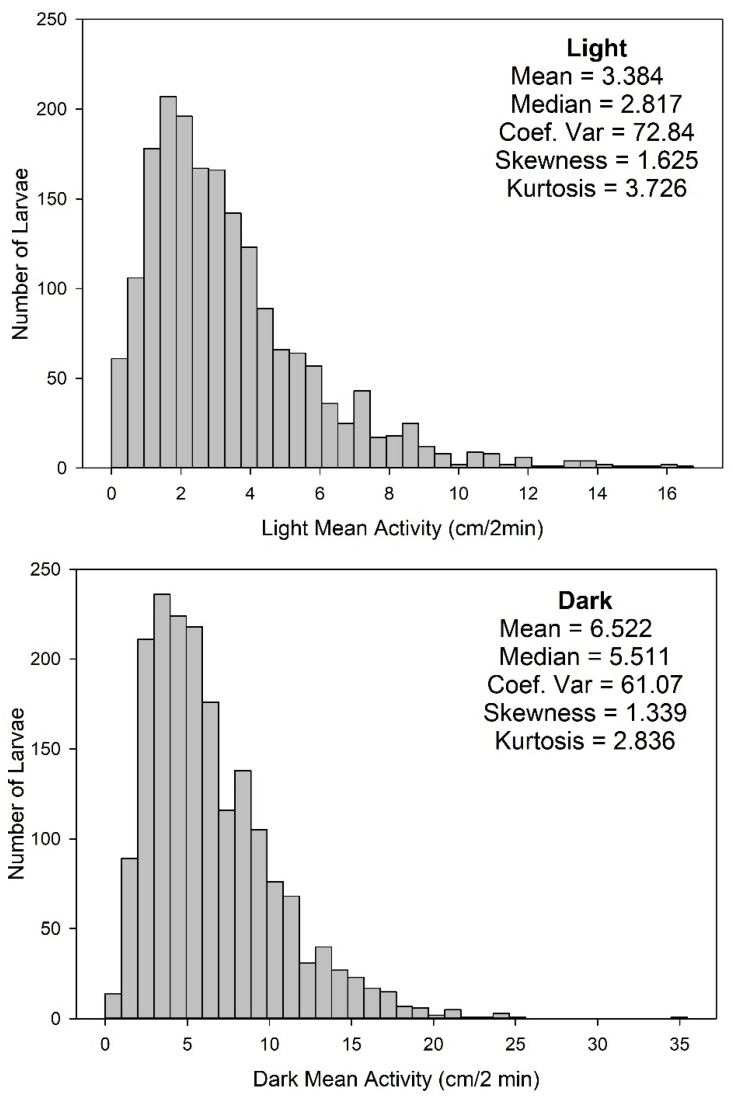
**Histograms of the Distribution of the Control Activity for the Light and Dark Periods of Testing.** The sum of the activity of the control animals (n = 1851) was plotted as a histogram to visualize the non-normal distribution of the data. Note that the activity intervals are different for the Light and Dark periods. Plots and data calculations were performed using SigmaPlot.

Under control conditions, the distributions of locomotion data were not normally distributed, but were markedly skewed (Figure 4). In the Light, there was a preponderance of low values and increasingly fewer instances of higher distance-moved values. Positive skew was also noted in the control values of distance moved during the Dark. Therefore, no “outliers” were removed, and nonparametric statistical analyses were conducted on concentration-response data (all data for each animal for the Light period or the Dark period were summed) using SAS software (v.9.4). Data were first analyzed using the Kruskal-Wallis Test assessing if there was an overall dose-response relationship between the activity in the Light or the Dark and the concentration of the test chemical. If the results of this test were significant (α ≤ 0.025 (Bonferroni corrected for the repeated measures aspect of the Light and Dark periods)) it was followed by Wilcoxon-Mann-Whitney post-hoc tests (α ≤ 0.05) that compared data for each concentration group to the vehicle-control group. 

The Kruskal-Wallis nonparametric test was also used for total activity in the Light or Dark periods to analyze the effect of developmental DMSO exposure on activity (Supplemental Figure S1). A repeated measures ANOVA was used to compare the Tower and DanioVision systems with activity as the dependent variable and system, chlorpyrifos concentration and Light/Dark as independent variables. In addition to statistical analyses, the percent change between each concentration and control was also calculated.

### 2.12. Comparison of Results with Previously Published Data

One of the goals of this study was to compare these present results to those reported in the literature. A systematic literature review was conducted (latest publication date was 30 November 2020) by gathering abstracts using the Abstract Sifter [78], searching by chemical name and/or CAS number in combination with “zebrafish” or “zebrafish and behavior” as search terms. After publications were gathered, each was further screened for methodological relevance by targeting publications that (1) specified a developmental window during chemical exposure (0–3 dpf); (2) had at least 24 h of chemical exposure; (3) included an acclimation period prior to behavioral testing; (4) conducted the behavioral test sometime between 5–7 dpf; and (5) the behavioral paradigm had at least one transition from Light to Dark. These methodological aspects were selected to focus on assays similar to our protocol. This decision was made due to the proposed influence of methodological variables on zebrafish behavior and toxicity outcome [79,80]. Information on how behavioral changes were reported, concentrations included in the dose response, and concentrations that were noted to cause significant effects were conflated into a spreadsheet and visually compared to our results.

## 3. Results

**Figure 5:**
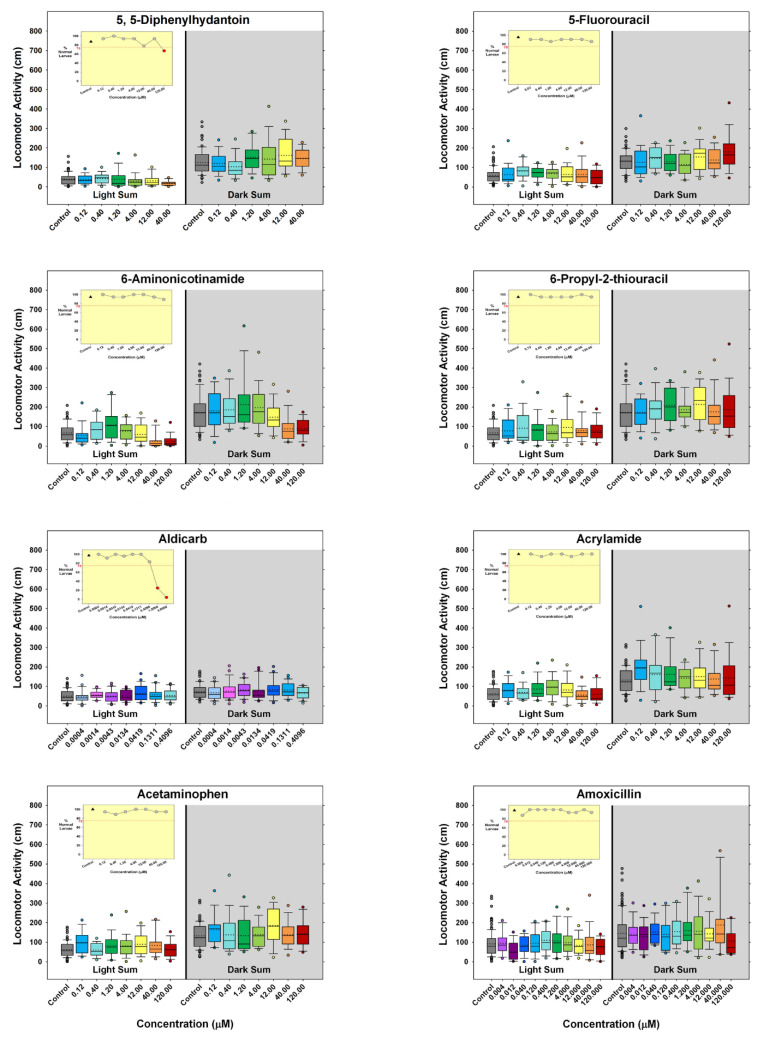

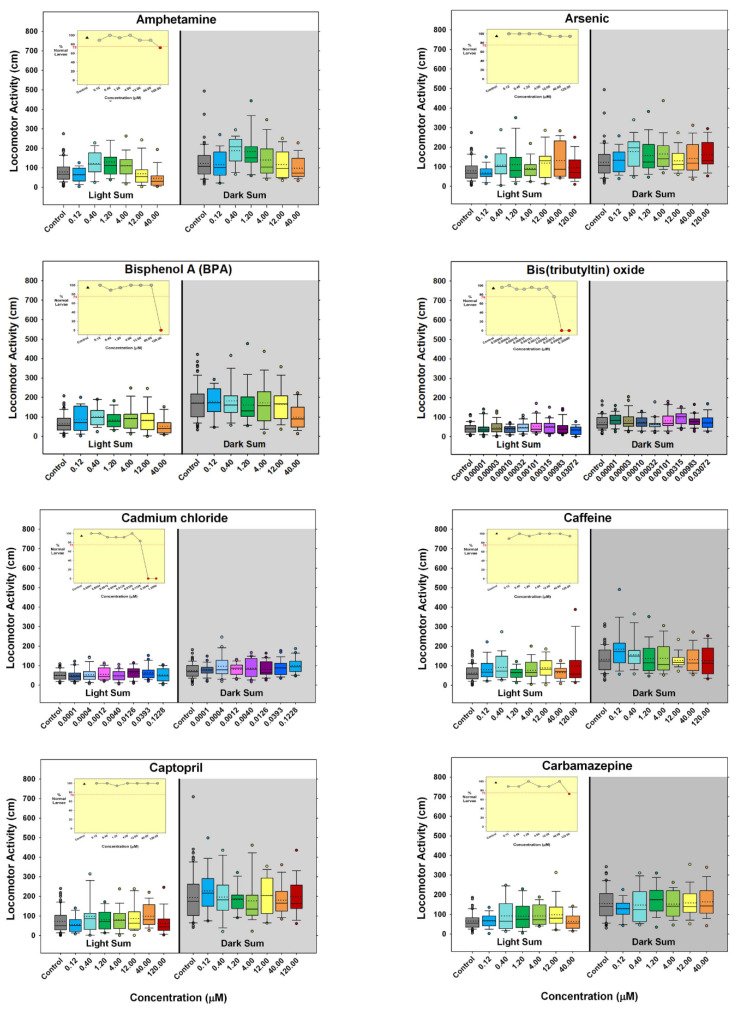

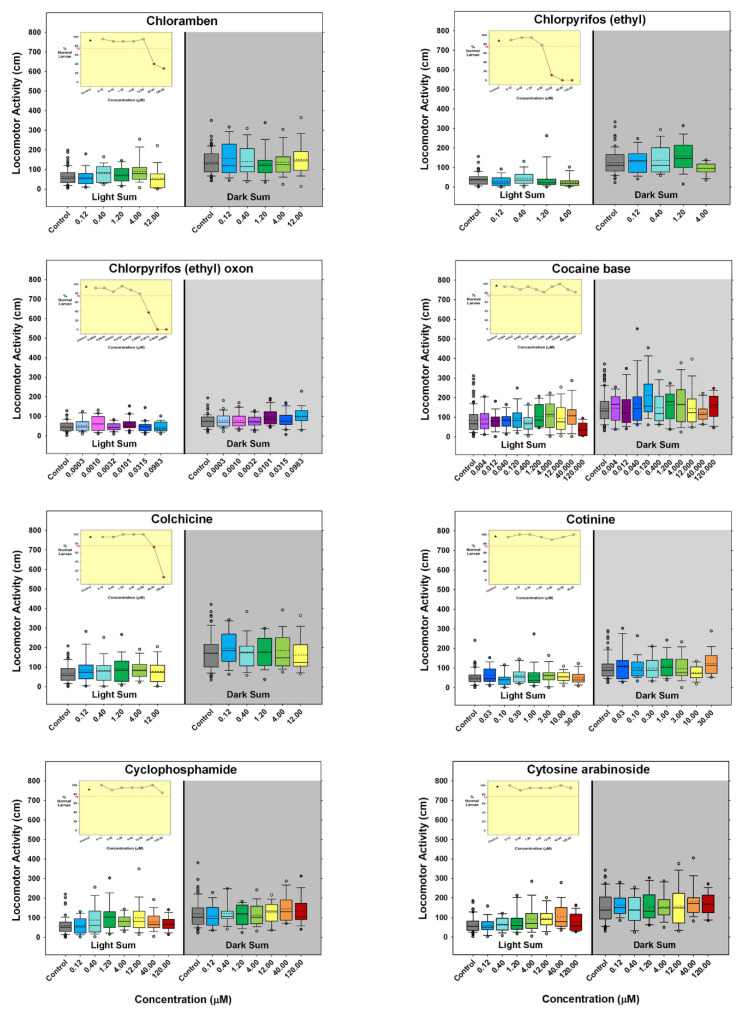

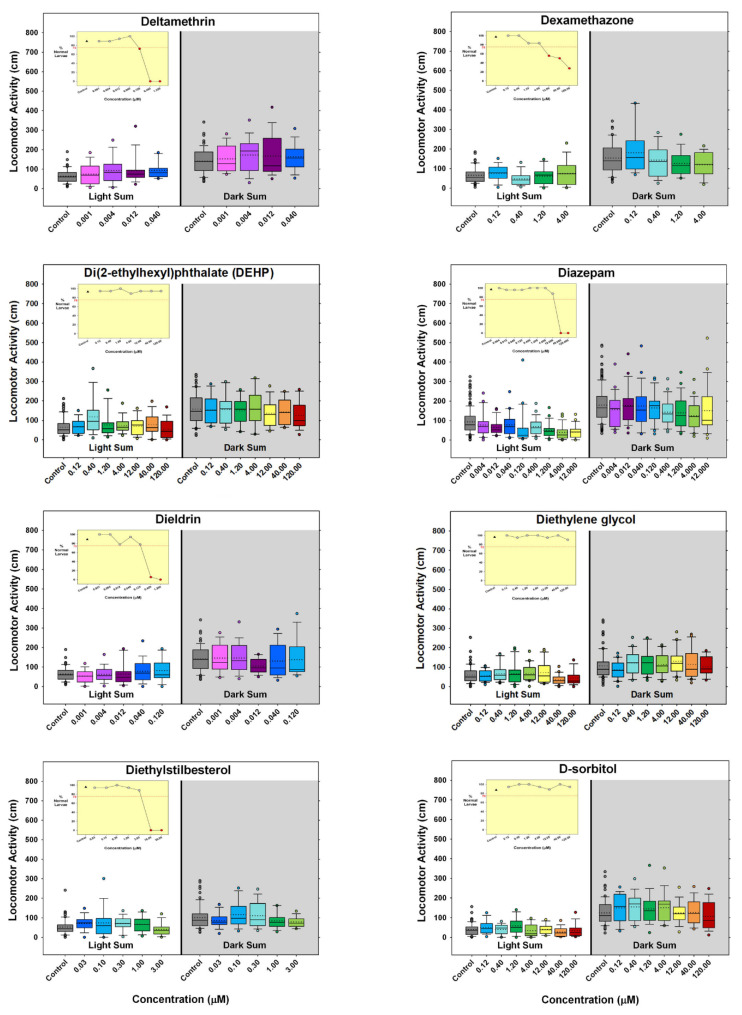

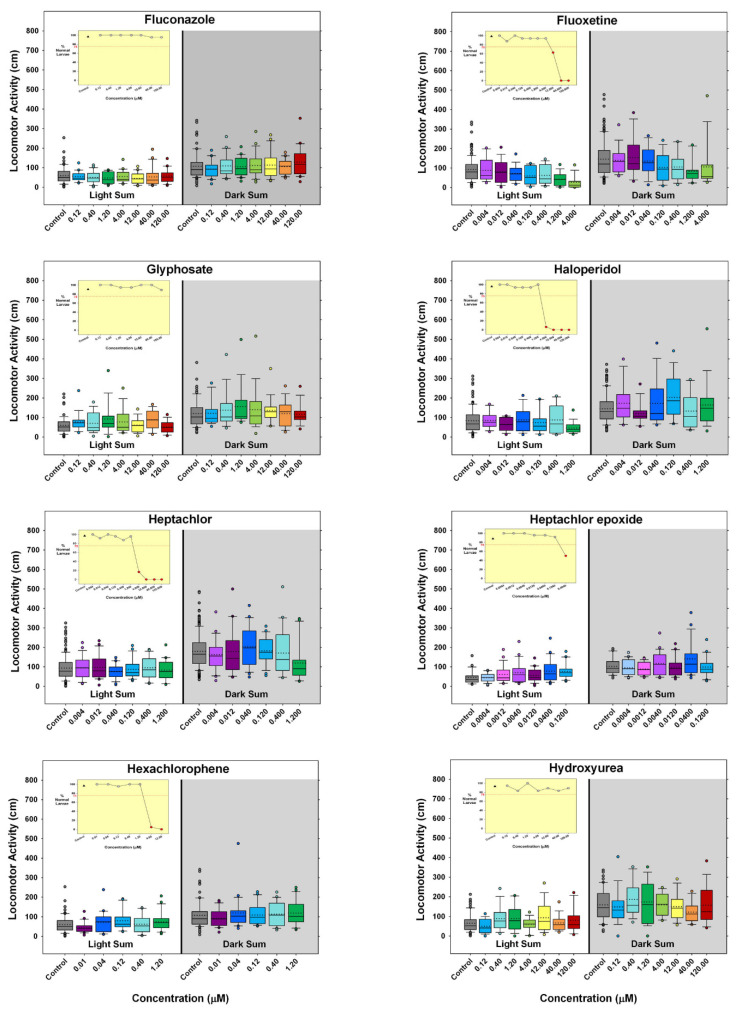

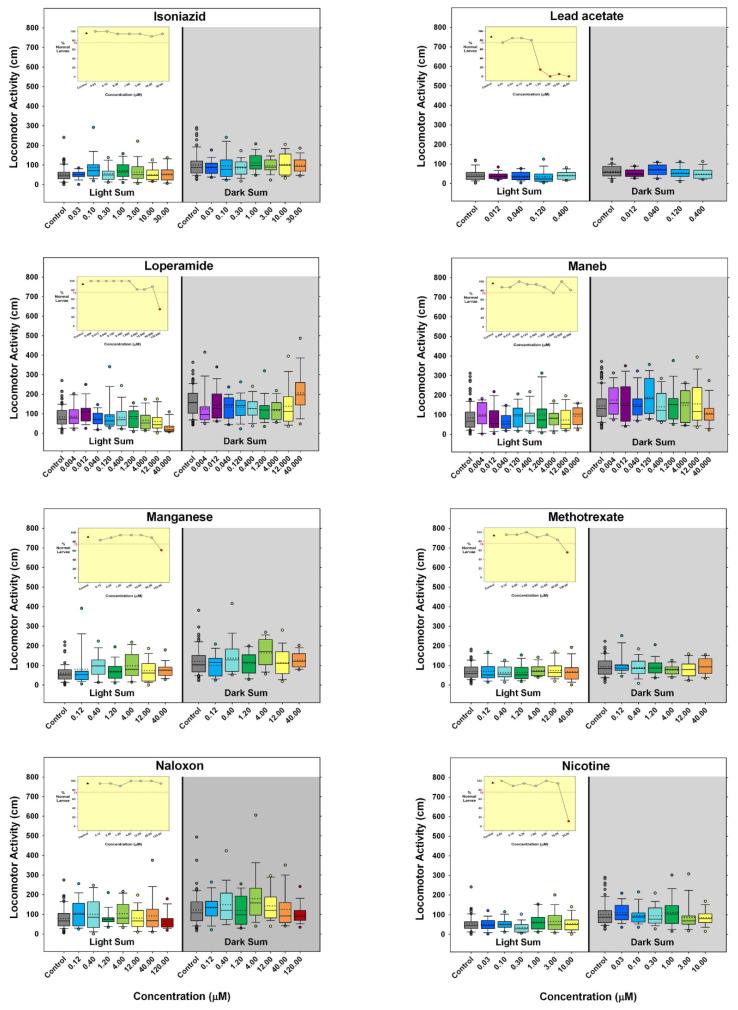

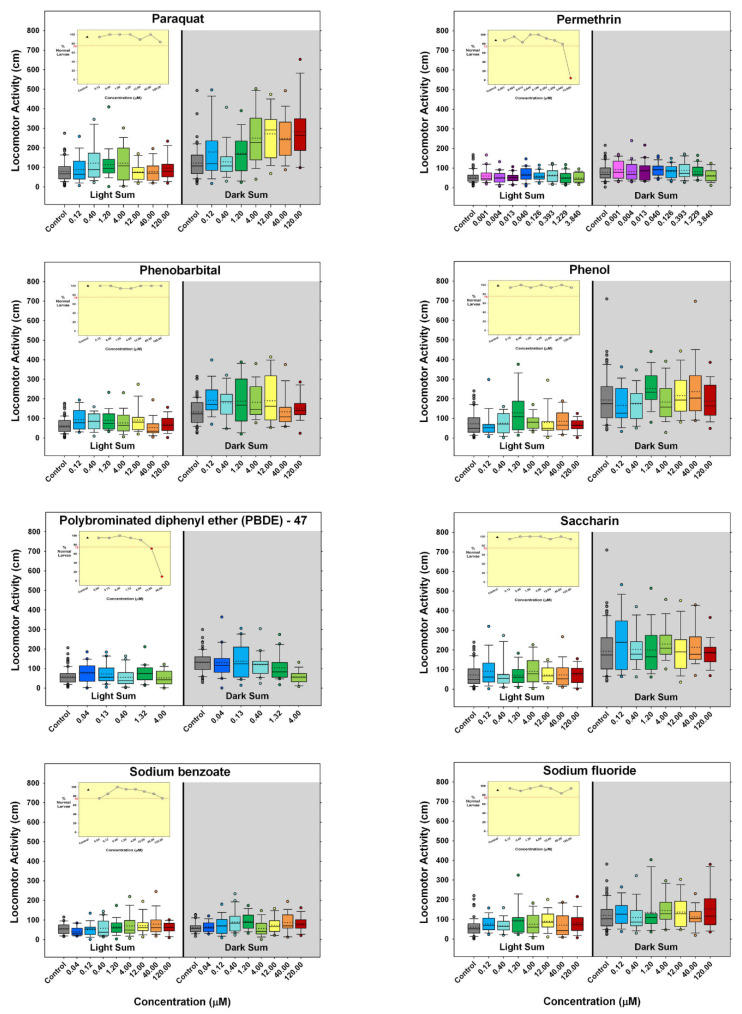

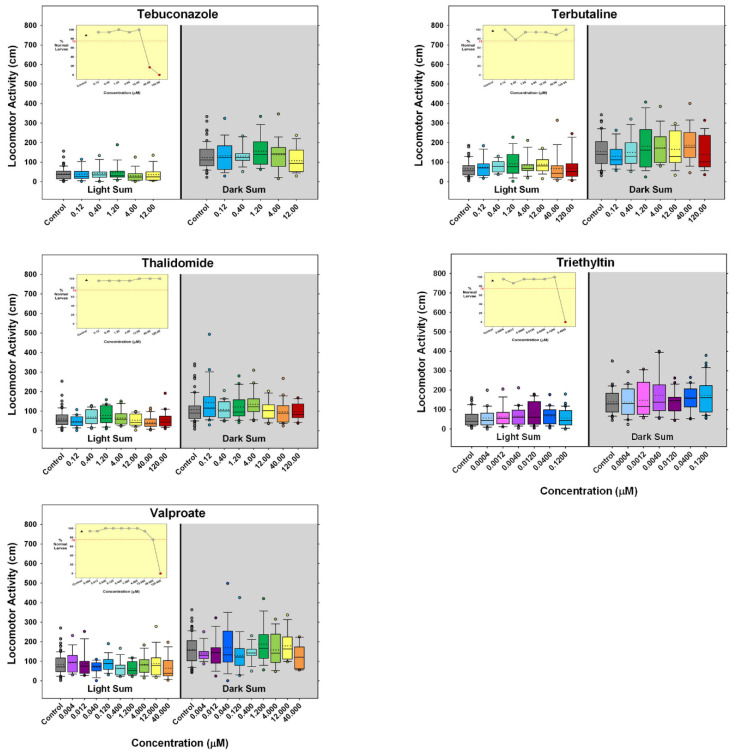
**Behavioral Concentration Response for Each Chemical Presented in a Box Plot with Developmental Toxicity as a Line Graph in the Inset.** Box plots show locomotor activity for both the Light and Dark (gray background) periods. The box represents the interquartile range (middle 50%), the top of the box to the top error bar is the upper quartile (75th percentile) while the bottom of the box to lower error bar is the lower quartile (25th percentile). The solid line in the middle of the box is the median and the dotted line in the middle of the box is the mean. The top whisker/error bar indicates the maximum and the bottom whisker/error bar indicates the minimum. The developmental toxicity inset shows the percent of normal larvae for the control and for each concentration. The dotted red line is the 75% line and concentration groups that fall below are considered developmentally toxic and not included in behavioral analyses. The triangle represents control data, and the gray circles indicate results at each concentration. All concentrations are in micromolar (µM).

Sixty-one chemicals were tested for both developmental toxicity and behavioral disruption. To determine whether developmental toxicity occurred, animals were assessed for death, non-hatching, or morphological abnormalities, including uninflated swim bladders. Normal looking embryos, such as the one depicted on Day 6 of our Experimental Design (Figure 1), have no obvious malformations, are of normal size and have an inflated swim bladder. Developmental toxicity data are shown in the inset graph on each box plot in Figure 5, and also in the summary figure for each chemical (Supplemental Figure S2). These data show the percent of normal larvae for each concentration tested. The red dashed line marks the 75% behavioral data inclusion cutoff with values that fall below that line indicated by a red circle. The black triangle represents negative control data for that chemical.

The highest concentration tested was 120 µM; if there was considerable developmental toxicity, the tested concentrations were decreased until at least four concentrations showed no developmental toxicity (i.e., the number of dead, malformed and uninflated swim bladders exceeded 25%). Of the 61 chemicals tested, approximately half (n = 30) did not show any toxicity at the highest concentration tested (Figure 4 and Figure 5). For the majority of the chemicals that did not cause toxicity, the highest concentration administered was 120 µM; however, for three chemicals (cotinine, isoniazid, maneb) the highest concentration was lower and ranged from 30–40 µM, due to solubility issues. Thirty-one chemicals did elicit developmental toxicity; twelve were toxic at the highest concentration only. For four of those twelve (heptachlor epoxide, nicotine, permethrin, triethyltin), the highest concentration tested was less than 120 µM, ranging from (0.4 to 30 µM). 

Looking at the lower concentrations, a total of thirteen chemicals were developmentally toxic at concentrations of 10 µM or lower. Seven chemicals (bis(tributyltin)oxide, cadmium chloride, chlorpyrifos oxon, deltamethrin, dieldrin, heptachlor epoxide and triethyltin) showed toxicity at the lowest range (0.1 and 1 µM). Six other chemicals (aldicarb, diethylstilbesterol, haloperidol, heptachlor, hexachlorophene, and lead acetate) were developmentally toxic in the 1 to 10 µM range. 

The developmental toxicity data on 6 dpf was used to determine which larvae would be included or removed from behavioral analyses. Concentrations with more than 25% dead or malformed larvae, and normal appearing larvae with uninflated swim bladders, were excluded from behavioral analyses. Furthermore, any individual larva that was not deemed normal was also removed from behavioral analysis, regardless of the concentration group.

The behavior data are also presented in Figure 5 as well as the supplementary summary figure (Supplementary Figure S2). Box plots showing the Light and Dark periods for each concentration were chosen to present the behavior data because of the amount of information they convey. Each box plot contains the minimum, maximum, median, and mean values, the interquartile range, the upper (75th percentile) and lower (25th percentile) quartiles, as well as outliers. Concentrations that were developmentally toxic (more than 25% of any concentration group was abnormal) appear on the inset graph, but not on the behavioral data box plot in this figure because that concentration was removed from behavior analysis. 

The behavior data are also presented in Figure 5 as well as the supplementary summary figure (Supplementary Figure S2). Box plots showing the Light and Dark periods for each concentration were chosen to present the behavior data because of the amount of information they convey. Each box plot contains the minimum, maximum, median, and mean values, the interquartile range, the upper (75th percentile) and lower (25th percentile) quartiles, as well as outliers. Concentrations that were developmentally toxic (more than 25% of the test group was abnormal) appear on the inset graph, but not on the behavioral data box plot in this figure because that concentration was removed from behavior analysis. In addition to the box plots, Supplementary Figure S2 also presents the behavior data as the mean of each 2 min epoch ± SEM. For normal behaving embryos, the 2-min data behavior pattern shows a gradual increase, then activity leveling off in the Light, followed by a characteristic sharp increase in behavior when changing from Light to Dark, which is then followed by a gradual decrease and leveling off. 

Eleven chemicals showed behavioral effects at concentrations that did not produce any developmental toxicity. For seven of them (amphetamine, diazepam, diethylstilbesterol, fluoxetine, heptachlor, loperamide, polybrominated diphenyl ether (PBDE-47)), developmental toxicity was observed at the highest concentration administered, so those concentrations were removed from behavioral analyses, and behavioral disruption in the otherwise normal looking embryos was observed at lower concentrations for those toxicants. In four chemicals (6-aminonicotinamide, cyclophosphamide, paraquat, phenobarbital) where no developmental toxicity (i.e., the number of dead, malformed and uninflated swim bladders exceeded 25%) was found at the tested concentrations, behavioral disruption was revealed.

Behavioral results showed differences for five chemicals in both the Light and Dark periods, while three (cyclophosphamide, diazepam, diethylstilbesterol) only produced effects in the Light, and three others (heptachlor, paraquat, PBDE-47) only produced behavioral effects in the Dark. Commonly, though not always, lower concentrations resulted in an increase in locomotion (hyperactivity) while higher concentrations decreased locomotion (hypoactivity). Four chemicals produced hyperactivity only, while six resulted in hypoactivity. One chemical (amphetamine) resulted in hyperactivity during both the Dark and Light periods at lower concentrations, and hypoactivity during the Light period at the highest concentration. 

**Figure 6:**
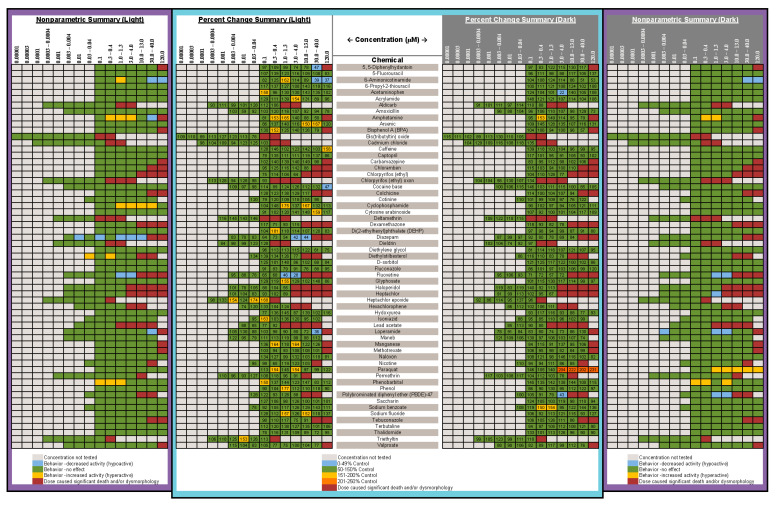
**Comparison of Nonparametric Statistical Results and Percent Change Calculations.** Comparison of the nonparametric statistical results (from Figure 3) and percent change calculations showing the degree of change in each concentration group compared to the controls. The middle column lists the chemical name, the outside columns show the nonparametric results for the Light and Dark periods, with the percent change columns next to them. Chemical concentrations are listed at the top of each column. Colored shading represents the following: light gray = concentration not tested; blue = decrease in activity; green = no effect; yellow = increase in activity; red = developmental toxicity. The percent change value is indicated in each cell and the data are color coded by 50% increments.

Results from the Kruskal-Wallis nonparametric test are listed in Figure 3. A comparison summary of the nonparametric results and percent change values are presented in Figure 6. In this Figure, the degree of change from control is identified in 50% increments, using different colors. We introduced the percent change summary as another way of looking at the data, and potentially identifying effects overlooked by traditional statistics. Overall, comparing percent change calculations to nonparametric statistical results showed that the two techniques were mostly in agreement. 

**Figure 7:**
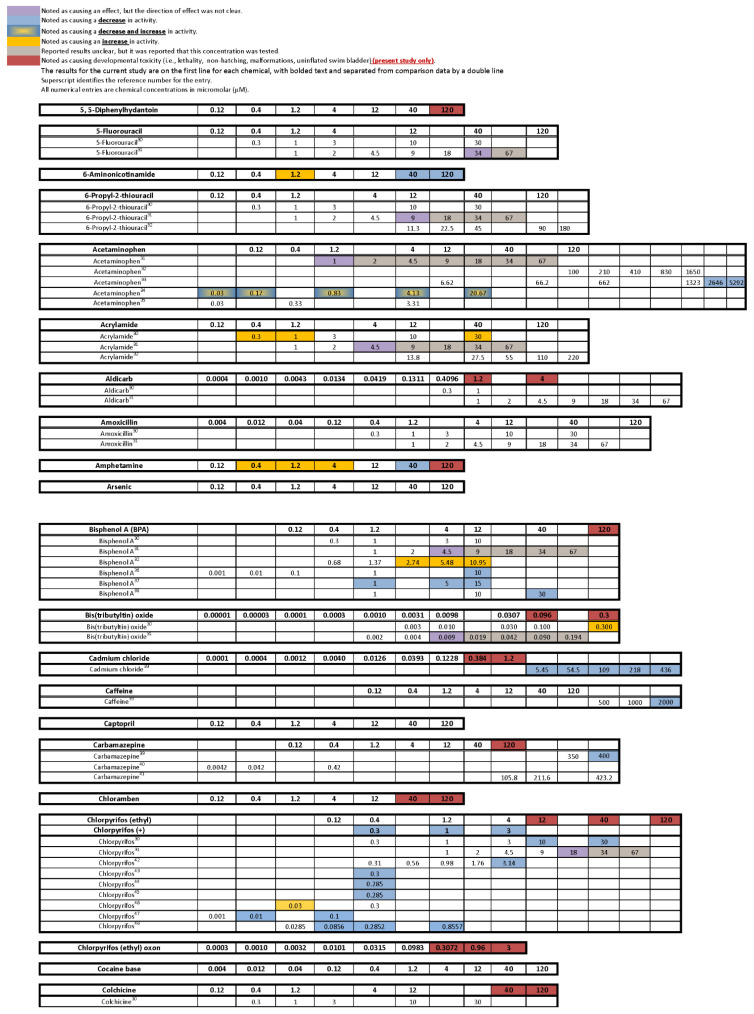

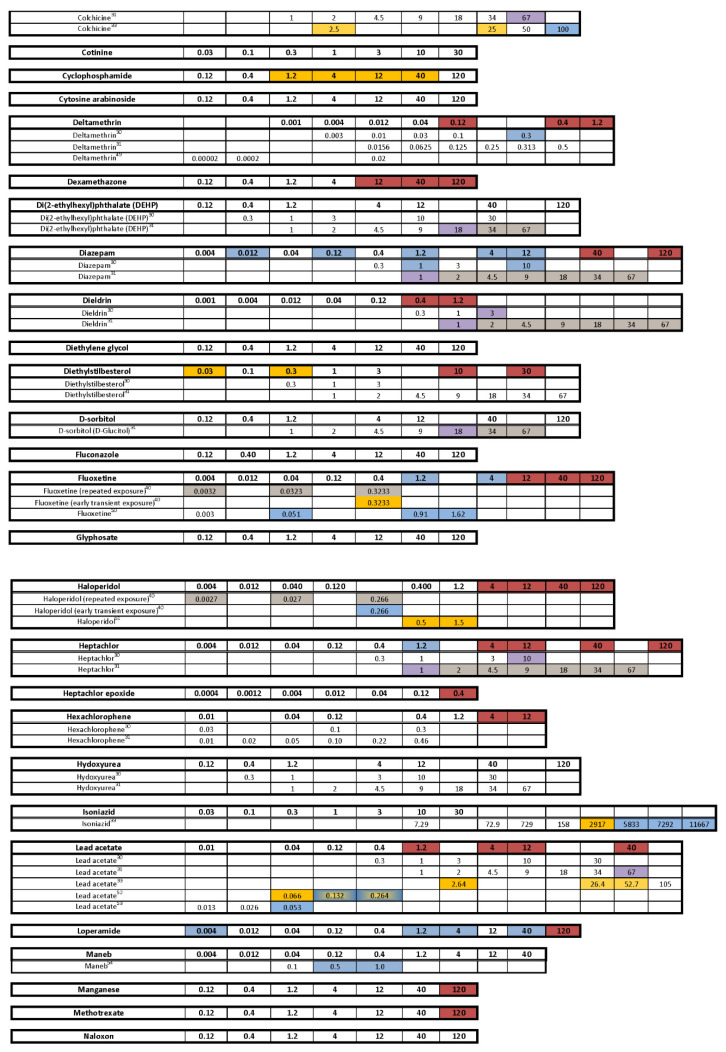

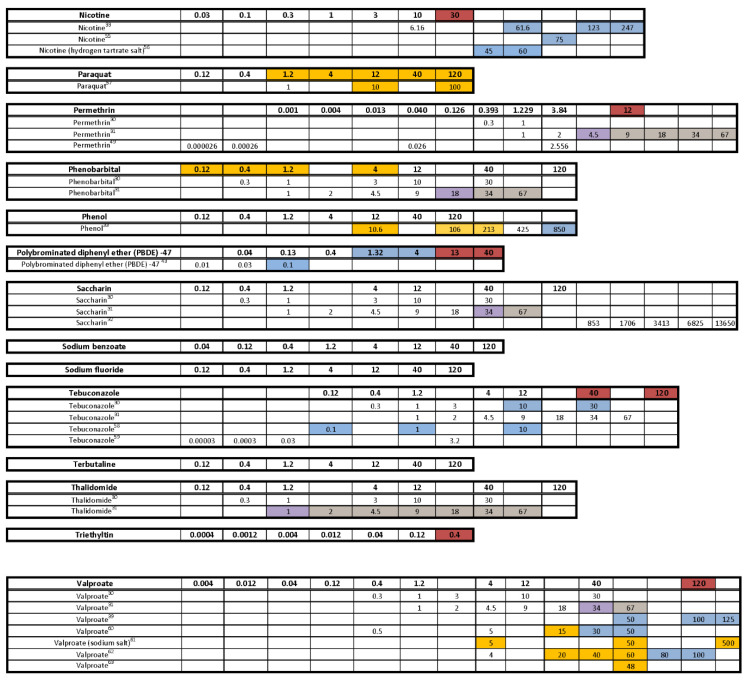
**Comparison of the Present Behavioral Results with Previous Studies from the Literature that Included Similar Experimental Conditions Testing the Same Chemicals.** To be included, all studies met the following criteria: chemical exposure began during 0–3 dpf and lasted at least 24 h; behavior was tested 5–7 dpf, included an acclimation period prior to testing and at least one transition from Light to Dark during the testing protocol. This figure was populated based on information reported by other researchers; the results were not interpreted or inferred. Some studies only reported the lowest effect dose and did not report results for other concentrations that may also have had an effect. Effects may have occurred in the acclimation, Light or Dark periods. Colored shading represents the following: blue = decrease in activity; yellow = increase in activity; blue with yellow center = both decrease and increase in activity; purple = direction of the effect could not be determined; gray = chemical concentration was tested, but results were unclear and effect could not be determined; red = developmental toxicity for the current study only. Superscript refers to publication number in the reference section of this manuscript. The results for the current study are on the first line for each chemical, with bold text. Chlorpyrifos was both a test chemical and a positive control in our study; the results for the positive control are indicated by the (+) in this figure. All concentrations are in micromolar (µM).

The comparison of our results with the results from other laboratories performing similar behavioral assays with larval zebrafish treated with the same chemicals during development is summarized in Figure 7. For 24 out of the total 61 chemicals, we were unable to find any published papers investigating the behavioral toxicity of those chemicals in larval zebrafish. We were, however, able to report information for 37 of the chemicals, and in many cases (29/37), found multiple papers that investigated the same chemical. 

## 4. Discussion

The current research evaluated a relatively large chemical library for gross developmental toxicity and behavioral effects (neurodevelopmental toxicity) following developmental exposure in embryonic/larval zebrafish. Then a subsequent comparison of our results to similar studies from other laboratories testing the same chemicals was made, and considerable variability among results was noted. We believe that part of this variability could be due to a general lack of comprehensive reporting of the experimental design and analyses. We have, therefore, endeavored to be detailed and measured in our experimental design and reporting. 

In the present experiments, we took a rigorous approach to the experimental design and analysis of the data. Regarding the experimental design, we attempted to remove any chemical from the solution the larva was reared in by replacing 100% of the solution twice before behavioral testing. If the chemical is still present during behavioral testing, it is difficult to determine whether the behavioral effects are due to the chemical’s action on brain development or are due to neuropharmacological actions. We suspect that this removal of the chemical before testing does separate developmental from pharmacological effects because when studying flame retardant chemicals [64] we obtained very different behavioral profiles depending on whether the chemicals were given acutely at the larval stage versus given during development and washed out before testing. Our approach to data analysis could also be regarded as conservative. If any concentration group presented with more than 25% abnormal animals, that entire concentration group was not included in the behavioral analysis; we believe those concentrations should be labeled as developmentally toxic. Within the concentration groups where there were ≥75% normal larvae, only larvae that presented as completely normal were included in any of the behavioral analyses. Moreover, our definition of normal appears to be stricter than some other laboratories: not only did the larva need to present without malformations, but the swim bladder had to be inflated. If the animal appeared normal with an uninflated swim bladder, that animal was not included in the behavioral analysis, as it is known that a zebrafish larva with an uninflated swim bladder does not behave normally in some assays [69,70]. In fact, if they do not inflate their swim bladder by 9 dpf, there is a high likelihood the larva will die [81]. Our approach to data analysis could also be regarded as conservative: because the data for the Light and Dark periods are not normally distributed (Figure 4), and because the number of independent observations in the control group was often more than the treated groups (Figure 3), nonparametric statistics were used. As the behavioral data in the Light and Dark periods are generated from the same animal, they are not independent observations and must be treated as repeated measures, so a Bonferroni correction was applied such that the α for the overall dose-response relationship for the Light or Dark was set to ≤0.025. Only if that overall dose response relationship was significant were step-down analyses conducted to determine which concentration groups were different from controls. In addition, because there have been admonishments in other publications to move beyond *p* values [82,83], we have included a table which shows the degree of change in each concentration group (Figure 6). This figure also includes a graphical representation of the results from the statistical analyses for comparison. The % change section of Figure 6 is color coded by 50% increments so that readers can judge for themselves about their degree of concern. 

One other issue with zebrafish behavioral data analysis that has been discussed is the issue of endpoints. In the present analyses, only two endpoints (total locomotor activity in either the Light or Dark period) are used to assess the Light/Dark locomotor response data. As the full 100 min, light/dark behavioral profiles are quite complex (Supplementary Figure S2), there are many other behavioral endpoints to be captured and analyzed (e.g., [84,85,86]). Perhaps the larval zebrafish behavioral assessment community can capture those other behavioral characteristics in an organized and consistent manner so that “behavioral barcodes” linked to modes of action can be developed, much like how the acute effects of neuroactive chemicals have been indexed to unique behavioral patterns (e.g., [87,88]). In addition to a deeper analysis of the Light/Dark locomotor assay in larval zebrafish, perhaps we should augment the larval testing battery with other behavioral assays delving into other sectors of nervous system function. Both anxiety and pre-pulse inhibition are two behavioral assessments that have been developed for larval zebrafish and associated with neuropsychiatric disorders in humans (reviewed in: [89,90])

Comparing our results to those previously published (Figure 7), we are prompted to ask some important questions:

(1) **Are there chemicals among multiple publications that consistently cause or do not cause behavioral effects? This would allow us to identify possible positive and negative controls.** There were five chemicals that appear to be candidates for positive controls: diazepam, fluoxetine, paraquat, PBDE-47, and chlorpyrifos. One publication reported decreased activity for diazepam in a similar concentration range as the present study, and the other paper reported behavioral changes, but whether it was an increase or decrease in activity was unclear as only a lowest effective dose was reported. As diazepam is known to be pharmacologically active at the gamma-aminobutyric acid receptor (reviewed in [91]), perhaps diazepam could be regarded as a positive control for GABAergic chemicals. For fluoxetine, one publication, as well as our own, reported decreased activity in larvae treated with fluoxetine during development, while another publication reported increased activity in animals treated with fluoxetine transiently during an early developmental window. Some of the effective concentration ranges aligned. As fluoxetine is a serotonin reuptake inhibitor, this chemical may serve as a positive control for the serotonergic disrupting class of chemicals. Although only one other publication tested paraquat in a developmental neurotoxicity test using zebrafish, the results were very similar to the present study, with both reporting markedly increased activity in the same dosage range. As paraquat has been reported to disrupt the development of the dopaminergic nervous system (reviewed in [92]), this chemical may serve as a positive control for the dopaminergic disrupting class of chemicals. The data for PBDE-47 as a possible positive control are a bit weaker mainly because only one other publication investigated the behavioral effects of developmental exposure to PBDE-47, and the effective concentration range did not overlap with our own data; however, both noted decreased activity. The fifth chemical that might serve as a positive control among testing publications is chlorpyrifos. There are multiple reports of developmental chlorpyrifos exposure producing behavioral alterations in larval zebrafish assays, but the range of effective concentrations spans four orders of magnitude. Because chlorpyrifos is an anticholinesterase, this chemical could serve as a positive control for the disruption of the cholinergic nervous system during development. In contrast, there are four chemicals that are candidates for negative controls, although the number of observations is smaller: aldicarb, amoxicillin, hexachlorophene and hydroxyurea. In all cases, there are two publications as well as the present study showing that developmental exposure to these chemicals in approximately the same concentration range did not produce behavioral alterations in the larval locomotor assay.
(2) **Are there chemicals that other publications have shown to produce behavioral changes after developmental exposure, but at concentrations that exceeded our concentration range or at concentrations that we deemed developmentally toxic?** Eight chemicals (aldicarb, cadmium chloride, caffeine, carbamazepine, deltamethrin, dieldrin, isoniazid, nicotine) would fall into that category. In fact, caffeine and isoniazid did not appear to produce behavioral effects unless tested in the millimolar range.
(3) **Are there unique chemicals that only our laboratory has tested that produced changes in larval locomotor activity after developmental exposure?** There were four chemicals that were tested in this publication that produced changes in locomotor activity after developmental exposure that other publications appear not to have tested: developmental exposure to 6-aminonicotinamide or loperamide produced decreased activity in the larvae, and developmental exposure to amphetamine produced an inverted “U” biphasic pattern of increased activity at lower concentrations and decreased activity at the higher concentrations. Cyclophosphamide also showed increased activity in the middle concentrations. In our laboratory embryos treated with diethylstilbesterol during development showed increased activity at concentrations below those that tested negative in other publications.
(4) **Are there chemicals that have shown behavioral effects in other studies, but were not positive in our study?** There were three chemicals, valproate, chlorpyrifos, and lead (Pb) that fall into this category. Positive results were expected for chlorpyrifos and valproate because they have tested positive previous times in our laboratory [42,62,63,64]. Specifically, we have published two papers showing developmental valproate exposure elicits behavioral changes in larval zebrafish [62,63]. It appears that the developmental toxicity profile in the present study is similar to the previously published papers: 120 µM concentration caused malformations and death in a large portion of the larvae, and 40 µM was on the cusp of developmental toxicity. The behavioral toxicity, however, was not apparent in this present study as it had been in the previous studies. The other two publications i.e., [62,63] tested about twice as many animals per concentration, so perhaps this present result is an issue of statistical power. Statistical power may have also played a role in the disparate results for chlorpyrifos in the present study. In this study we tested chlorpyrifos in two different scenarios: one as a positive control throughout the study and the other as one of the chemicals under investigation. The results are summarized for both in Figure 7 with the positive control data listed as “Chlorpyrifos+”, and the data for the test chemical listed as “Chlorpyrifos (ethyl).” As our positive control with many more observations (n = 115–132 per concentration), chlorpyrifos produced positive results in the same pattern that we often see: hypoactivity in both the Light and Dark periods, with the Dark period activity being less sensitive than the Light period activity (details in Figure 2). When testing chlorpyrifos as one of our test chemicals, however, with fewer observations (n = 14–16 per concentration), we hypothesize that there was less statistical power to detect the change. These negative results for chlorpyrifos or valproate indicate that we may need to increase the number of observations at each concentration in future developmental neurotoxicity screens. A power analysis was done when setting up our experimental design, but because the behavioral data are skewed, and require nonparametric analysis, it is difficult to perform an accurate power analysis for non-normally distributed endpoints. Lead (Pb) was another chemical where we expected a positive result given that four out of the five previous publications reported behavioral changes in larval zebrafish (Figure 7). Our results showed developmental toxicity ≥ 1.2 µM. Many of the larvae in the 1.2 and 4.0 µM concentrations showed a preponderance of uninflated swim bladders in the absence of other malformations, and therefore were not included in the behavioral assessment. If these animals had been tested in the behavioral protocol, there would have been markedly decreased activity in the Light period. One possibility to consider would be that swim bladder inflation may be a neurotoxic endpoint. Inflation of the swim bladder not only requires innervation [93,94], but it also requires a behavioral repertoire where the larva seeks out the air/water interface to take a gulp of air [95]. So perhaps swim bladder non-inflation belongs intercalated between a morphological and behavioral endpoint, and if an animal presents with an uninflated swim bladder, this could be logged as a potentially neurotoxic endpoint without behavioral confirmation.
(5) **Were there chemicals that showed considerable variation in the published results?** Four publications, including ours, tested 6-propyl-2-thiouracil with overlapping concentrations spanning about three orders of magnitude; only one publication out of the four reported changes in behavior. Six publications including our own tested acetaminophen, again with many testing in the same concentration ranges, and yet only three of the publications reporting changes in behavior. There was some overlap in the positive concentrations in two of the publications, but the third publication only found behavioral changes at millimolar concentrations. Only one out of four publications found that developmental carbamazepine produced behavioral alterations in larval zebrafish tested in the Light/Dark transition assay. For deltamethrin, only one out of four publications found behavioral changes, whereas our laboratory reported developmental toxicity in the concentration range where the behavioral changes were reported. Three out of four publications did not find behavioral alterations after developmental saccharin exposure. Interestingly, saccharin is one of the few chemicals in this testing library that was classified as a ”favorable” negative control chemical for developmental neurotoxicity screens [96], meaning that an expert panel’s assessment of the chemical showed very little to no evidence that the chemical produces developmental neurotoxicity. Five publications studied the effects of tebuconazole on behavioral profiles in larval zebrafish with two publications reporting a positive result, and the other three publications testing in that same concentration range reported negative results. There were also contrasting results with thalidomide, where one out of three publications reported behavioral changes, but the other two publications reported a negative result in the same concentration range. These types of discrepancies indicate that the zebrafish larval Light/Dark locomotor assay will require more protocol and analysis standardization among laboratories.

Even though an effort was made to target similar assays for composing the summary in Figure 7, differences among the assay procedures and analyses could lead to the differing results. A lack of standardized reporting of specific experimental conditions created challenges in cataloging the results. Surprisingly, many experimental factors such as age, temperature, duration of chemical exposure, presence/absence of chemical during testing or presence/absence of the chorion were not specified in many publications. Rarely were the larval assessment criteria (i.e., morphological features that classified a larva as abnormal or not) clearly specified. Lack of standardization in reporting also makes it difficult to understand the specifics of the experimental design and subsequent analyses. Even with these omissions and differences, some chemicals have been identified that appear to be consistent positives or negatives across multiple laboratories. 

In this publication we tested a relatively large group of chemicals for developmental neurotoxicity potential using a zebrafish behavioral assay and compared our results to publications using the same chemicals and employing a similar experimental design. There appears to be considerable variability within the literature regarding larval zebrafish behavioral alterations after developmental exposure to some of the chemicals. This comparison also allowed identification of some chemicals that are consistent positives and negatives across publications and prompts us to identify ways to improve the experimental design and interpretation of the assay that we conduct in our own laboratory. As a step toward data transparency and inter-laboratory collaboration, we have included all of our raw behavioral data to allow exploration of the data by other investigators and to encourage more zebrafish behavioral data sharing in the future. 

## Figures and Tables

**Table 1:** **List of Chemicals Tested**. Each chemical is listed by row. The columns (from left to right) contain the following information on a given chemical: name; CAS number; DTXSID, molecular weight; solvent in which the chemical was dissolved; predicted median and range of water solubility; and predicted median and range of the octanol/water partition coefficient. Values obtained from https://comptox.epa.gov/dashboard/ Last Accessed: 25 January 2022.

Chemical	Cas #	DTXSID	Molecular Weight	Solvent	Water Solubility (µmol/L) Predicted Median	Water Solubility (µmol/L) Predicted Range	Octanol Water Coeff (LogKow) Predicted Median	Octanol Water Coeff (LogKow) Predicted Range
5, 5-Diphenylhydantoin	57-41-0	DTXSID8020541	252.3	DMSO	5.67 × 10^4^	1.07 × 10^2^ to 6.00 × 10^6^	2.39	2.16 to 2.52
5-Fluorouracil	51-21-8	DTXSID2020634	130.1	DMSO	1.44 × 10^5^	3.07 × 10^4^ to 3.69 × 10^6^	−0.906	−1.37 to −0.810
6-Aminonicotinamide	329-89-5	DTXSID5051446	137.1	DMSO	6.68×10^4^	6.41 × 10^4^ to 1.28 × 10^5^	0.027	−0.730 to 0.698
6-Propyl-2-thiouracil	51-52-5	DTXSID5021209	170.2	DMSO	3.00 × 10^4^	6.93 × 10^3^ to 4.98 × 10^6^	0.523	−0.386 to 1.37
Acetaminophen	103-90-2	DTXSID2020006	151.1	DMSO	1.47 × 10^5^	3.95 × 10^4^ to 5.70 × 10^6^	0.372	0.270 to 0.462
Acrylamide	79-06-1	DTXSID5020027	71.1	DMSO	7.05 × 10^6^	2.66 × 10^6^ to 8.99 × 10^6^	−0.726	−0.810 to −0.670
Aldicarb	116-06-3	DTXSID0039223	190.3	DMSO	2.79 × 10^4^	2.55 × 10^4^ to 3.03 × 10^4^	1.13	1.13 to 1.36
Amoxicillin	26787-78-0	DTXSID3037044	365.4	DMSO	9.36 × 10^3^	5.58 × 10^3^ to 4.93 × 10^6^	0.742	0.48 to 0.97
Amphetamine	51-63-8	DTXSID2057865	184.3	H_2_O	5.70 × 10^6^	1.33 × 10^5^ to 1.13 × 10^7^	1.81	0.602 to 1.82
Arsenic	7784-46-5	DTXSID5020104	129.9	H_2_O	-	-	−3.28	−3.28
Bisphenol A (BPA)	80-05-7	DTXSID7020182	228.3	DMSO	1.00 × 10^3^	7.45 × 10^2^ to 6.76 × 10^6^	3.53	3.32 to 3.64
Bis(tributyltin) Oxide	56-35-9	DTXSID9020166	596.1	DMSO	1.5 × 10^−1^	1.5 × 10^−1^	4.05	4.05
Cadmium chloride	654054-66-7	-	183.3	DMSO	-	-	-	-
Caffeine	58-08-2	DTXSID0020232	194.2	DMSO	8.30 × 10^4^	1.36× 10^4^ to 7.14 × 10^6^	0.045	−0.131 to 0.283
Captopril	62571-86-2	DTXSID1037197	217.2	DMSO	9.47 × 10^4^	3.98 × 10^4^ to 2.46 × 10^6^	0.481	0.272 to 0.840
Carbamazepine	298-46-4	DTXSID4022731	236.3	DMSO	2.83 × 10^2^	2.55 × 10^1^ to 7.00 × 10^6^	2.37	2.25 to 2.67
Chloramben	133-90-4	DTXSID2020262	206.0	DMSO	3.40 × 10^3^	2.92 × 10^3^ to 4.68 × 10^3^	2.15	0.912 to 2.52
Chlorpyrifos (ethyl)	2921-88-2	DTXSID4020458	350.6	DMSO	2.83	1.02 to 7.00 × 10^6^	4.78	4.66 to 4.96
Chlorpyrifos (ethyl) oxon	5598-15-2	DTXSID1038666	334.5	DMSO	2.10 × 10^2^	7.76 × 10^1^ to 2.26 × 10^2^	3.32	2.89 to 3.73
Cocaine base	50-36-2	DTXSID2038443	184.3	H_2_O	4.93 × 10^6^	5.73 × 10^3^ to 9.85 × 10^6^	2.79	2.3 to 3.08
Colchicine	64-86-8	DTXSID5024845	399.4	DMSO	5.65 × 10^4^	5.25 × 10^2^ to 7.00 × 10^6^	1.2	0.920 to 1.86
Cotinine	486-56-6	DTXSID1047576	176.2	DMSO	2.99 × 10^6^	3.70 × 10^4^ to 9.02 × 10^6^	0.119	−0.228 to 0.340
Cyclophosphamide	6055-19-2	DTXSID6024888	279.1	H_2_O	1.52 × 10^5^	5.58 × 10^4^ to 8.02 × 10^6^	0.526	0.230 to 1.30
Cytosine arabinoside	147-94-4	DTXSID3022877	243.2	DMSO	4.54 × 10^5^	4.39 × 10^4^ to 8.32 × 10^6^	−2.32	−2.51 to −1.94
Deltamethrin	52918-63-5	DTXSID8020381	505.2	DMSO	1.96 × 10^−2^	1.86 × 10^−3^ to 7.00 10^6^	6.19	6.12 to 6.20
Dexamethasone	50-02-2	DTXSID3020384	392.4	DMSO	1.95 × 10^2^	1.05 × 10^2^ to 7.00 × 10^6^	1.89	1.72 to 1.92
Di(2-ethylhexyl)phthalate (DEHP)	117-81-7	DTXSID5020607	390.6	DMSO	4.23 × 10^−1^	2.90 × 10^−3^ to 7.00 × 10^6^	8.15	7.52 to 8.71
Diazepam	439-14-5	DTXSID4020406	284.7	DMSO	1.91 × 10^2^	1.07 × 10^2^ to 7.07 × 10^6^	2.91	2.70 to 2.92
Dieldrin	60-57-1	DTXSID9020453	380.9	DMSO	1.57	5.42 × 10^−1^ to 2.60	4.94	4.88 to 5.12
Diethylene Glycol	111-46-6	DTXSID8020462	106.1	DMSO	6.51 × 10^6^	5.40 × 10^6^ to 9.42 × 10^6^	−1.28	−1.51 to −1.09
Diethyl-stilbesterol	56-53-1	DTXSID3020465	268.4	DMSO	4.37 × 10^1^	1.24 × 10^1^ to 6.88 × 10^6^	5.35	4.80 to 5.93
D-sorbitol	50-70-4	DTXSID5023588	182.2	DMSO	3.31 × 10^6^	1.72 × 10^6^ to 6.07 × 10^6^	−3.15	−4.67 to −2.38
Fluconazole	86386-73-4	DTXSID3020627	306.2	DMSO	9.68 × 10^3^	1.35 × 10^3^ to 7.15 × 10^6^	0.501	0.250 to 0.698
Fluoxetine	56296-78-7	DTXSID7020635	345.8	DMSO	1.94 × 10^2^	2.37 × 10^1^ to 1.02 × 10^7^	4.09	0.768 to 4.23
Glyphosate	1071-83-6	DTXSID1024122	169.1	H_2_O	1.99 × 10^6^	6.56 × 10^4^ to 8.41 × 10^6^	−2.88	−4.47 to −2.26
Haloperidol	52-86-8	DTXSID4034150	375.9	DMSO	3.10 × 10^1^	2.34 × 10^1^ to 9.11 × 10^6^	3.84	3.01 to 4.29
Heptachlor	76-44-8	DTXSID3020679	373.3	DMSO	9.25 × 10^−2^	7.39 × 10^−2^ to 3.82 × 10^−1^	5.7	5.46 to 6.10
Heptachlor epoxide	1024-57-3	DTXSID1024126	389.3	DMSO	5.68 × 10^−1^	5.68 × 10^−1^	5.29	4.98 to 5.47
Hexachlorophene	70-30-4	DTXSID6020690	406.9	DMSO	1.73 × 10^2^	9.43× 10^−3^ to 6.66 × 10^6^	7.23	6.92 to 7.54
Hydoxy-urea	127-07-1	DTXSID6025438	76.1	DMSO	5.42 × 10^6^	2.95 × 10^6^ to 1.32 × 10^7^	−1.74	−1.80 to −1.54
Isoniazid	54-85-3	DTXSID8020755	137.1	DMSO	7.66 × 10^5^	1.22 × 10^5^ to 7.32 × 10^6^	0.754	−0.887 to −0.635
Lead acetate	6080-56-4	DTXSID3031521	379.3	H_2_O	7.77 × 10^6^	2.14 × 10^6^ to 1.34 × 10^7^	−0.285	−2.21 to −7.10 x 10^−2^
Loperamide	34552-83-5	DTXSID00880006	513.5	DMSO	4.46 × 10^6^	2.21 × 10^1^ to 8.91 × 10^6^	4.26	1.32 to 4.47
Maneb	12427-38-2	DTXSID9020794	265.3	DMSO	1.01 × 10^6^	7.72 × 10^5^ to 1.25 × 10^6^	1.4	−2.70 to 1.66
Manganese	7773-01-5	DTXSID9040681	126.0	H_2_O	-	-	-	-
Methotrexate	59-05-2	DTXSID4020822	454.4	DMSO	3.2 × 10^3^	1.89 × 10^2^ to 5.37 × 10^6^	−0.922	−1.85 to −0.241
Naloxon	51481-60-8	DTXSID90199452	399.9	H_2_O	4.06 × 10^3^	2.74 × 10^3^ to 7.99 × 10^6^	1.45	0.243 to 1.53
Nicotine	54-11-5	DTXSID1020930	162.2	DMSO	6.15 × 10^6^	8.00 × 10^4^ to 1.10 × 10^7^	0.91	0.720 to 1.17
Paraquat	1910-42-5	DTXSID7024243	257.1	H_2_O	4.88 × 10^6^	2.76 × 10^6^ to 7.00 × 10^6^	−4.58	−5.11 to −4.50
Permethrin	52645-53-1	DTXSID8022292	391.2	DMSO	1.32 × 10^−1^	2.49 × 10^−2^ to 7.00 × 10^6^	6.82	6.47 to 7.43
Phenobarbital	57-30-7	DTXSID0021123	254.2	DMSO	1.68 × 10^4^	1.02 × 10^4^ to 3.89 × 10^5^	−0.285	−2.29 to 1.13
Phenol	108-95-2	DTXSID5021124	94.1	DMSO	6.04 × 10^5^	2.78 × 10^5^ to 4.91 × 10^6^	1.5	1.46 to 1.63
Polybrominated diphenyl ether (PBDE)-47	5436-43-1	DTXSID3030056	485.8	DMSO	5.61 × 10^−3^	3.01 × 10^−3^ to 1.23 × 10^−1^	6.79	6.59 to 7.39
Saccharin	82385-42-0	DTXSID7021992	205.1	DMSO	1.91 × 10^4^	9.43 × 10^3^ to 1.85 × 10^6^	0.705	−2.01 to 0.910
Sodium benzoate	532-32-1	DTXSID1020140	144.1	H_2_O	3.32 × 10^5^	6.44 × 10^4^ to 2.84 × 10^6^	0.158	−2.27 to 1.90
Sodium fluoride	7681-49-4	DTXSID2020630	42.0	H_2_O	1.42 × 10^7^	1.42 × 10^7^	−0.77	−0.77
Tebuconazole	107534-96-3	DTXSID9032113	307.8	DMSO	1.03 × 10^2^	8.04 × 10^1^ to 7.09 × 10^6^	3.72	3.58 to 3.89
Terbutaline	23031-32-5	DTXSID3045437	274.3	DMSO	4.71.× 10^6^	4.63 × 10^4^ to 9.37 × 10^6^	0.477	0.439 to 0.523
Thalidomide	50-35-1	DTXSID9022524	258.2	DMSO	1.74 × 10^3^	6.49 × 10^2^ to 6.42 × 10^6^	0.405	−0.240 to 0.541
Triethyltin	2767-54-6	DTXSID9040712	285.8	DMSO	1.38 × 10^3^	1.38 × 10^3^	1.84	1.84
Valproate	99-66-1	DTXSID6023733	144.2	DMSO	1.99 × 10^4^	6.20 × 10^3^ to 3.33 × 10^6^	2.73	2.65 to 2.96

## Data Availability

All raw data are included in the supplementary information and will also be uploaded to https://edg.epa.gov/metadata/catalog/main/home.page.

## Supplementary Materials

The following supporting information can be downloaded at: https://www.mdpi.com/article/10.3390/toxics10050256/s1. Figure S1, Effect of DMSO on Light/Dark Locomotor Activity; Figure S2, Time Course Behavioral Graph, Activity Box Plots and Developmental Toxicity for each Chemical; Table S1, Raw Data.
