# Supplementary material for: Developmental Neurotoxicity and Behavioral Screening in Larval Zebrafish with a Comparison to Other Published Results
Source: Toxics. 2022 May 17;10(5):256. doi: 10.3390/toxics10050256 (PMC9145655; doi:10.3390/toxics10050256)
Supplement: Supplementary file 1 [file toxics-10-00256-s001.zip › Figure S1.pdf]

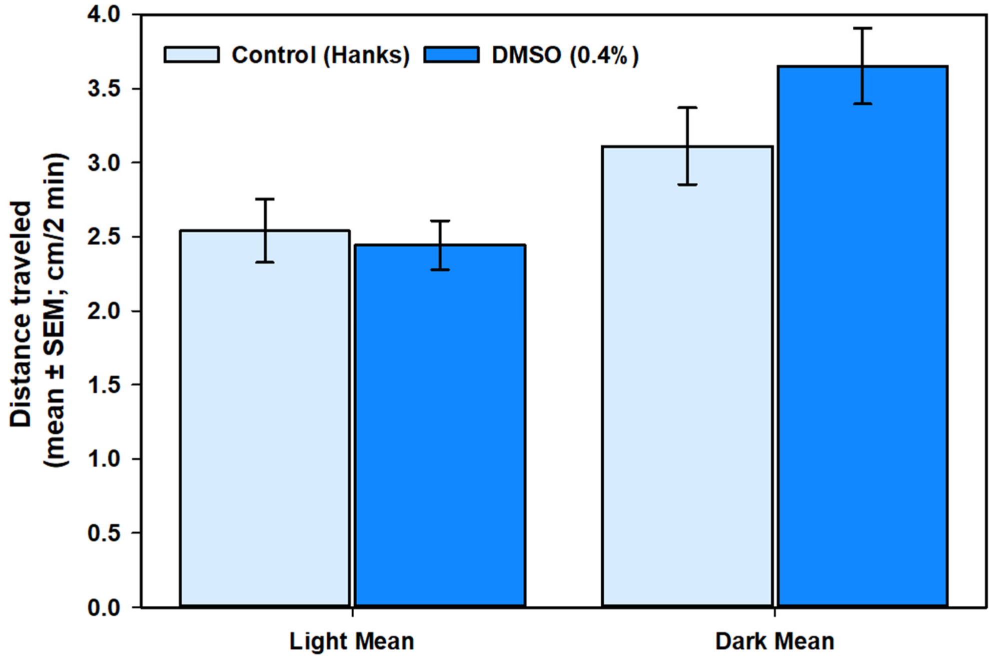

Supplemental Figure S1—Effect of DMSO on Light/Dark Locomotor Activity. The experiment was conducted under the same experimental conditions as were the chemical exposures with both DMSO exposed and non-DMSO exposed animals on the same microtiter plate. For non-DMSO exposed animals, 1 µl of water was added in place of the DMSO.
