# Supplementary material for: Developmental Neurotoxicity and Behavioral Screening in Larval Zebrafish with a Comparison to Other Published Results
Source: Toxics. 2022 May 17;10(5):256. doi: 10.3390/toxics10050256 (PMC9145655; doi:10.3390/toxics10050256)
Supplement: Supplementary file 1 [file toxics-10-00256-s001.zip › Figure S2.pdf]

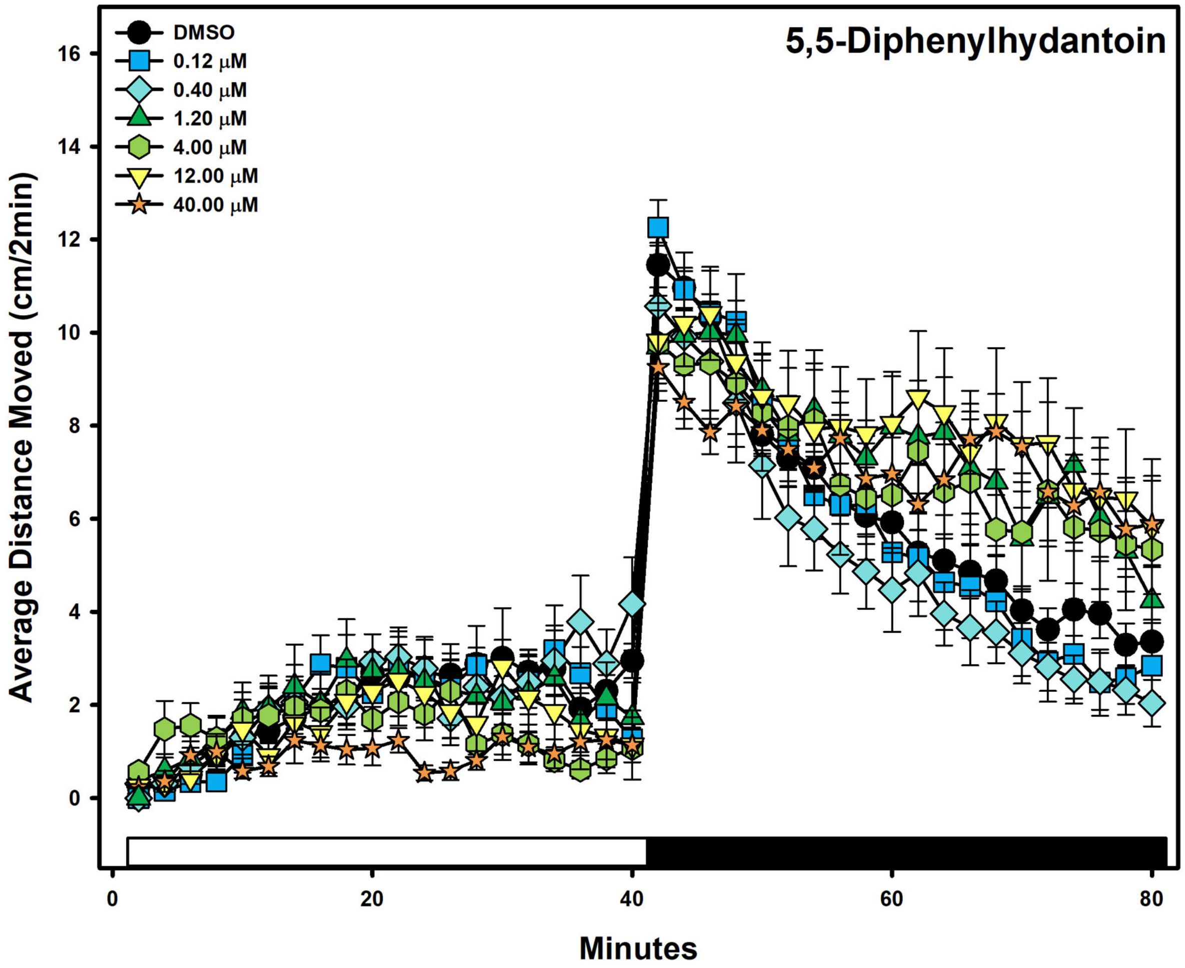

5,5-Diphenylhydantoin
DMSO
0.12 µM
0.40 µM
1.20 µM
4.00 µM
12.00 µM
40.00 µM
Average Distance Moved (cm/2min)
0
2
4
6
8
10
12
14
16
0
20
40
60
80
Minutes

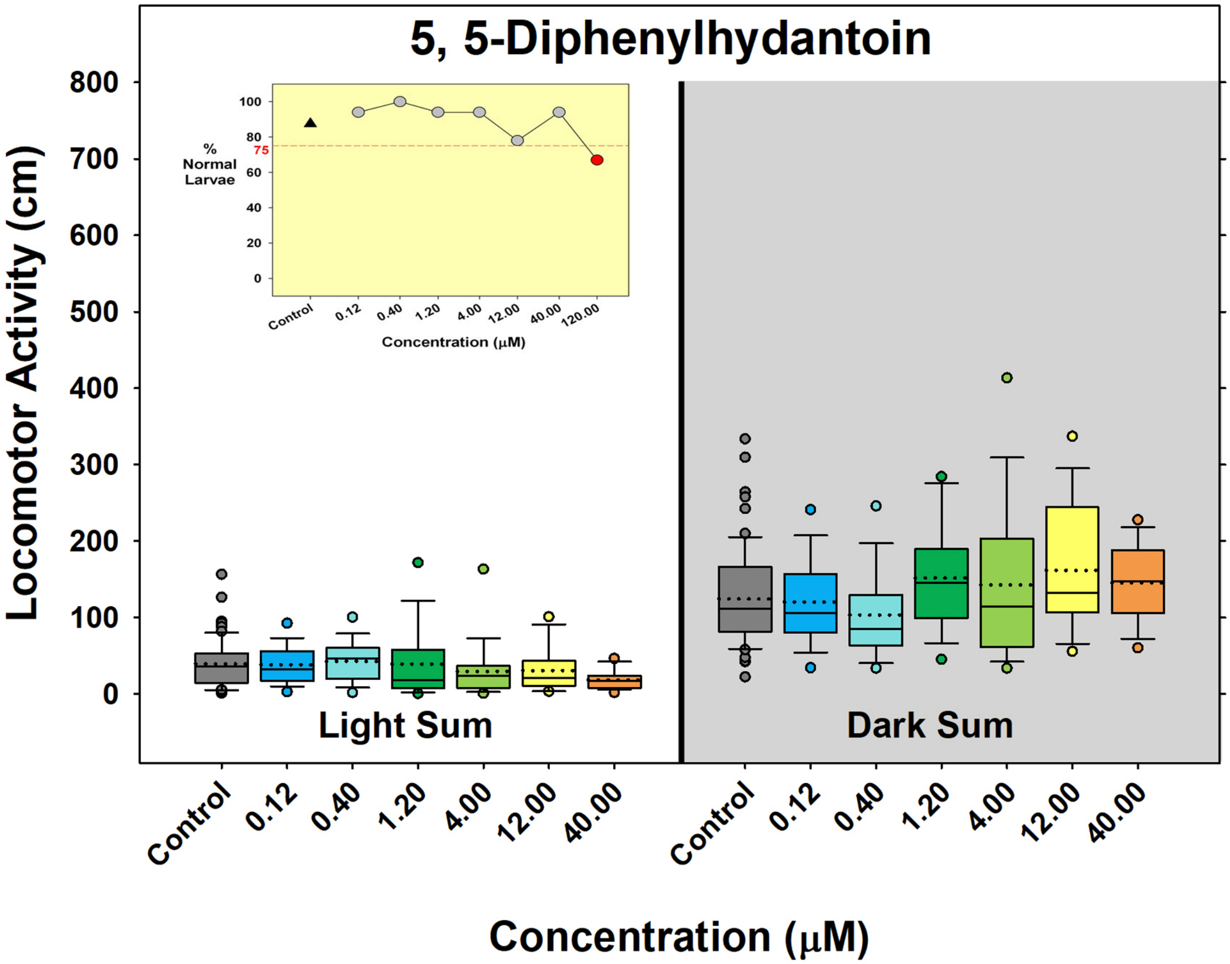

5, 5-Diphenylhydantoin
Locomotor Activity (cm)
0
100
200
300
400
500
600
700
800
% Normal Larvae
75
Control
0.12
0.40
1.20
4.00
12.00
40.00
120.00
Concentration (µM)
Light Sum
Dark Sum
Control
0.12
0.40
1.20
4.00
12.00
40.00
Control
0.12
0.40
1.20
4.00
12.00
40.00
Concentration (µM)

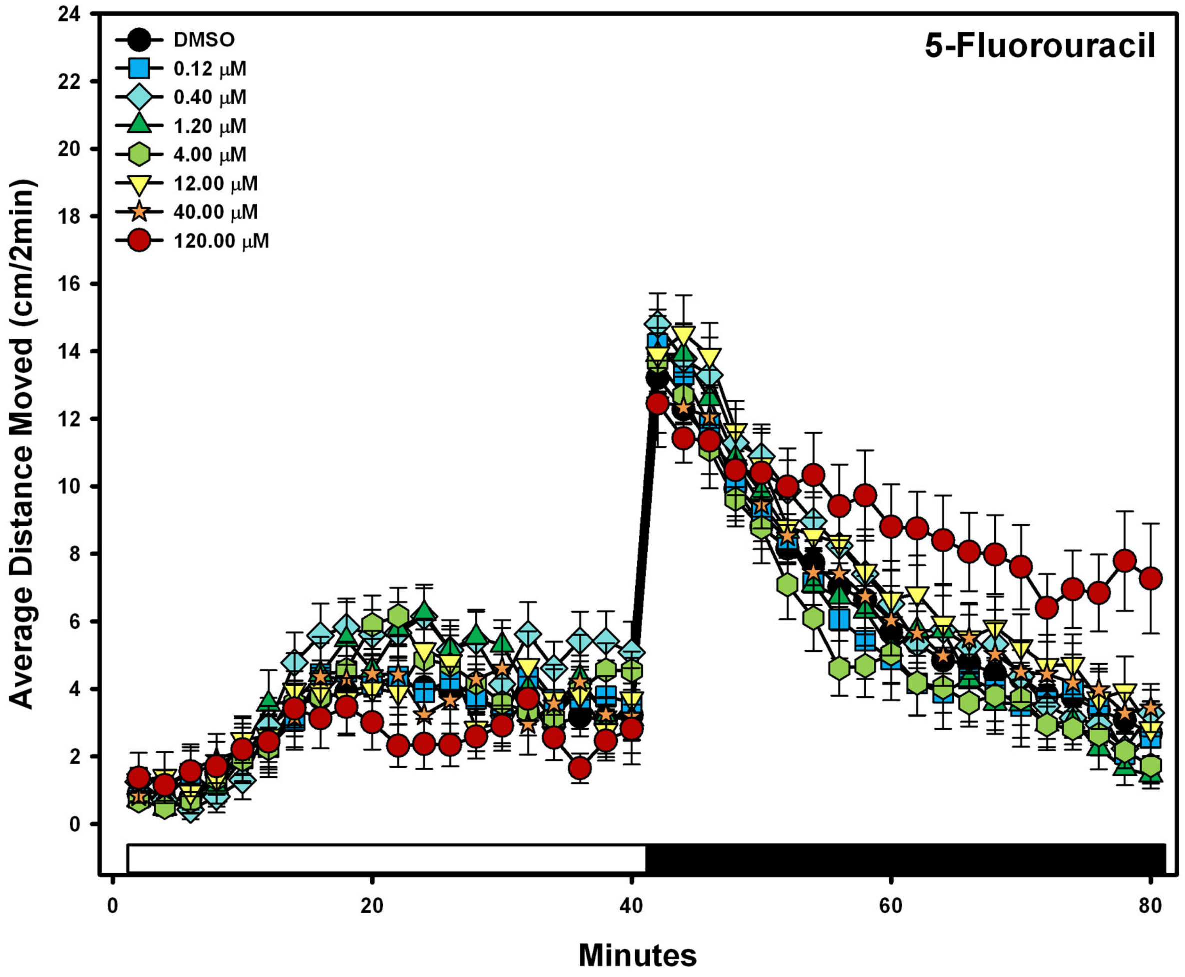

5-Fluorouracil
DMSO
0.12 μM
0.40 μM
1.20 μM
4.00 μM
12.00 μM
40.00 μM
120.00 μM
Average Distance Moved (cm/2min)
0
2
4
6
8
10
12
14
16
18
20
22
24
0
20
40
60
80
Minutes

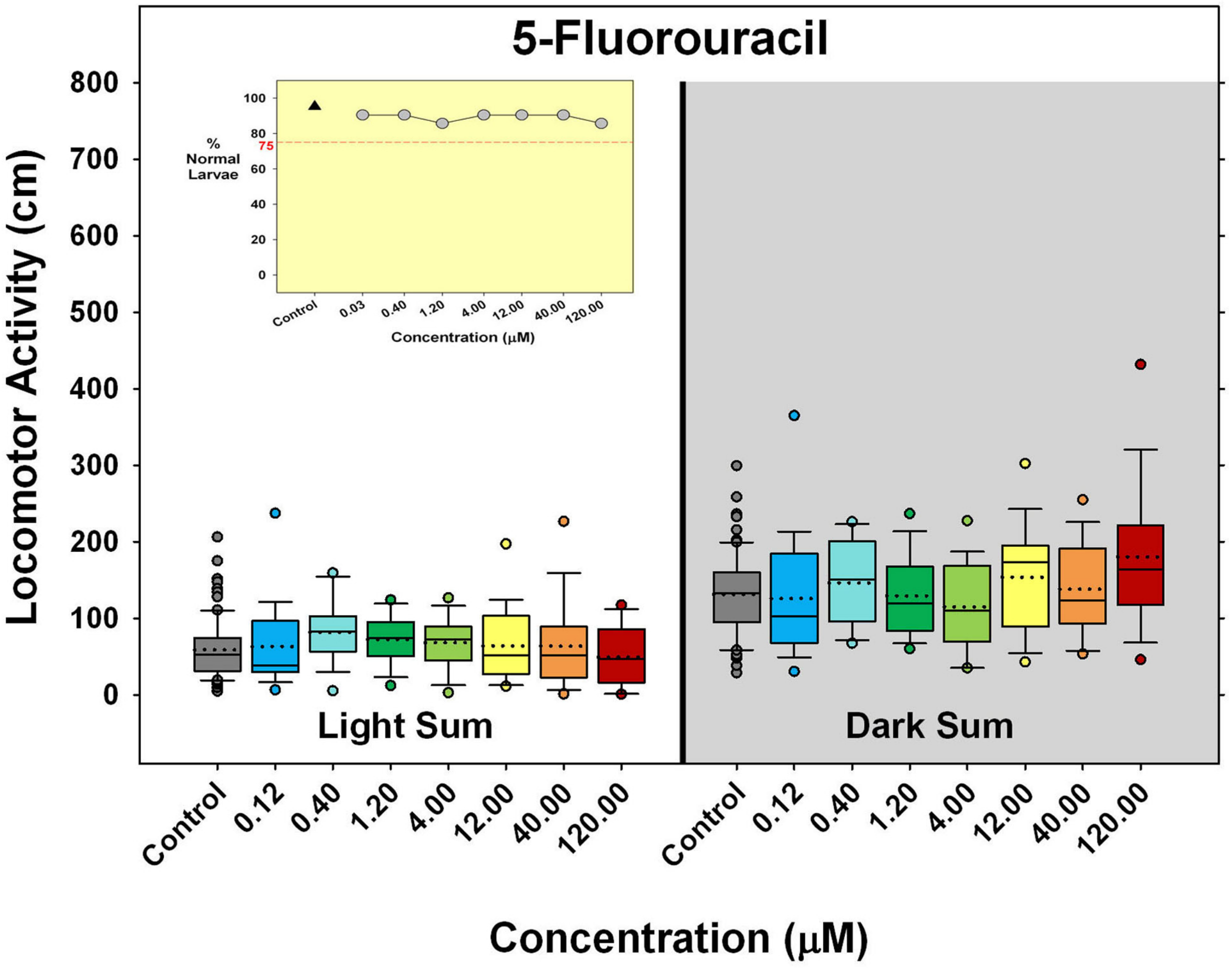

5-Fluorouracil
Locomotor Activity (cm)
0
100
200
300
400
500
600
700
800
% Normal Larvae
75
0
20
40
60
80
100
Control
0.03
0.40
1.20
4.00
12.00
40.00
120.00
Concentration (μM)
Light Sum
Dark Sum
Control
0.12
0.40
1.20
4.00
12.00
40.00
120.00
Control
0.12
0.40
1.20
4.00
12.00
40.00
120.00
Concentration (μM)

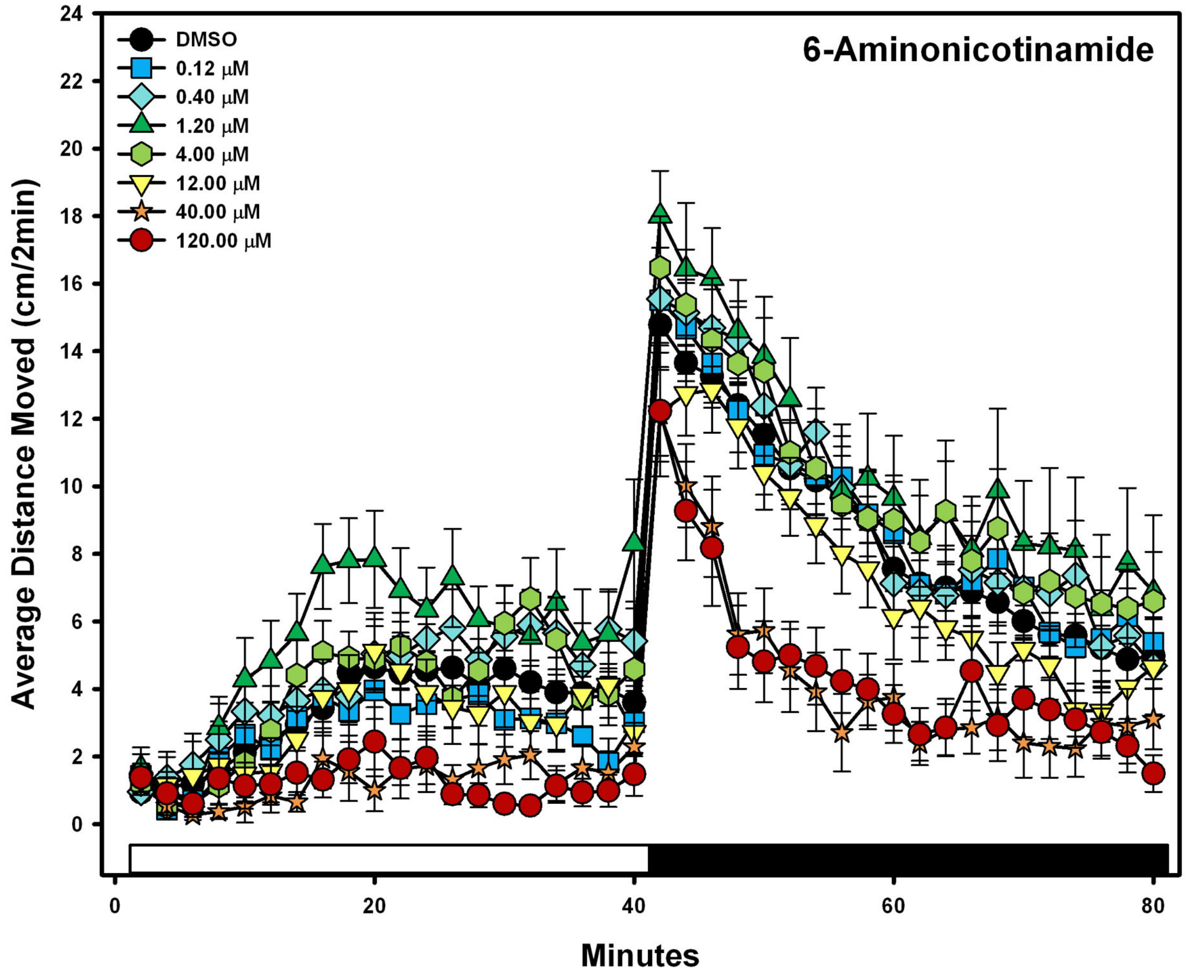
6-Aminonicotinamide
DMSO
0.12 µM
0.40 µM
1.20 µM
4.00 µM
12.00 µM
40.00 µM
120.00 µM
Average Distance Moved (cm/2min)
Minutes

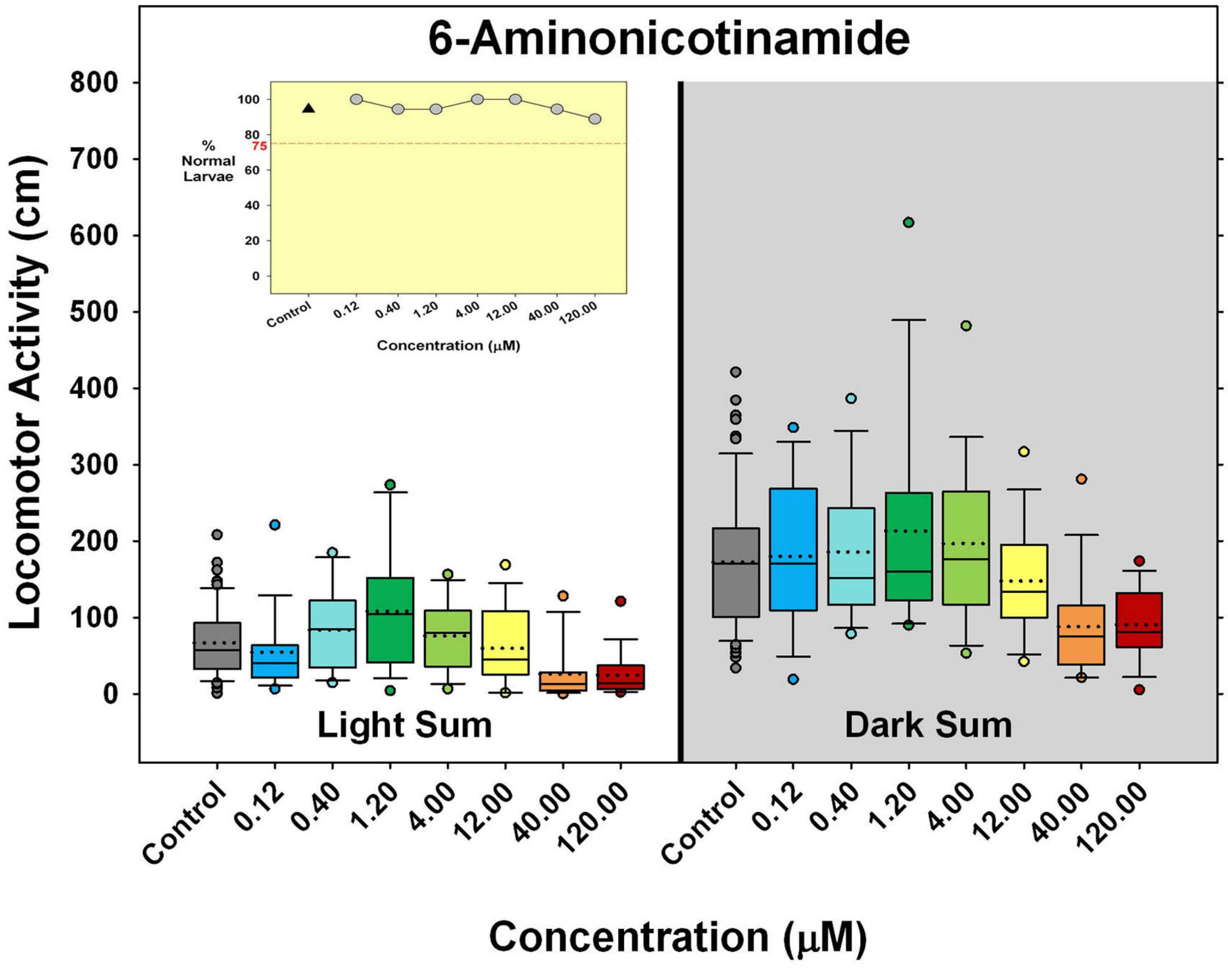
6-Aminonicotinamide
Locomotor Activity (cm)
% Normal Larvae
75
Concentration (µM)
Control
0.12
0.40
1.20
4.00
12.00
40.00
120.00
Light Sum
Dark Sum
Concentration (µM)

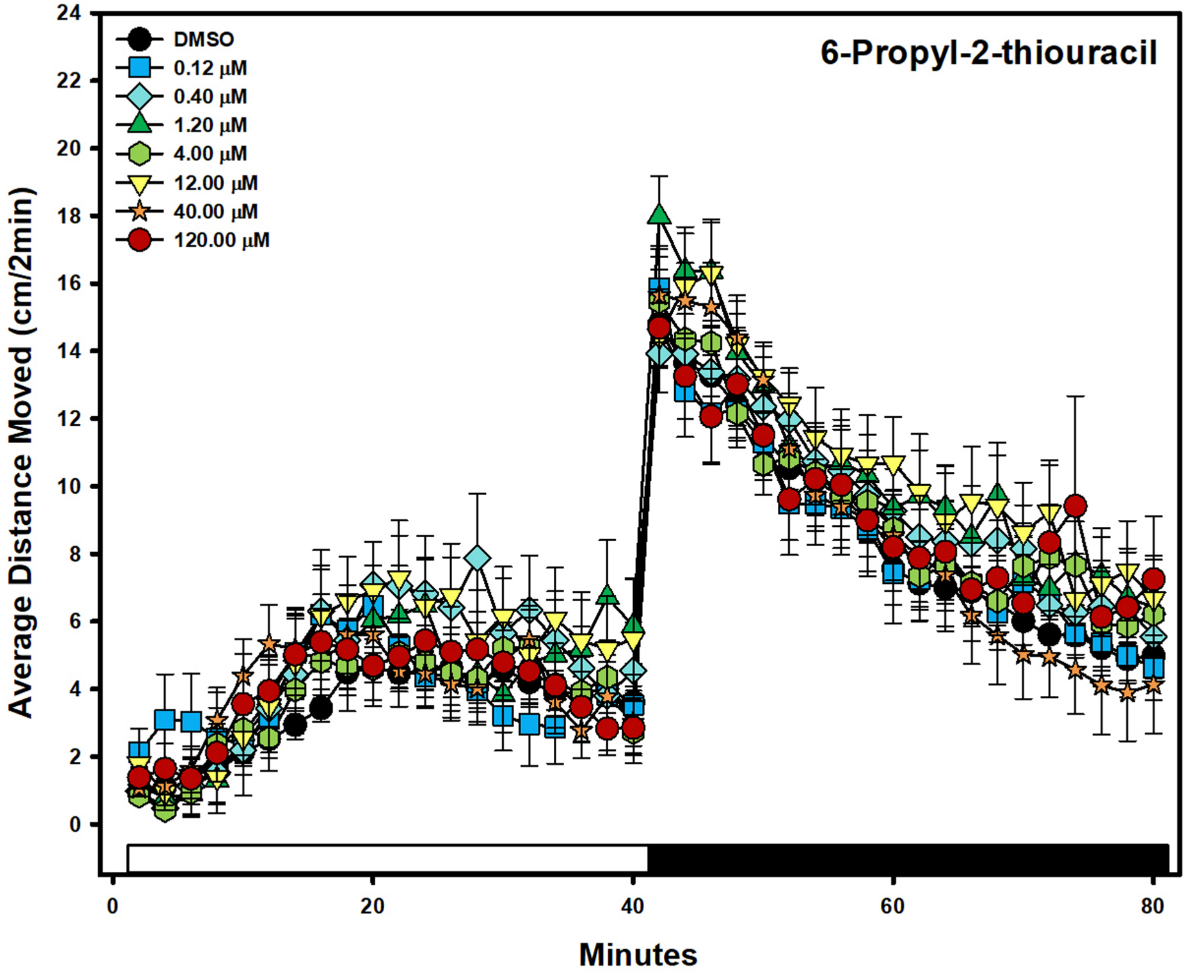
6-Propyl-2-thiouracil
DMSO
0.12 µM
0.40 µM
1.20 µM
4.00 µM
12.00 µM
40.00 µM
120.00 µM
Average Distance Moved (cm/2min)
Minutes

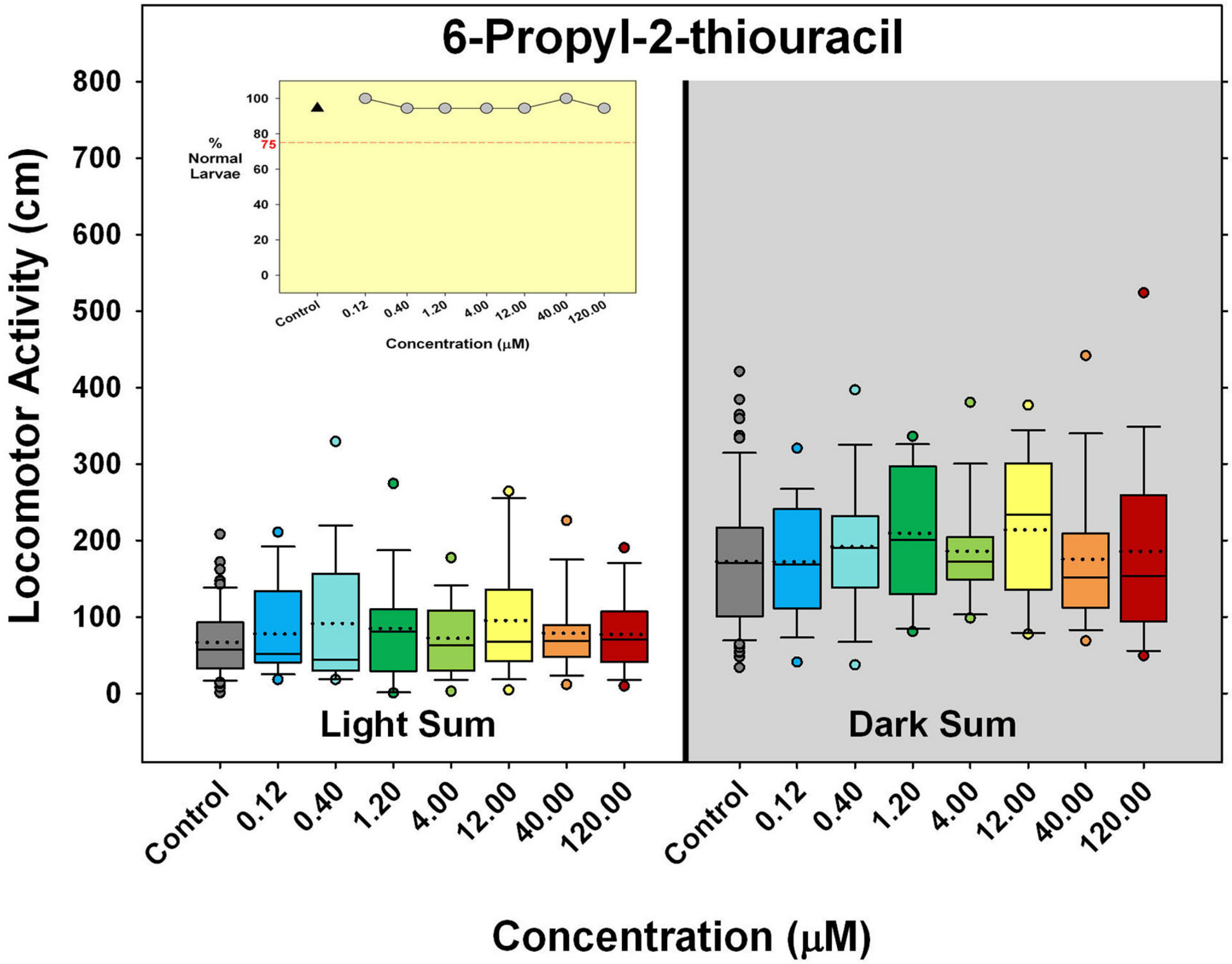
6-Propyl-2-thiouracil
Locomotor Activity (cm)
% Normal Larvae
Concentration (µM)
Control
0.12
0.40
1.20
4.00
12.00
40.00
120.00
Light Sum
Dark Sum
Concentration (µM)

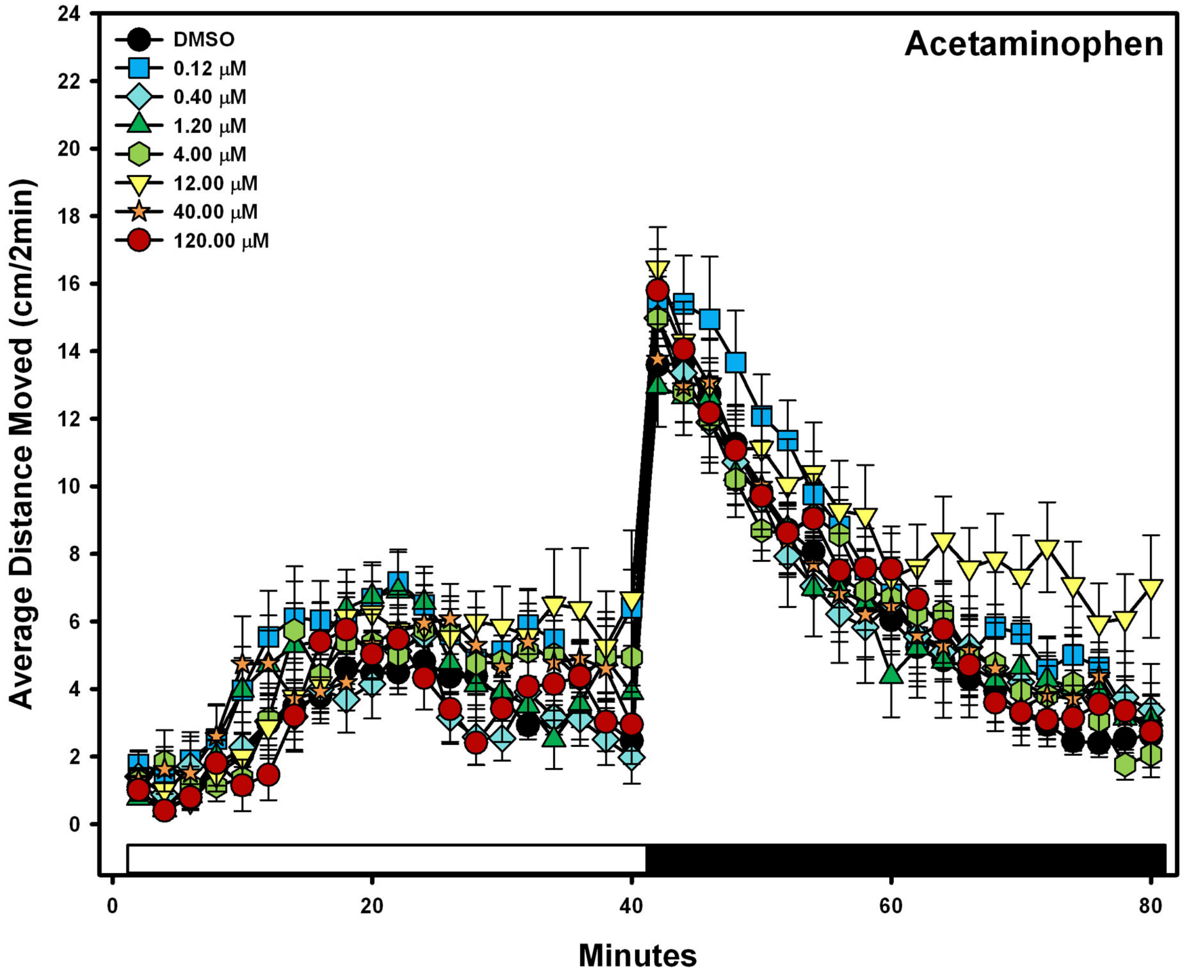
Acetaminophen
DMSO
0.12 μM
0.40 μM
1.20 μM
4.00 μM
12.00 μM
40.00 μM
120.00 μM
Average Distance Moved (cm/2min)
Minutes

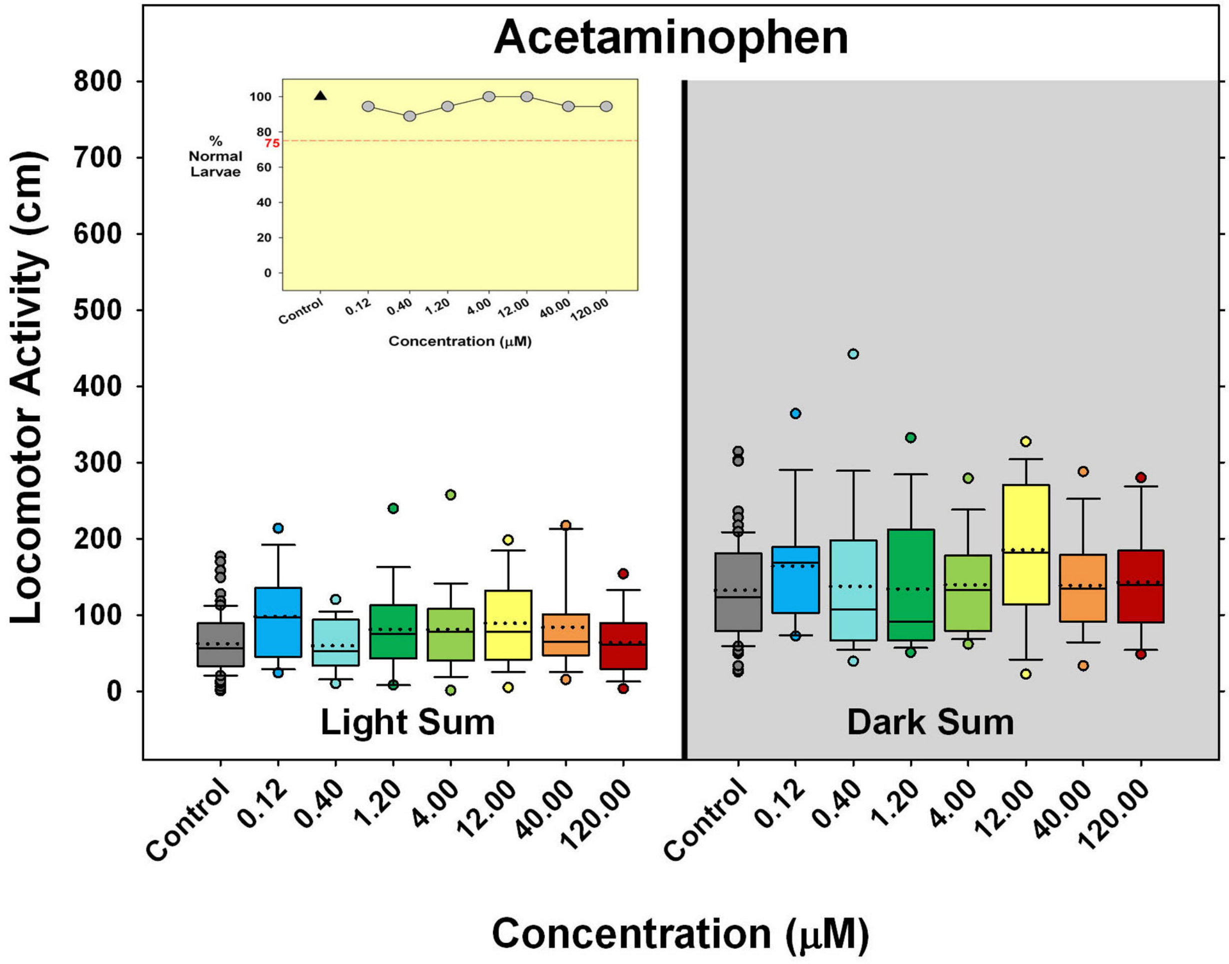
Acetaminophen
Locomotor Activity (cm)
% Normal Larvae
Concentration (μM)
Light Sum
Dark Sum
Control
0.12
0.40
1.20
4.00
12.00
40.00
120.00
Concentration (μM)

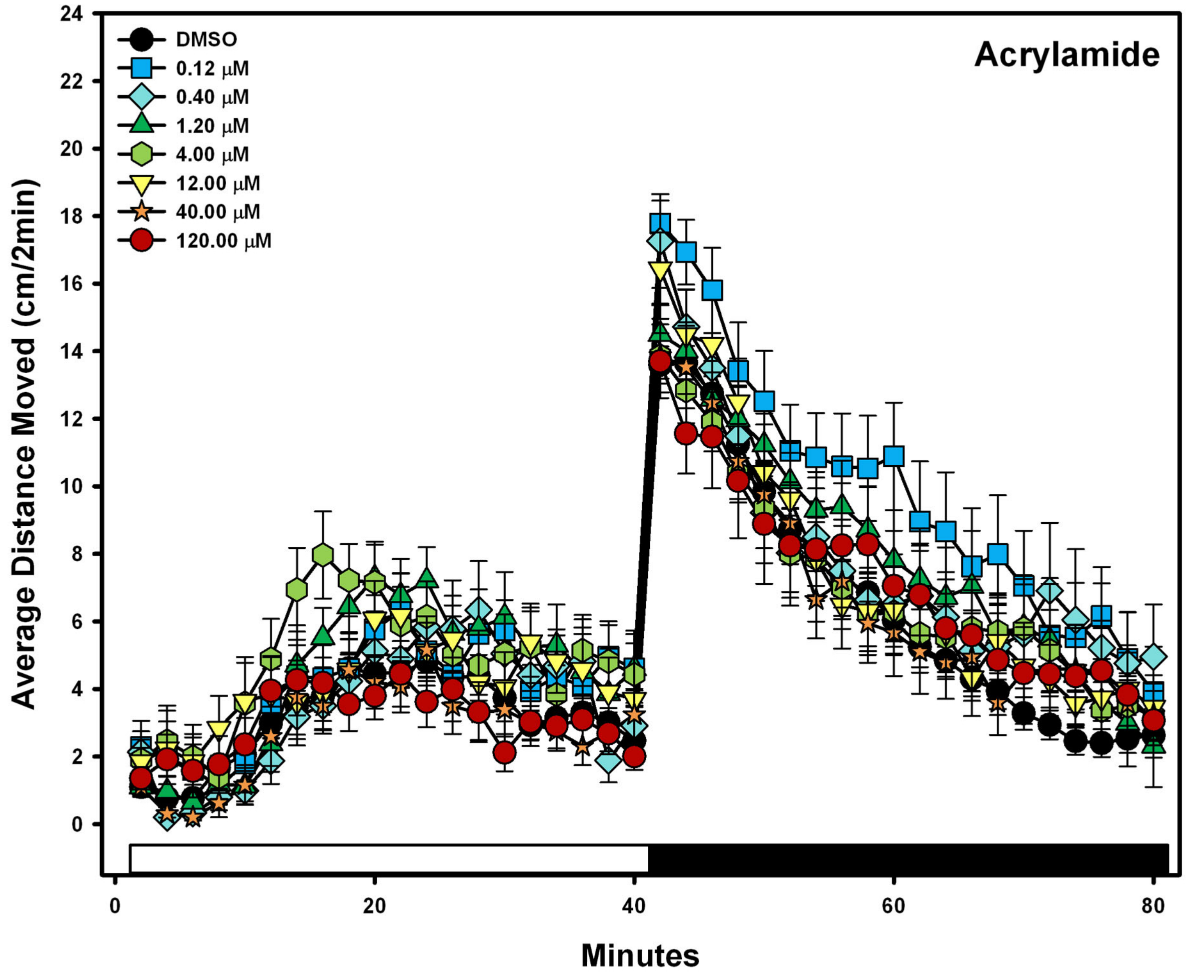

Acrylamide
DMSO
0.12 µM
0.40 µM
1.20 µM
4.00 µM
12.00 µM
40.00 µM
120.00 µM
Average Distance Moved (cm/2min)
Minutes

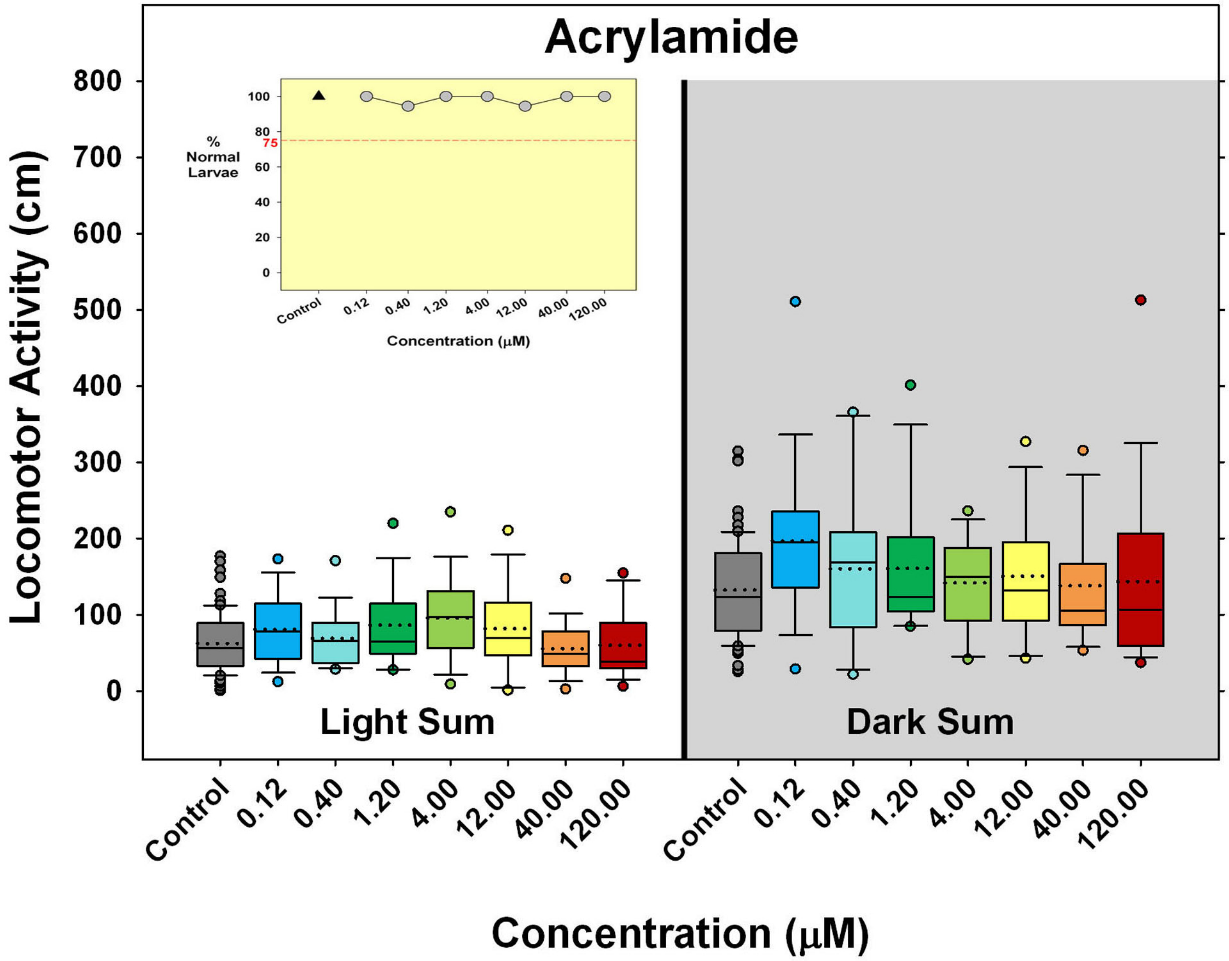

Acrylamide
% Normal Larvae
75
Control
0.12
0.40
1.20
4.00
12.00
40.00
120.00
Concentration (µM)
Locomotor Activity (cm)
Light Sum
Dark Sum
Concentration (µM)

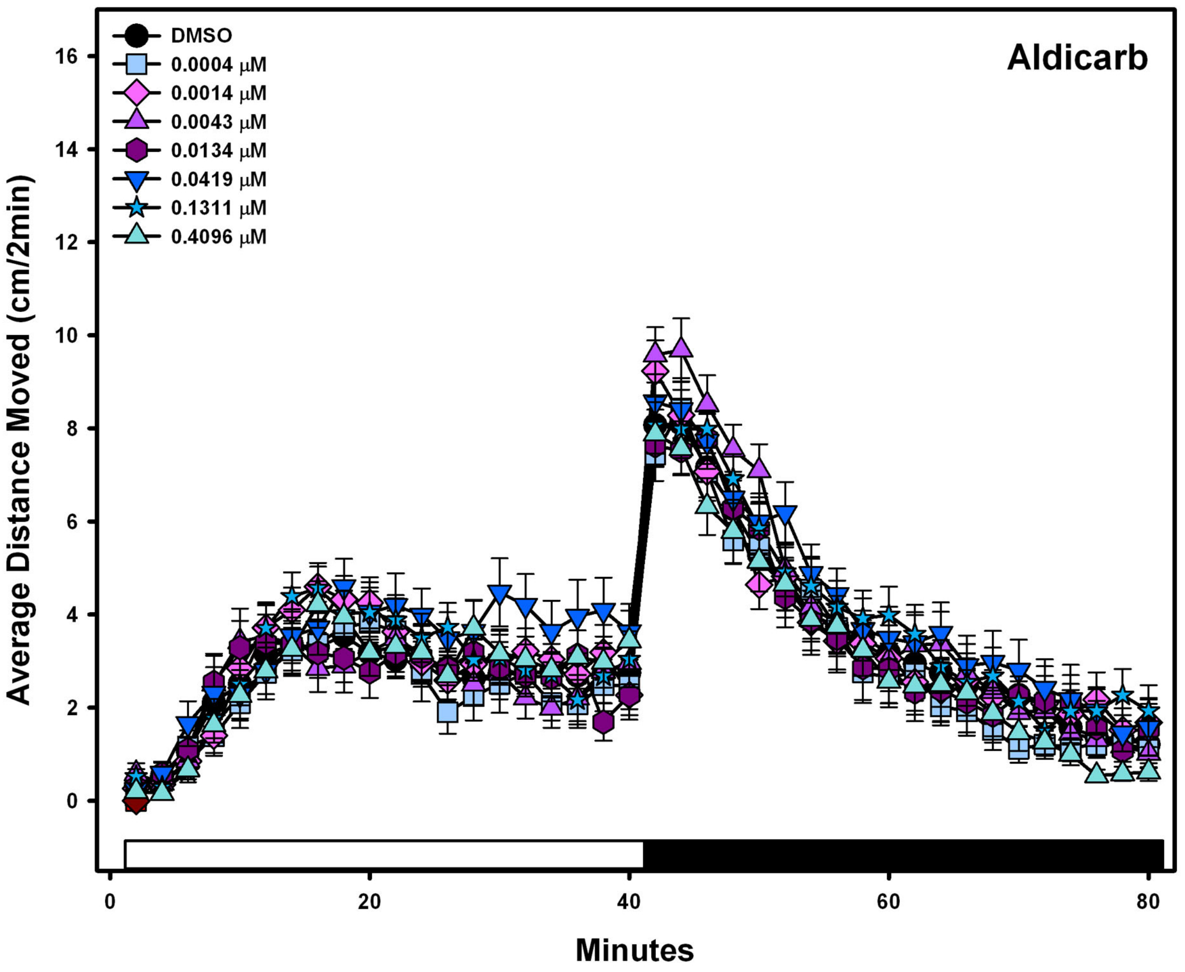
Aldicarb
DMSO
0.0004 μM
0.0014 μM
0.0043 μM
0.0134 μM
0.0419 μM
0.1311 μM
0.4096 μM
Average Distance Moved (cm/2min)
Minutes

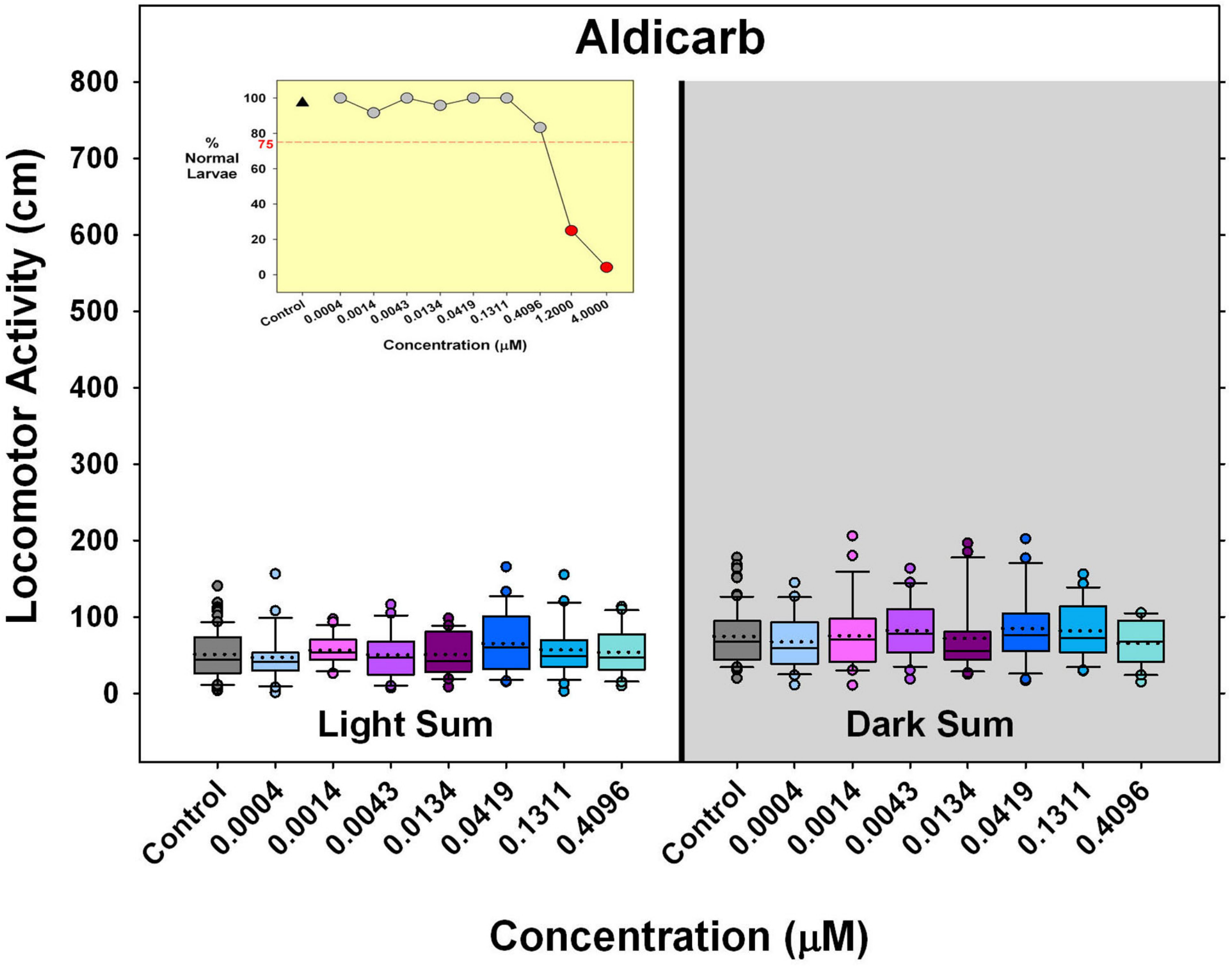
Aldicarb
Locomotor Activity (cm)
% Normal Larvae
75
Concentration (μM)
Light Sum
Dark Sum
Control
0.0004
0.0014
0.0043
0.0134
0.0419
0.1311
0.4096
1.2000
4.0000
Concentration (μM)

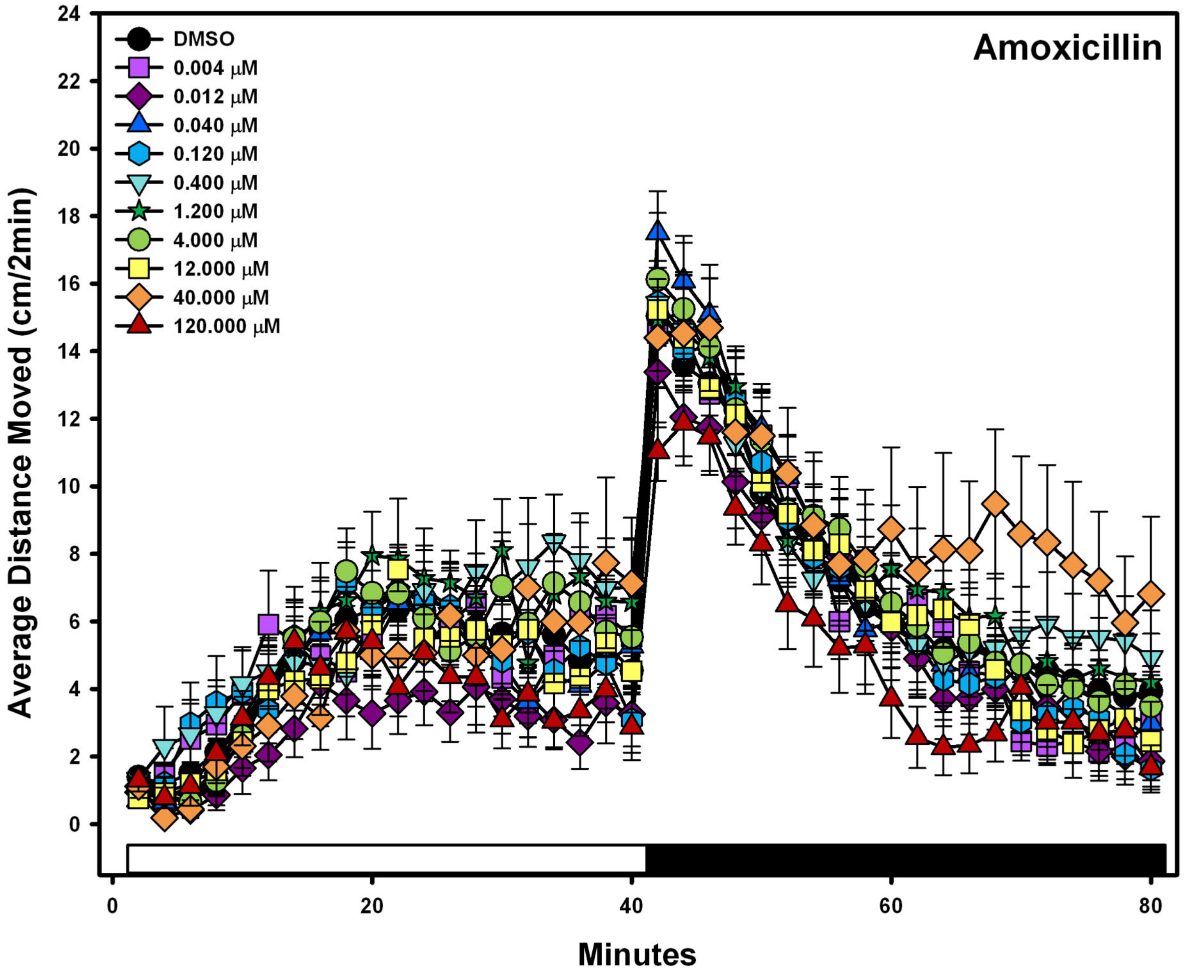
Amoxicillin
DMSO
0.004 µM
0.012 µM
0.040 µM
0.120 µM
0.400 µM
1.200 µM
4.000 µM
12.000 µM
40.000 µM
120.000 µM
Average Distance Moved (cm/2min)
Minutes

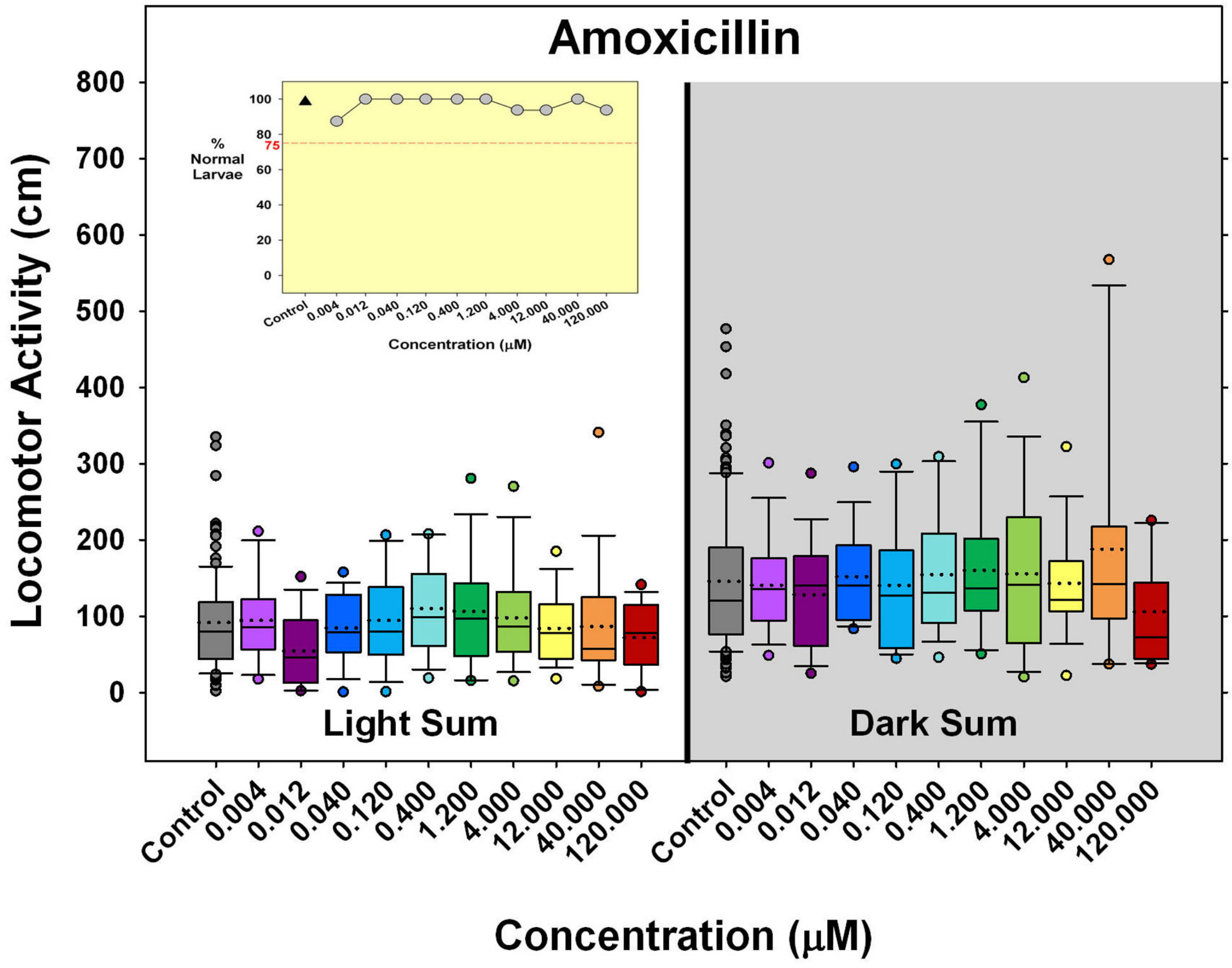
Amoxicillin
Locomotor Activity (cm)
% Normal Larvae
Concentration (µM)
Light Sum
Dark Sum
Control
0.004
0.012
0.040
0.120
0.400
1.200
4.000
12.000
40.000
120.000
Concentration (µM)

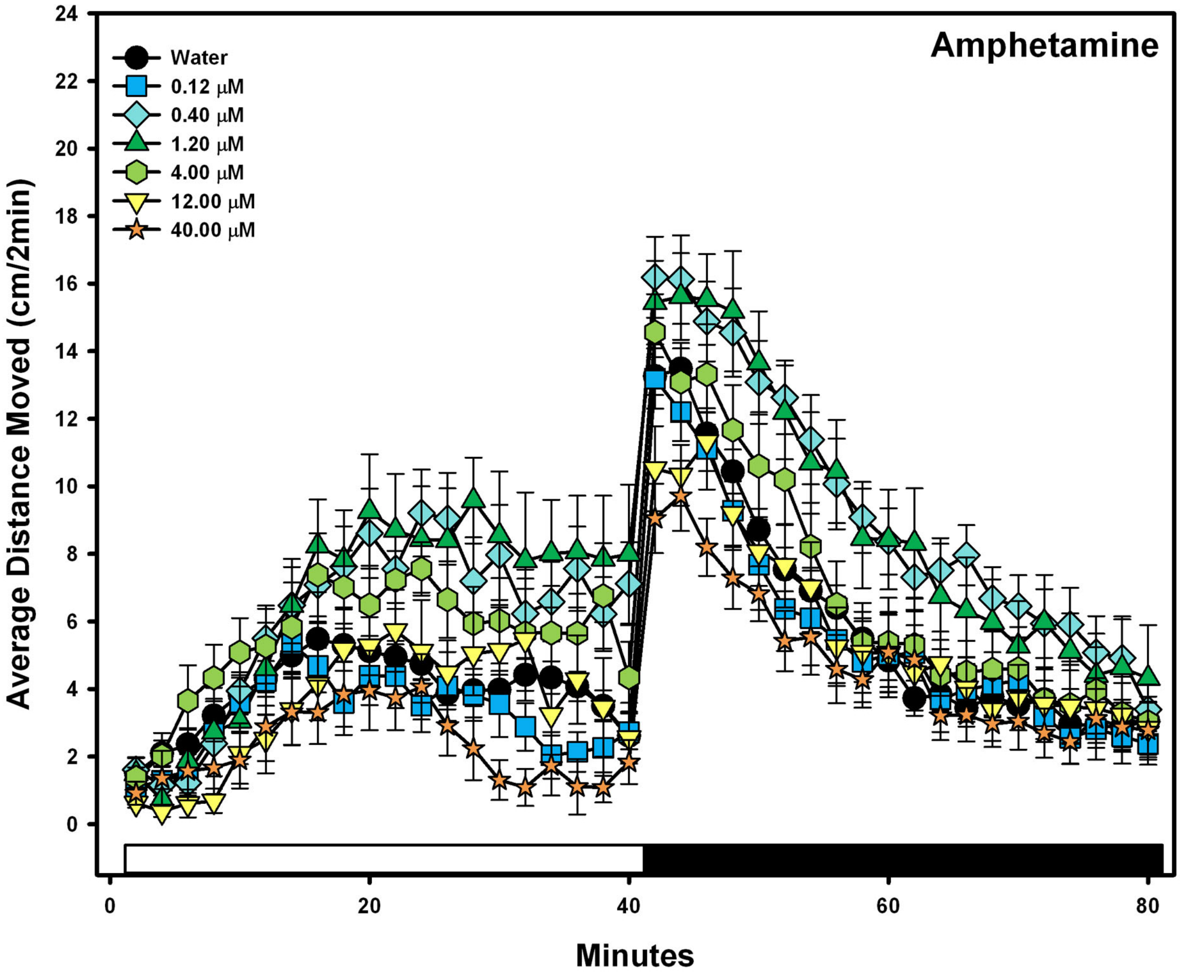
Amphetamine
Water
0.12 µM
0.40 µM
1.20 µM
4.00 µM
12.00 µM
40.00 µM
Average Distance Moved (cm/2min)
Minutes

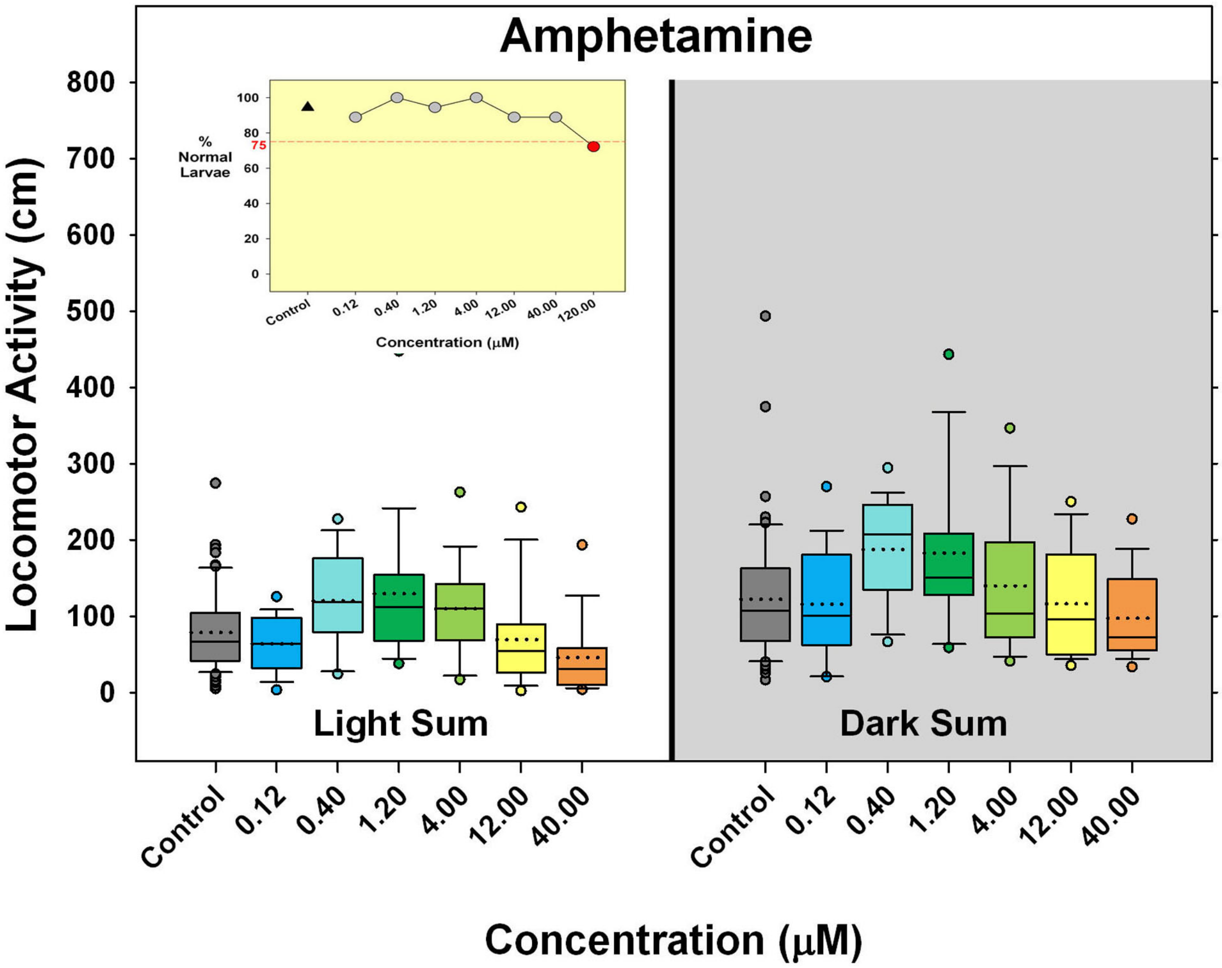
Amphetamine
Locomotor Activity (cm)
% Normal Larvae
75
Concentration (µM)
Control
0.12
0.40
1.20
4.00
12.00
40.00
120.00
Light Sum
Dark Sum
Concentration (µM)

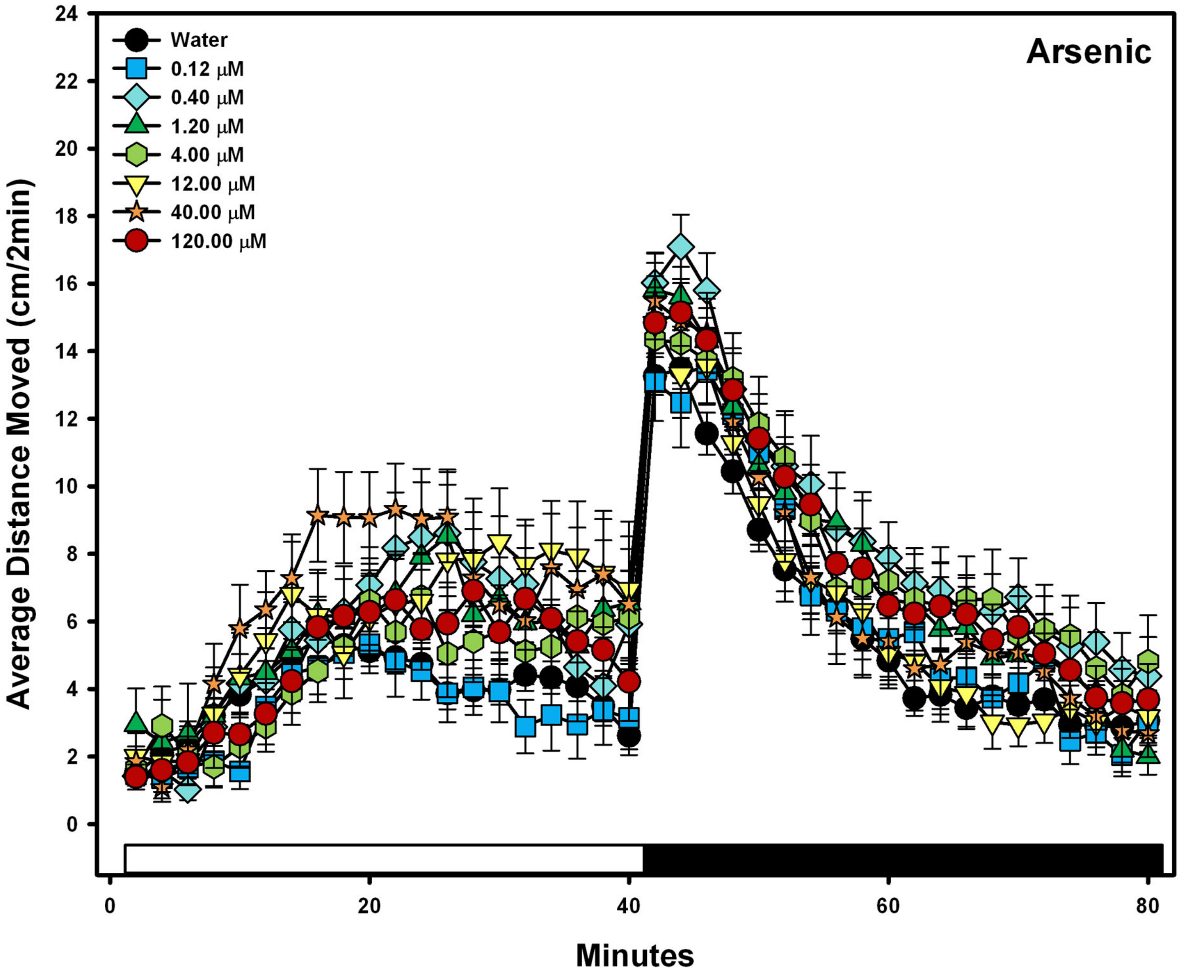
Arsenic
Water
0.12 μM
0.40 μM
1.20 μM
4.00 μM
12.00 μM
40.00 μM
120.00 μM
Average Distance Moved (cm/2min)
Minutes
0
20
40
60
80

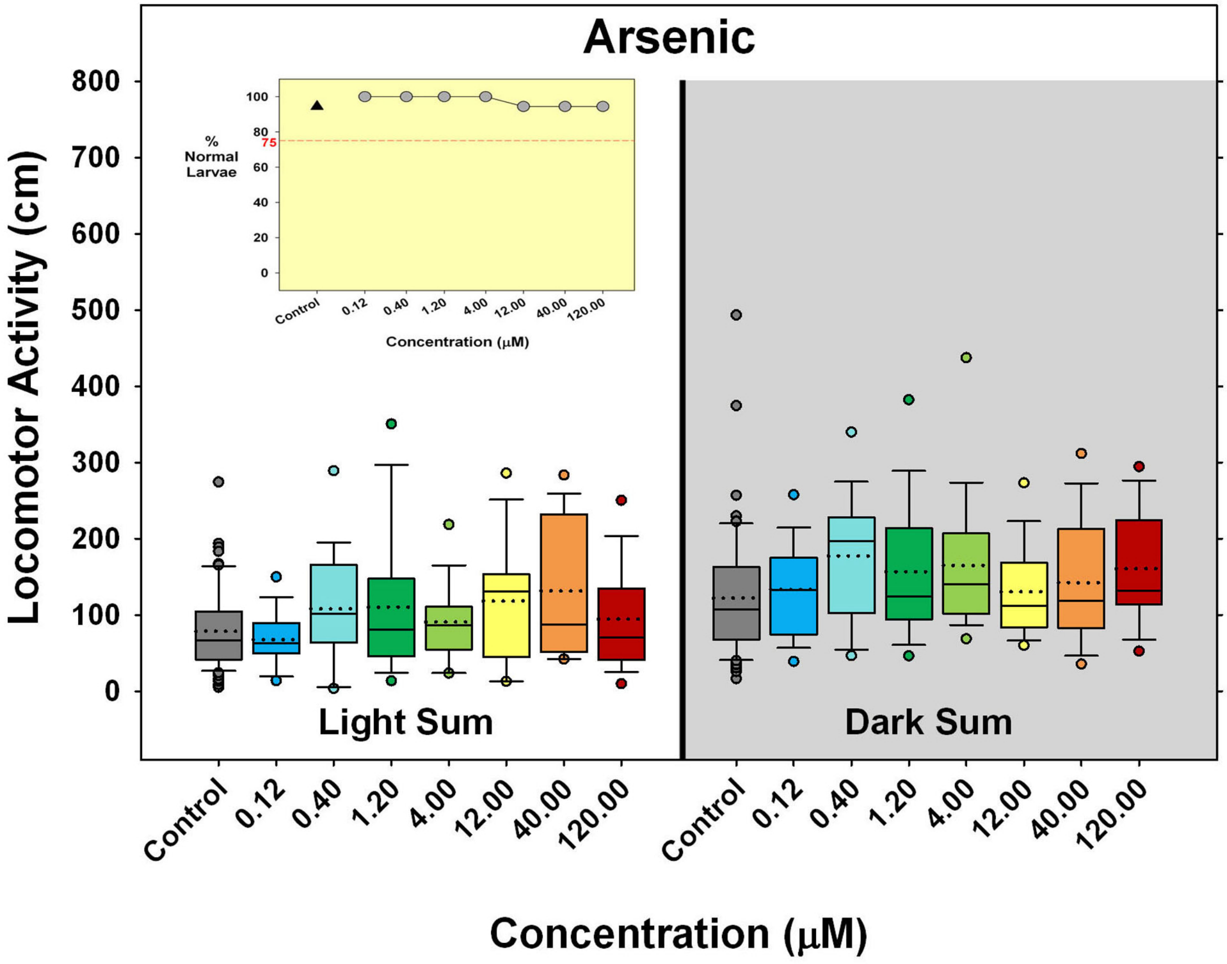
Arsenic
% Normal Larvae
75
Concentration (μM)
Locomotor Activity (cm)
Light Sum
Dark Sum
Control
0.12
0.40
1.20
4.00
12.00
40.00
120.00
Concentration (μM)

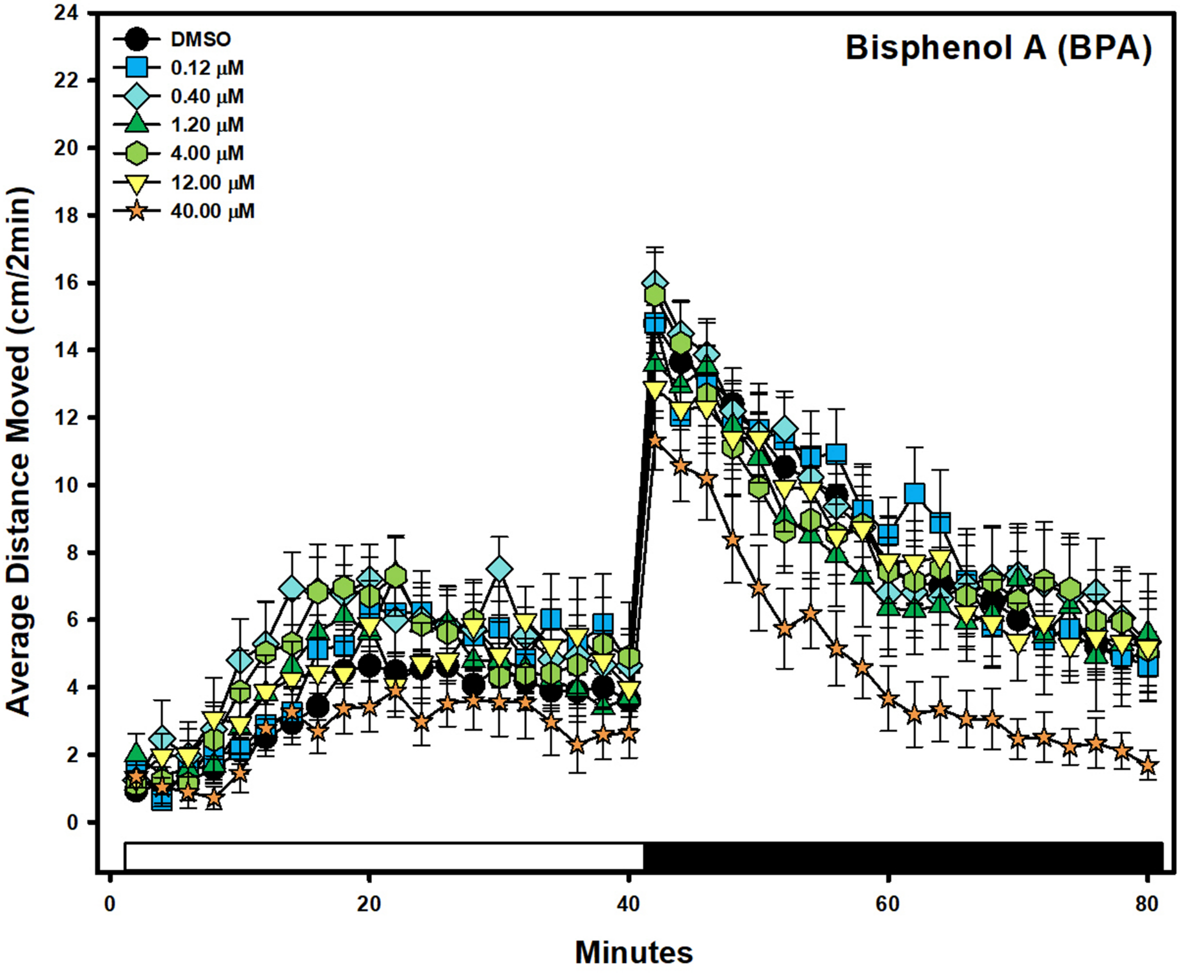

Bisphenol A (BPA)
DMSO
0.12 µM
0.40 µM
1.20 µM
4.00 µM
12.00 µM
40.00 µM
Average Distance Moved (cm/2min)
Minutes

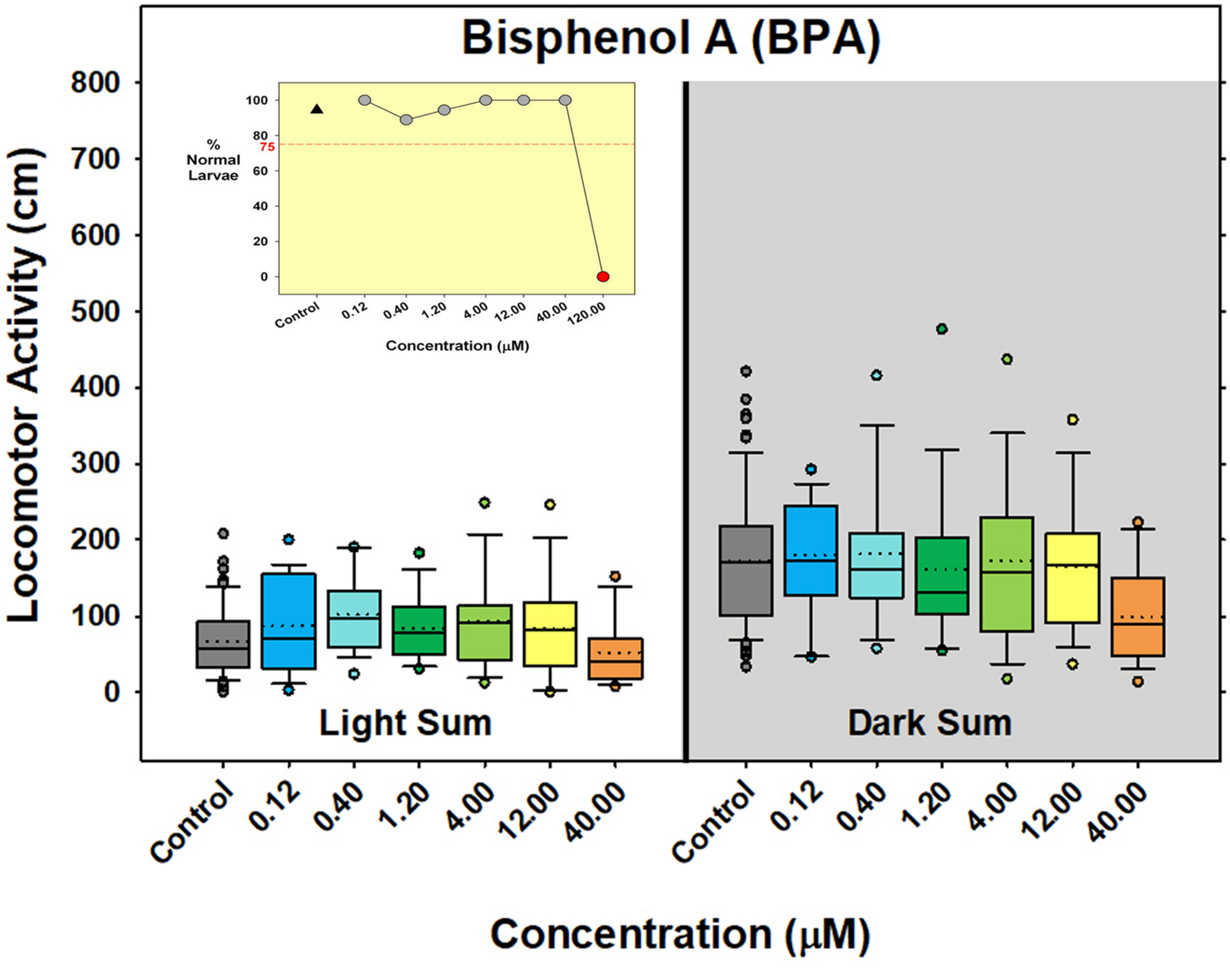

Bisphenol A (BPA)
Locomotor Activity (cm)
% Normal Larvae
75
Concentration (µM)
Control
0.12
0.40
1.20
4.00
12.00
40.00
120.00
Light Sum
Dark Sum
Concentration (µM)

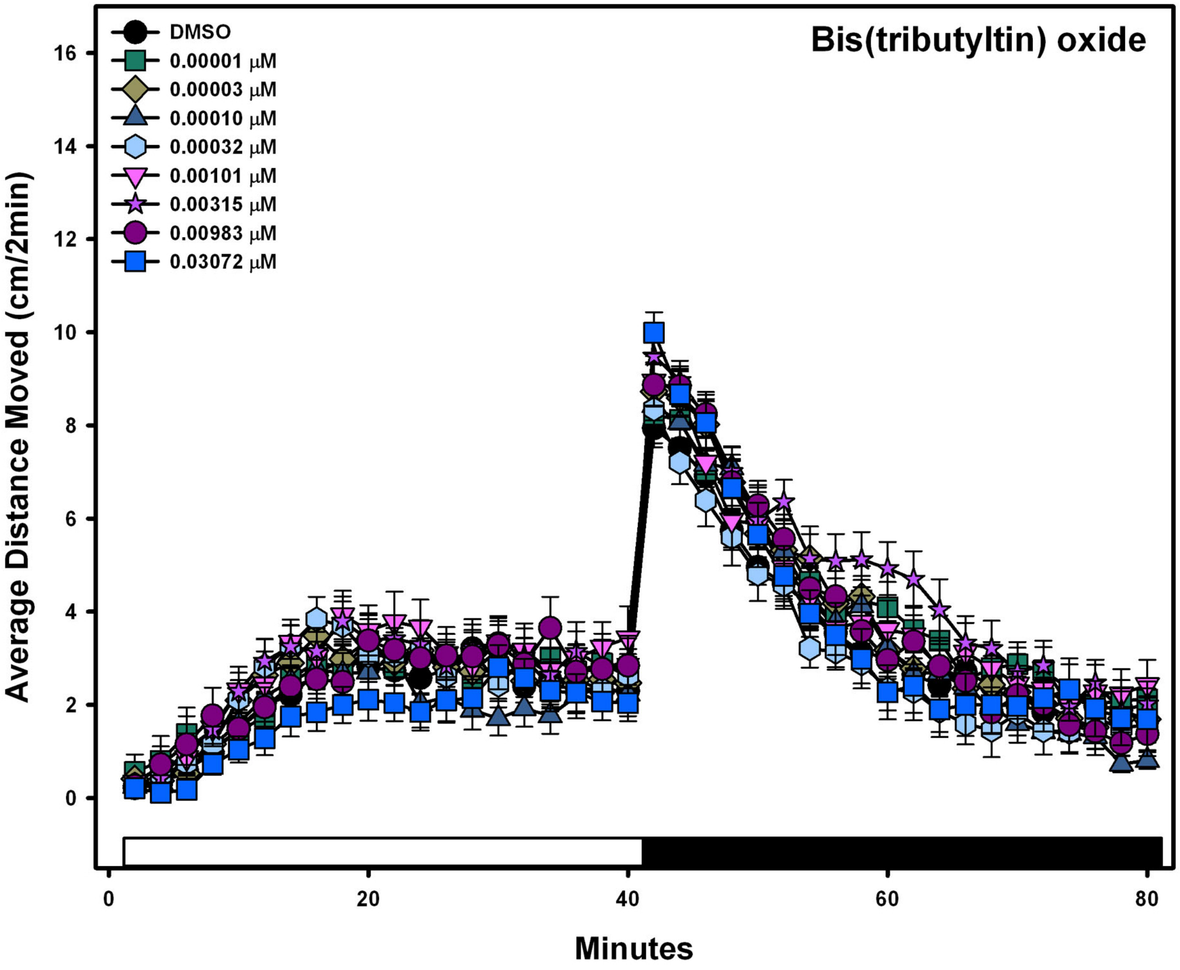
Bis(tributyltin) oxide
DMSO
0.00001 µM
0.00003 µM
0.00010 µM
0.00032 µM
0.00101 µM
0.00315 µM
0.00983 µM
0.03072 µM
Average Distance Moved (cm/2min)
0
2
4
6
8
10
12
14
16
0
20
40
60
80
Minutes

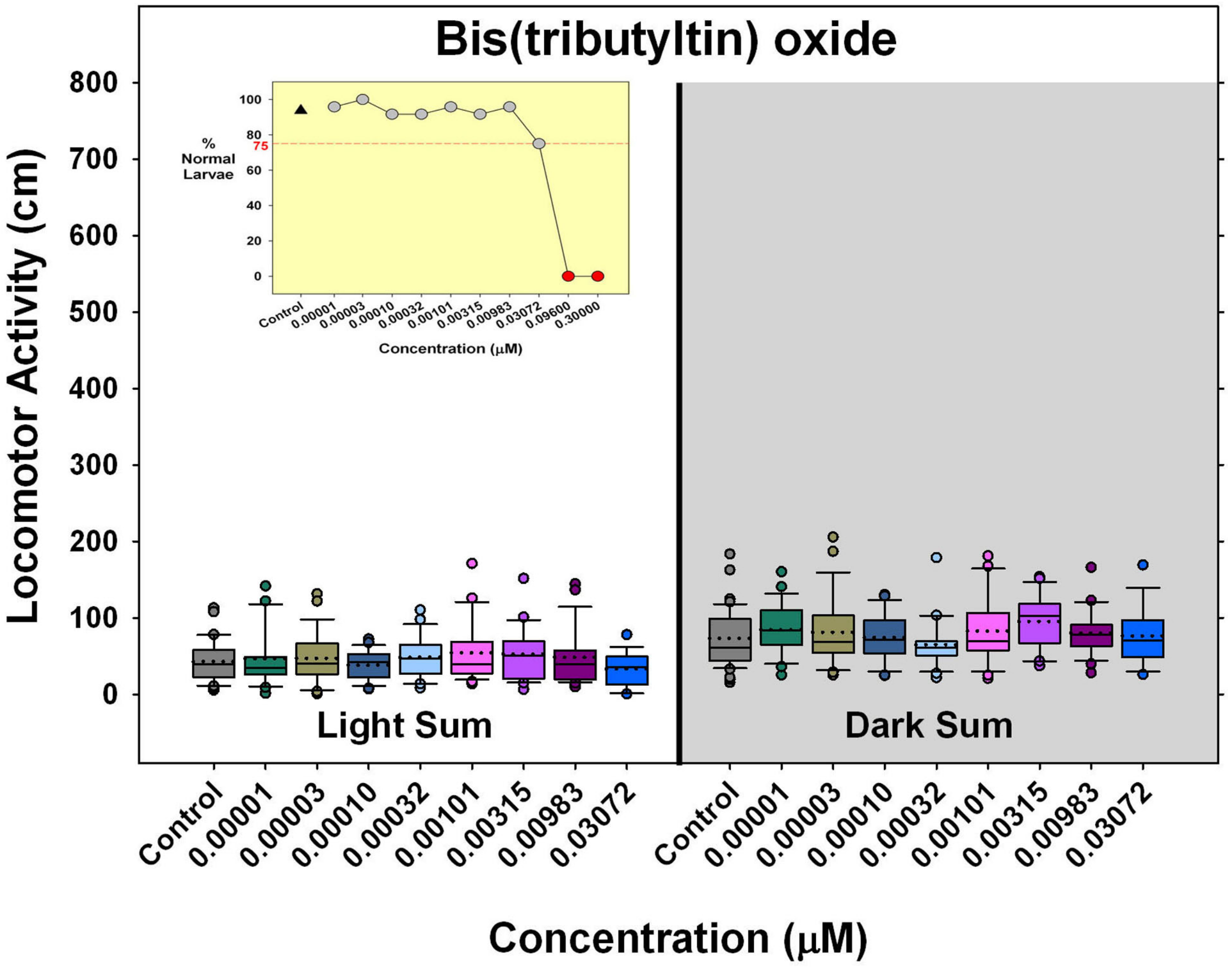
Bis(tributyltin) oxide
Locomotor Activity (cm)
0
100
200
300
400
500
600
700
800
% Normal Larvae
75
Control 0.00001 0.00003 0.00010 0.00032 0.00101 0.00315 0.00983 0.03072 0.09600 0.30000
Concentration (µM)
Light Sum
Dark Sum
Control 0.00001 0.00003 0.00010 0.00032 0.00101 0.00315 0.00983 0.03072
Control 0.00001 0.00003 0.00010 0.00032 0.00101 0.00315 0.00983 0.03072
Concentration (µM)

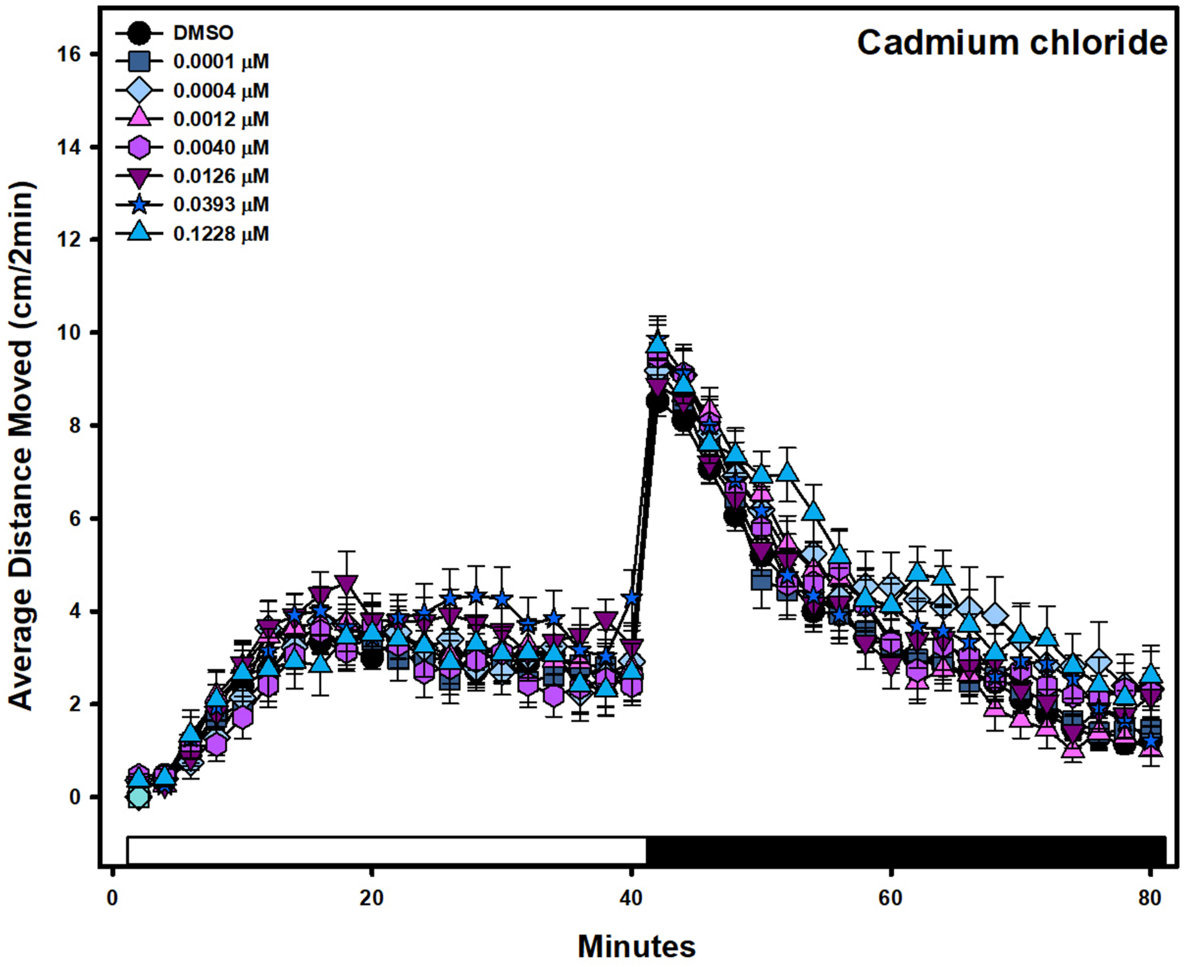

Cadmium chloride
DMSO
0.0001 µM
0.0004 µM
0.0012 µM
0.0040 µM
0.0126 µM
0.0393 µM
0.1228 µM
Average Distance Moved (cm/2min)
Minutes

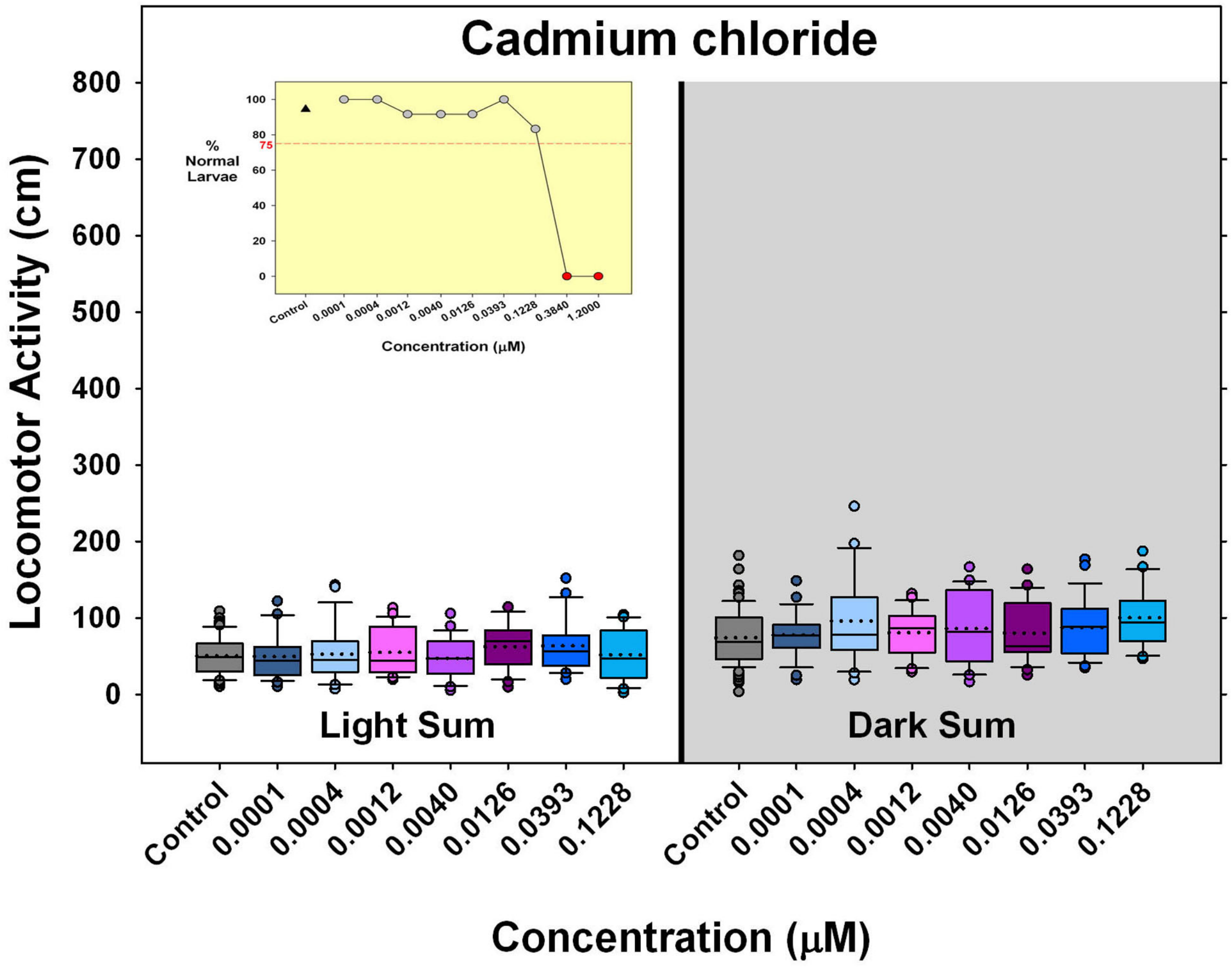

Cadmium chloride
Locomotor Activity (cm)
% Normal Larvae
Concentration (µM)
Light Sum
Dark Sum
Control
0.0001
0.0004
0.0012
0.0040
0.0126
0.0393
0.1228
Concentration (µM)

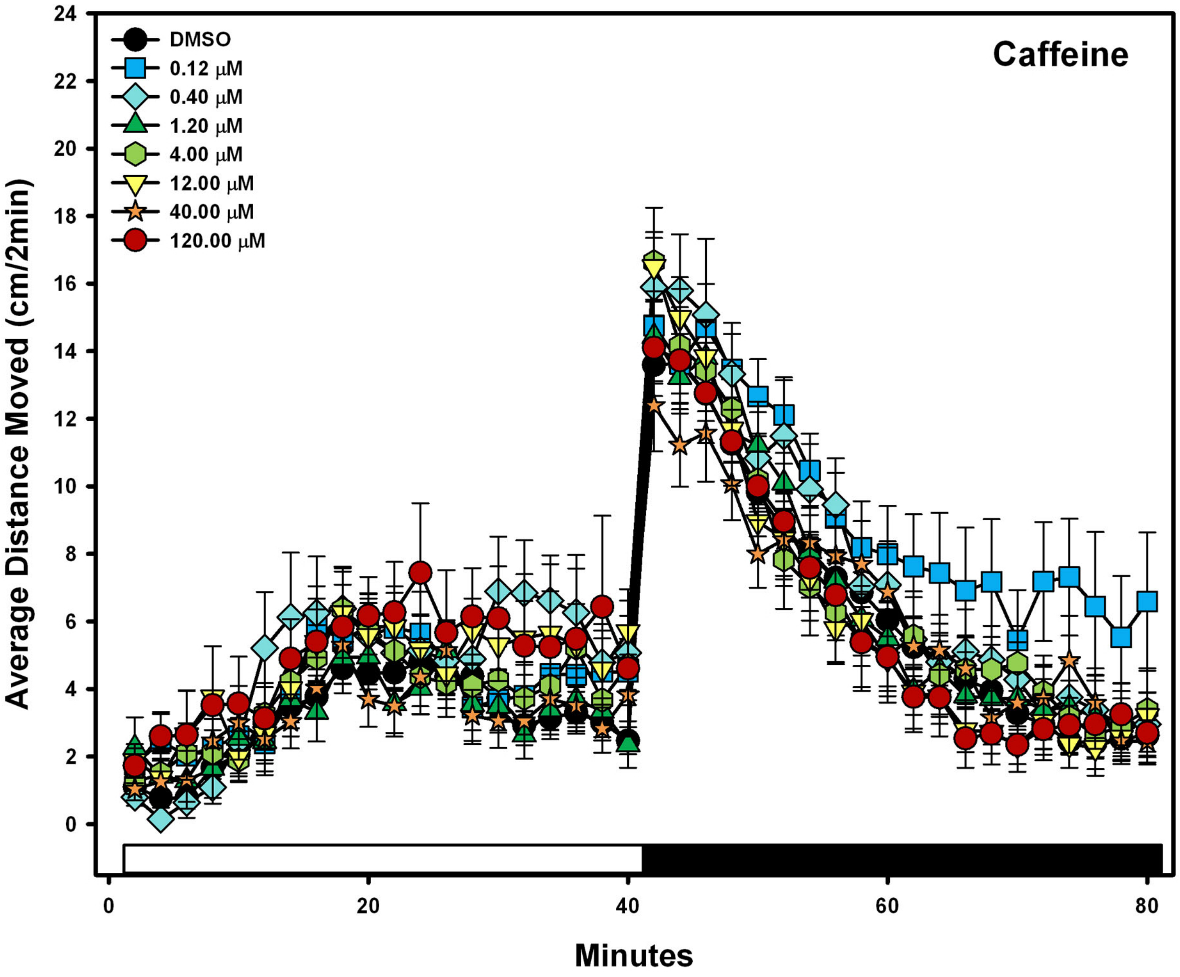
Caffeine
DMSO
0.12 µM
0.40 µM
1.20 µM
4.00 µM
12.00 µM
40.00 µM
120.00 µM
Average Distance Moved (cm/2min)
0
2
4
6
8
10
12
14
16
18
20
22
24
0
20
40
60
80
Minutes

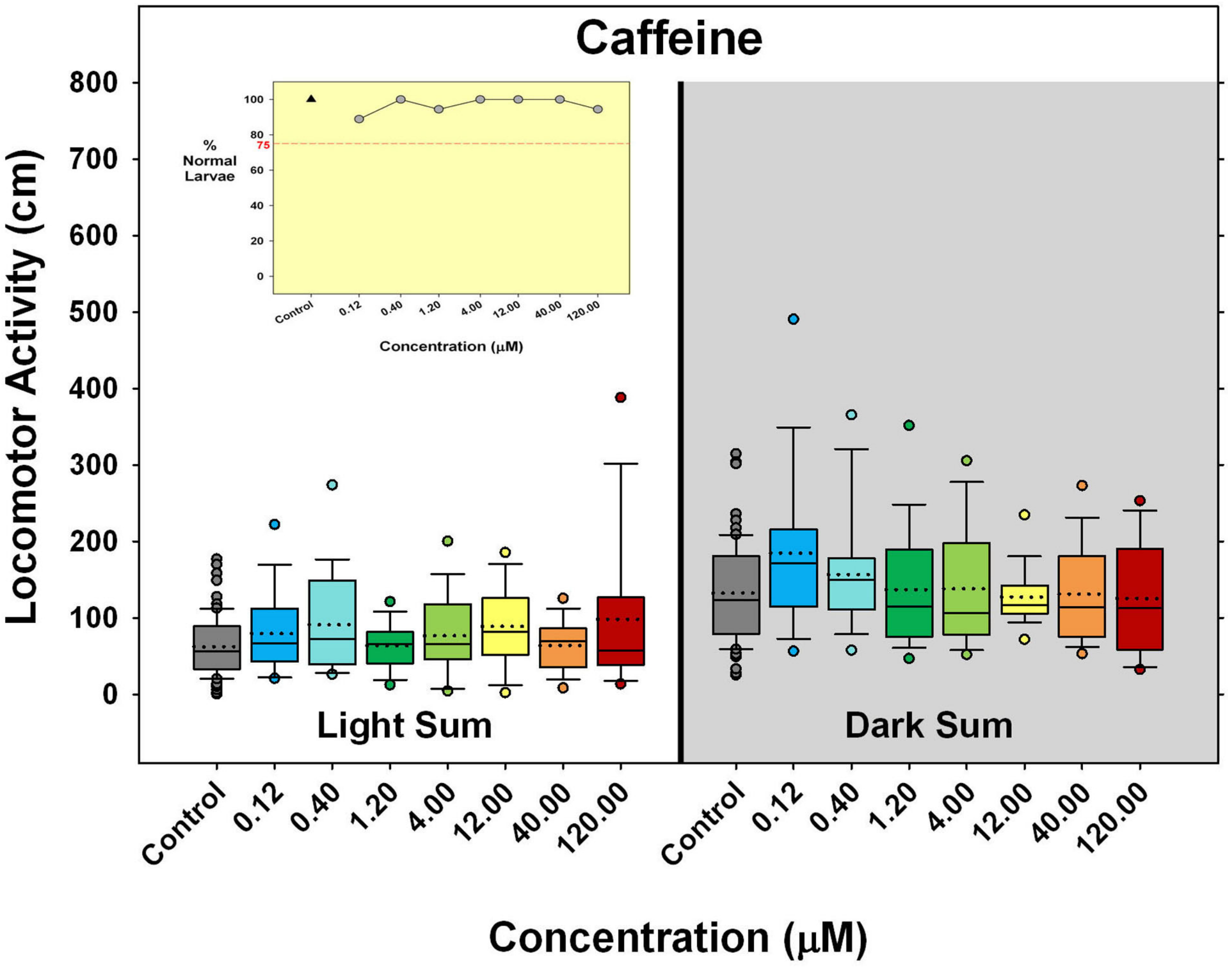
Caffeine
Locomotor Activity (cm)
0
100
200
300
400
500
600
700
800
% Normal Larvae
0
20
40
60
75
80
100
Control
0.12
0.40
1.20
4.00
12.00
40.00
120.00
Concentration (µM)
Light Sum
Dark Sum
Control
0.12
0.40
1.20
4.00
12.00
40.00
120.00
Control
0.12
0.40
1.20
4.00
12.00
40.00
120.00
Concentration (µM)

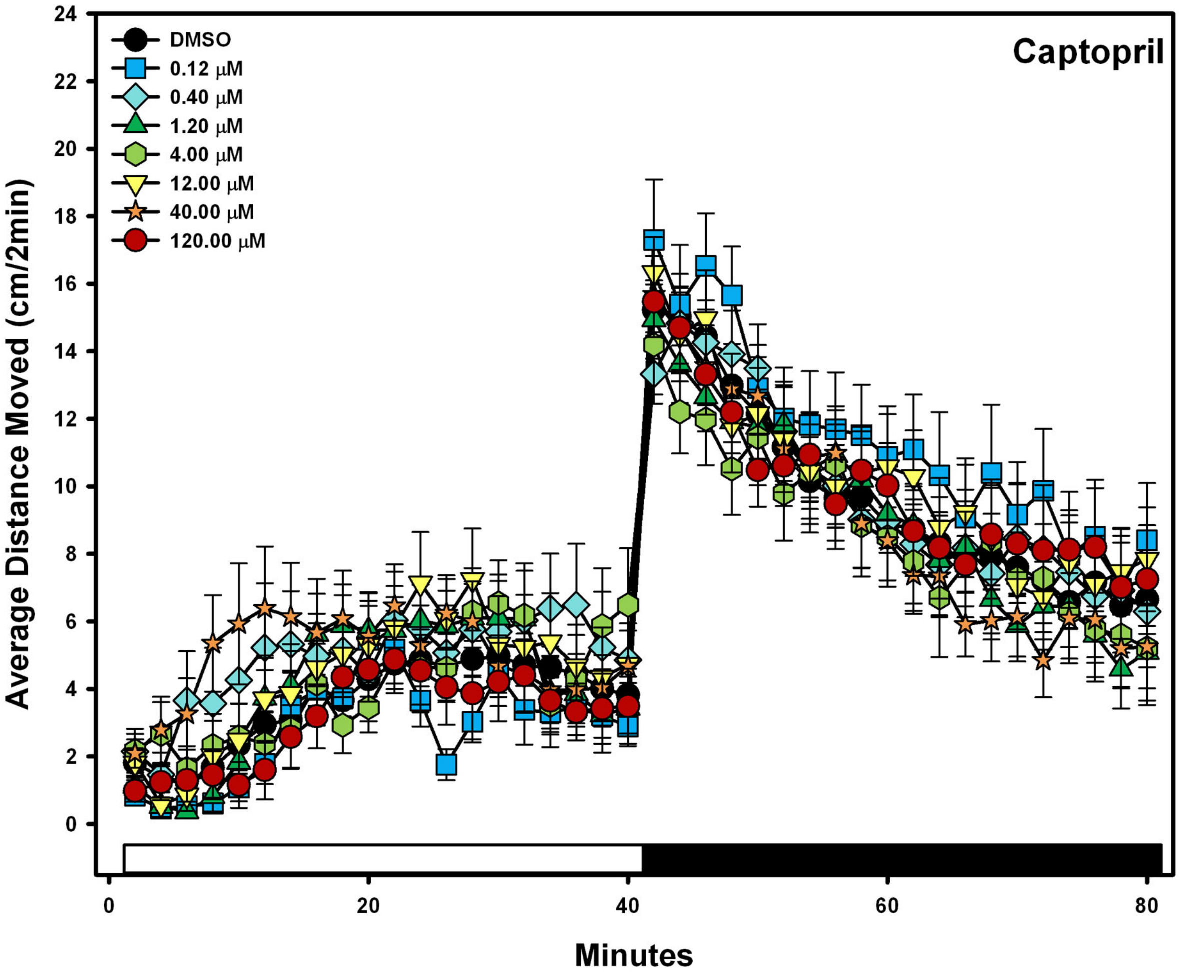
Captopril
DMSO
0.12 µM
0.40 µM
1.20 µM
4.00 µM
12.00 µM
40.00 µM
120.00 µM
Average Distance Moved (cm/2min)
Minutes

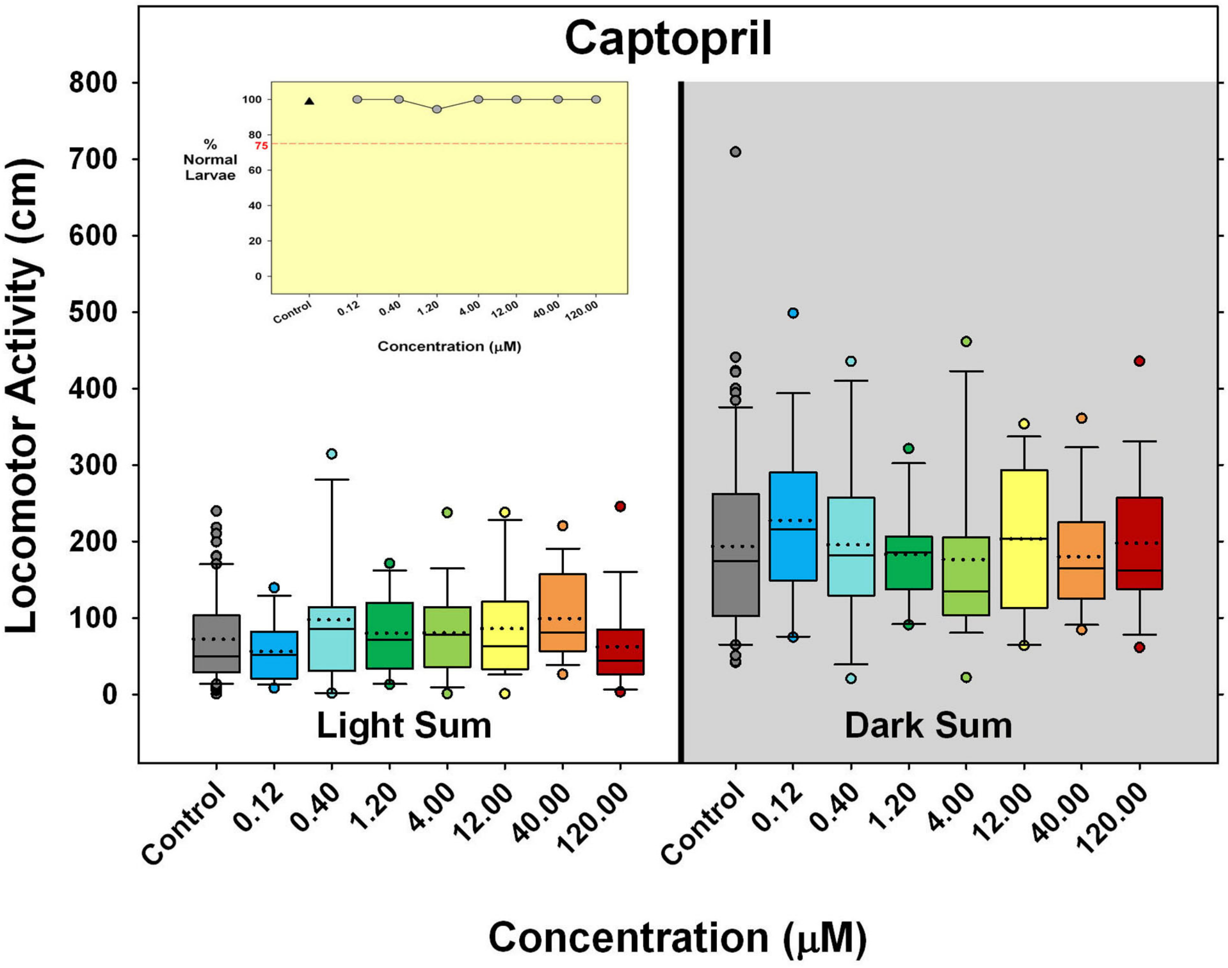
Captopril
% Normal Larvae
Concentration (µM)
Control
0.12
0.40
1.20
4.00
12.00
40.00
120.00
Locomotor Activity (cm)
Light Sum
Dark Sum
Concentration (µM)

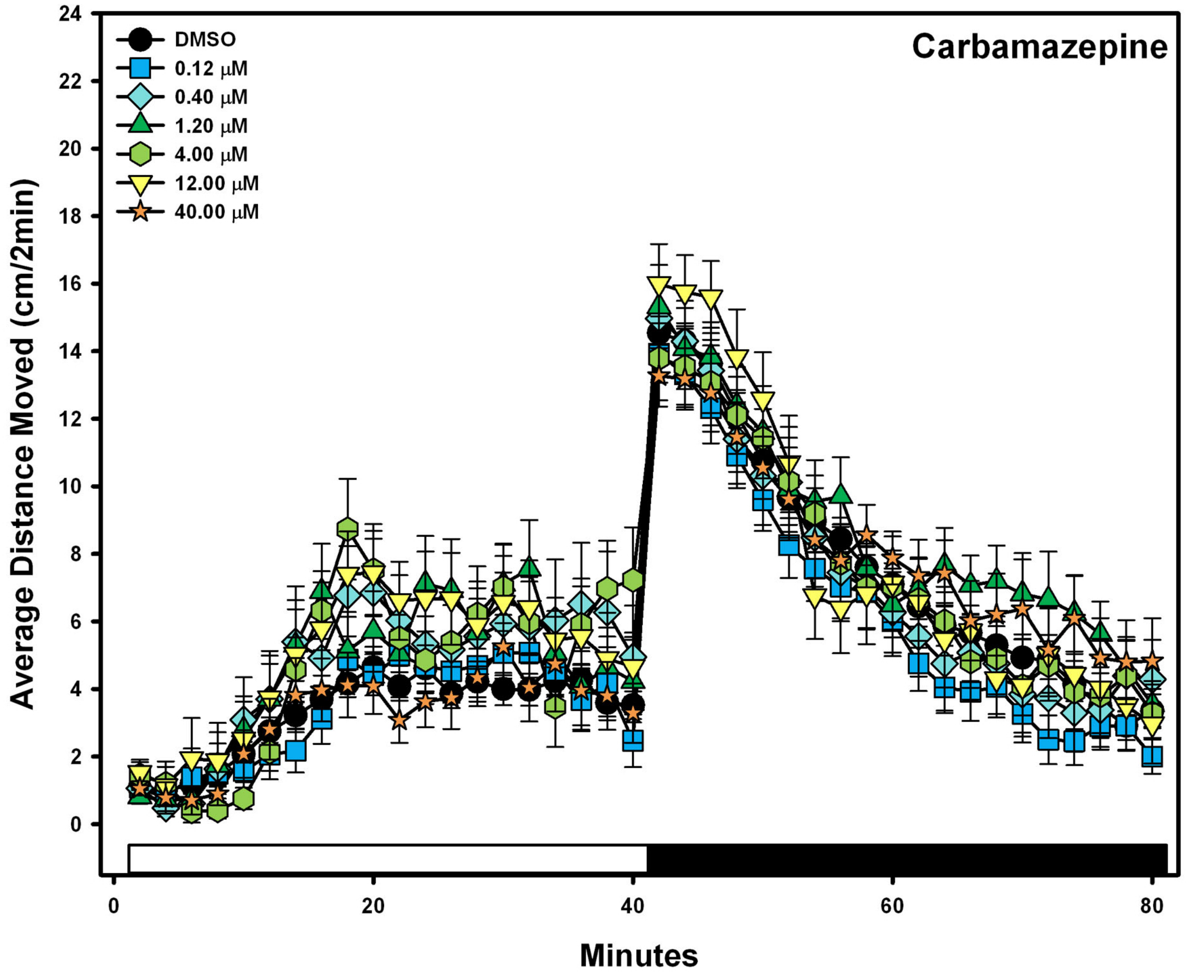
Carbamazepine
DMSO
0.12 μM
0.40 μM
1.20 μM
4.00 μM
12.00 μM
40.00 μM
Average Distance Moved (cm/2min)
Minutes

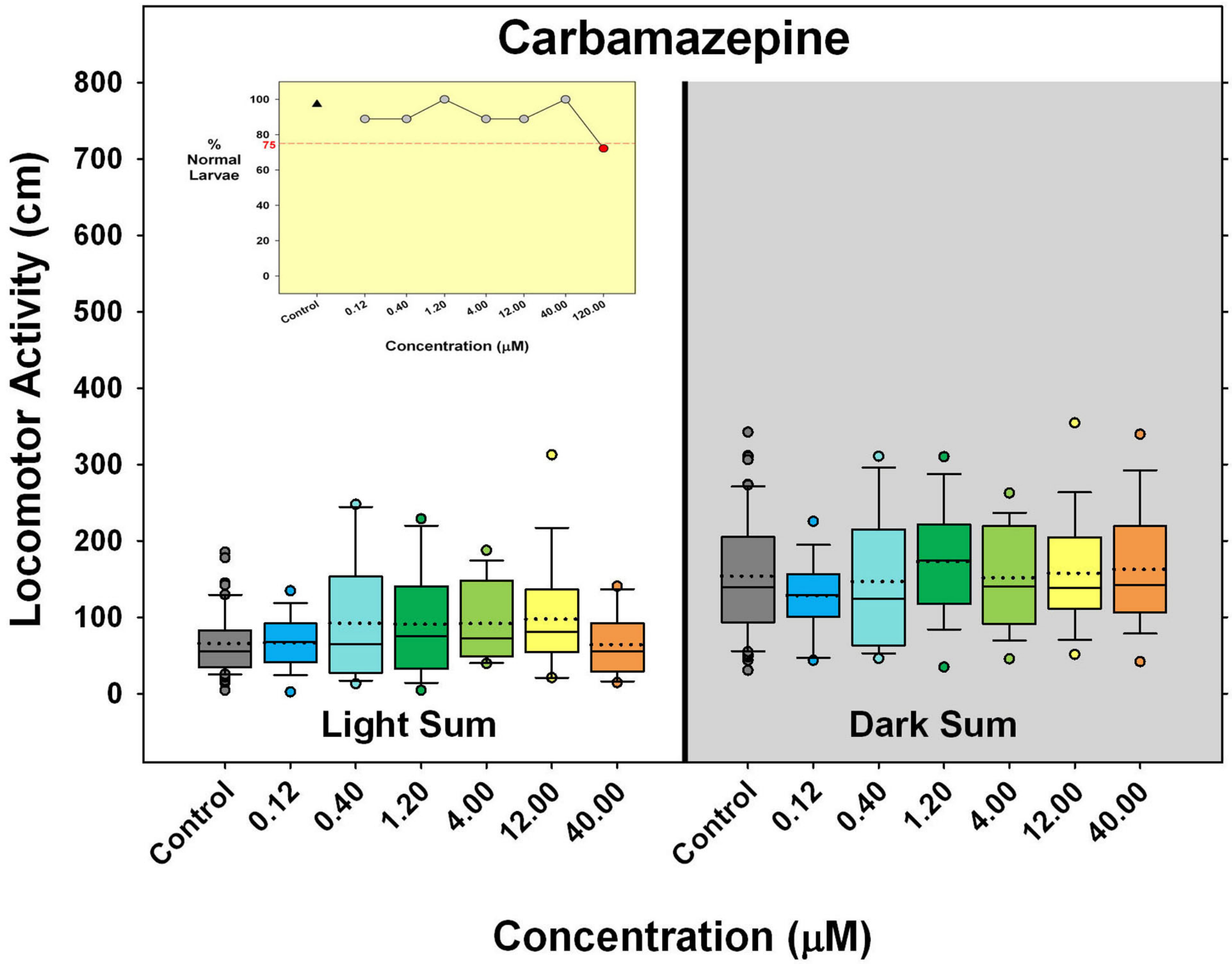
Carbamazepine
Locomotor Activity (cm)
% Normal Larvae
Concentration (μM)
Light Sum
Dark Sum
Control
0.12
0.40
1.20
4.00
12.00
40.00
120.00
Concentration (μM)

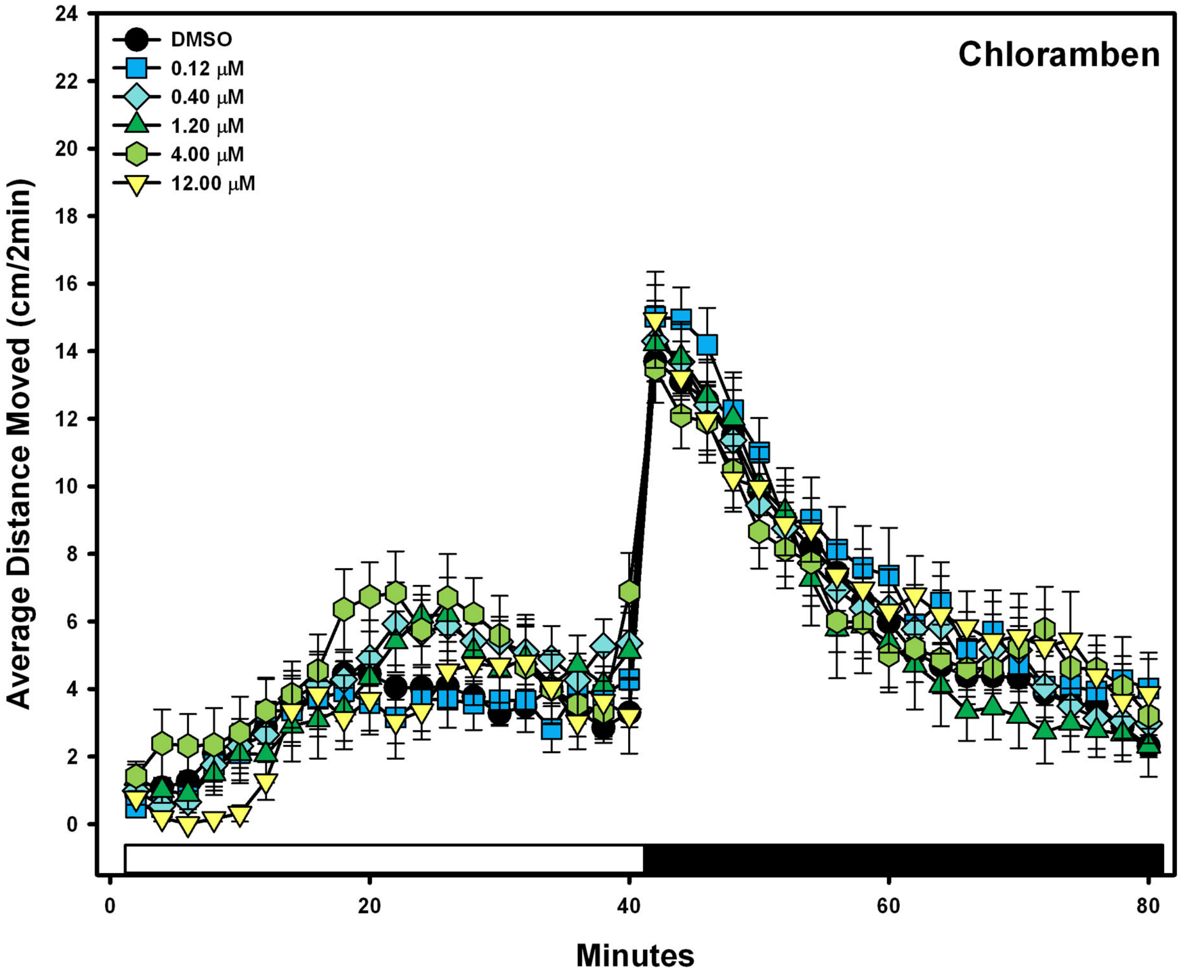

Chloramben
DMSO
0.12 µM
0.40 µM
1.20 µM
4.00 µM
12.00 µM
Average Distance Moved (cm/2min)
Minutes

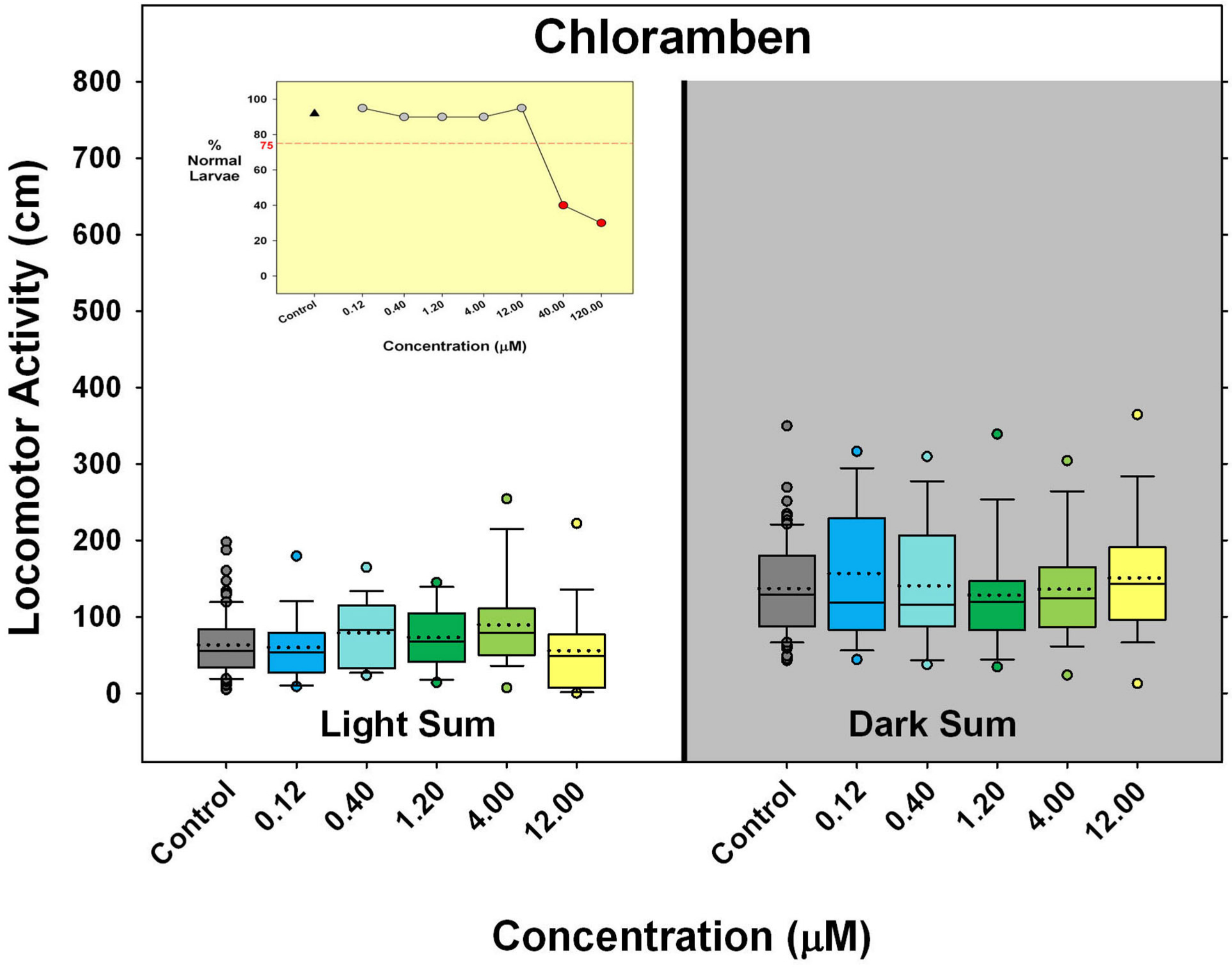

Chloramben
Locomotor Activity (cm)
Light Sum
Dark Sum
Control
0.12
0.40
1.20
4.00
12.00
% Normal Larvae
75
40.00
120.00
Concentration (µM)

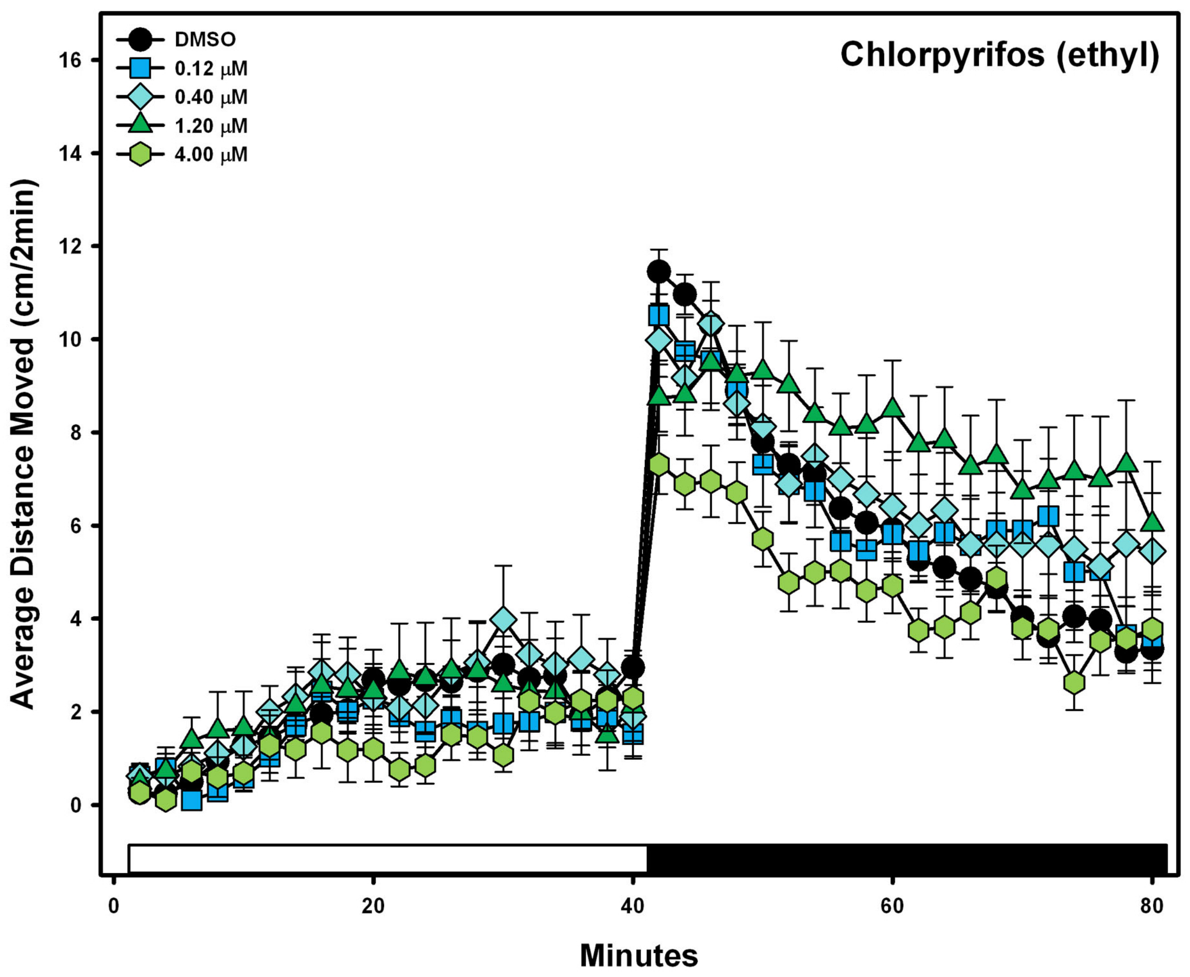

Chlorpyrifos (ethyl)
DMSO
0.12 µM
0.40 µM
1.20 µM
4.00 µM
Average Distance Moved (cm/2min)
Minutes

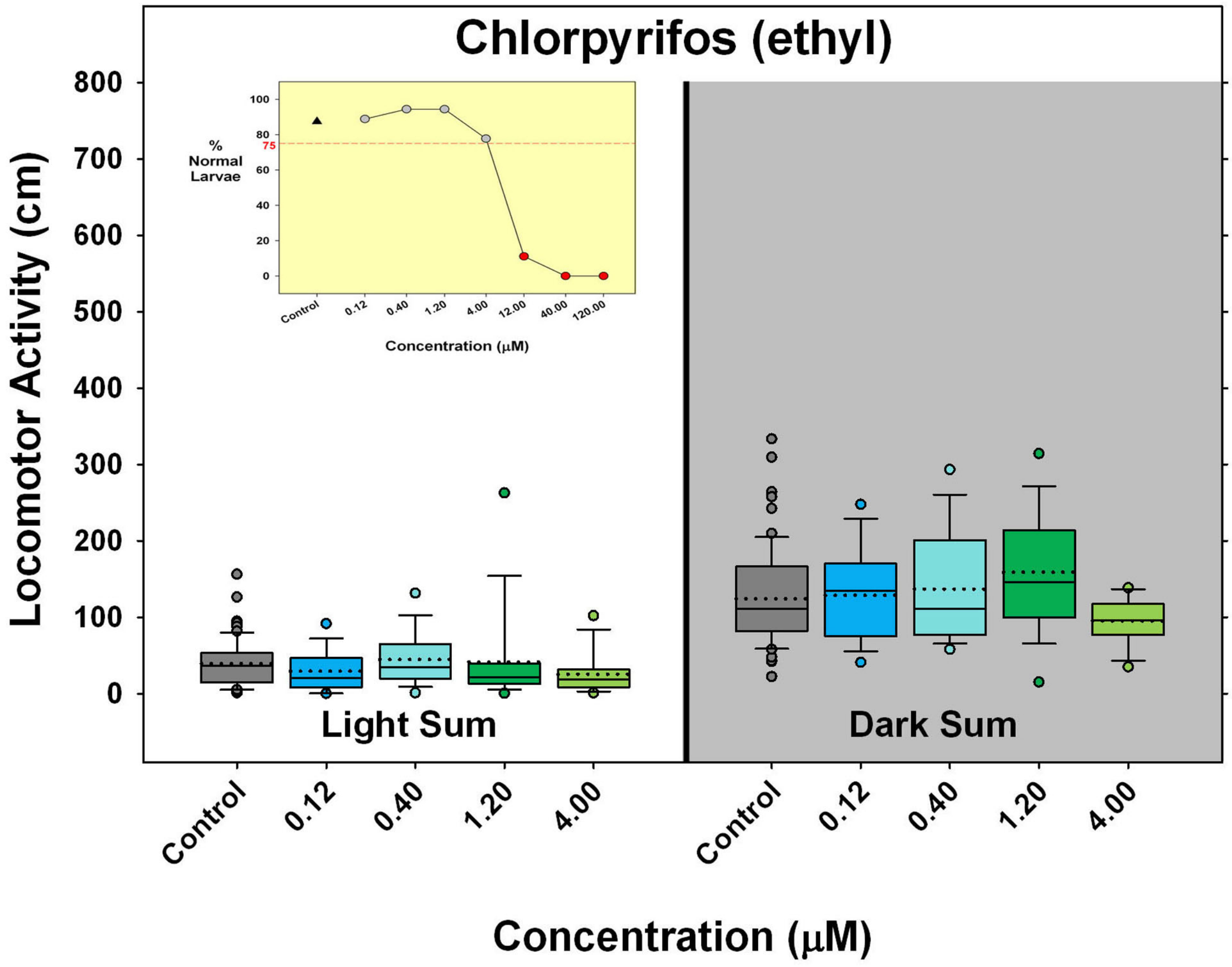

Chlorpyrifos (ethyl)
Locomotor Activity (cm)
% Normal Larvae
Concentration (µM)
Light Sum
Dark Sum
Control
0.12
0.40
1.20
4.00
Concentration (µM)

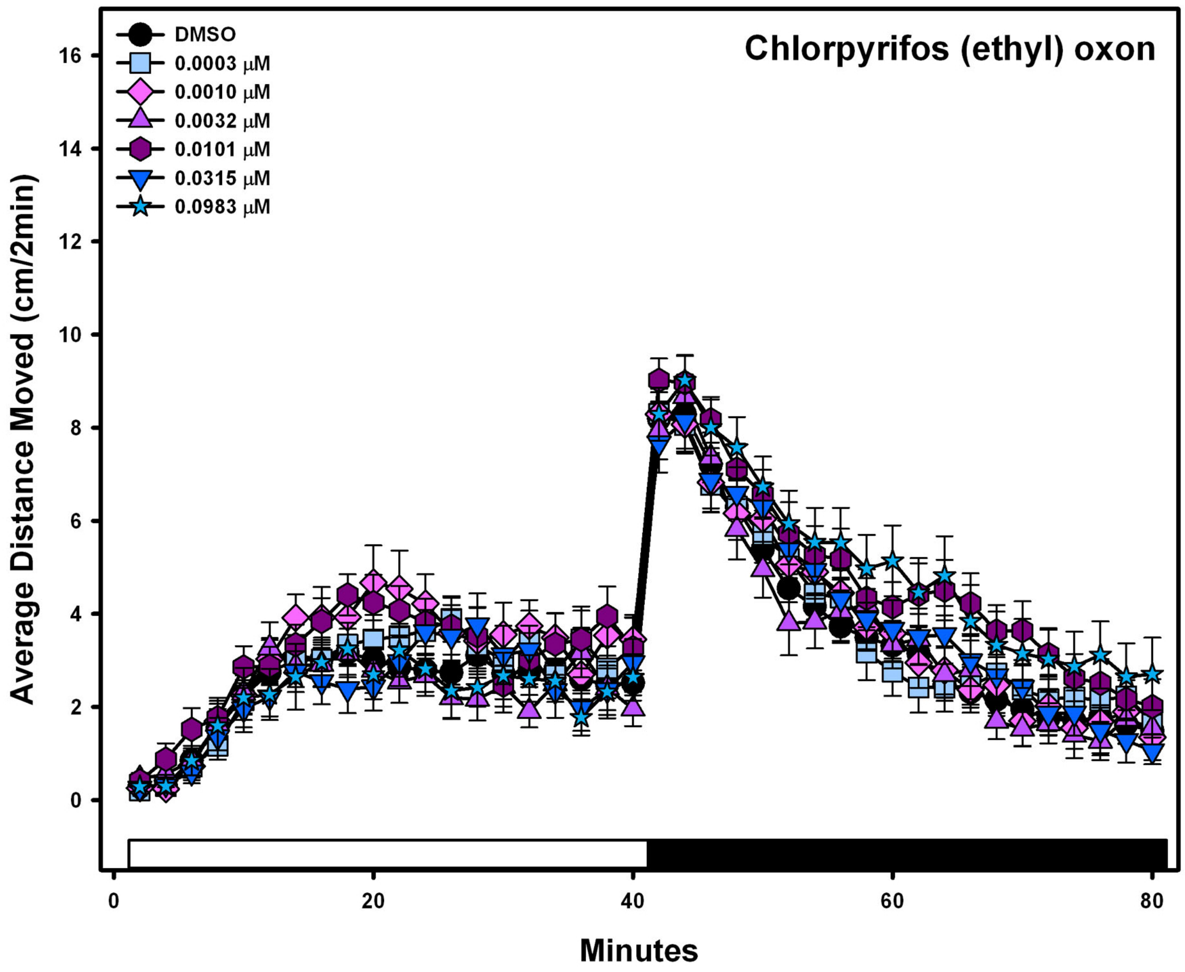

Chlorpyrifos (ethyl) oxon
DMSO
0.0003 μM
0.0010 μM
0.0032 μM
0.0101 μM
0.0315 μM
0.0983 μM
Average Distance Moved (cm/2min)
Minutes

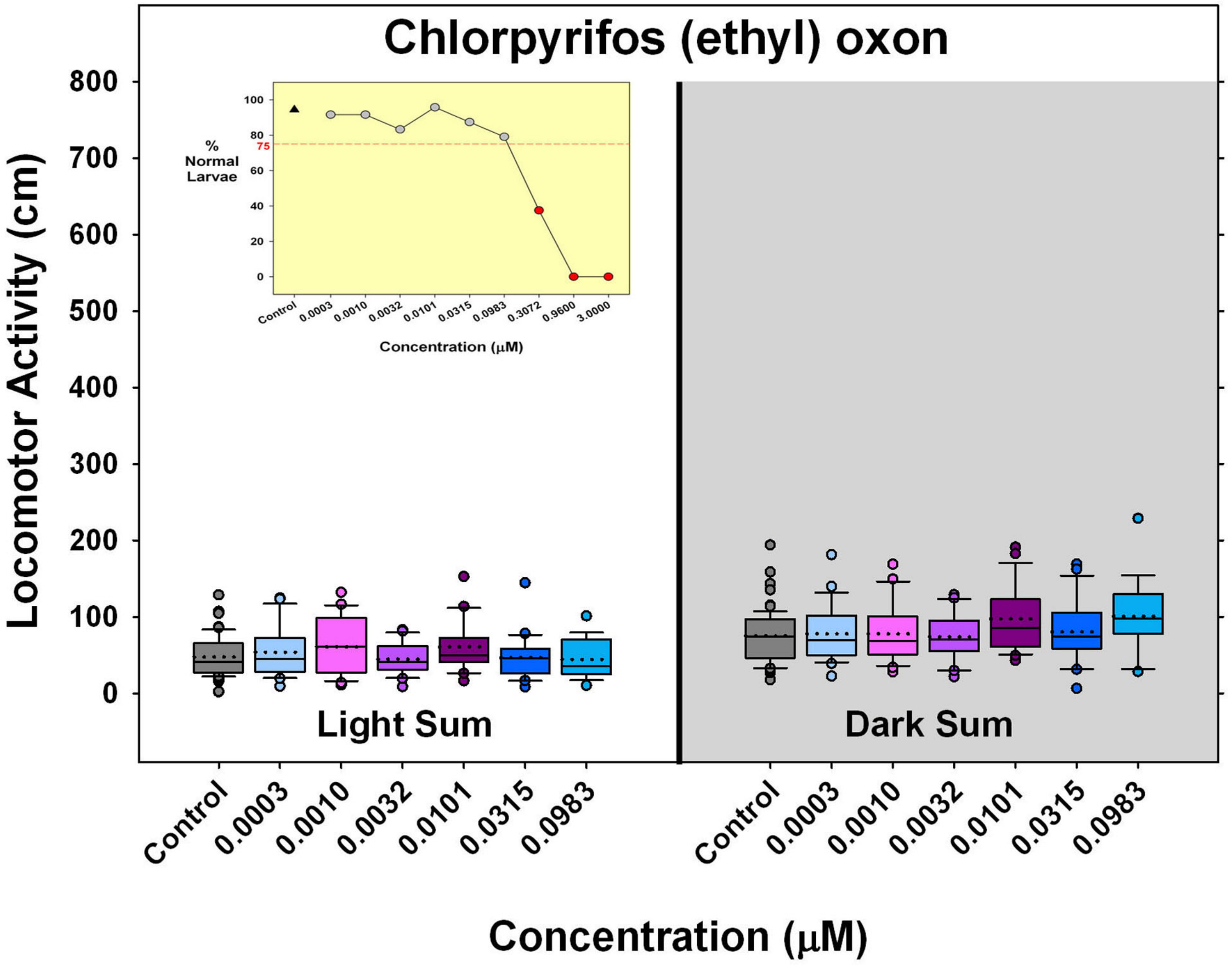

Chlorpyrifos (ethyl) oxon
Locomotor Activity (cm)
% Normal Larvae
75
Concentration (μM)
Control
0.0003
0.0010
0.0032
0.0101
0.0315
0.0983
0.3072
0.9600
3.0000
Light Sum
Dark Sum
Concentration (μM)

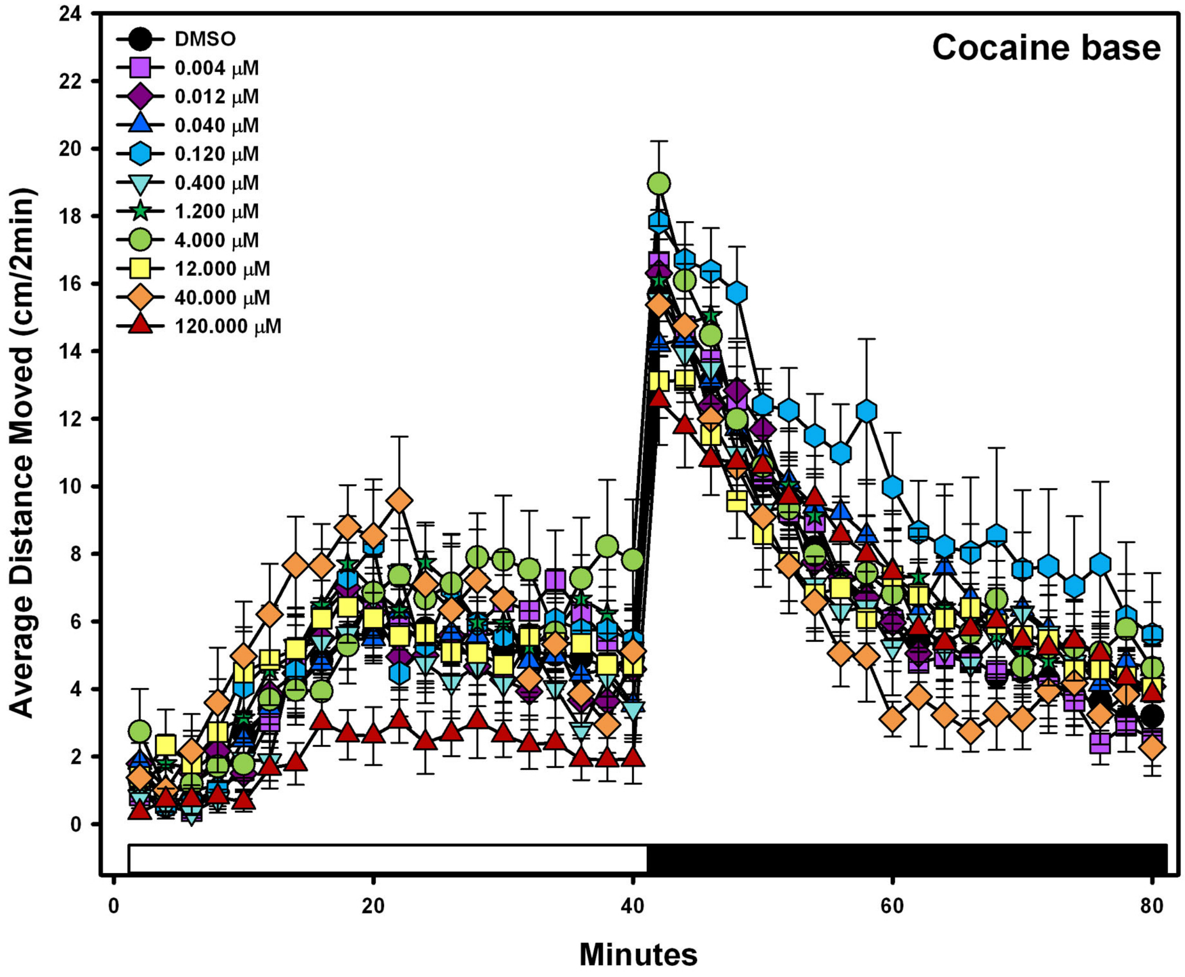

Cocaine base
DMSO
0.004 µM
0.012 µM
0.040 µM
0.120 µM
0.400 µM
1.200 µM
4.000 µM
12.000 µM
40.000 µM
120.000 µM
Average Distance Moved (cm/2min)
Minutes

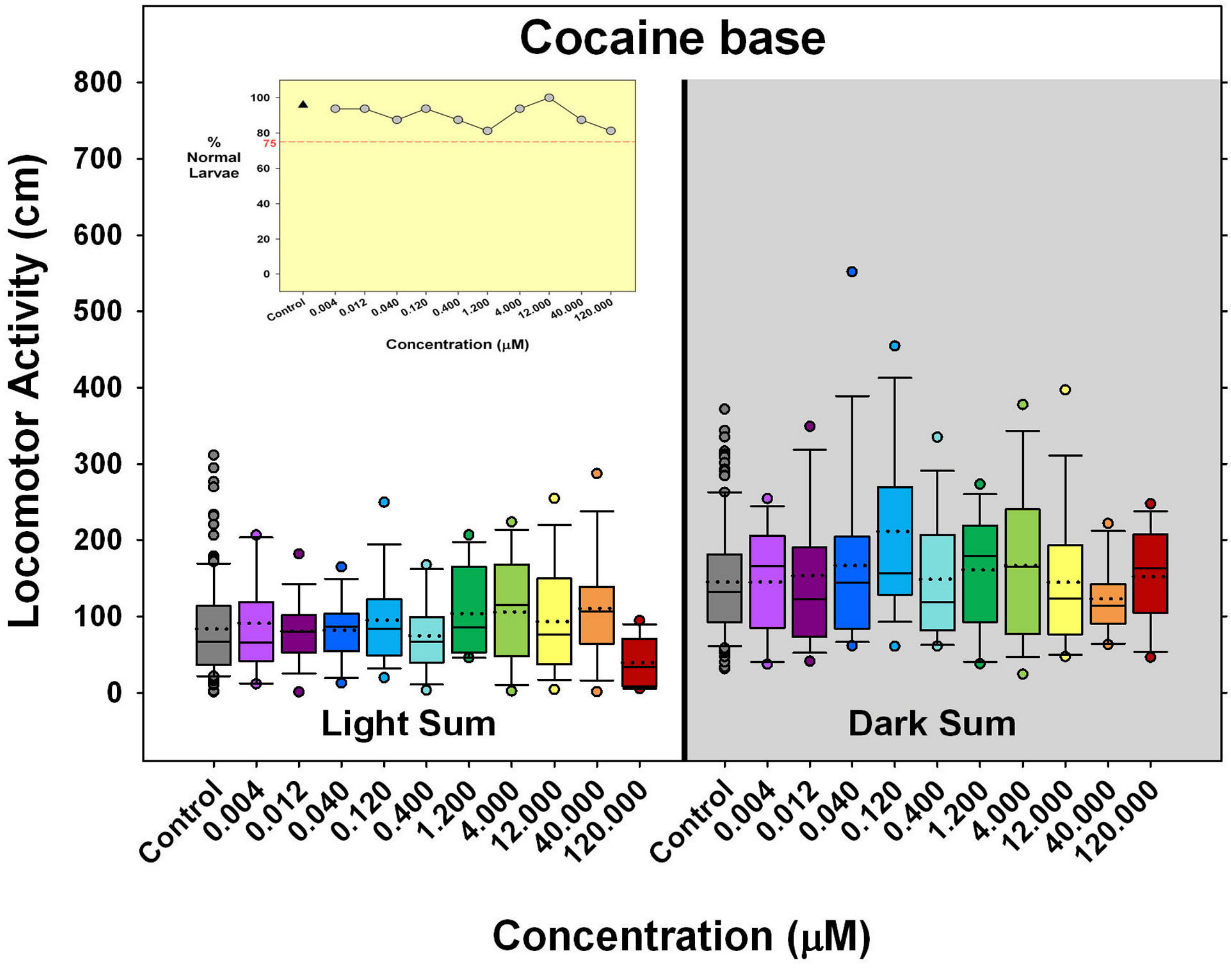

Cocaine base
Locomotor Activity (cm)
% Normal Larvae
Concentration (µM)
Light Sum
Dark Sum
Control
0.004
0.012
0.040
0.120
0.400
1.200
4.000
12.000
40.000
120.000
Concentration (µM)

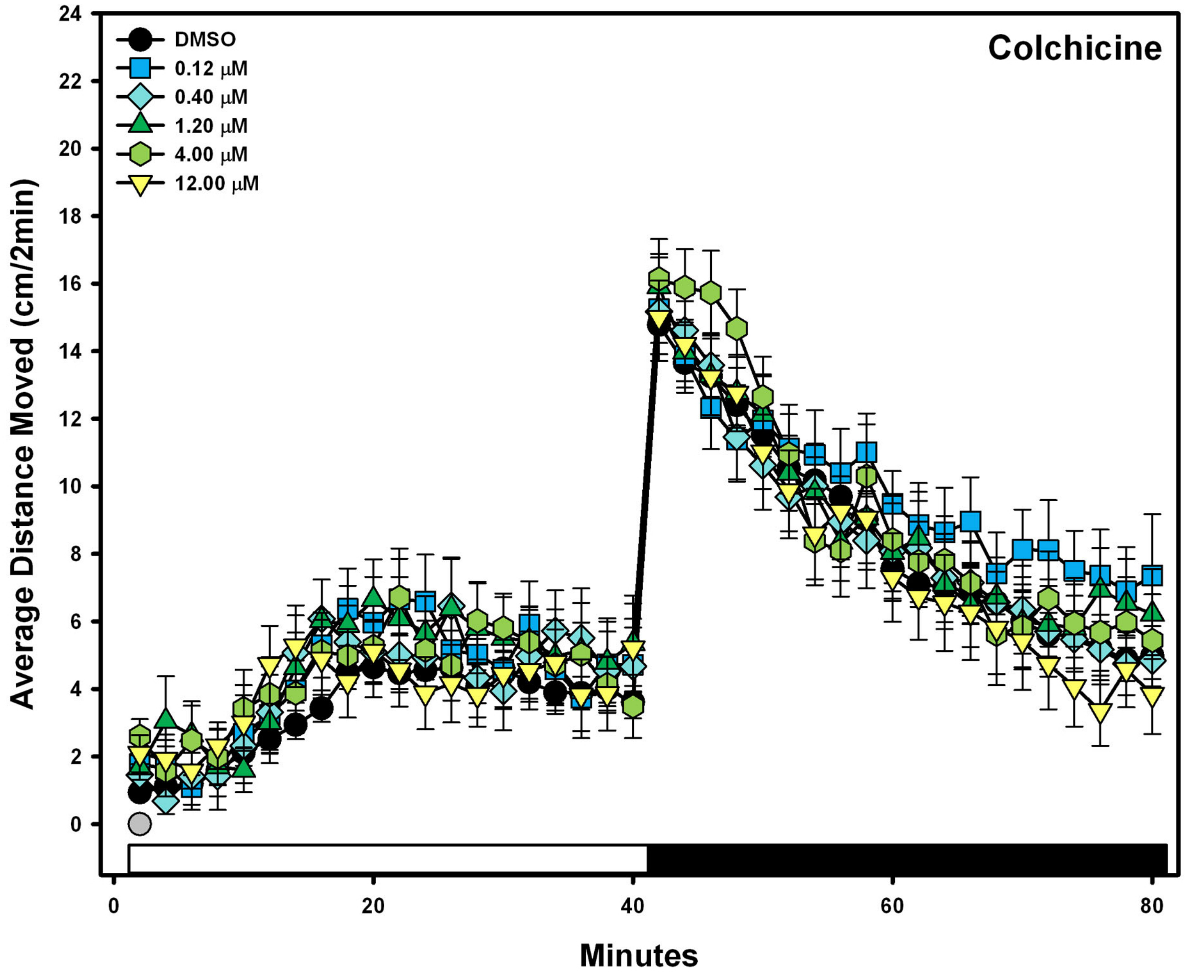

Colchicine
DMSO
0.12 μM
0.40 μM
1.20 μM
4.00 μM
12.00 μM
Average Distance Moved (cm/2min)
Minutes

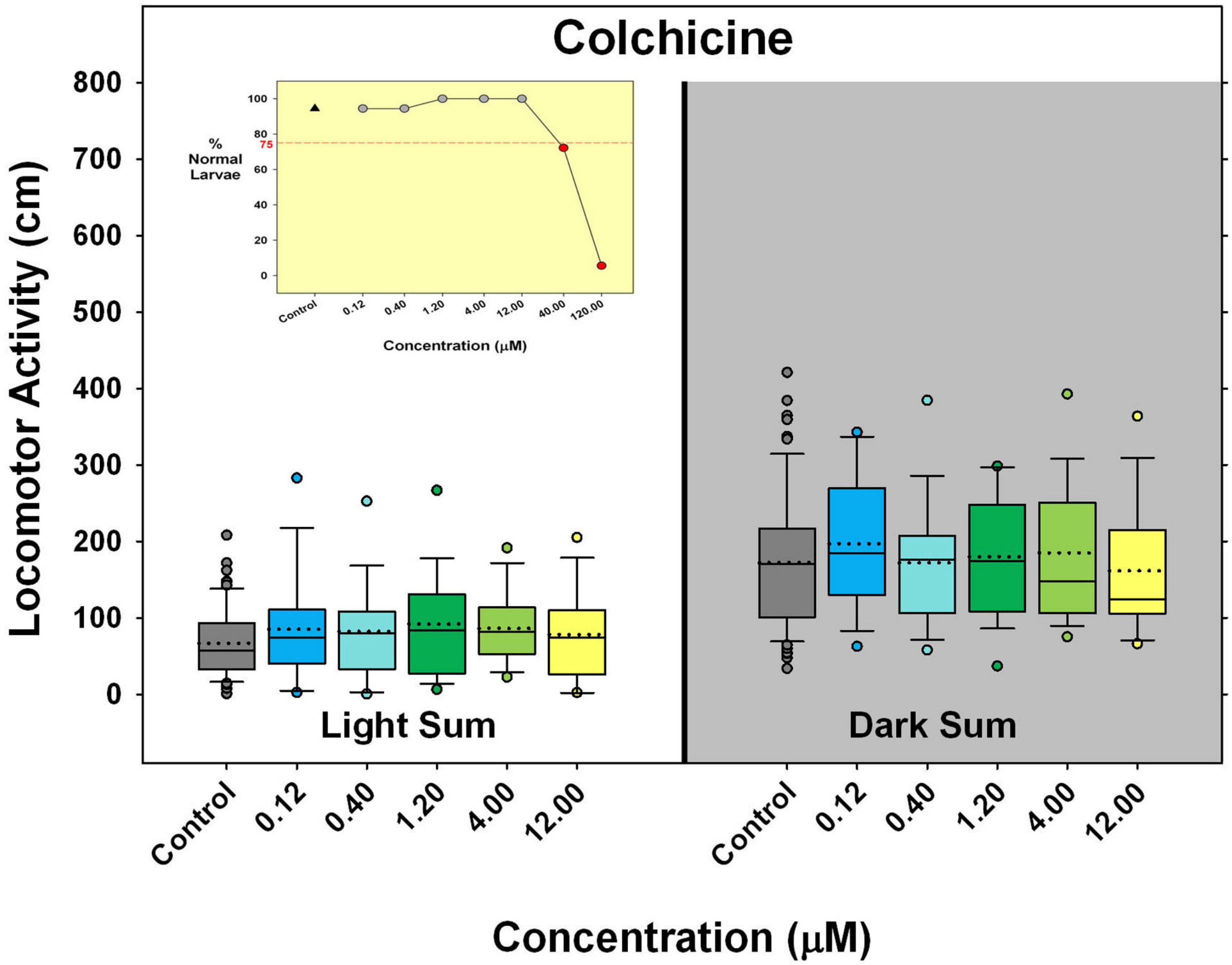

Colchicine
Locomotor Activity (cm)
% Normal Larvae
Concentration (μM)
Light Sum
Dark Sum
Control
0.12
0.40
1.20
4.00
12.00
Concentration (μM)

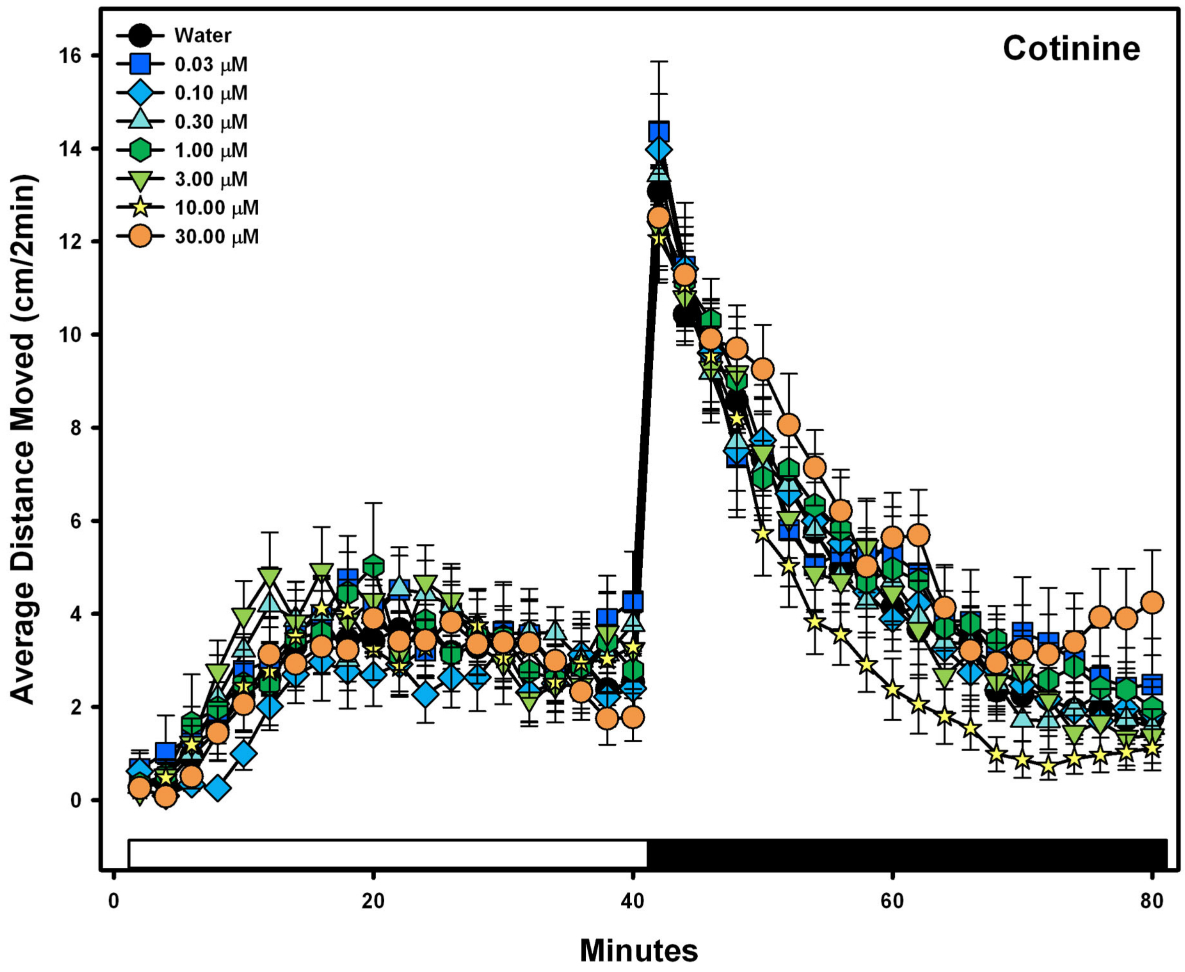

Water
0.03 μM
0.10 μM
0.30 μM
1.00 μM
3.00 μM
10.00 μM
30.00 μM
Cotinine
Average Distance Moved (cm/2min)
Minutes

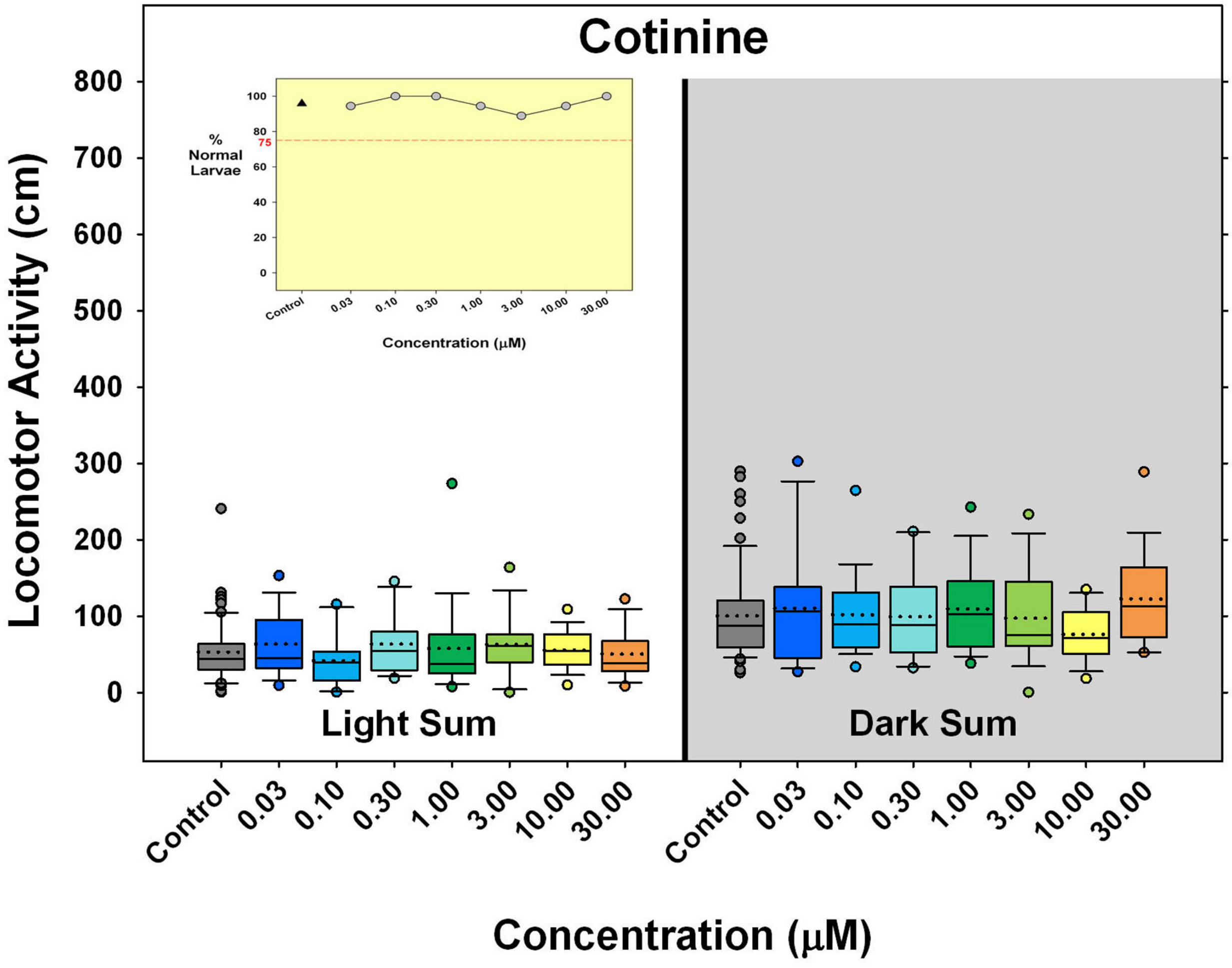

Cotinine
Locomotor Activity (cm)
% Normal Larvae
75
Concentration (μM)
Light Sum
Dark Sum
Control
0.03
0.10
0.30
1.00
3.00
10.00
30.00
Concentration (μM)

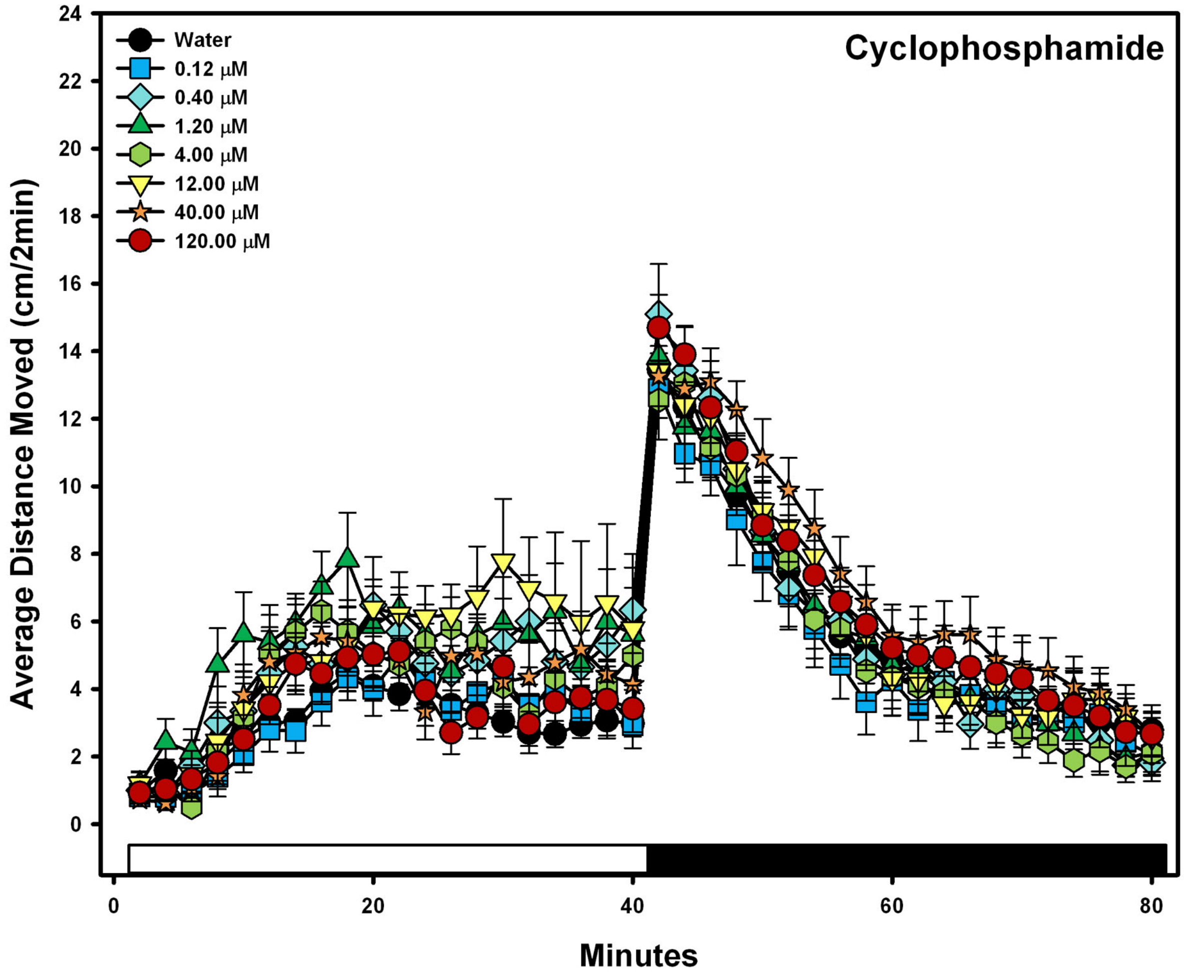
Cyclophosphamide
Water
0.12 μM
0.40 μM
1.20 μM
4.00 μM
12.00 μM
40.00 μM
120.00 μM
Average Distance Moved (cm/2min)
Minutes

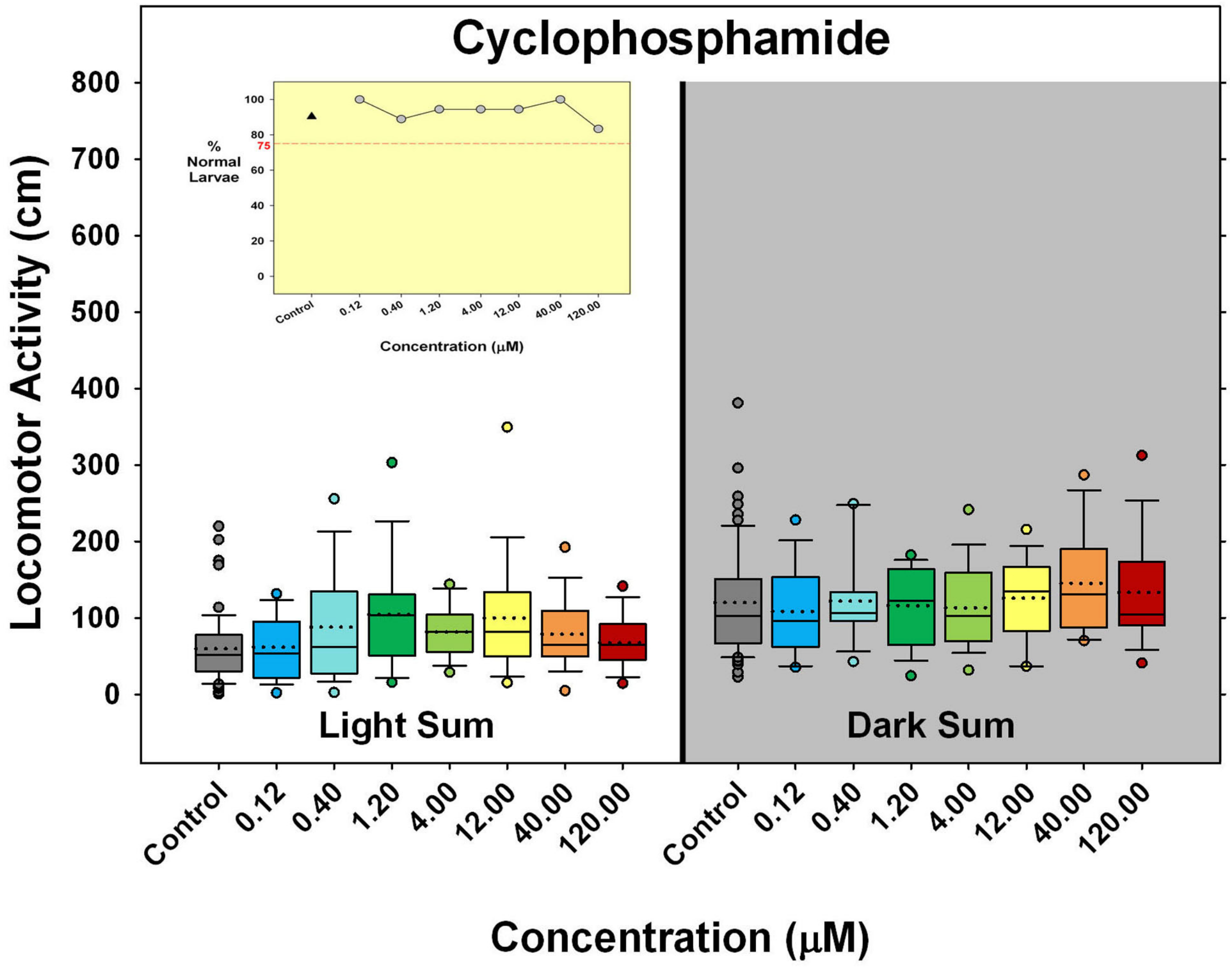
Cyclophosphamide
Locomotor Activity (cm)
% Normal Larvae
Concentration (μM)
Light Sum
Dark Sum
Control
0.12
0.40
1.20
4.00
12.00
40.00
120.00
Concentration (μM)

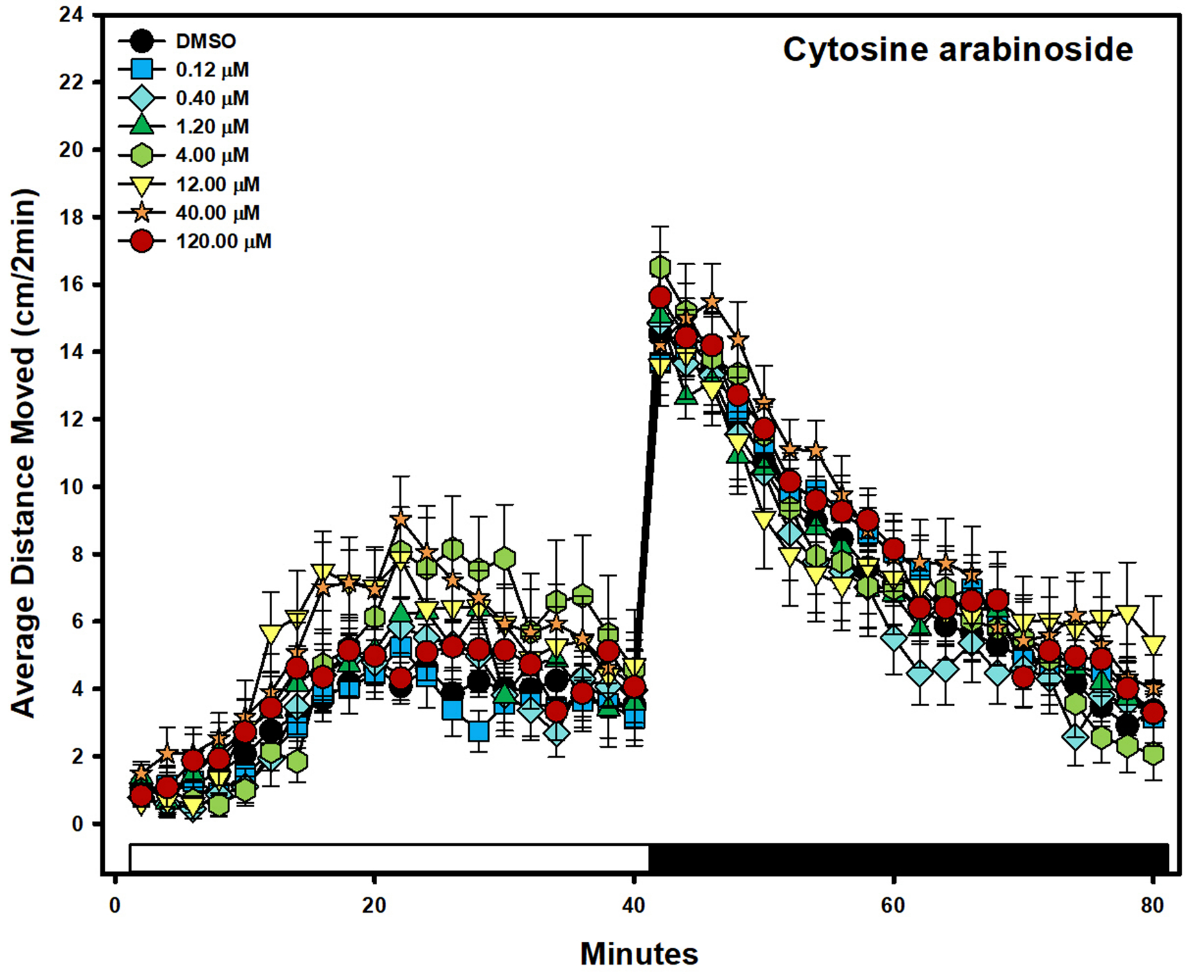

Cytosine arabinoside
DMSO
0.12 µM
0.40 µM
1.20 µM
4.00 µM
12.00 µM
40.00 µM
120.00 µM
Average Distance Moved (cm/2min)
Minutes

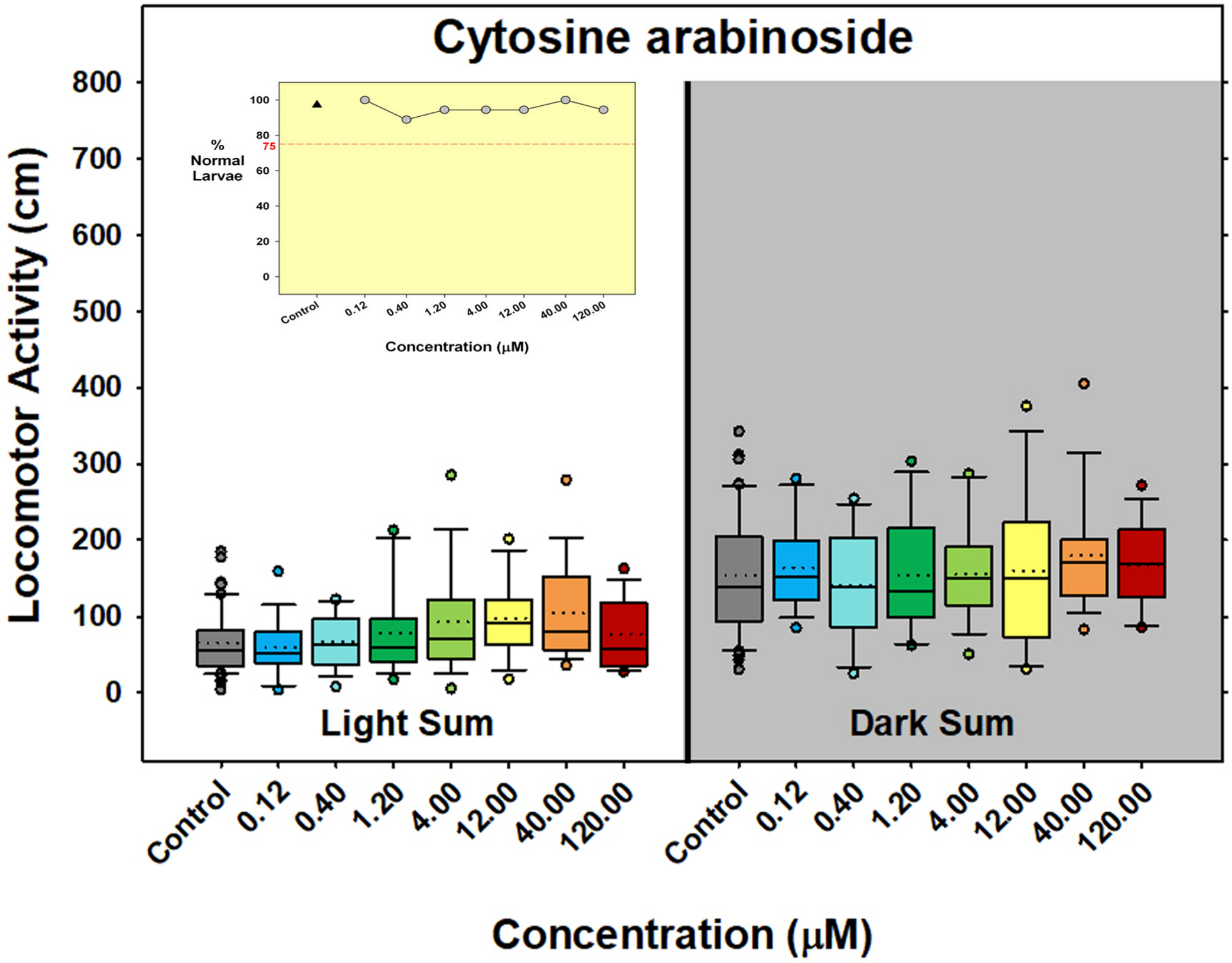

Cytosine arabinoside
Locomotor Activity (cm)
% Normal Larvae
Concentration (µM)
Control
0.12
0.40
1.20
4.00
12.00
40.00
120.00
Light Sum
Dark Sum
Concentration (µM)

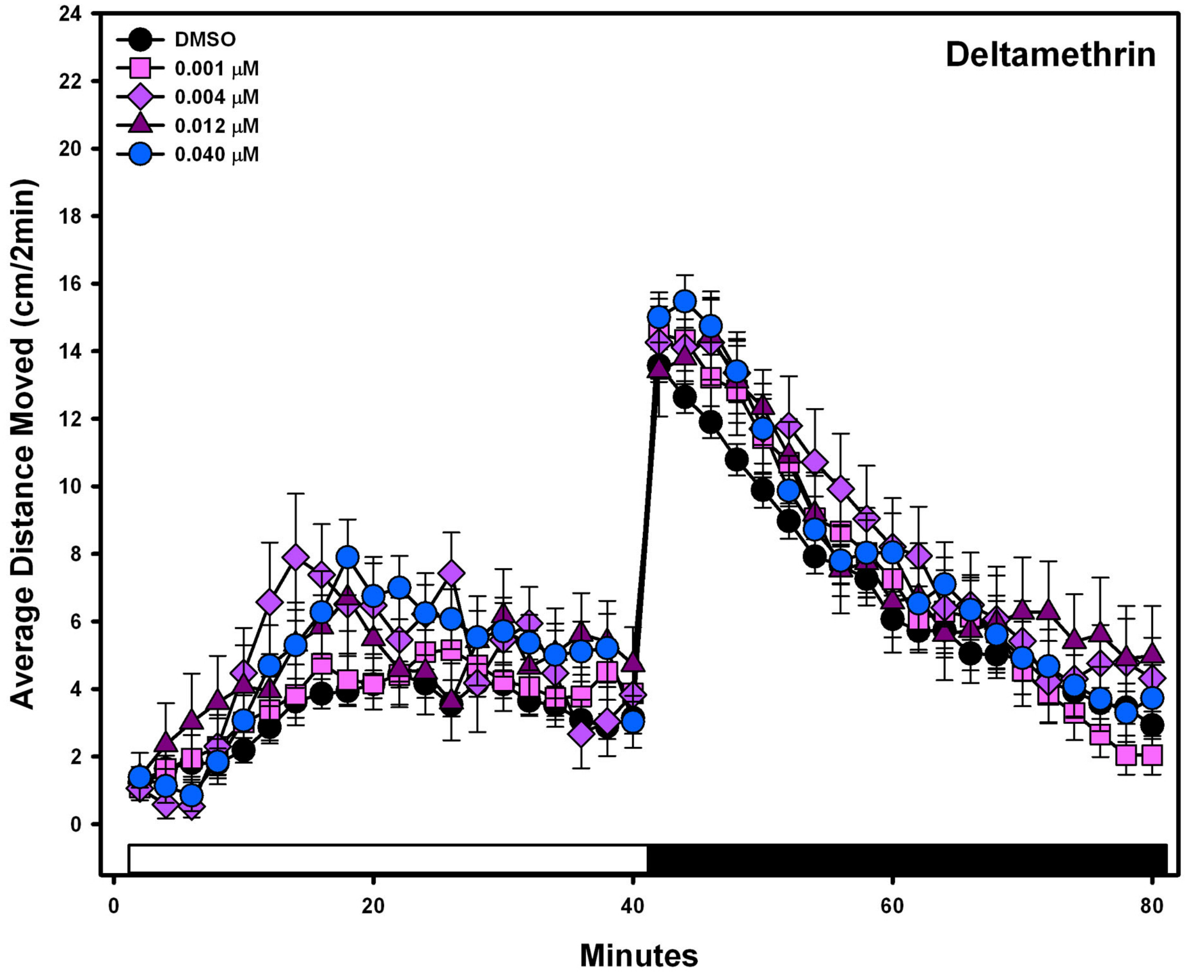
DMSO
0.001 μM
0.004 μM
0.012 μM
0.040 μM
Deltamethrin
Average Distance Moved (cm/2min)
Minutes

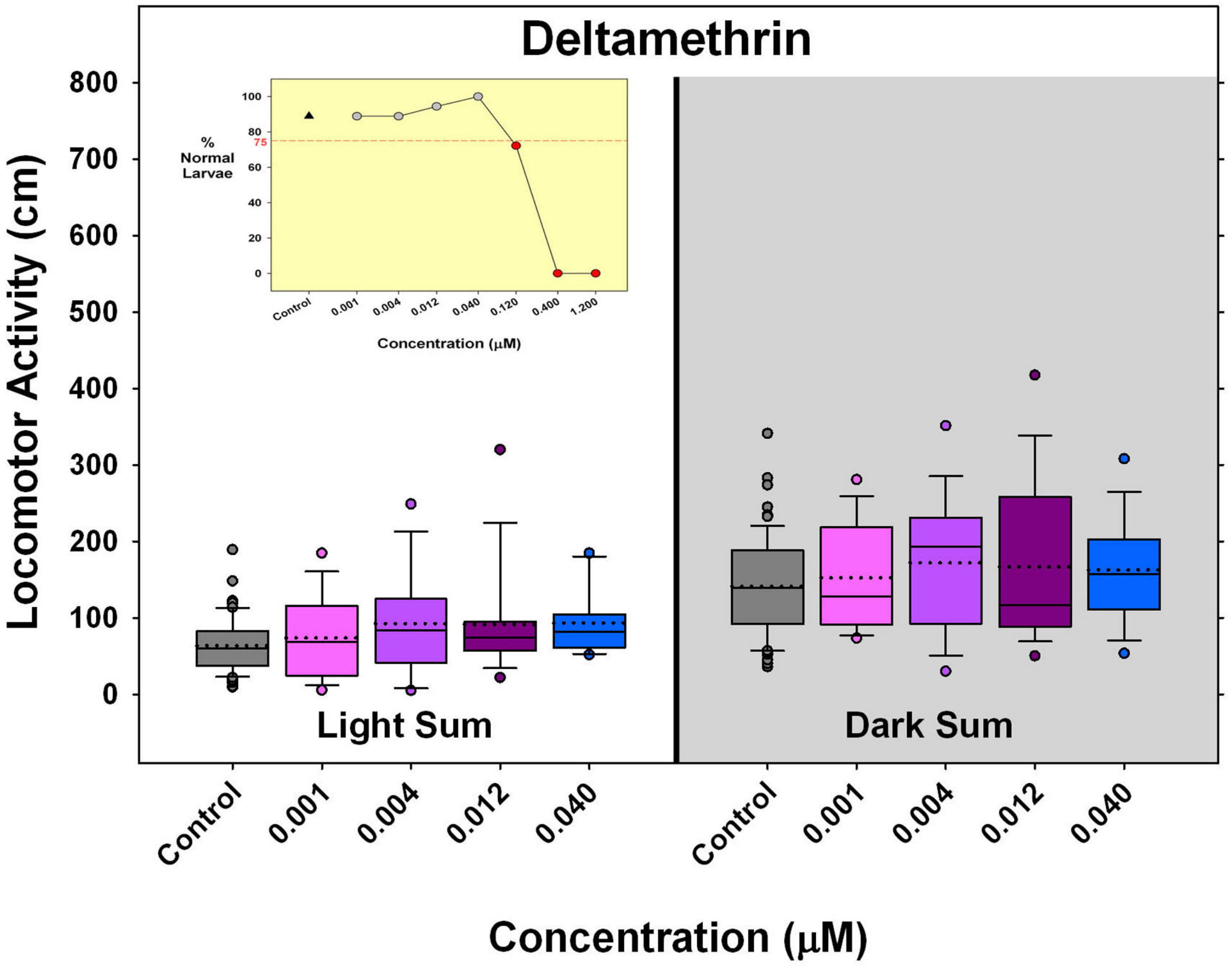
Deltamethrin
Locomotor Activity (cm)
% Normal Larvae
Concentration (μM)
Light Sum
Dark Sum
Control
0.001
0.004
0.012
0.040
Concentration (μM)

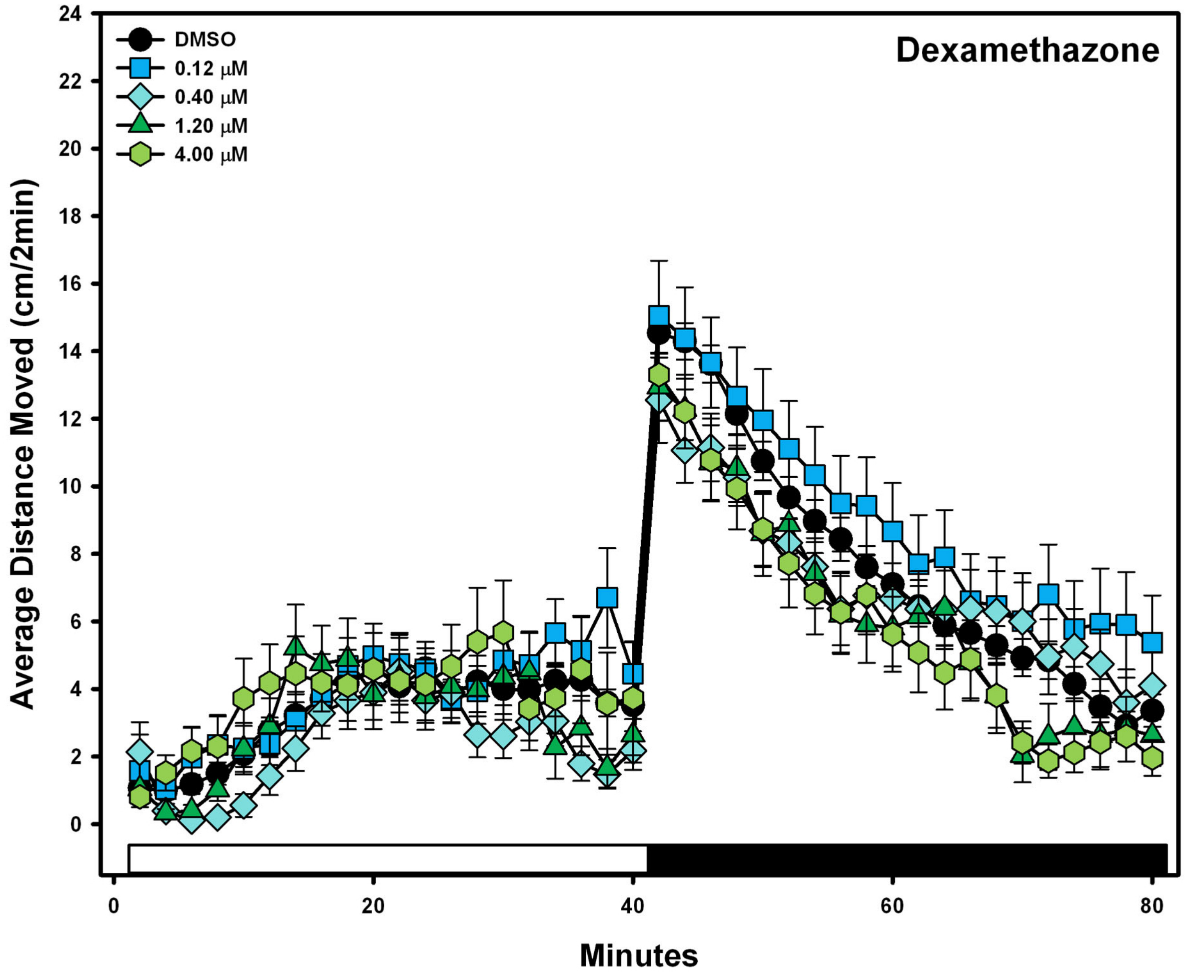
Dexamethazone
DMSO
0.12 µM
0.40 µM
1.20 µM
4.00 µM
Average Distance Moved (cm/2min)
Minutes
0
20
40
60
80

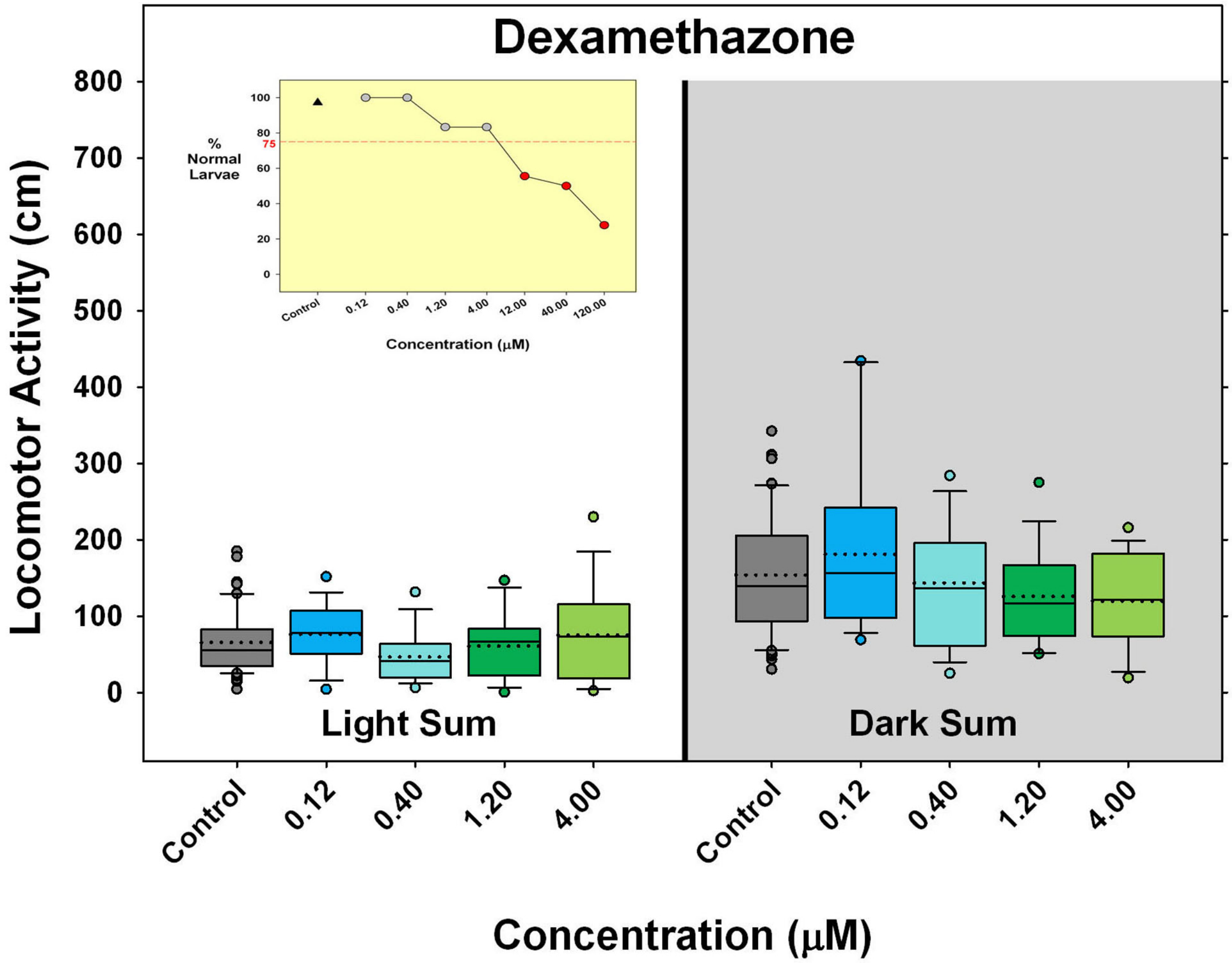
Dexamethazone
Locomotor Activity (cm)
% Normal Larvae
Concentration (µM)
Control
0.12
0.40
1.20
4.00
12.00
40.00
120.00
Light Sum
Dark Sum
Concentration (µM)

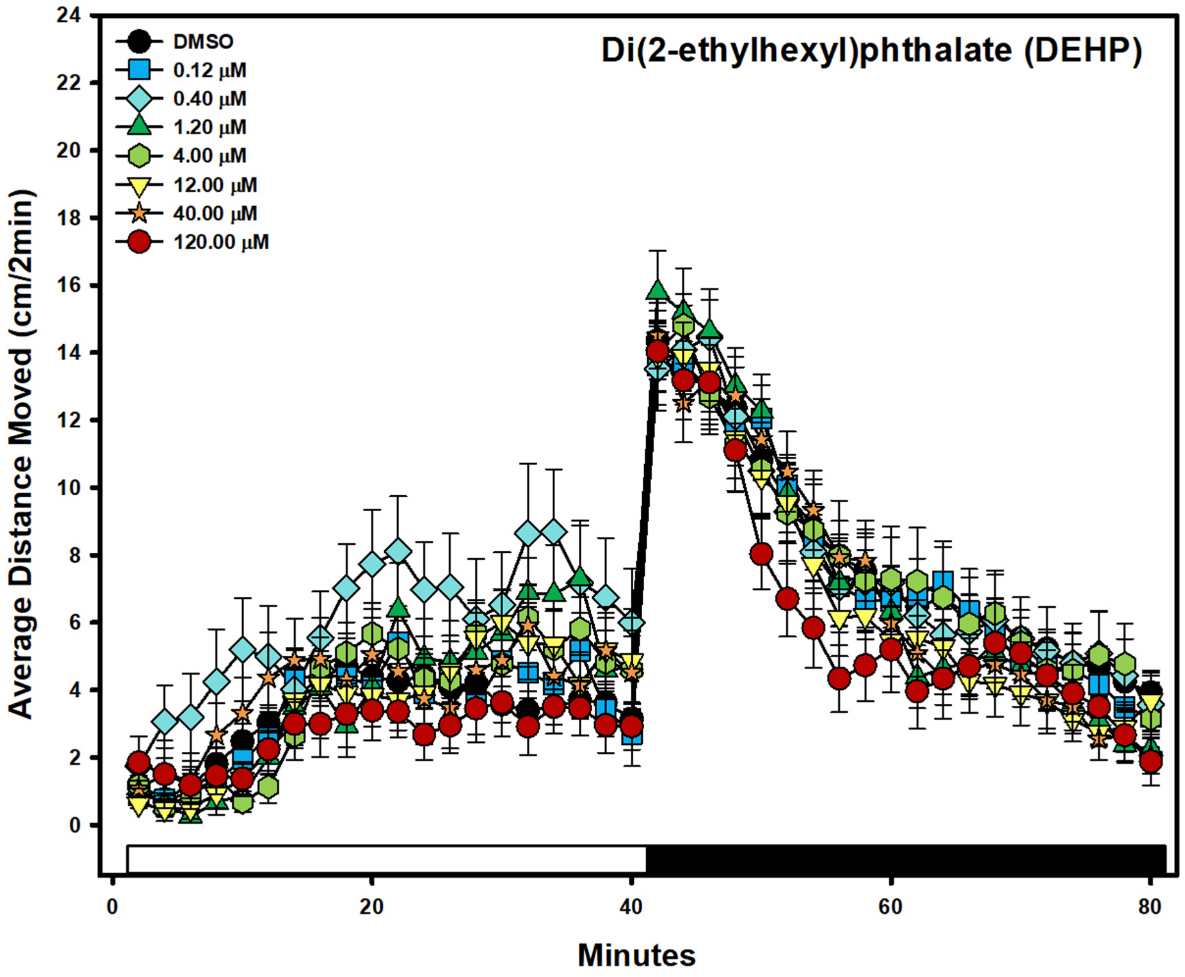
Di(2-ethylhexyl)phthalate (DEHP)
DMSO
0.12 µM
0.40 µM
1.20 µM
4.00 µM
12.00 µM
40.00 µM
120.00 µM
Average Distance Moved (cm/2min)
Minutes

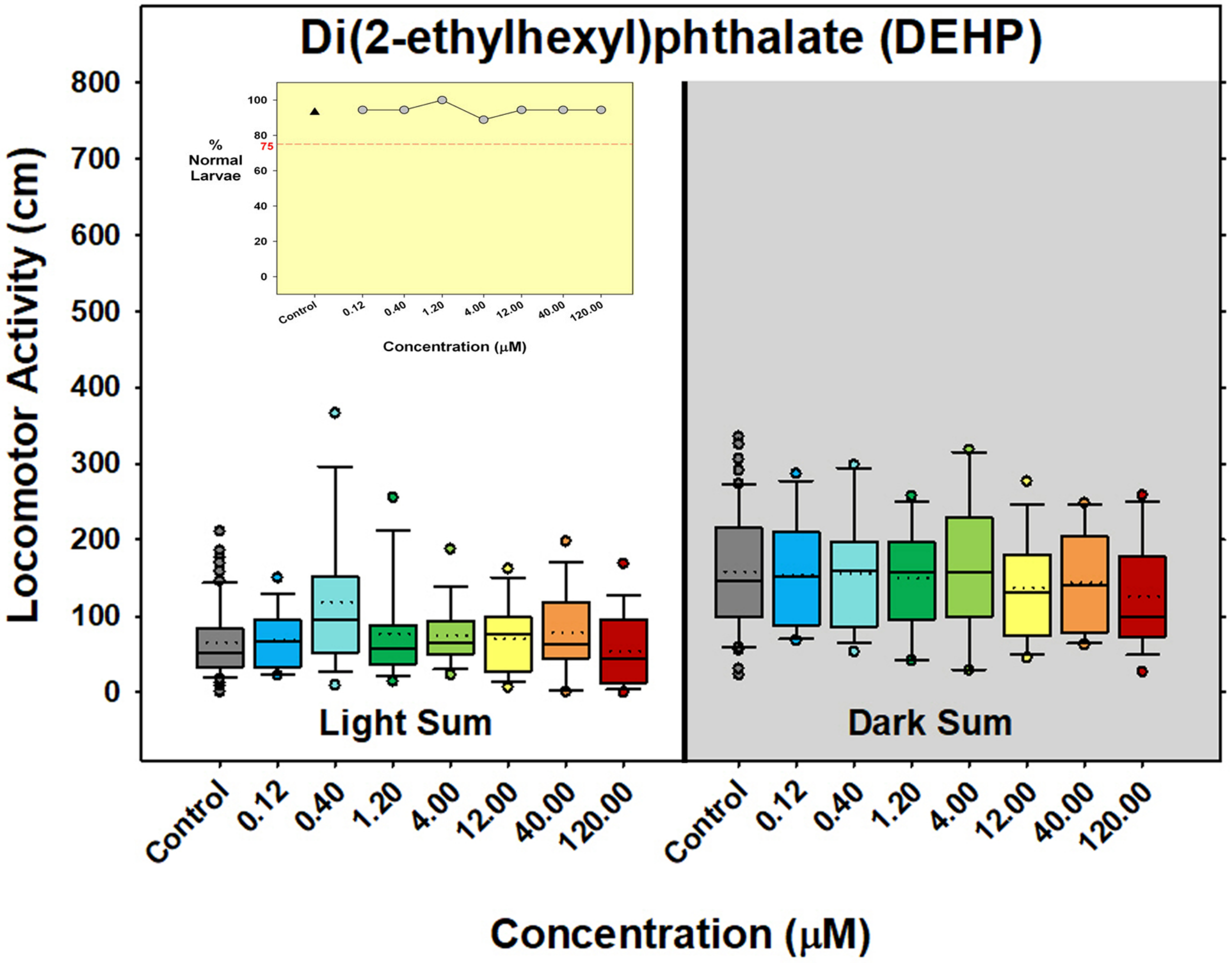
Di(2-ethylhexyl)phthalate (DEHP)
Locomotor Activity (cm)
% Normal Larvae
Concentration (µM)
Light Sum
Dark Sum
Control
0.12
0.40
1.20
4.00
12.00
40.00
120.00
Concentration (µM)

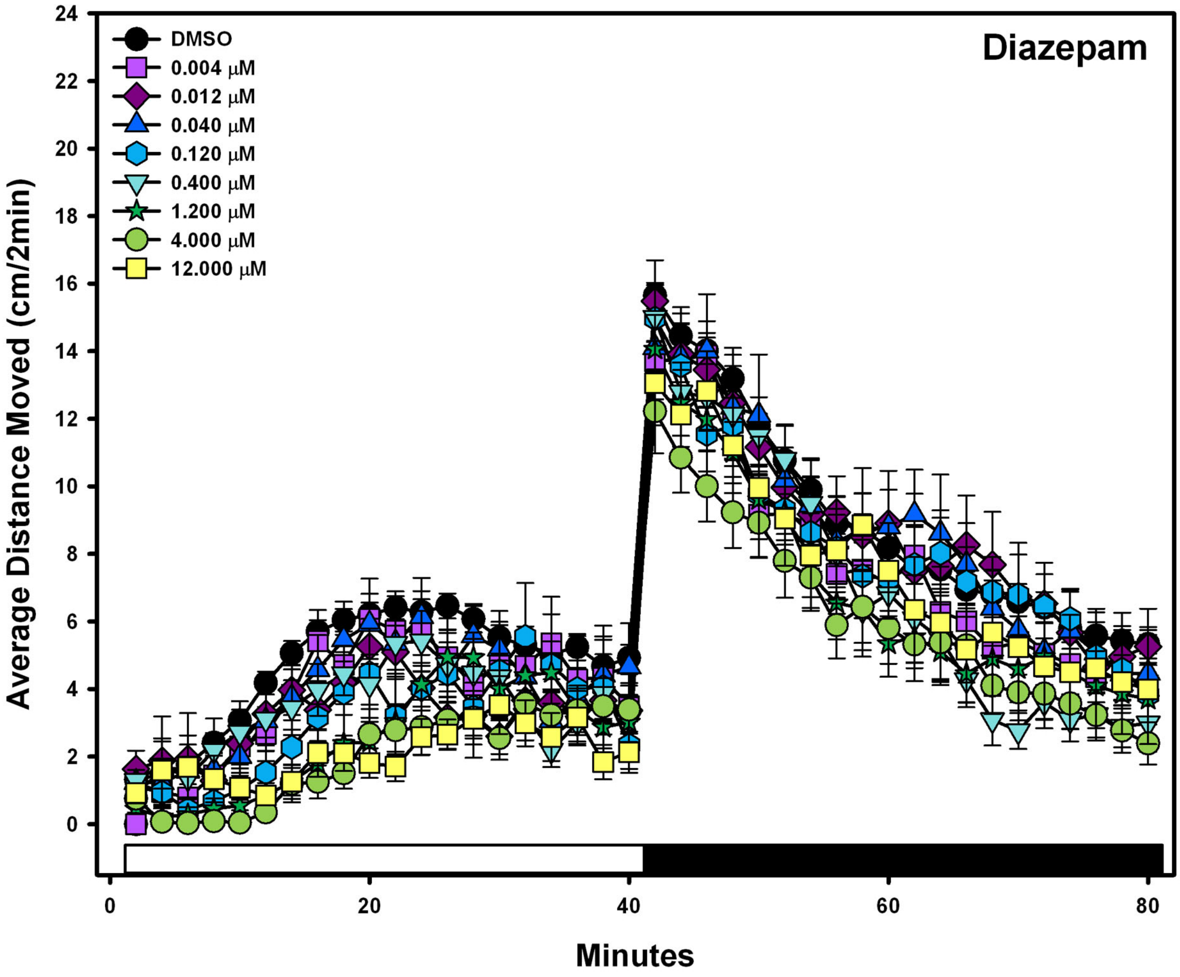
Diazepam
DMSO
0.004 µM
0.012 µM
0.040 µM
0.120 µM
0.400 µM
1.200 µM
4.000 µM
12.000 µM
Average Distance Moved (cm/2min)
Minutes
0
20
40
60
80
0
2
4
6
8
10
12
14
16
18
20
22
24

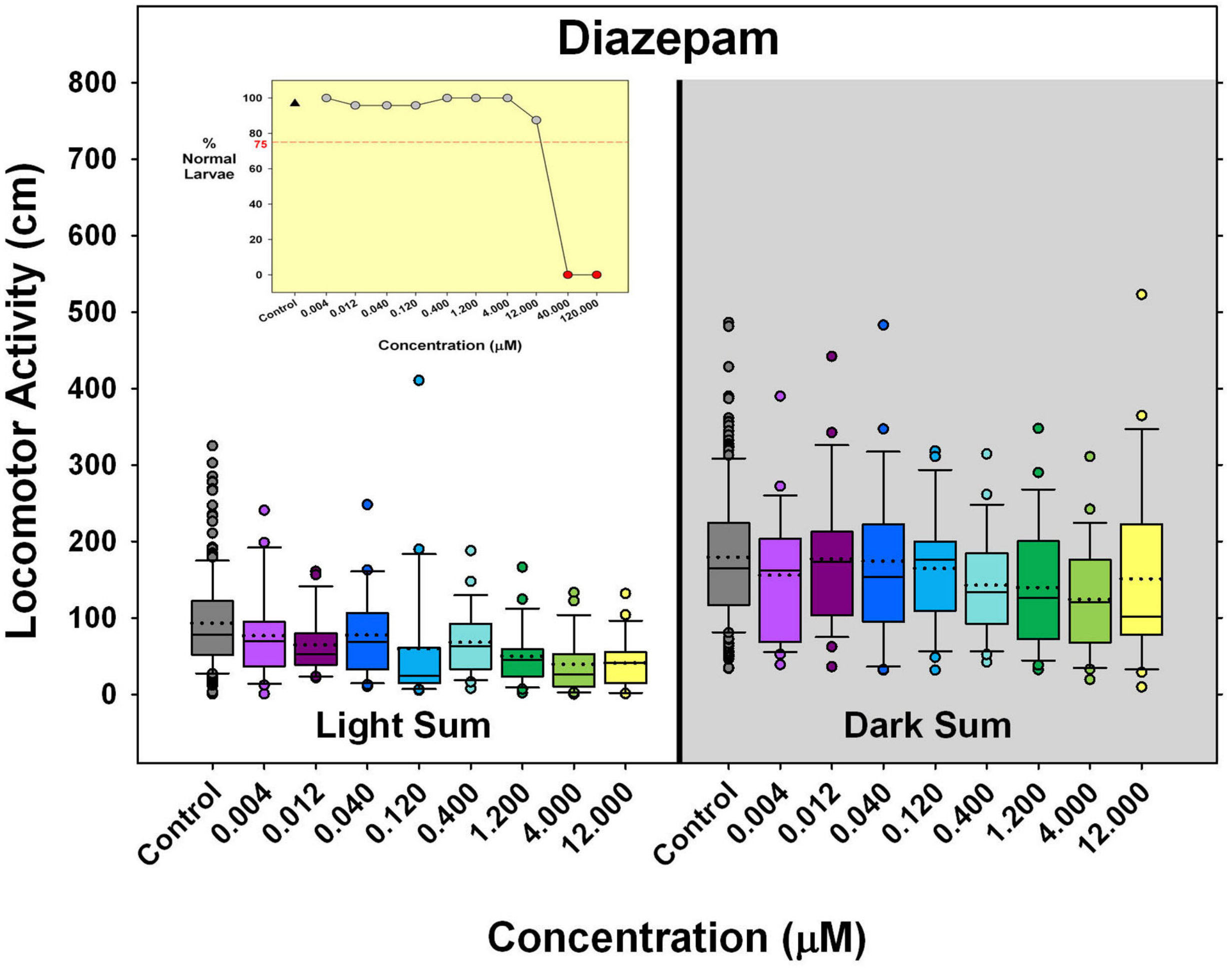
Diazepam
Locomotor Activity (cm)
0
100
200
300
400
500
600
700
800
% Normal Larvae
75
Control
0.004
0.012
0.040
0.120
0.400
1.200
4.000
12.000
40.000
120.000
Concentration (µM)
Light Sum
Dark Sum
Control
0.004
0.012
0.040
0.120
0.400
1.200
4.000
12.000
Control
0.004
0.012
0.040
0.120
0.400
1.200
4.000
12.000
Concentration (µM)

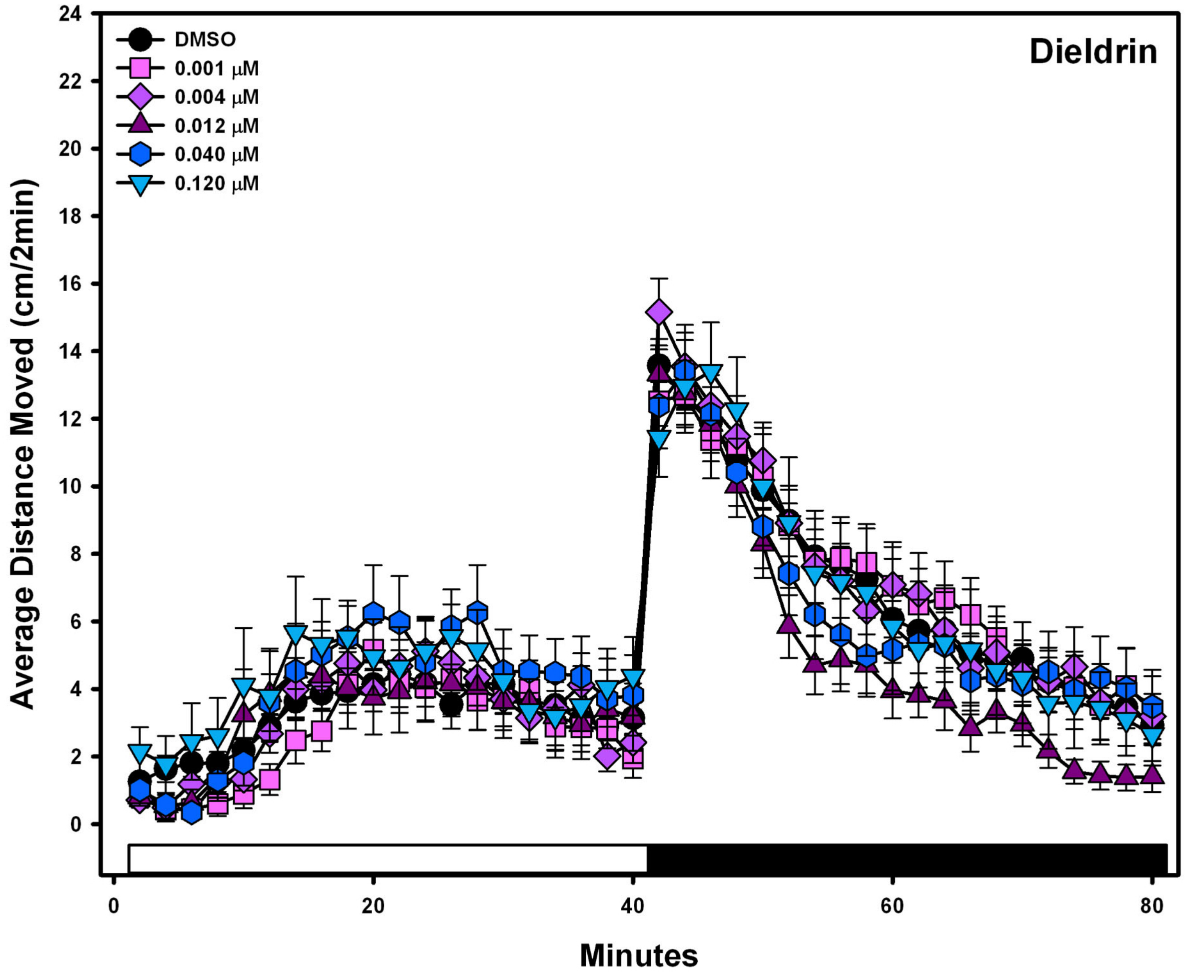
Dieldrin
DMSO
0.001 µM
0.004 µM
0.012 µM
0.040 µM
0.120 µM
Average Distance Moved (cm/2min)
Minutes

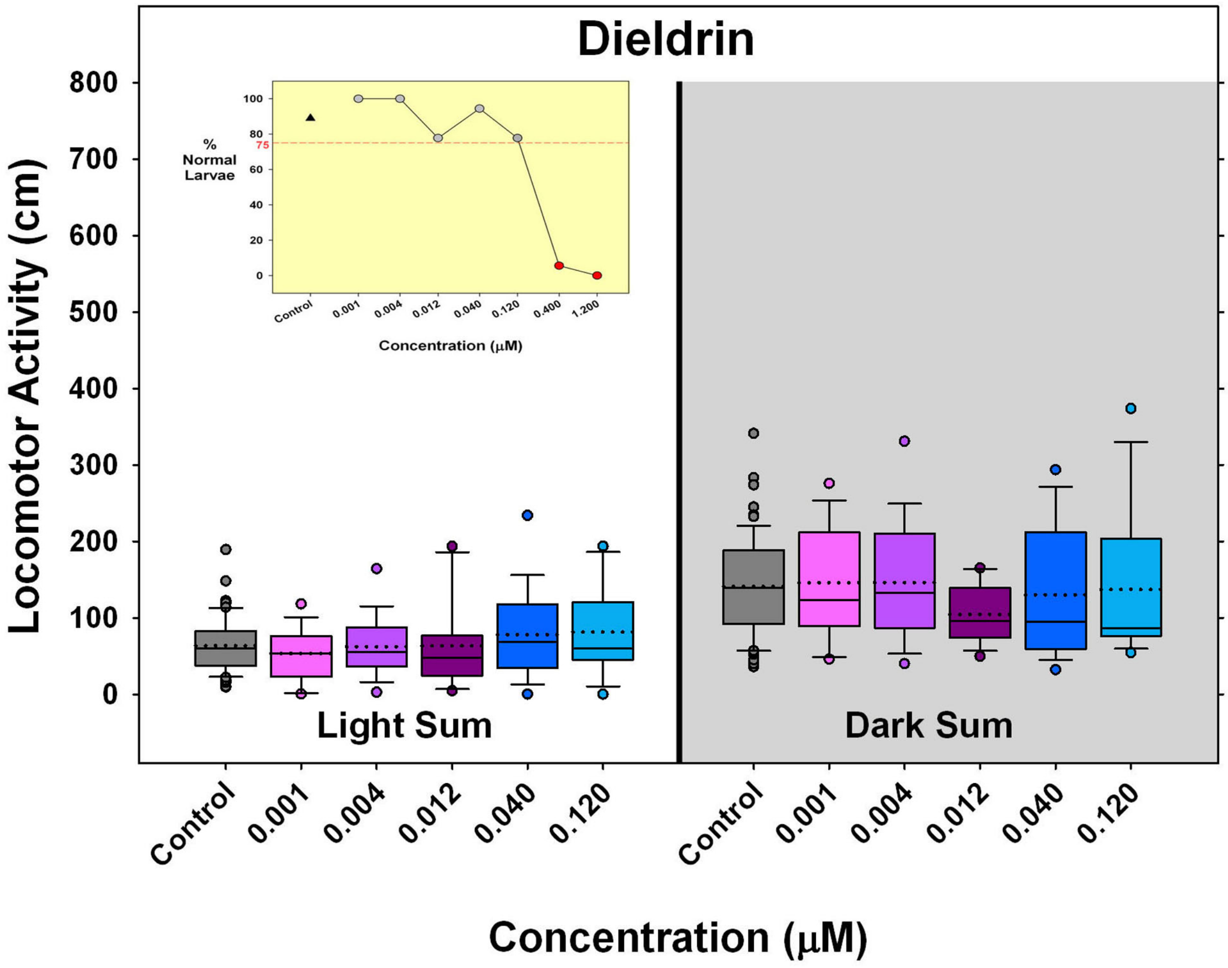
Dieldrin
Locomotor Activity (cm)
% Normal Larvae
Concentration (µM)
Light Sum
Dark Sum
Control
0.001
0.004
0.012
0.040
0.120
Concentration (µM)

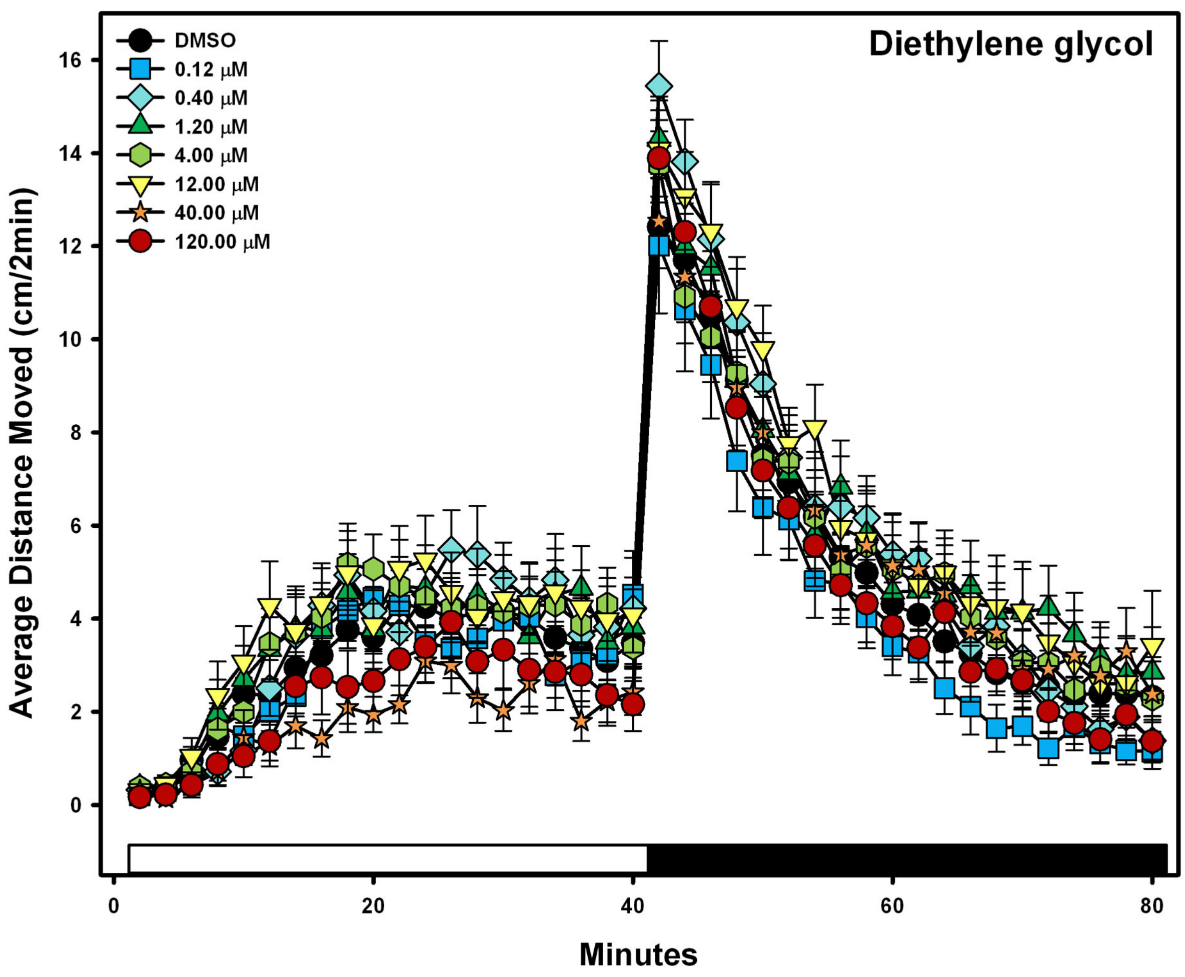

Diethylene glycol
DMSO
0.12 µM
0.40 µM
1.20 µM
4.00 µM
12.00 µM
40.00 µM
120.00 µM
Average Distance Moved (cm/2min)
Minutes
0
2
4
6
8
10
12
14
16
20
40
60
80

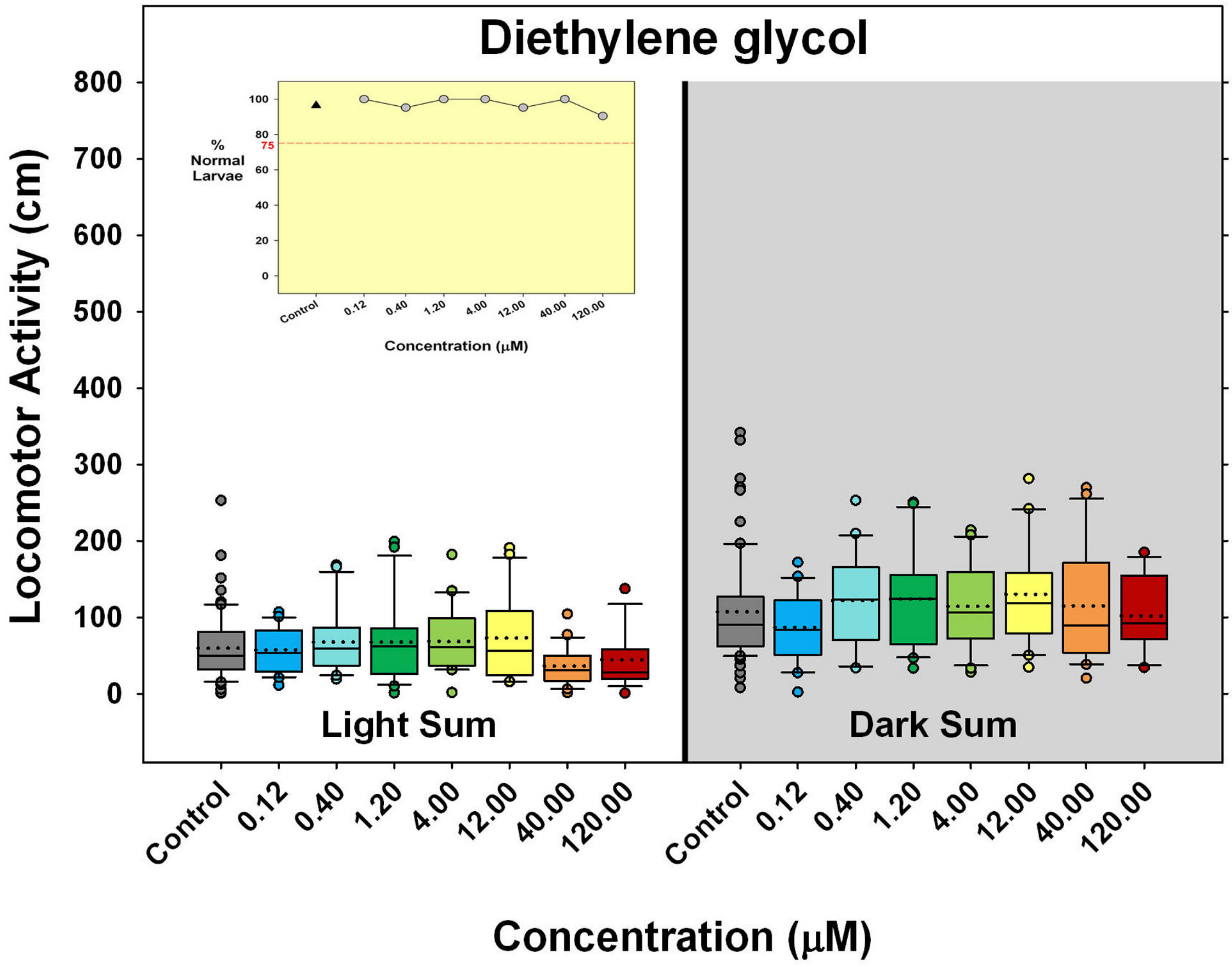

Diethylene glycol
Locomotor Activity (cm)
% Normal Larvae
75
Concentration (µM)
Light Sum
Dark Sum
Control
0.12
0.40
1.20
4.00
12.00
40.00
120.00
0
100
200
300
400
500
600
700
800

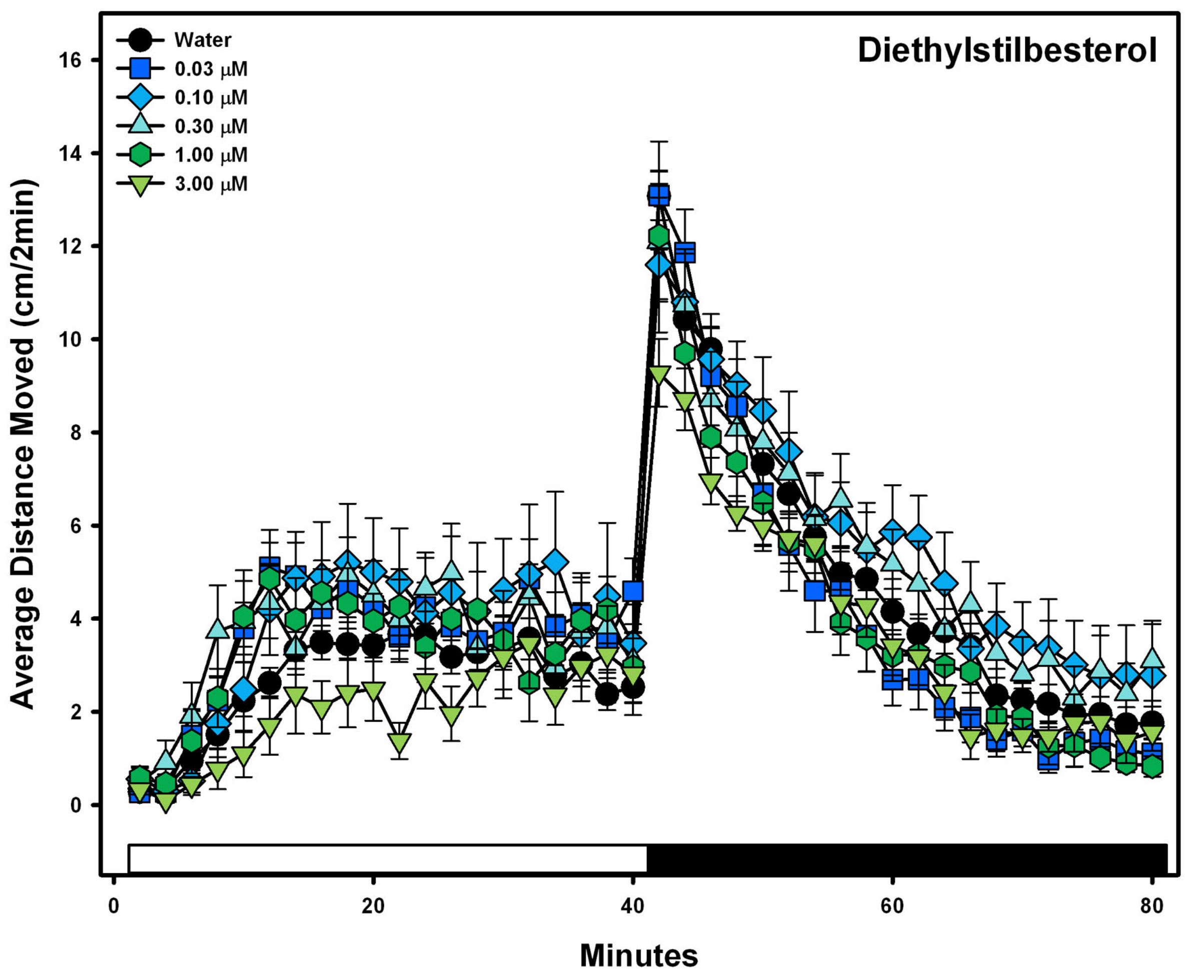

Water
0.03 μM
0.10 μM
0.30 μM
1.00 μM
3.00 μM
Diethylstilbesterol
Average Distance Moved (cm/2min)
Minutes

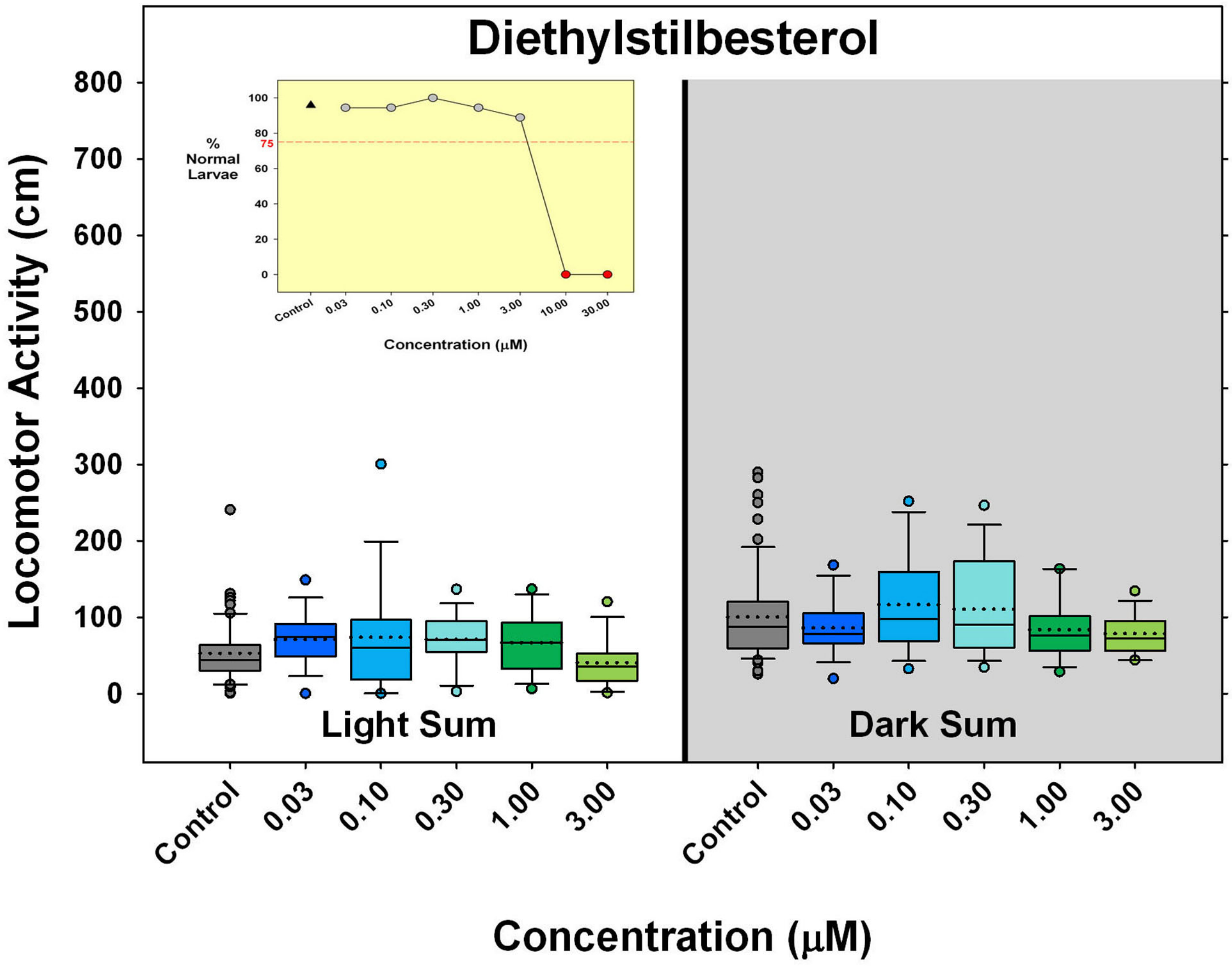

Diethylstilbesterol
Locomotor Activity (cm)
% Normal Larvae
Concentration (μM)
Light Sum
Dark Sum
Control
0.03
0.10
0.30
1.00
3.00
10.00
30.00
Concentration (μM)

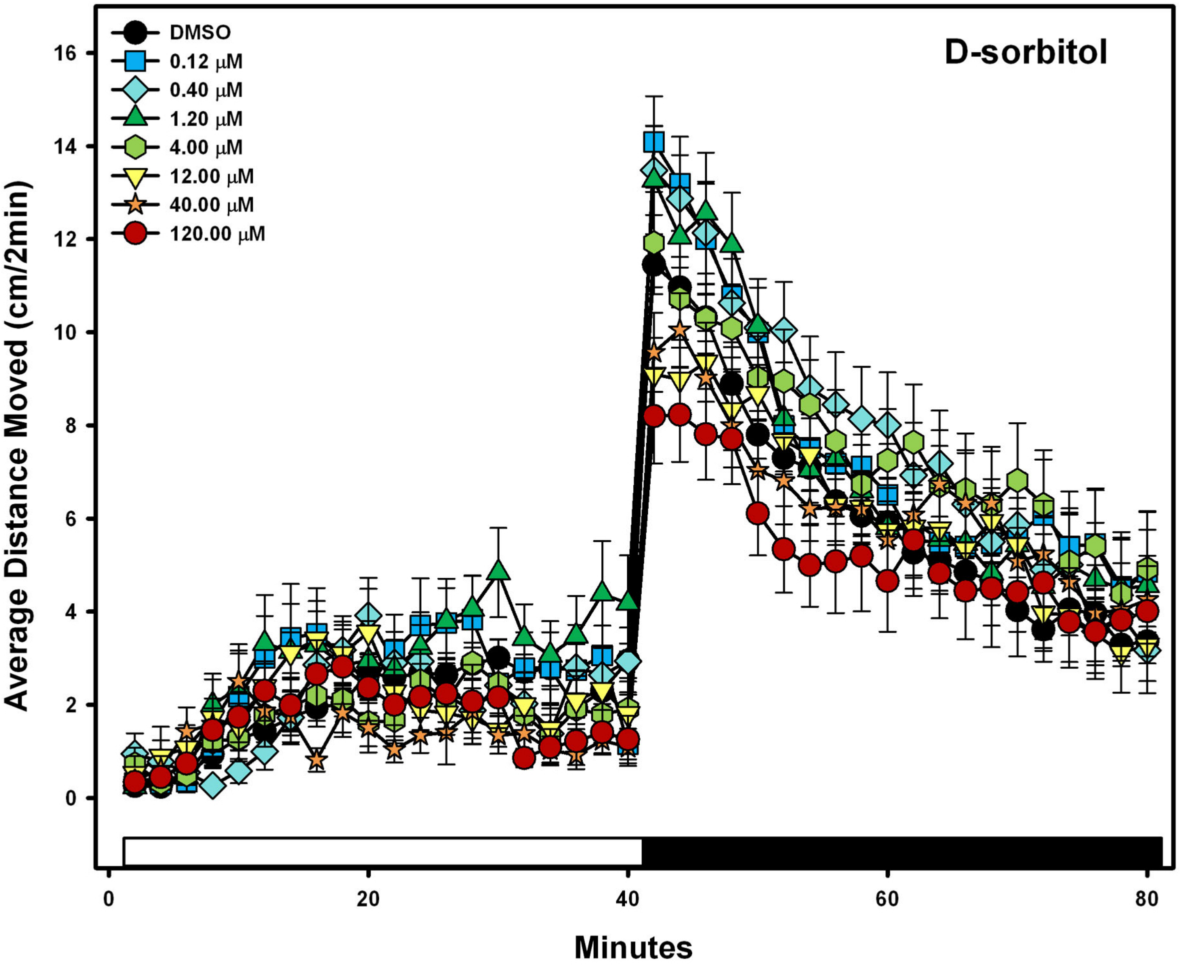
D-sorbitol
DMSO
0.12 µM
0.40 µM
1.20 µM
4.00 µM
12.00 µM
40.00 µM
120.00 µM
Average Distance Moved (cm/2min)
Minutes
0
20
40
60
80
0
2
4
6
8
10
12
14
16

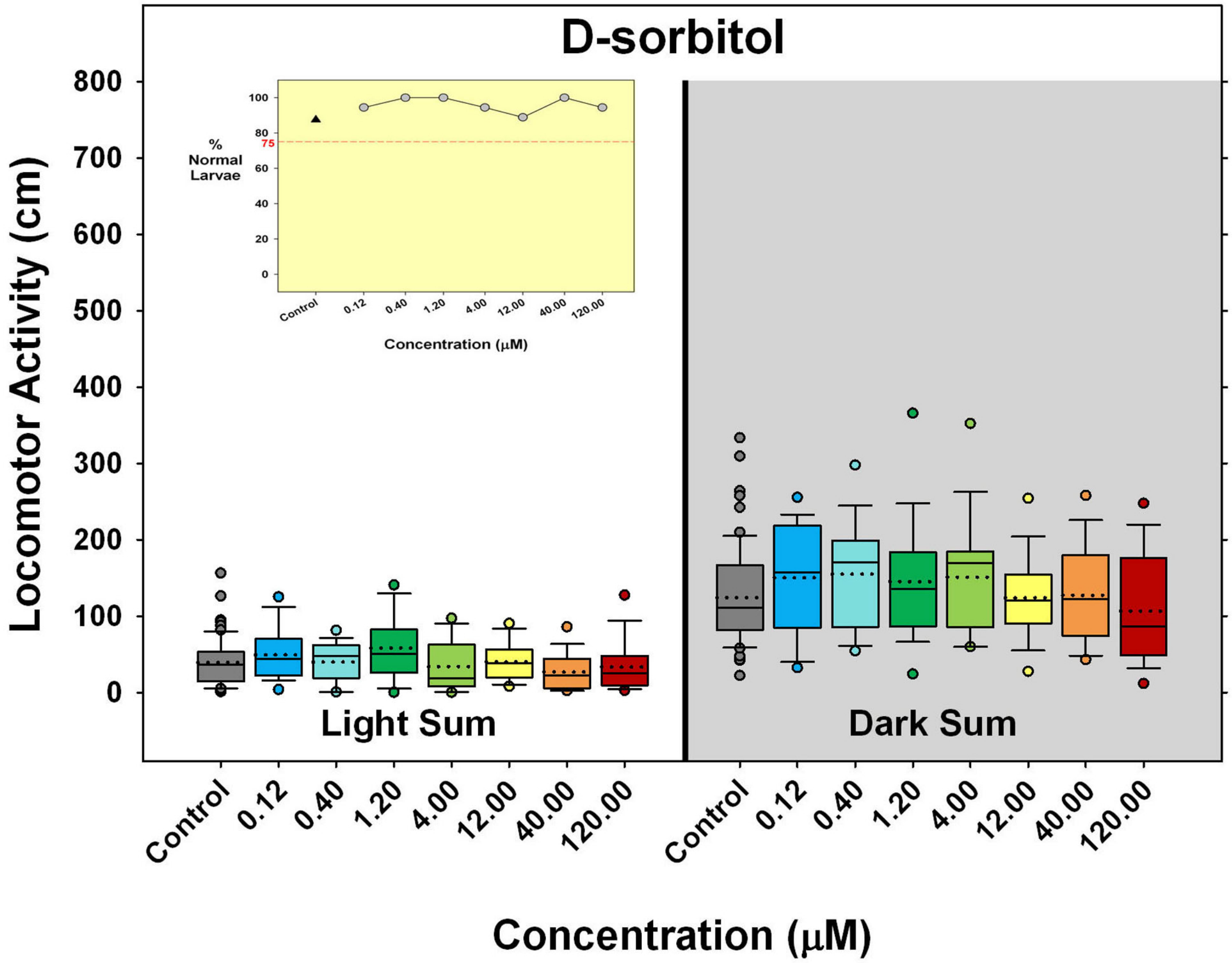
D-sorbitol
Locomotor Activity (cm)
800
700
600
500
400
300
200
100
0
% Normal Larvae
75
Concentration (µM)
Light Sum
Dark Sum
Control
0.12
0.40
1.20
4.00
12.00
40.00
120.00
Concentration (µM)

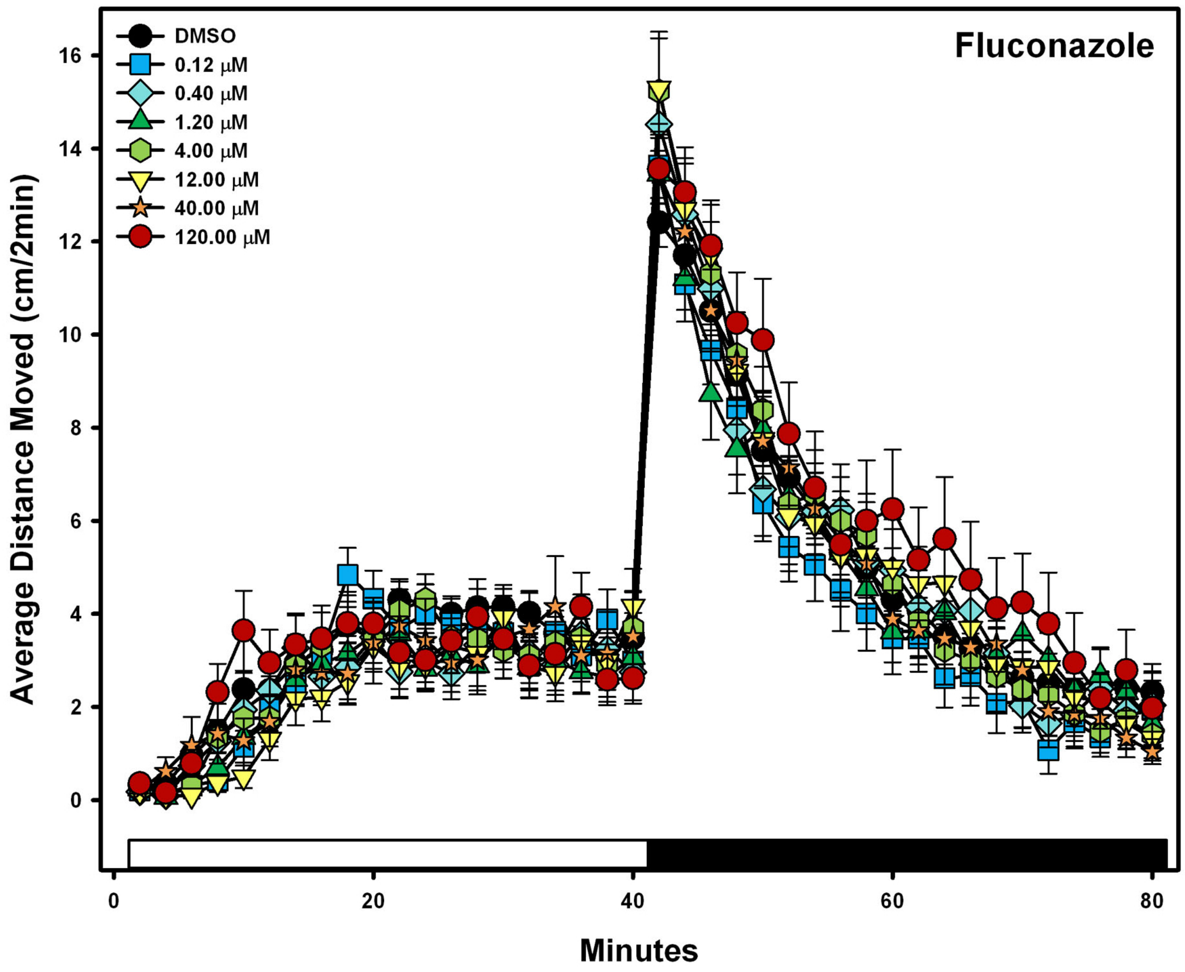
Fluconazole
DMSO
0.12 μM
0.40 μM
1.20 μM
4.00 μM
12.00 μM
40.00 μM
120.00 μM
Average Distance Moved (cm/2min)
Minutes
0
2
4
6
8
10
12
14
16
20
40
60
80

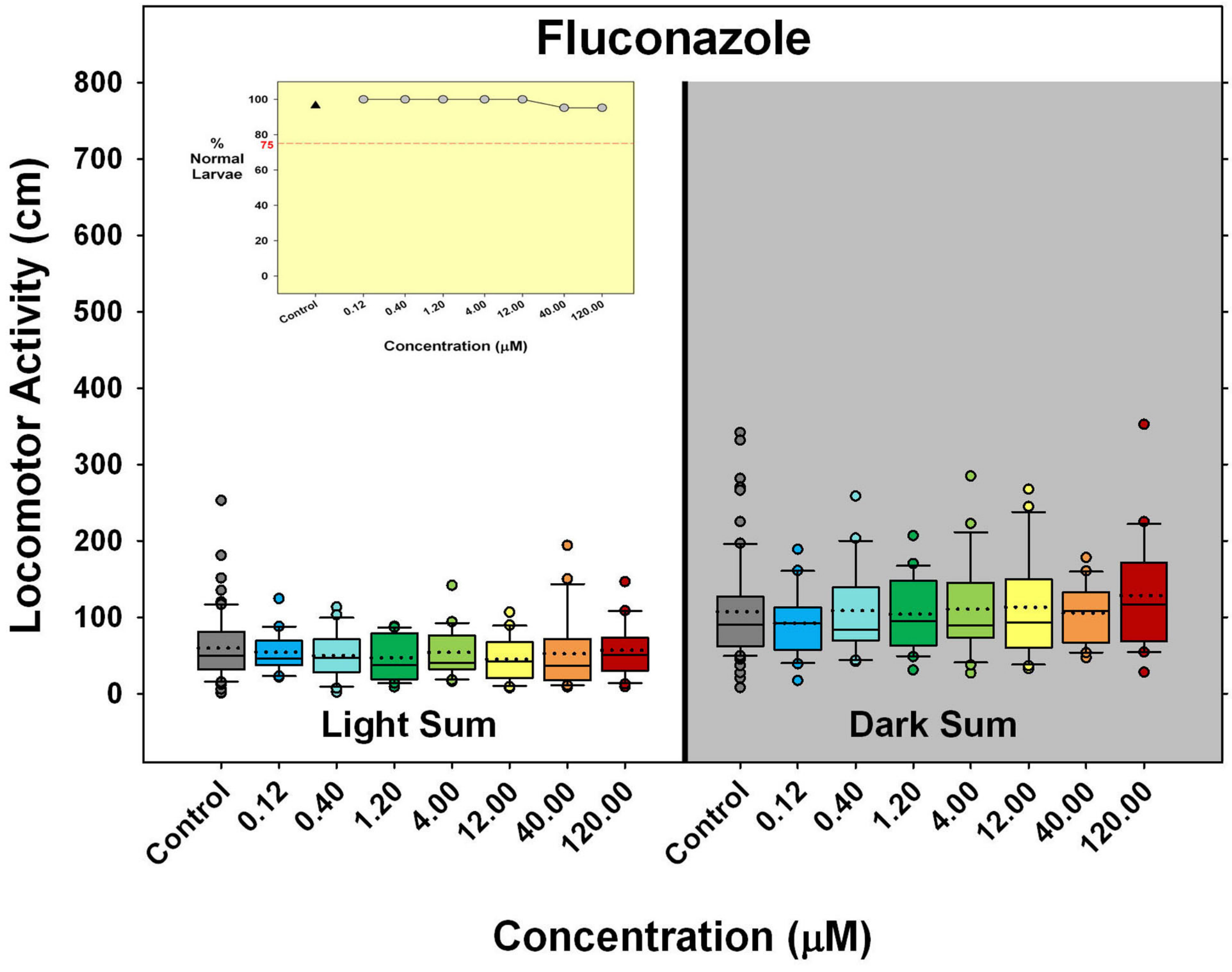
Fluconazole
Locomotor Activity (cm)
800
700
600
500
400
300
200
100
0
% Normal Larvae
75
Control
0.12
0.40
1.20
4.00
12.00
40.00
120.00
Concentration (μM)
Light Sum
Dark Sum
Concentration (μM)

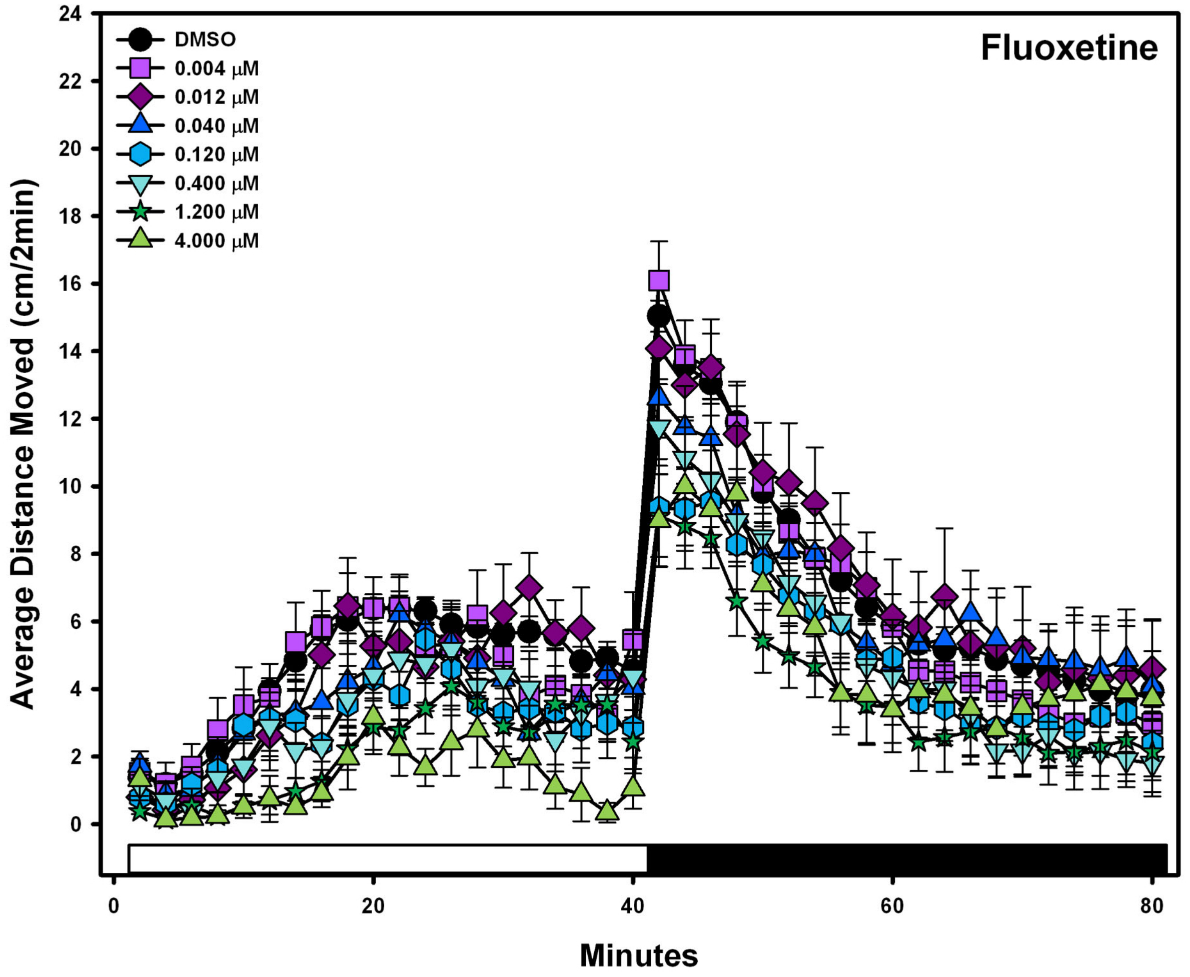

Fluoxetine
DMSO
0.004 μM
0.012 μM
0.040 μM
0.120 μM
0.400 μM
1.200 μM
4.000 μM
Average Distance Moved (cm/2min)
Minutes

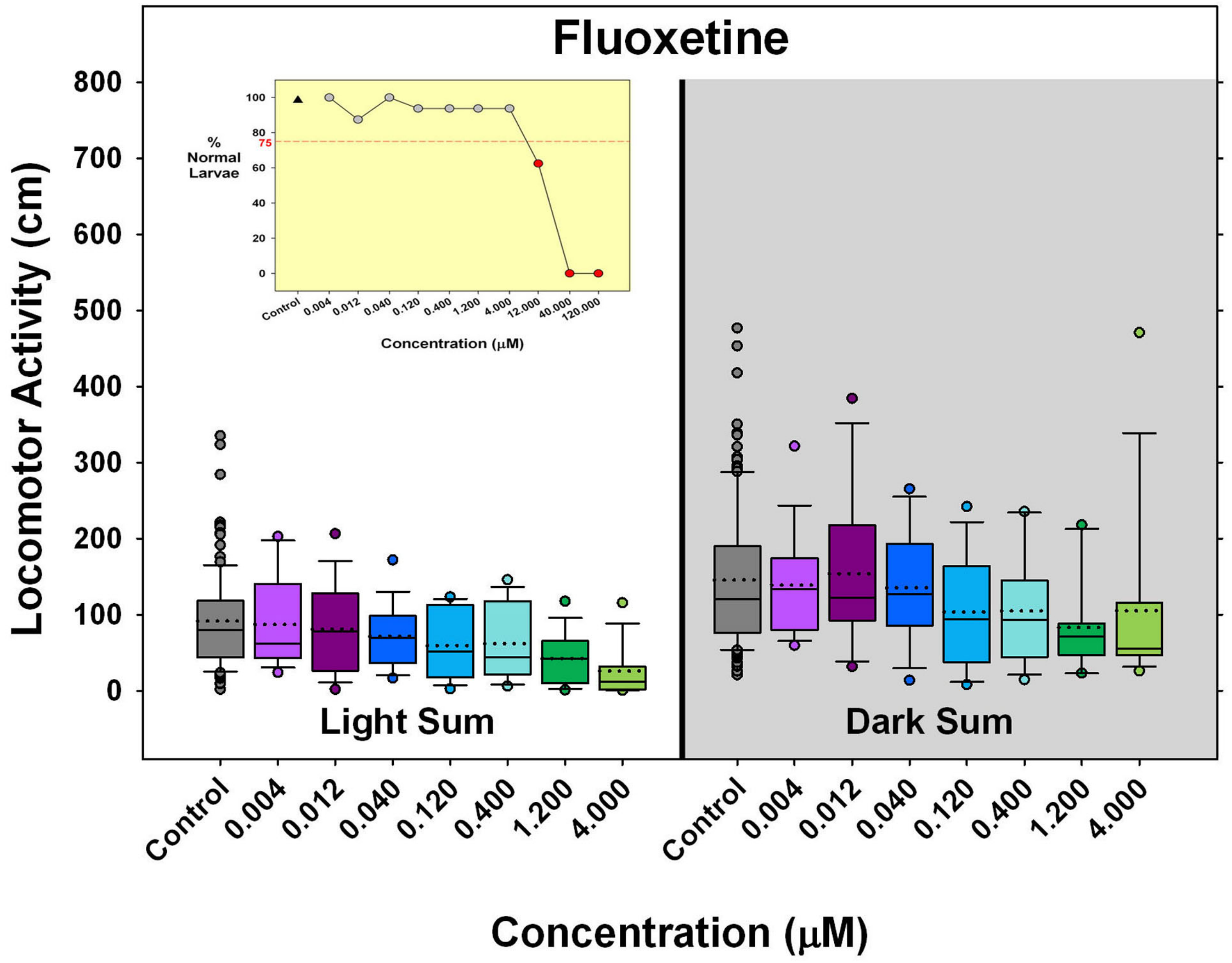

Fluoxetine
Locomotor Activity (cm)
% Normal Larvae
Concentration (μM)
Light Sum
Dark Sum
Control
0.004
0.012
0.040
0.120
0.400
1.200
4.000
Concentration (μM)

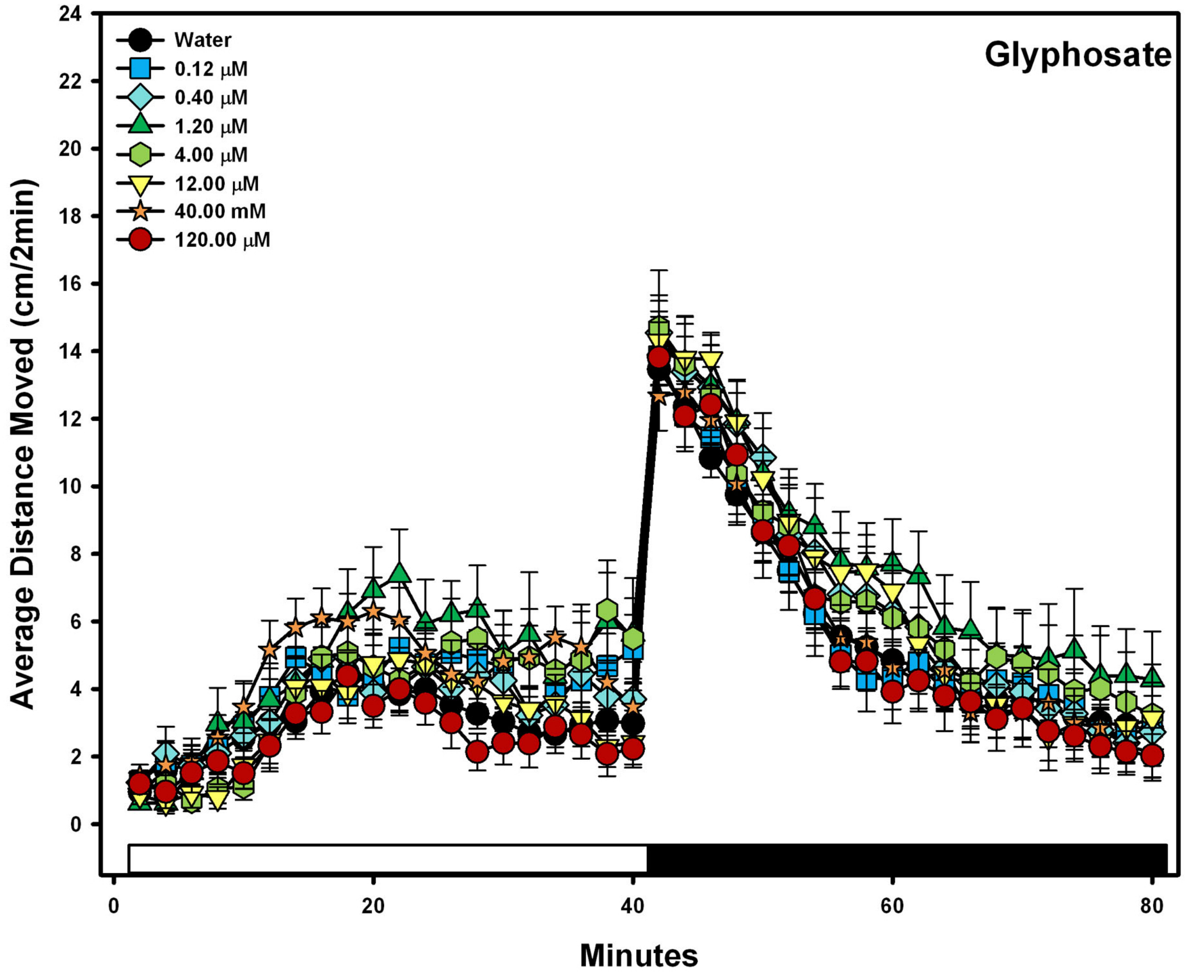
Glyphosate
Water
0.12 µM
0.40 µM
1.20 µM
4.00 µM
12.00 µM
40.00 mM
120.00 µM
Average Distance Moved (cm/2min)
Minutes

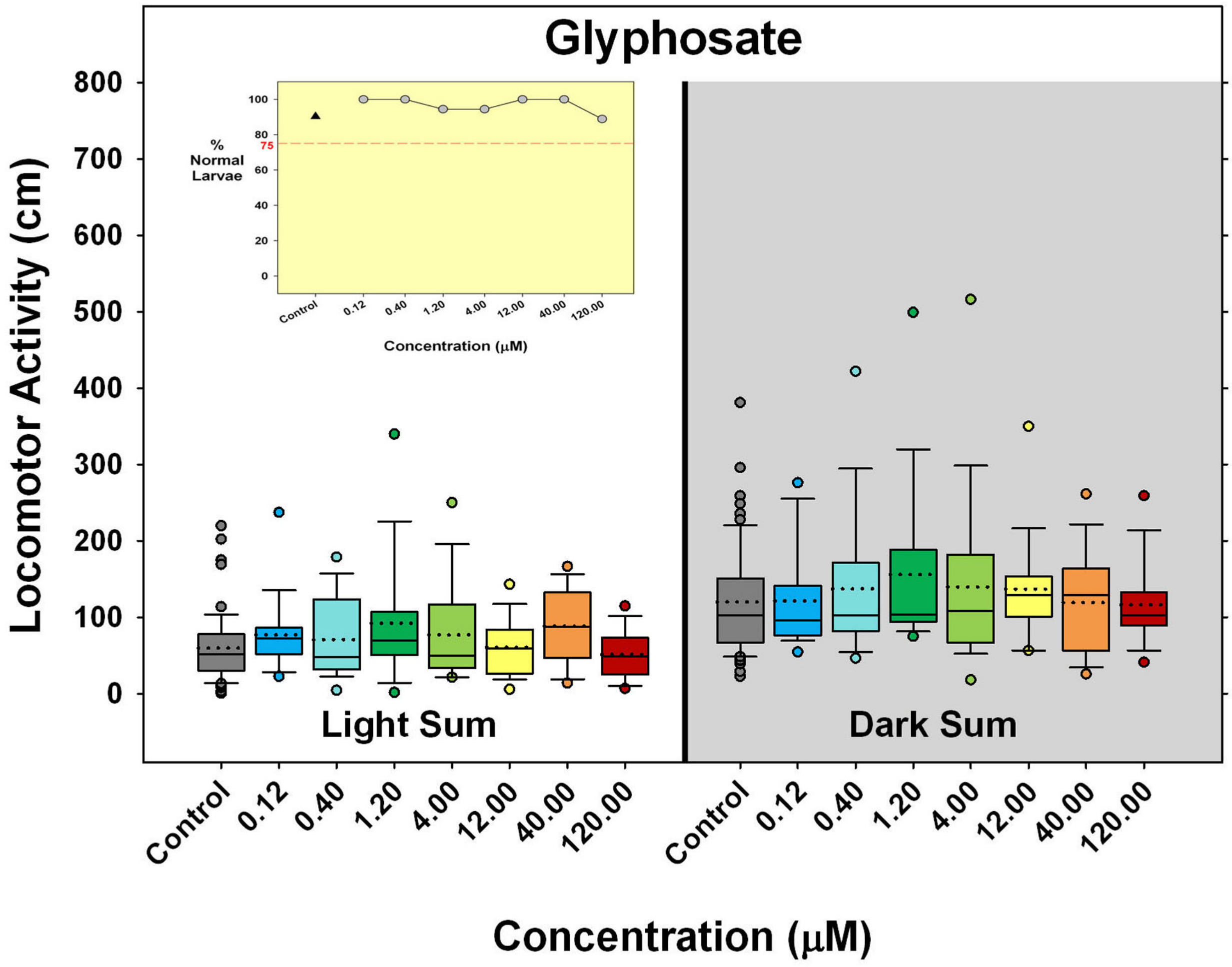
Glyphosate
Locomotor Activity (cm)
% Normal Larvae
75
Concentration (µM)
Control
0.12
0.40
1.20
4.00
12.00
40.00
120.00
Light Sum
Dark Sum
Concentration (µM)

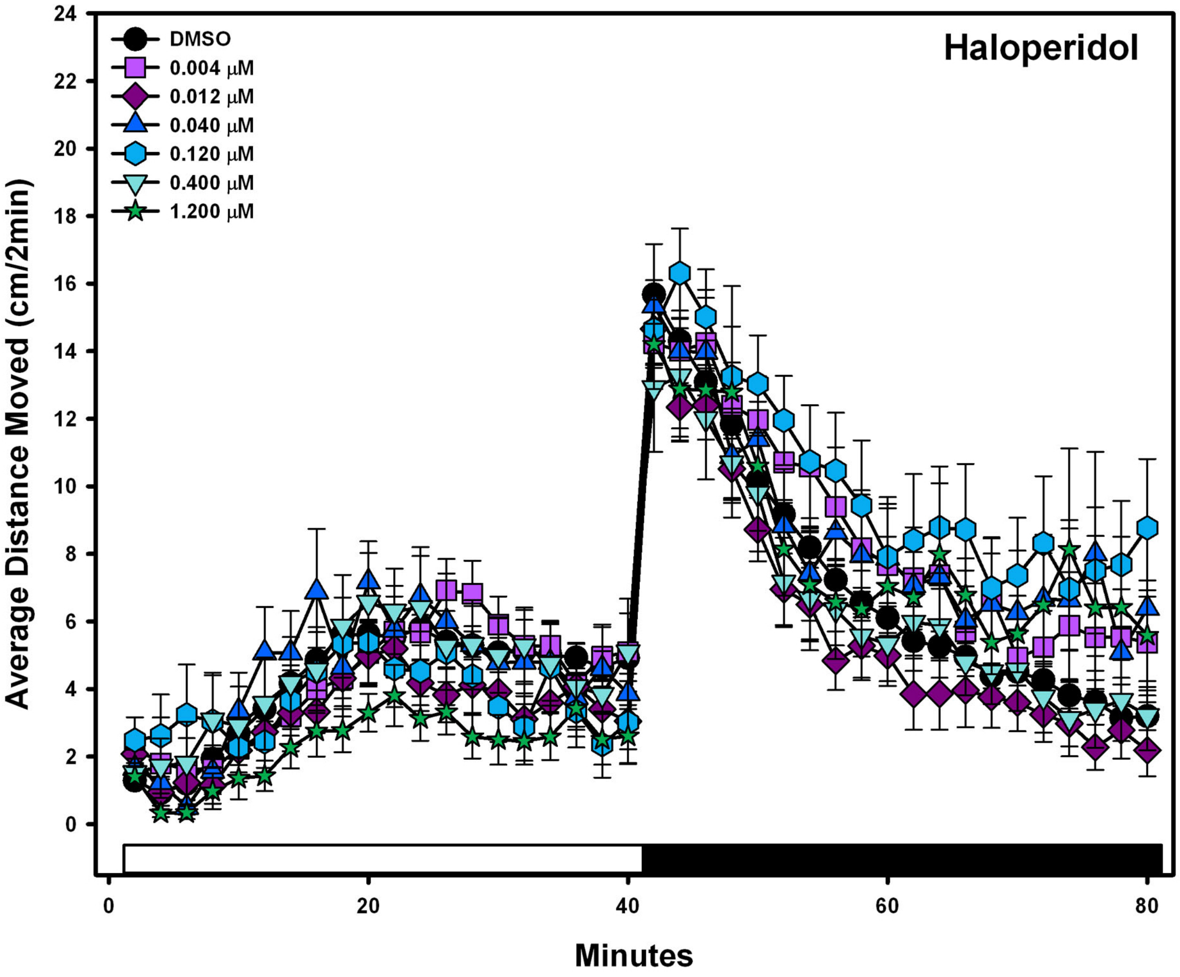

Haloperidol
DMSO
0.004 μM
0.012 μM
0.040 μM
0.120 μM
0.400 μM
1.200 μM
Average Distance Moved (cm/2min)
Minutes

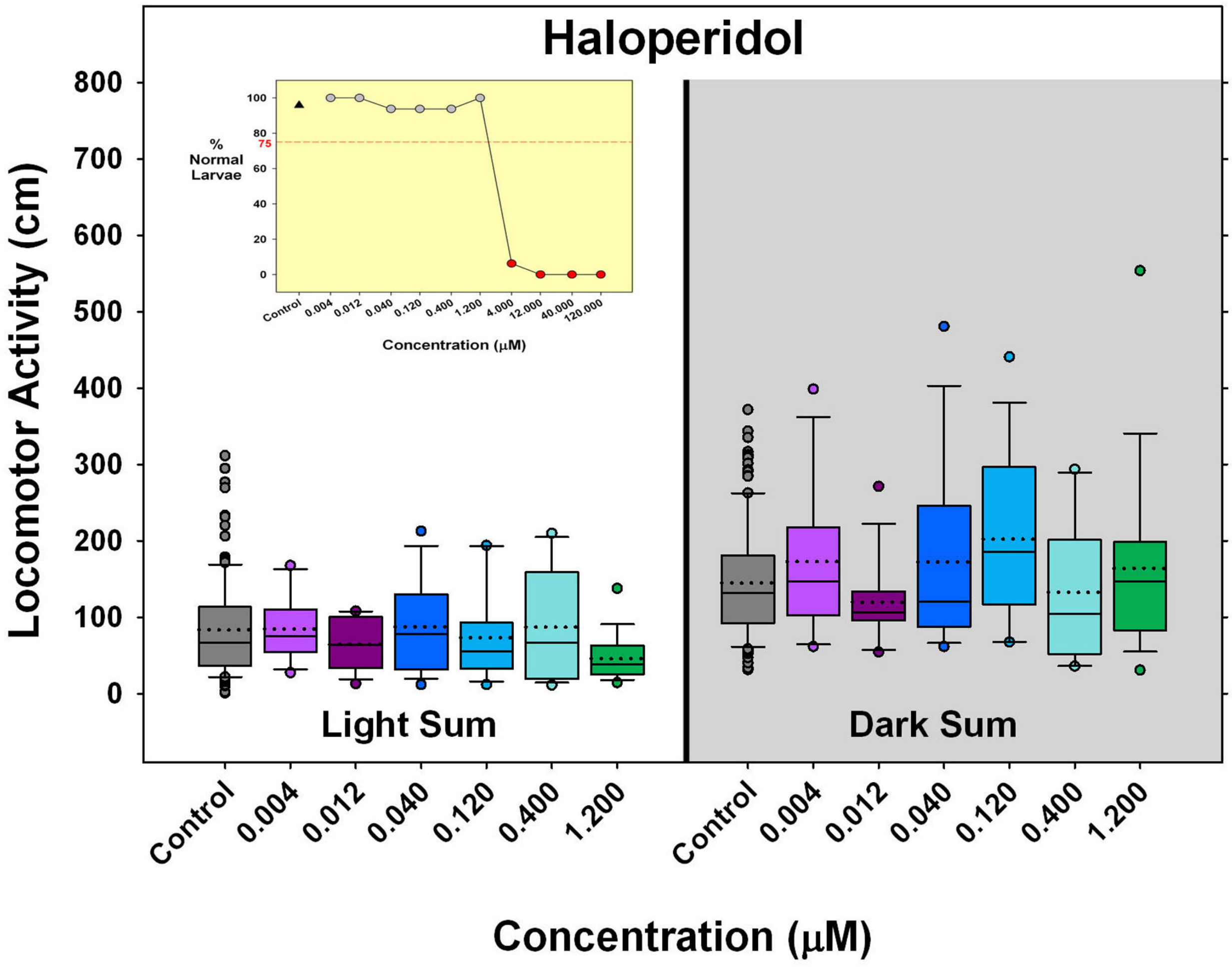

Haloperidol
Locomotor Activity (cm)
% Normal Larvae
Concentration (μM)
Light Sum
Dark Sum
Control
0.004
0.012
0.040
0.120
0.400
1.200
Concentration (μM)

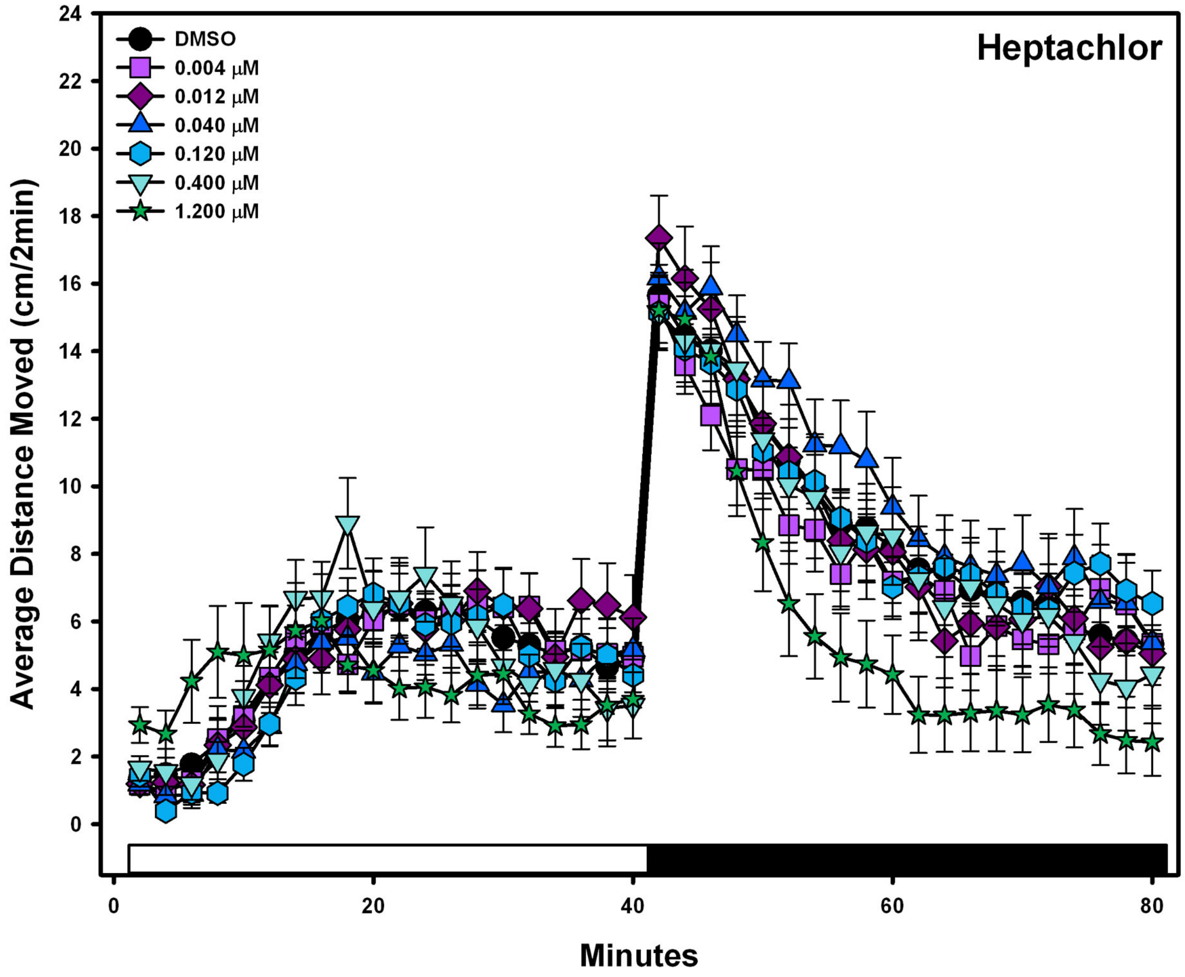

Heptachlor
DMSO
0.004 µM
0.012 µM
0.040 µM
0.120 µM
0.400 µM
1.200 µM
Average Distance Moved (cm/2min)
Minutes

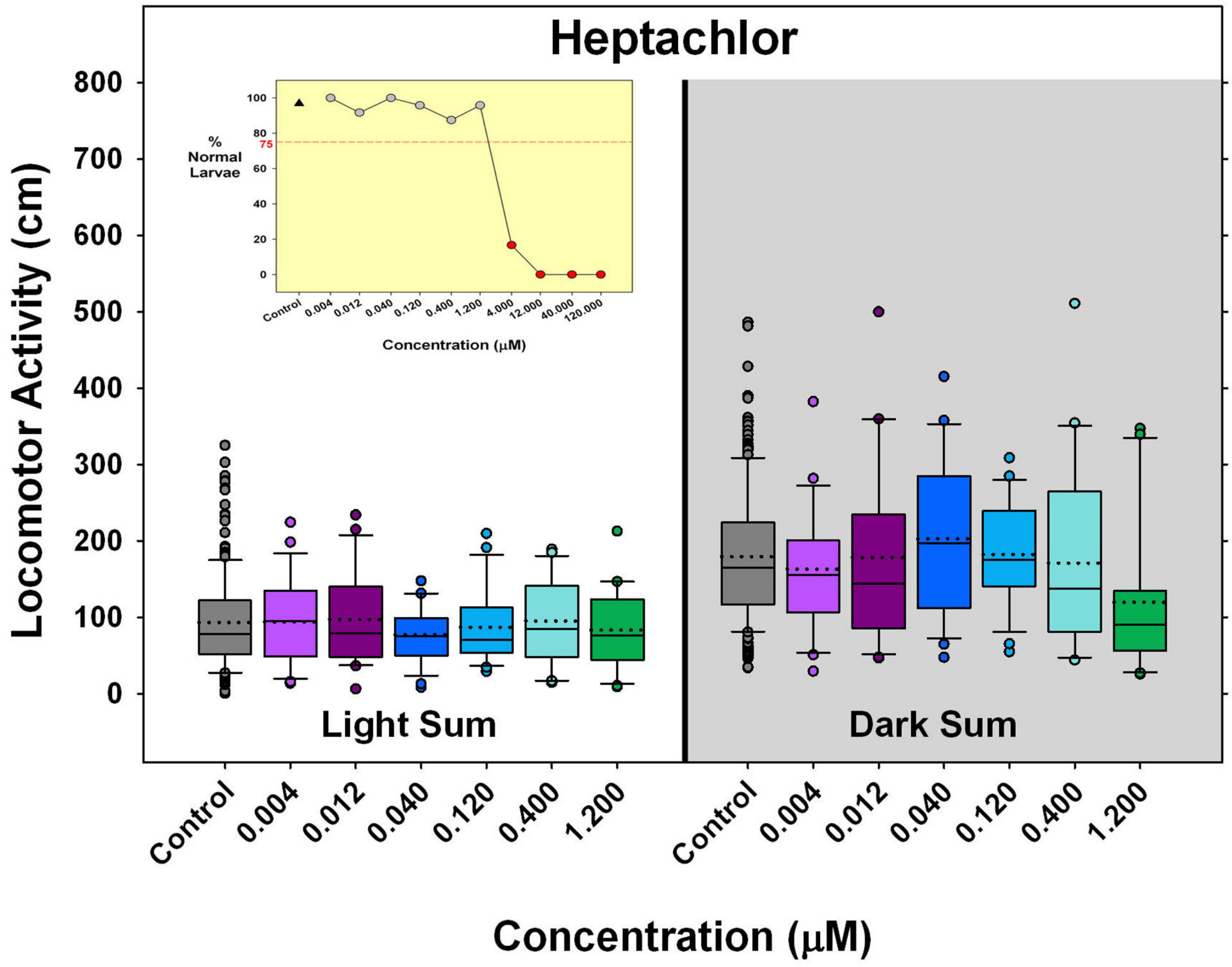

Heptachlor
Locomotor Activity (cm)
% Normal Larvae
Concentration (µM)
Light Sum
Dark Sum
Control
0.004
0.012
0.040
0.120
0.400
1.200
Concentration (µM)

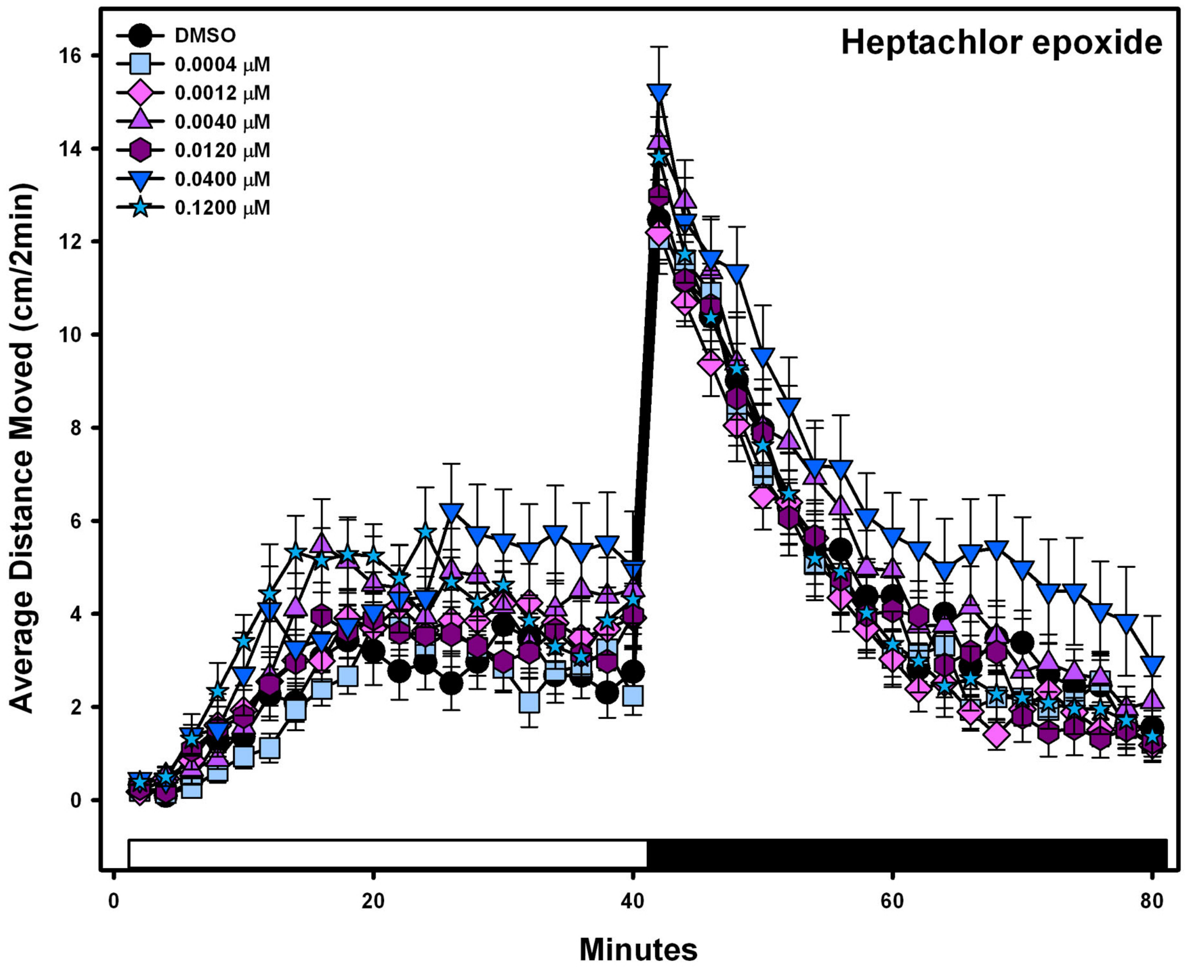

Heptachlor epoxide
DMSO
0.0004 µM
0.0012 µM
0.0040 µM
0.0120 µM
0.0400 µM
0.1200 µM
Average Distance Moved (cm/2min)
Minutes

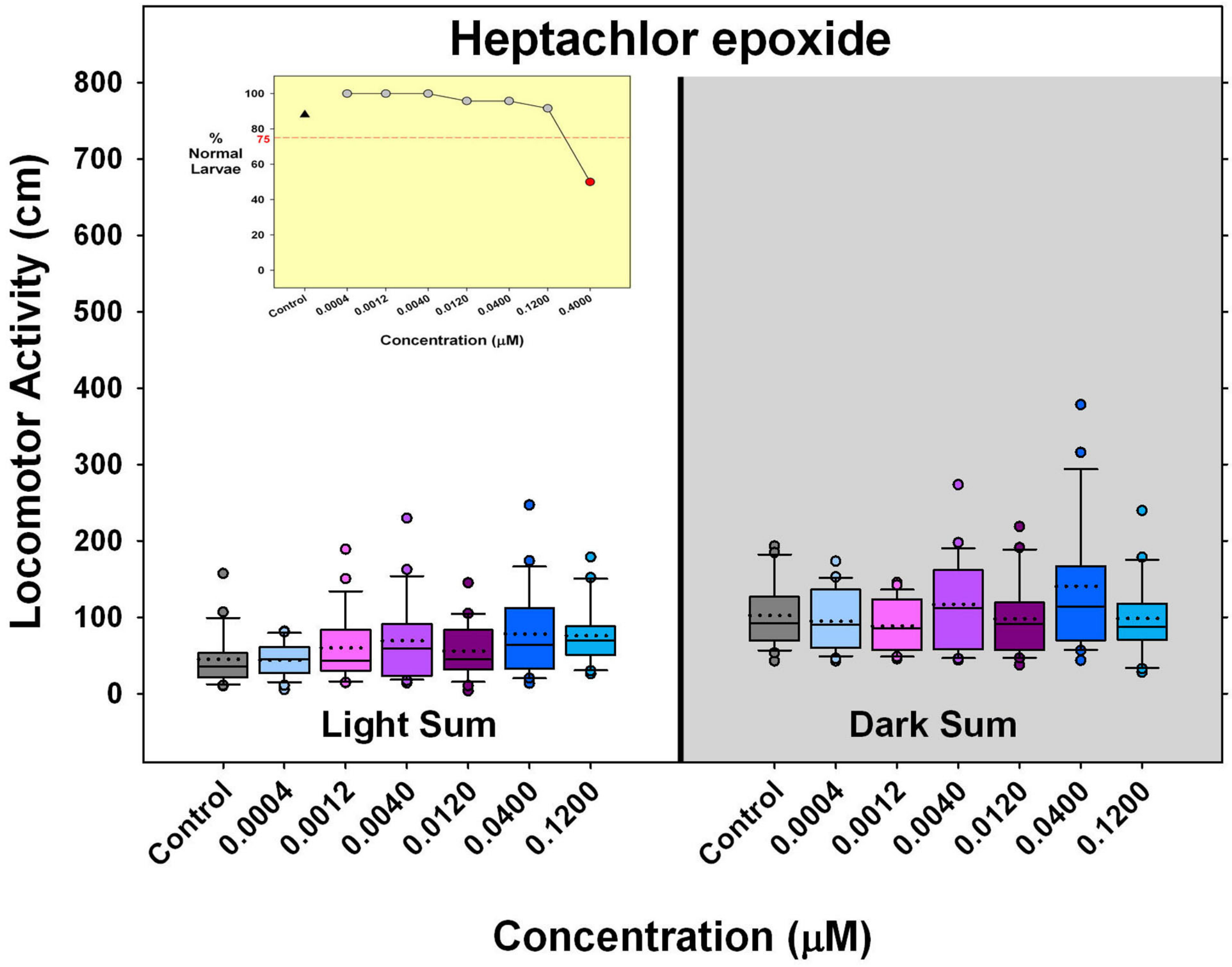

Heptachlor epoxide
% Normal Larvae
75
Concentration (µM)
Locomotor Activity (cm)
Light Sum
Dark Sum
Control
0.0004
0.0012
0.0040
0.0120
0.0400
0.1200
Concentration (µM)

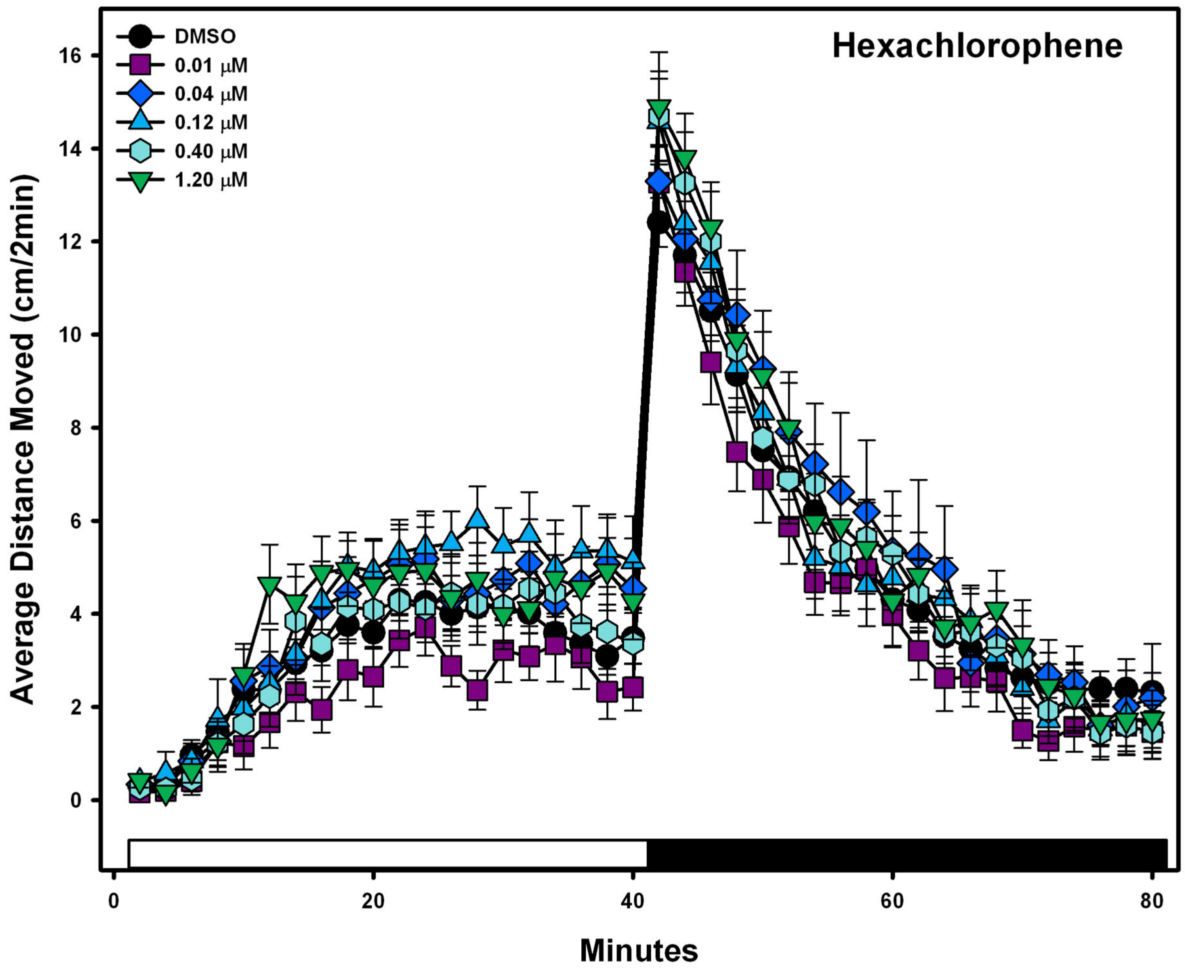

Hexachlorophene
DMSO
0.01 μM
0.04 μM
0.12 μM
0.40 μM
1.20 μM
Average Distance Moved (cm/2min)
Minutes
0
2
4
6
8
10
12
14
16
0
20
40
60
80

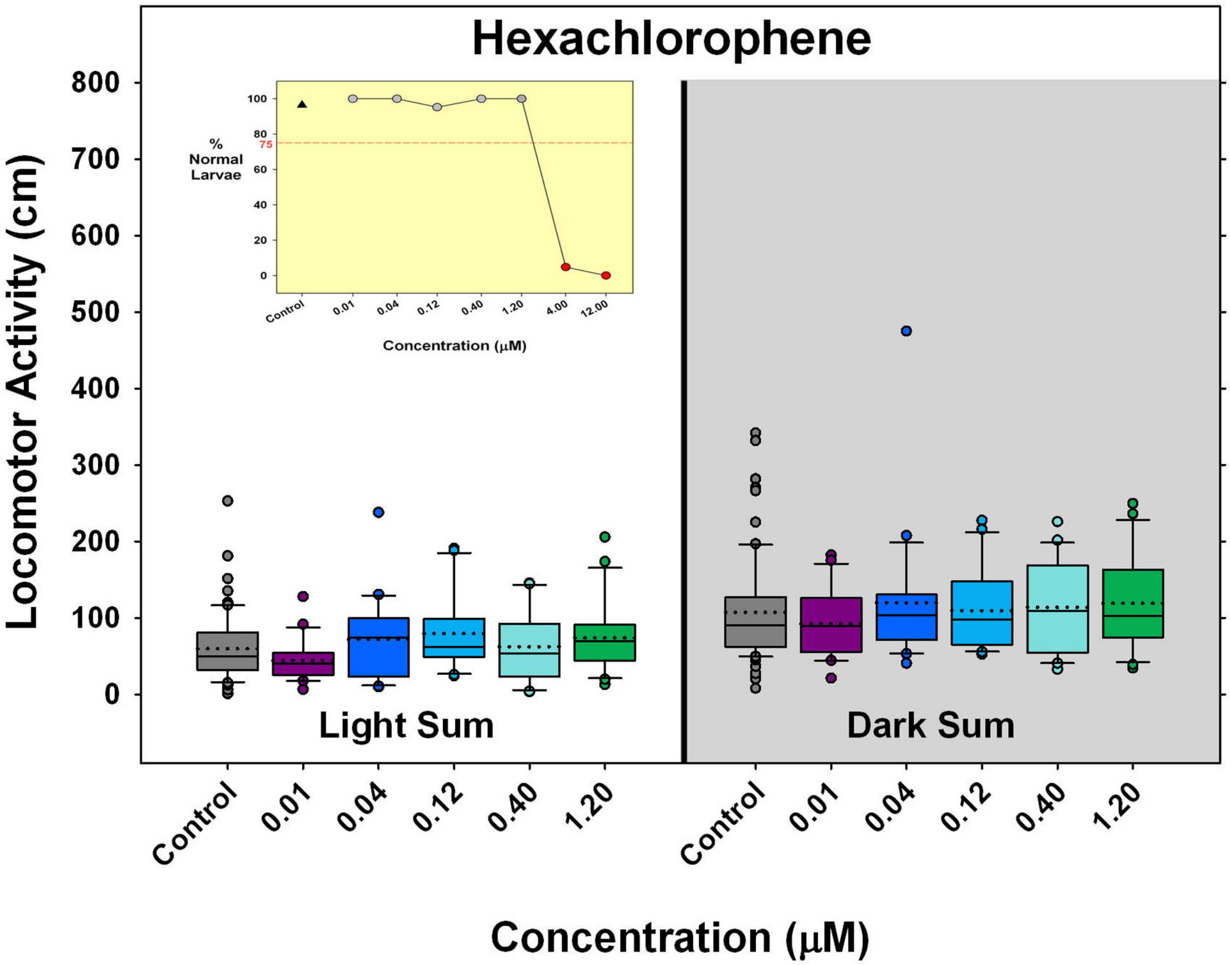

Hexachlorophene
Locomotor Activity (cm)
800
700
600
500
400
300
200
100
0
% Normal Larvae
Concentration (μM)
Control
0.01
0.04
0.12
0.40
1.20
4.00
12.00
Light Sum
Dark Sum
Control
0.01
0.04
0.12
0.40
1.20
Control
0.01
0.04
0.12
0.40
1.20
Concentration (μM)

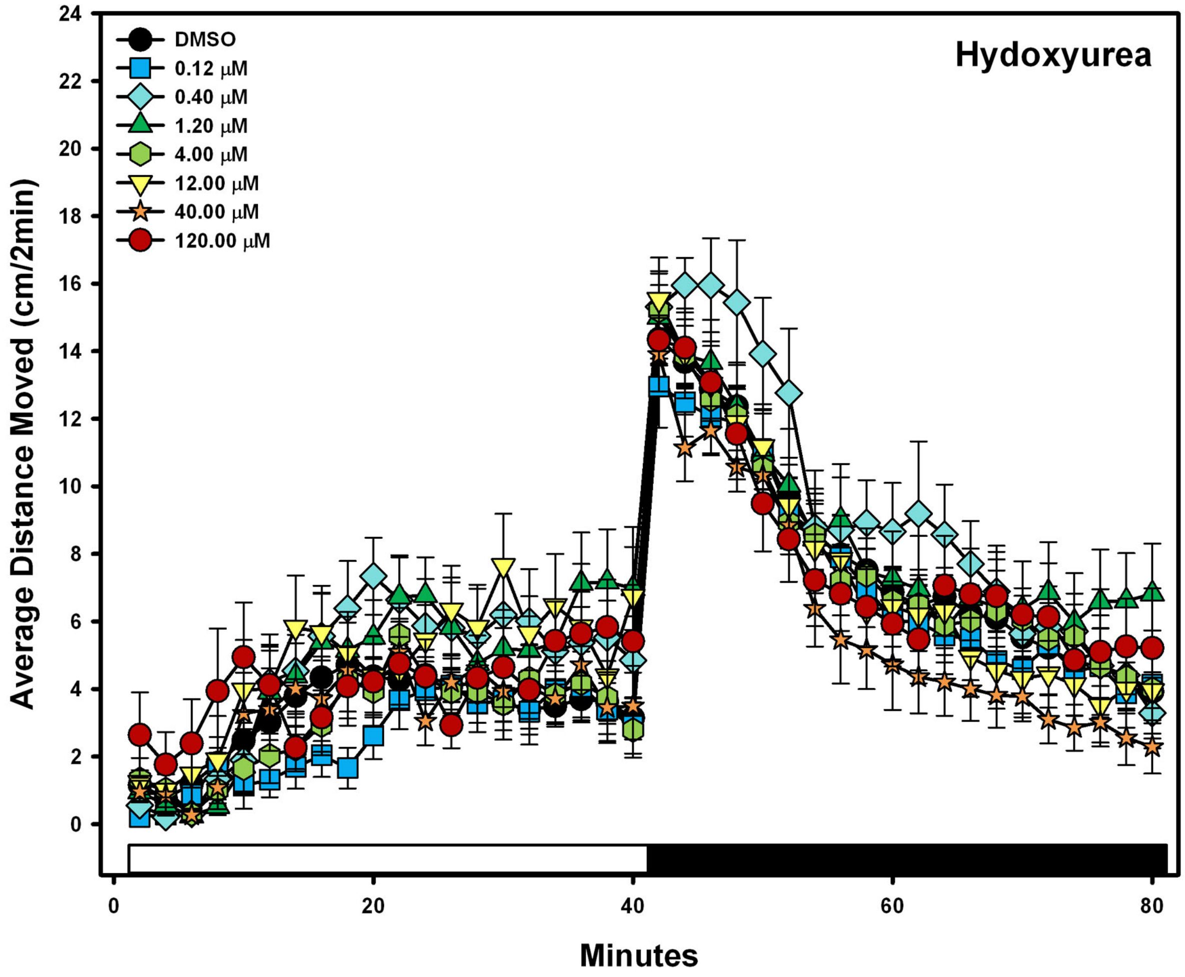

Hydoxyurea
DMSO
0.12 µM
0.40 µM
1.20 µM
4.00 µM
12.00 µM
40.00 µM
120.00 µM
Average Distance Moved (cm/2min)
Minutes

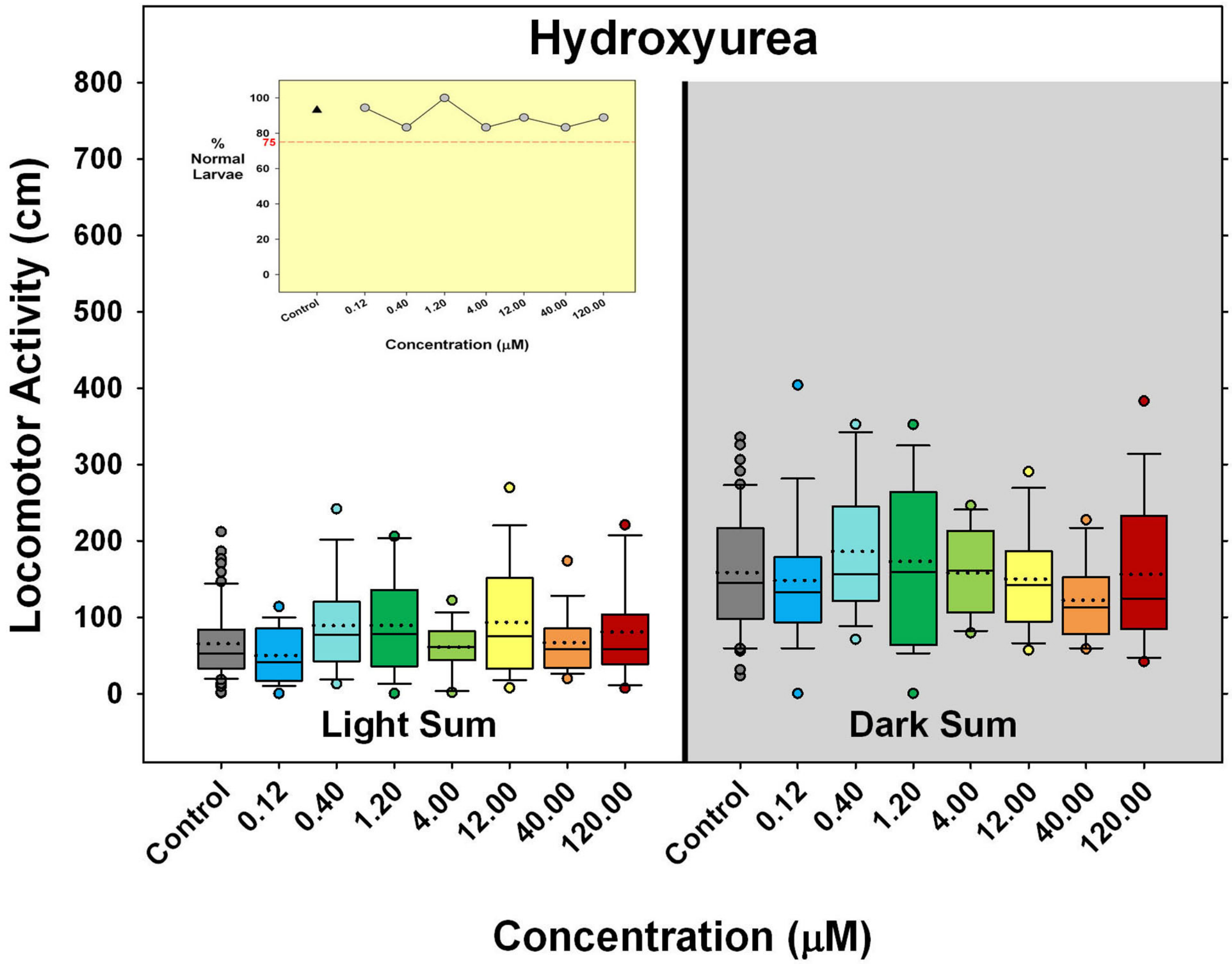

Hydroxyurea
Locomotor Activity (cm)
% Normal Larvae
75
Concentration (µM)
Light Sum
Dark Sum
Control
0.12
0.40
1.20
4.00
12.00
40.00
120.00
Concentration (µM)

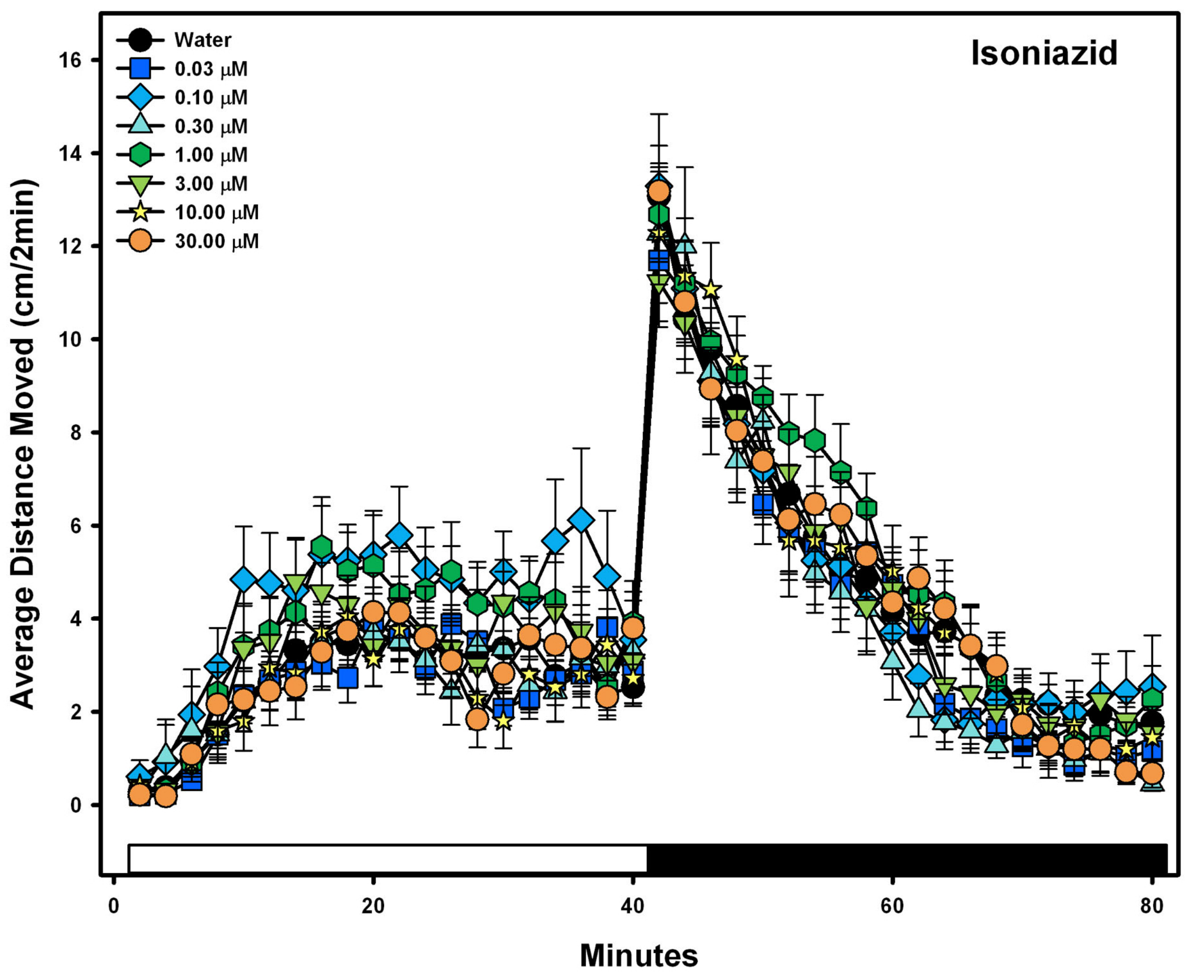

Isoniazid
Water
0.03 µM
0.10 µM
0.30 µM
1.00 µM
3.00 µM
10.00 µM
30.00 µM
Average Distance Moved (cm/2min)
Minutes
0
2
4
6
8
10
12
14
16
0
20
40
60
80

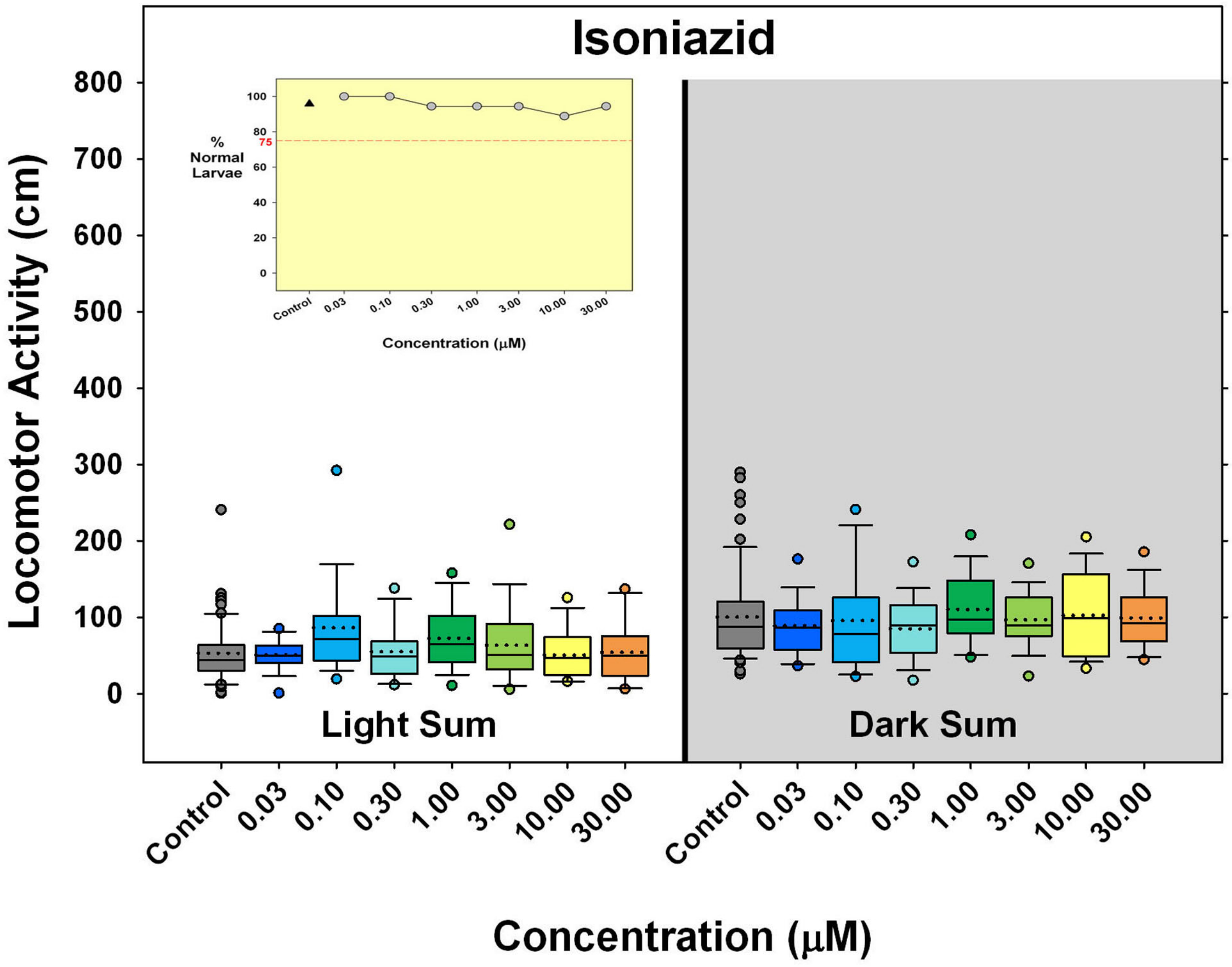

Isoniazid
Locomotor Activity (cm)
800
700
600
500
400
300
200
100
0
% Normal Larvae
75
Concentration (µM)
Light Sum
Dark Sum
Control
0.03
0.10
0.30
1.00
3.00
10.00
30.00
Concentration (µM)

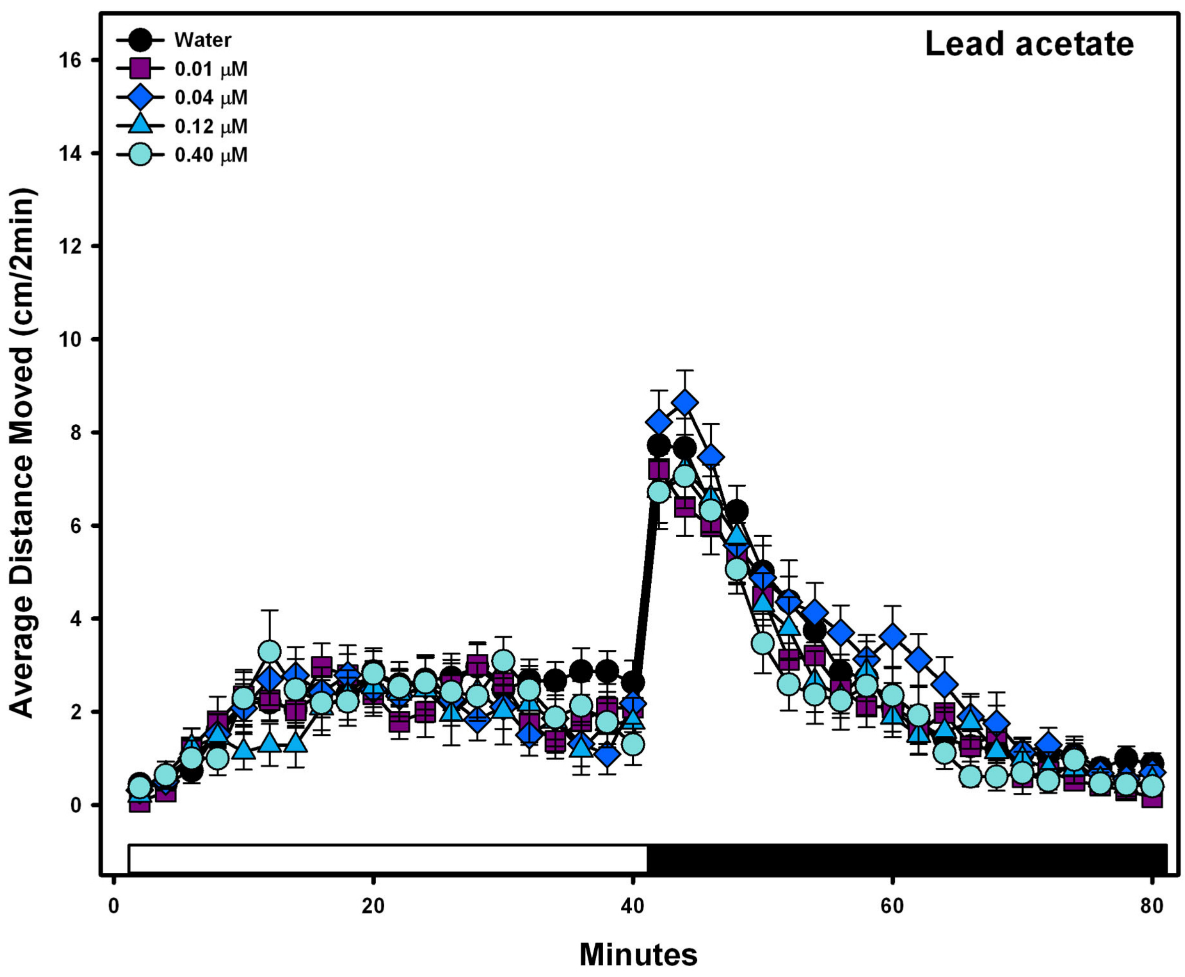
Lead acetate
Water
0.01 μM
0.04 μM
0.12 μM
0.40 μM
Average Distance Moved (cm/2min)
16
14
12
10
8
6
4
2
0
0
20
40
60
80
Minutes

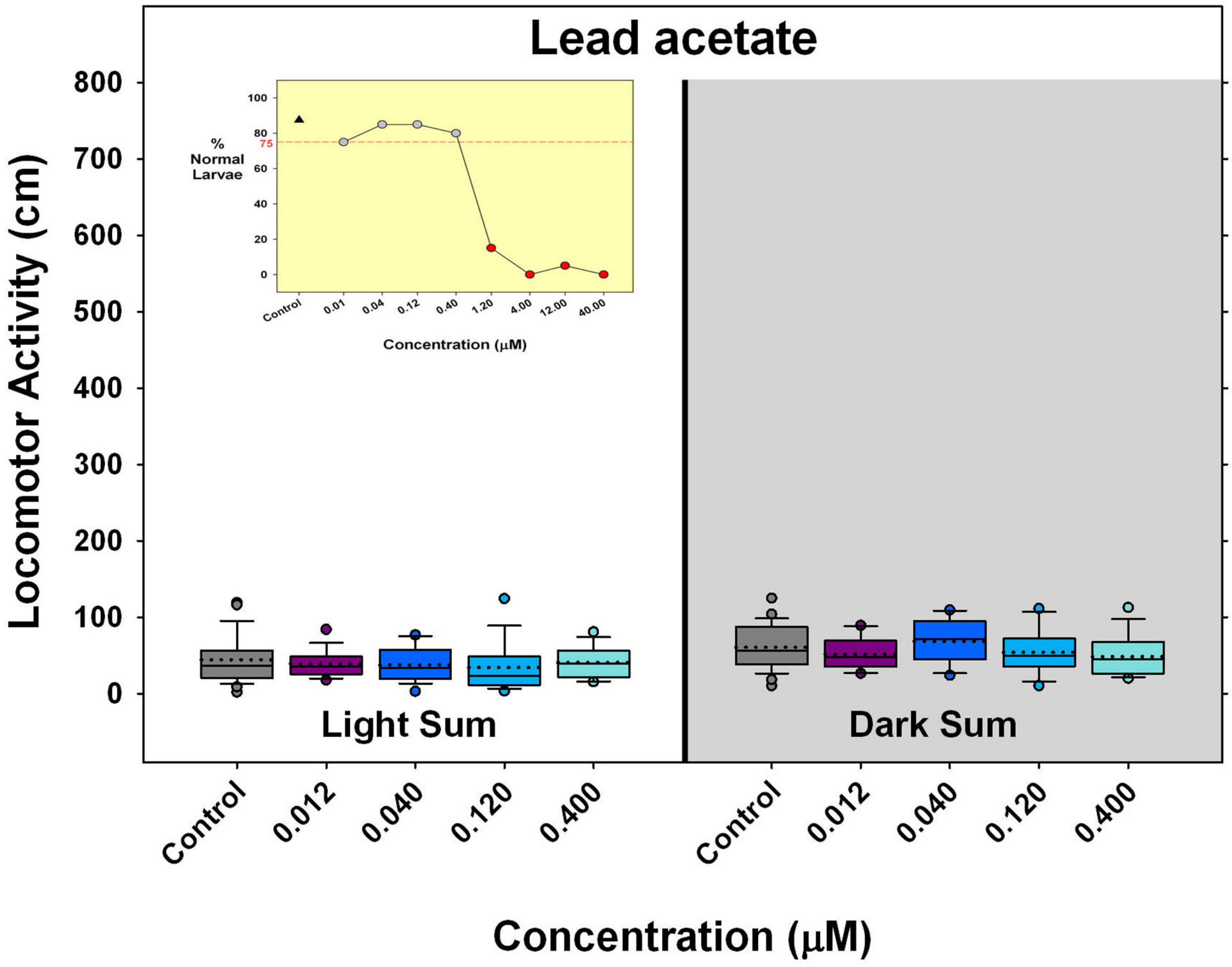
Lead acetate
Locomotor Activity (cm)
800
700
600
500
400
300
200
100
0
% Normal Larvae
100
80
75
60
40
20
0
Control
0.01
0.04
0.12
0.40
1.20
4.00
12.00
40.00
Concentration (μM)
Light Sum
Dark Sum
Control
0.012
0.040
0.120
0.400
Control
0.012
0.040
0.120
0.400
Concentration (μM)

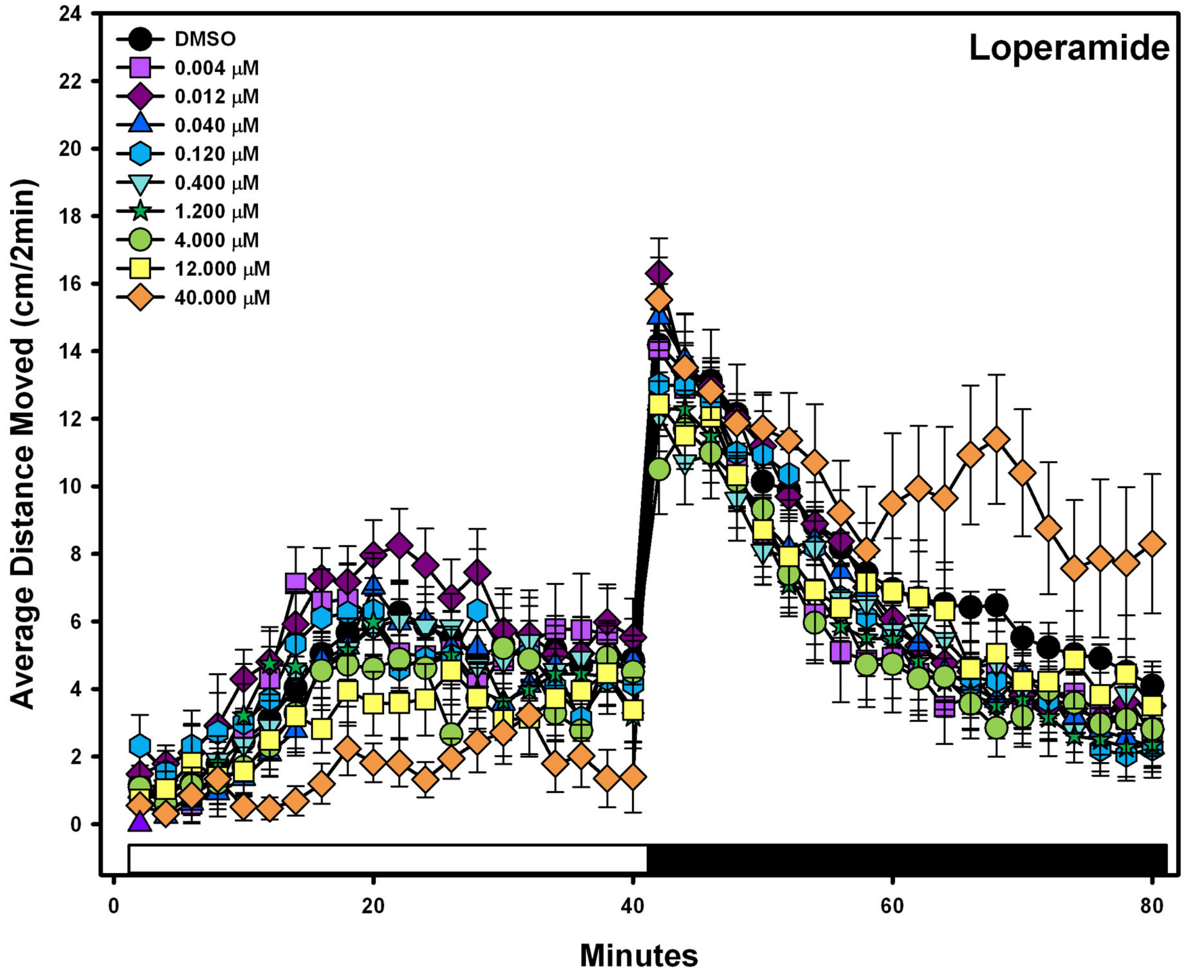
Loperamide
DMSO
0.004 μM
0.012 μM
0.040 μM
0.120 μM
0.400 μM
1.200 μM
4.000 μM
12.000 μM
40.000 μM
Average Distance Moved (cm/2min)
Minutes

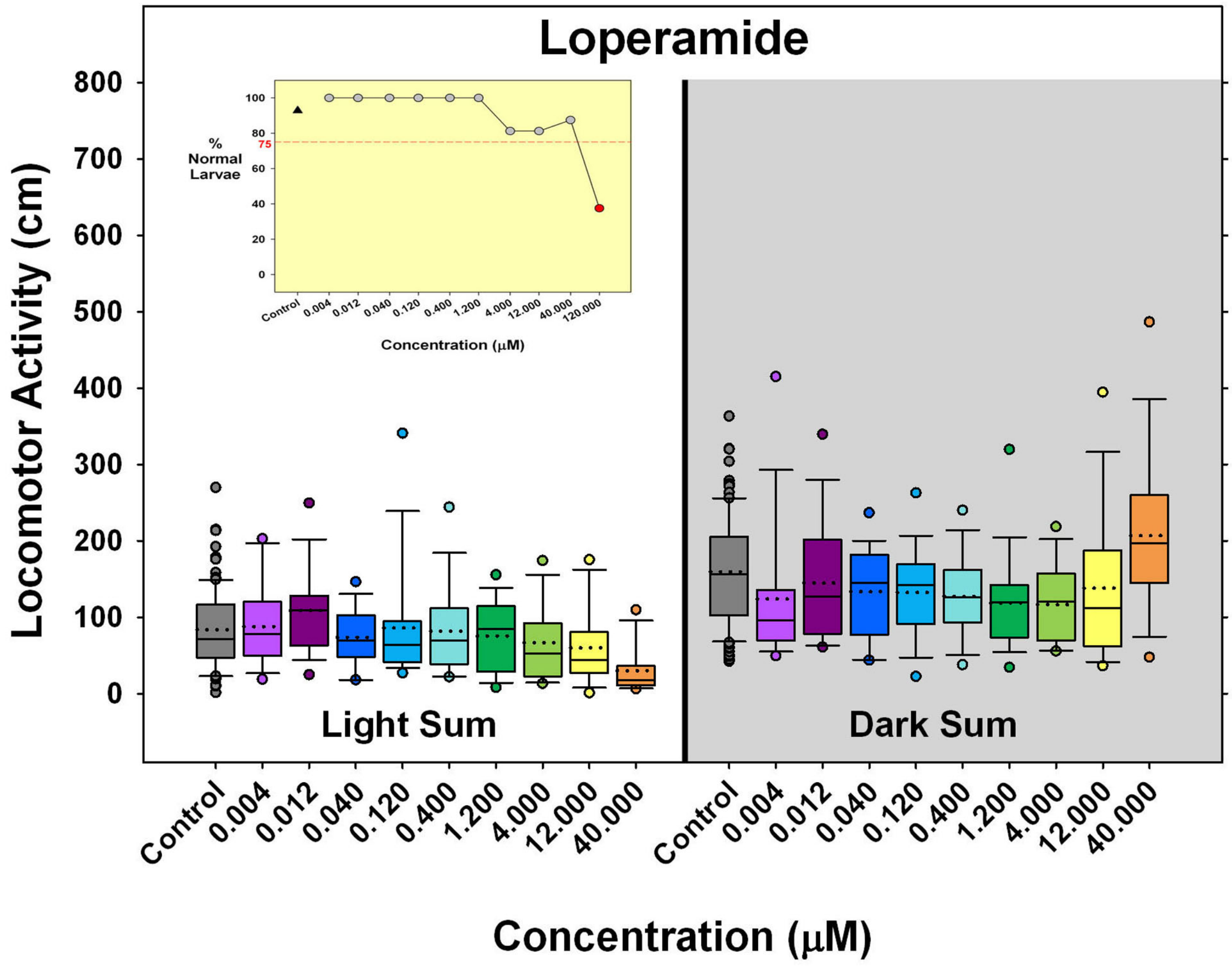
Loperamide
Locomotor Activity (cm)
% Normal Larvae
Concentration (μM)
Light Sum
Dark Sum
Control
0.004
0.012
0.040
0.120
0.400
1.200
4.000
12.000
40.000
120.000

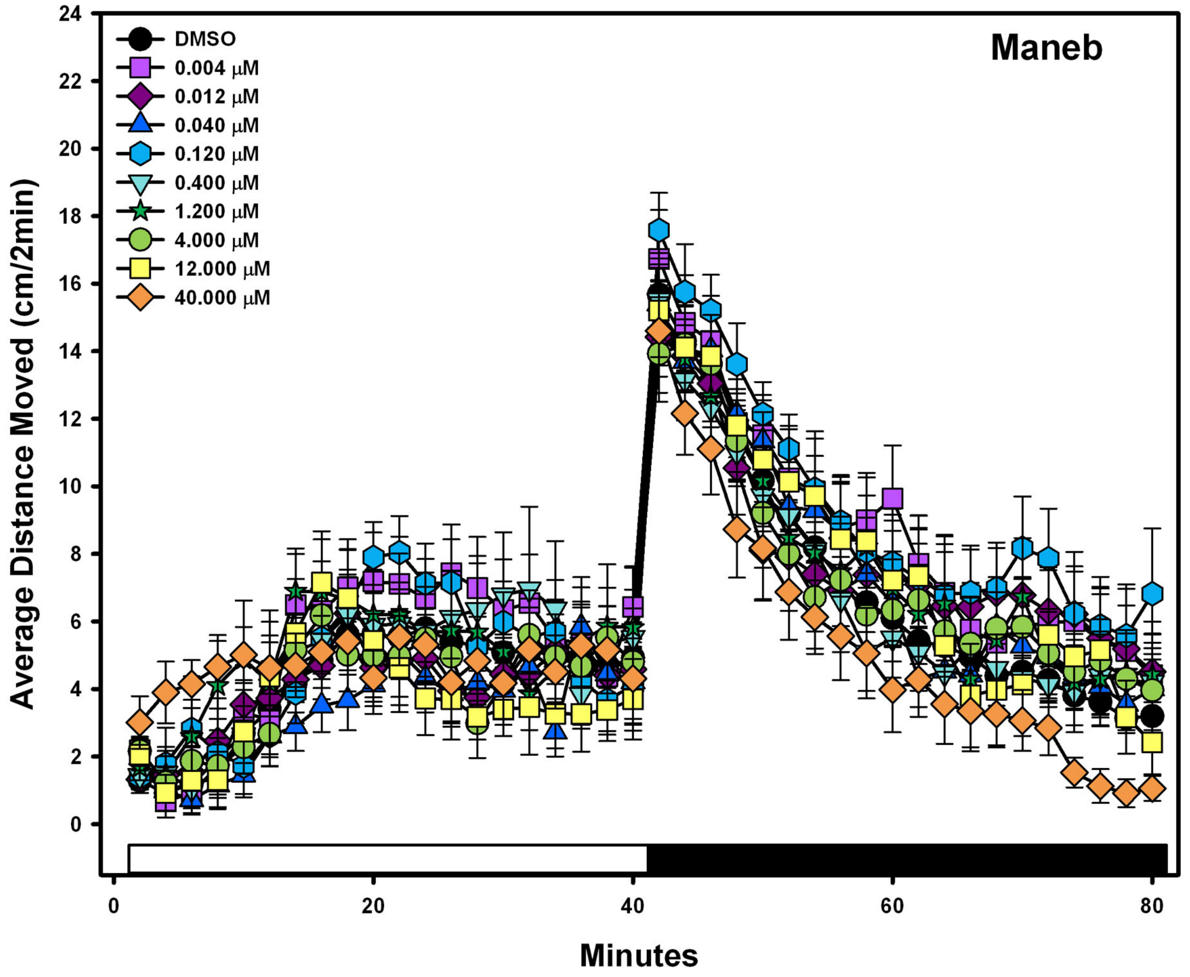

Maneb
DMSO
0.004 μM
0.012 μM
0.040 μM
0.120 μM
0.400 μM
1.200 μM
4.000 μM
12.000 μM
40.000 μM
Average Distance Moved (cm/2min)
Minutes

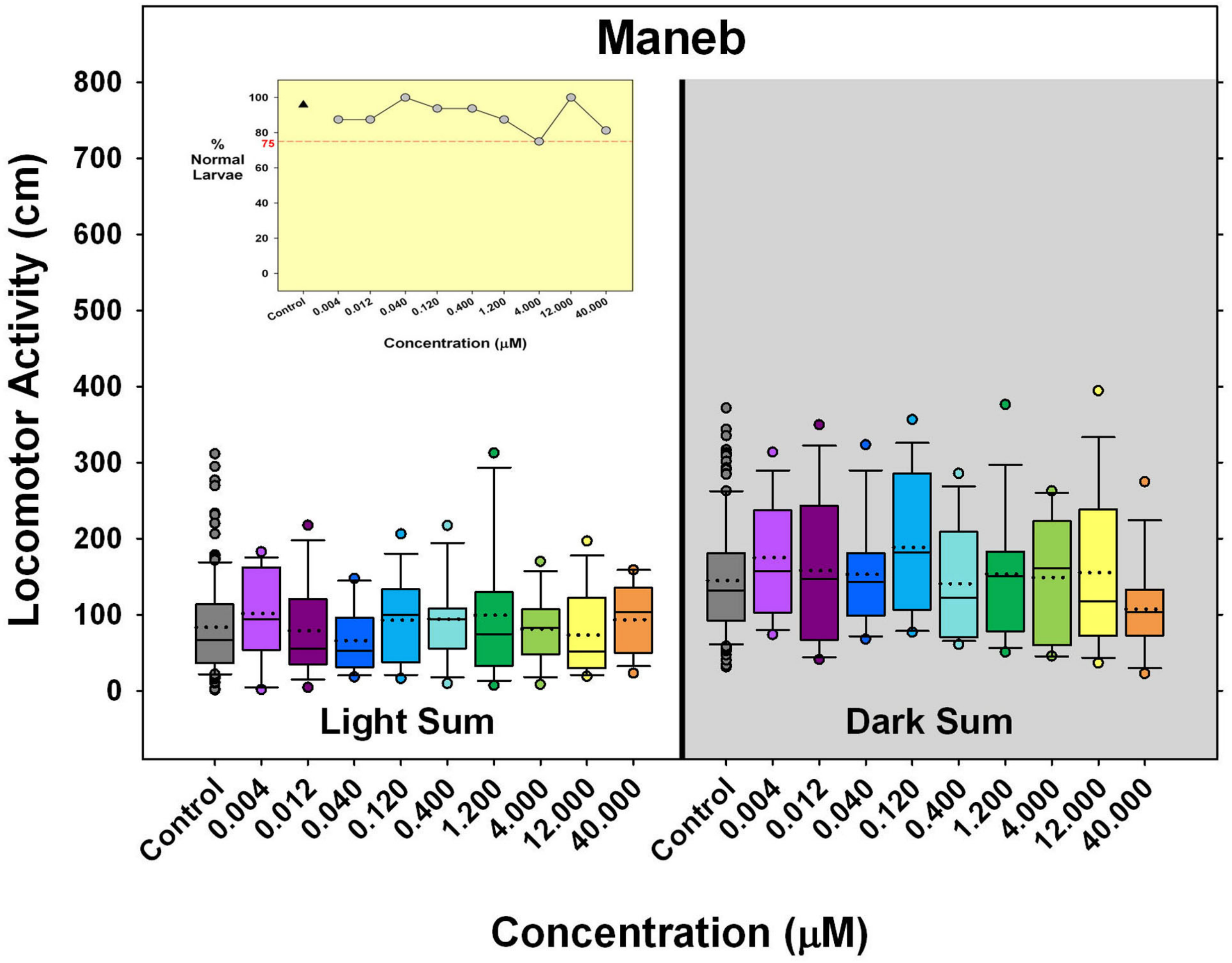

Maneb
Locomotor Activity (cm)
% Normal Larvae
75
Concentration (μM)
Light Sum
Dark Sum
Control
0.004
0.012
0.040
0.120
0.400
1.200
4.000
12.000
40.000
Concentration (μM)

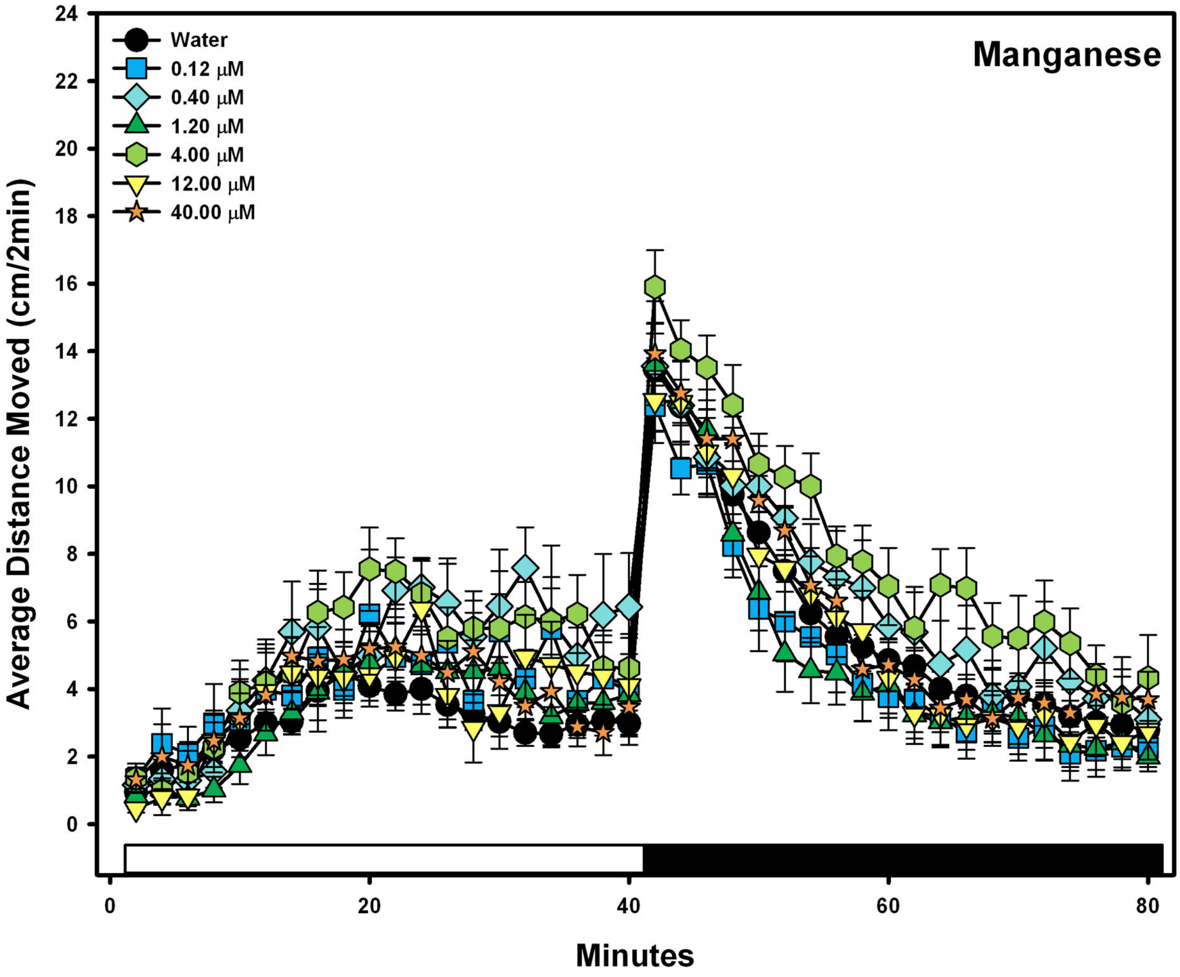

Manganese
Water
0.12 µM
0.40 µM
1.20 µM
4.00 µM
12.00 µM
40.00 µM
Average Distance Moved (cm/2min)
Minutes

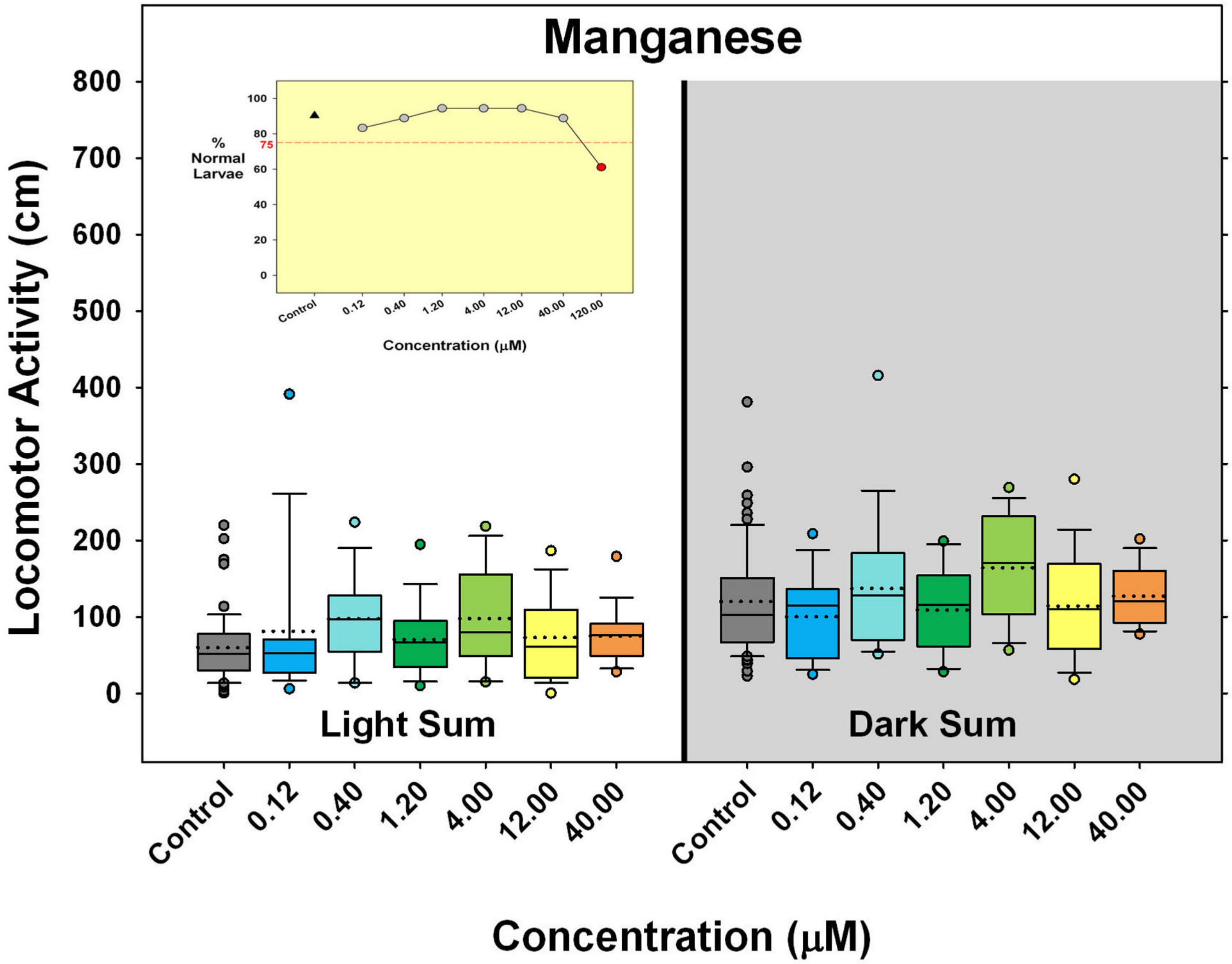

Manganese
Locomotor Activity (cm)
Light Sum
Dark Sum
Control
0.12
0.40
1.20
4.00
12.00
40.00
120.00
% Normal Larvae
75
Concentration (µM)

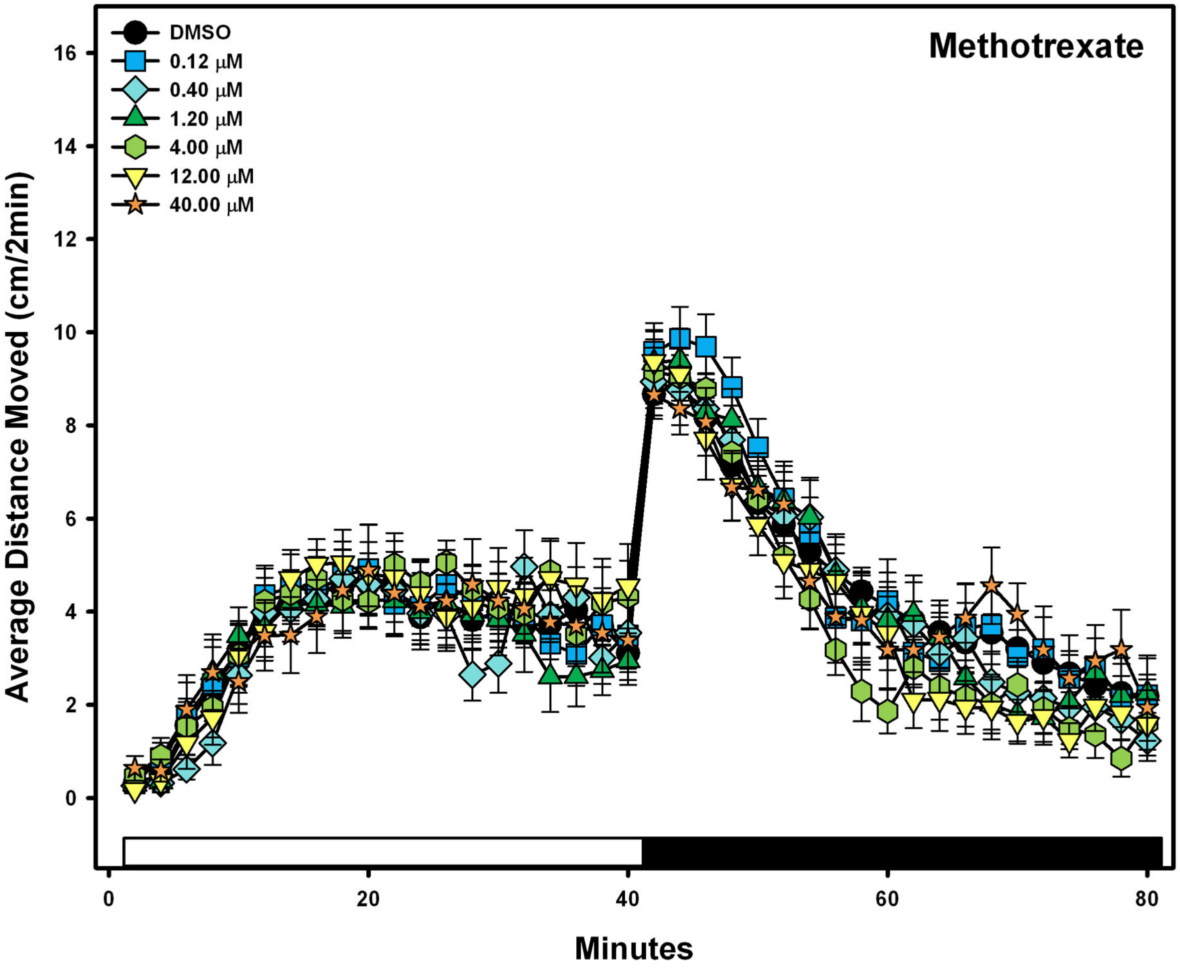
Methotrexate
DMSO
0.12 μM
0.40 μM
1.20 μM
4.00 μM
12.00 μM
40.00 μM
Average Distance Moved (cm/2min)
Minutes
0
2
4
6
8
10
12
14
16
0
20
40
60
80

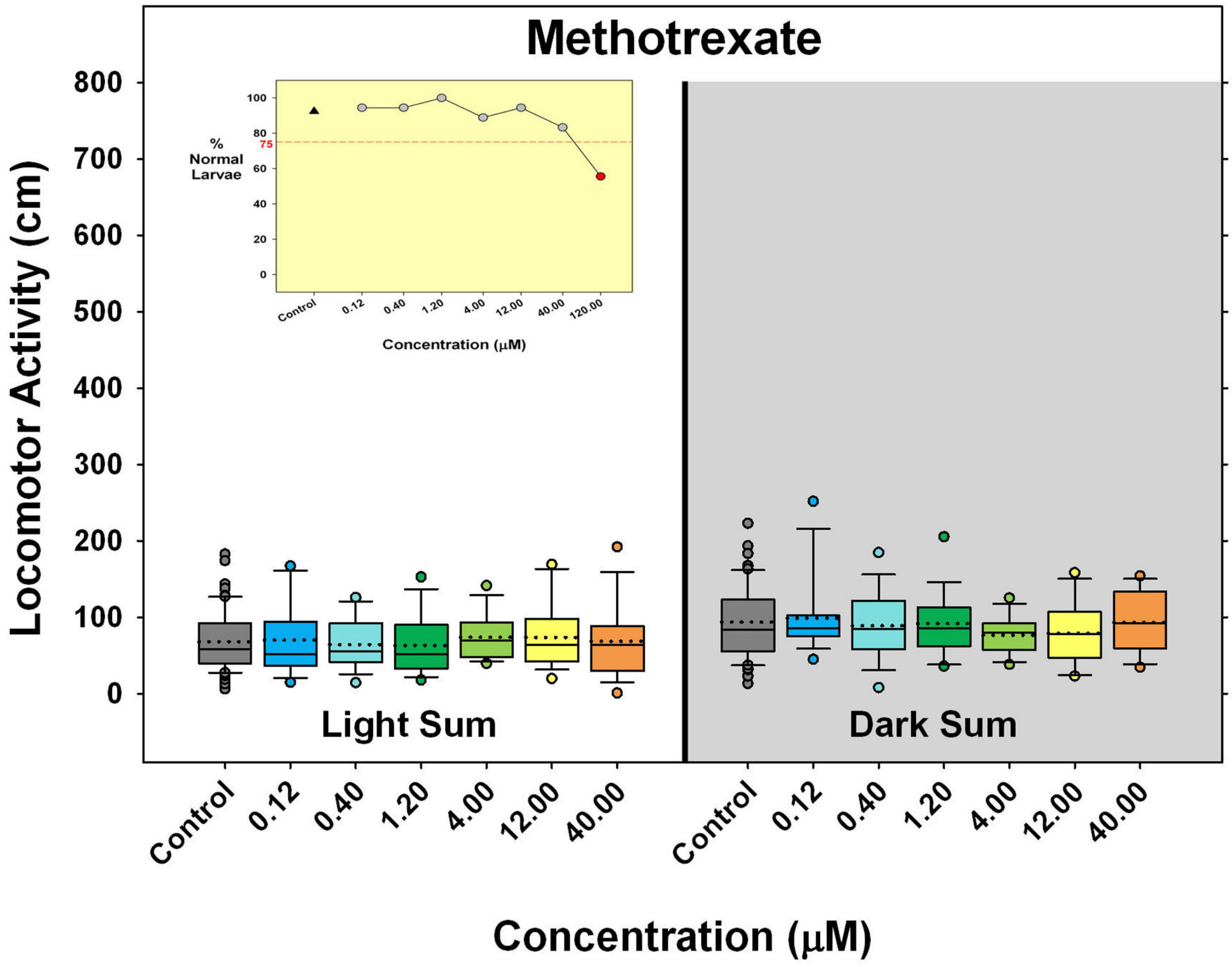
Methotrexate
Locomotor Activity (cm)
0
100
200
300
400
500
600
700
800
% Normal Larvae
75
Concentration (μM)
Control
0.12
0.40
1.20
4.00
12.00
40.00
120.00
Light Sum
Dark Sum
Concentration (μM)

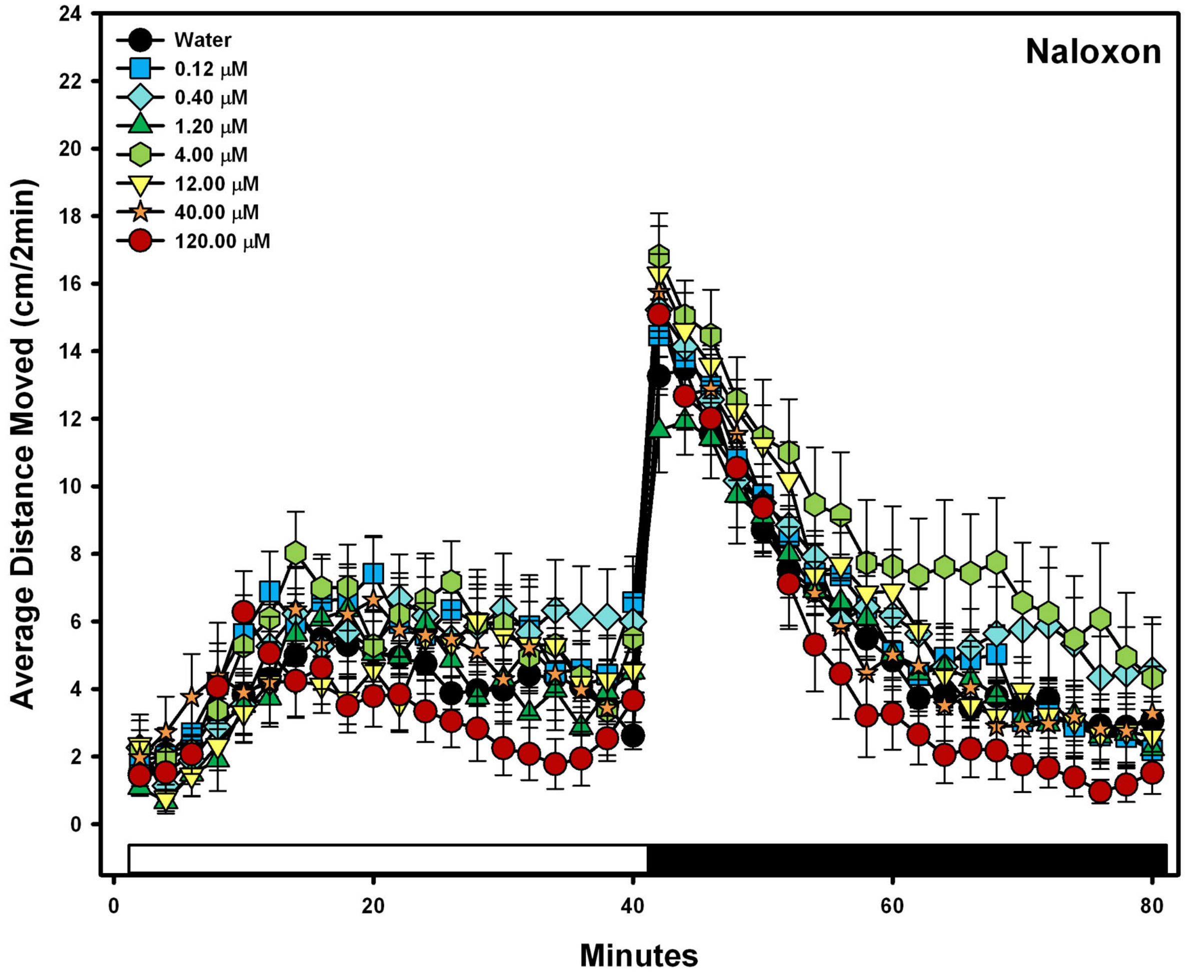

Naloxon
Water
0.12 µM
0.40 µM
1.20 µM
4.00 µM
12.00 µM
40.00 µM
120.00 µM
Average Distance Moved (cm/2min)
Minutes

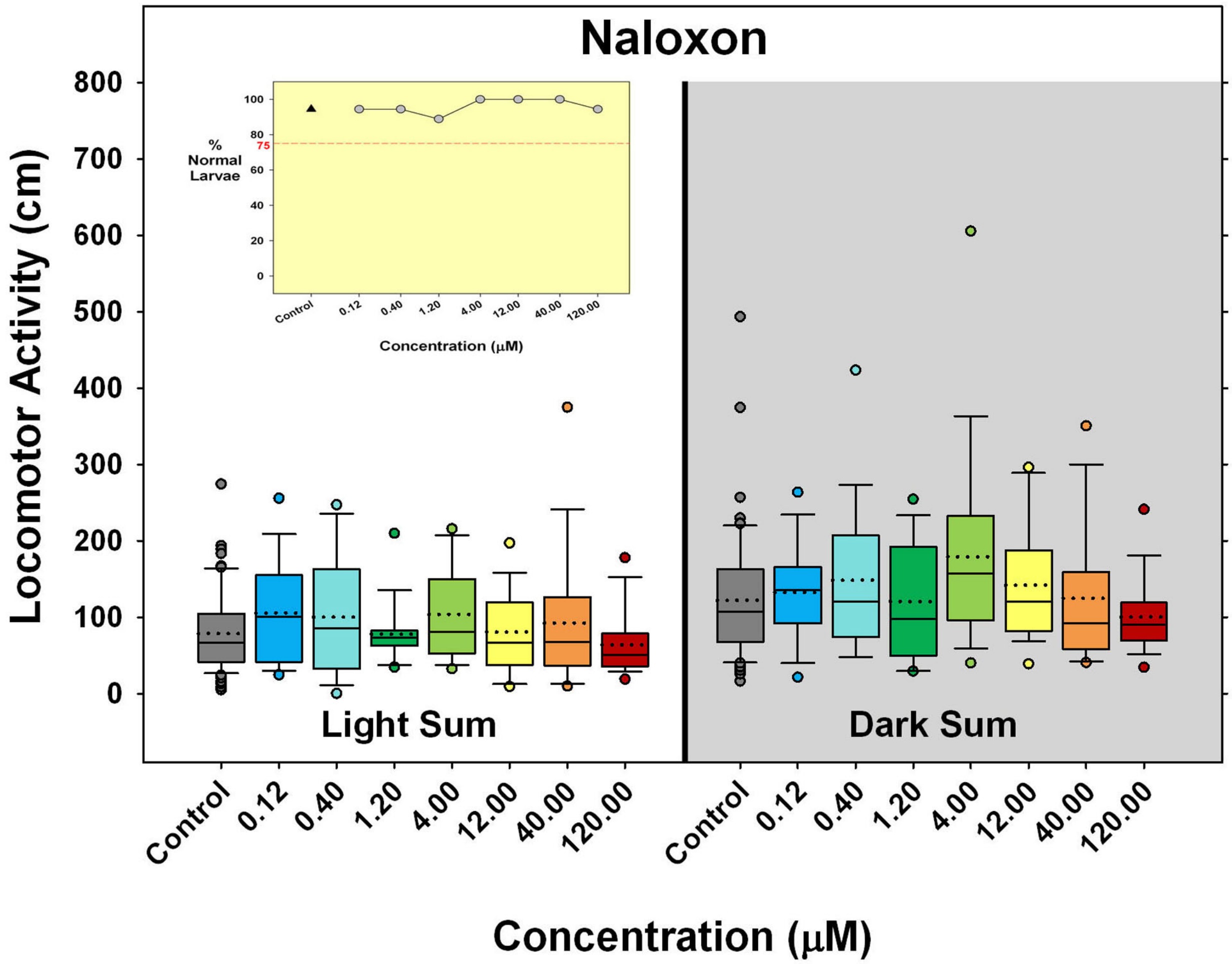

Naloxon
Locomotor Activity (cm)
% Normal Larvae
75
Concentration (µM)
Light Sum
Dark Sum
Control
0.12
0.40
1.20
4.00
12.00
40.00
120.00
Concentration (µM)

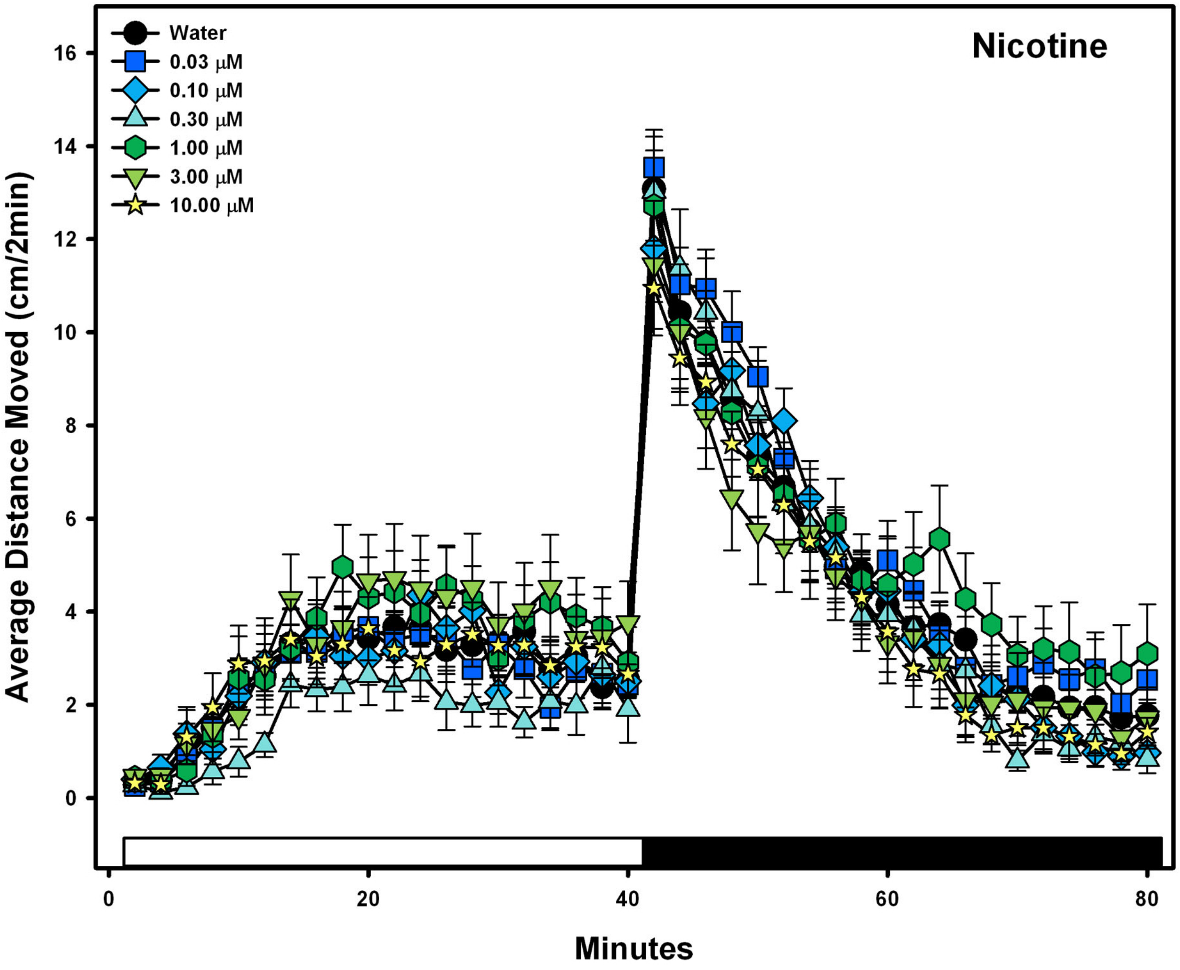

Water
0.03 µM
0.10 µM
0.30 µM
1.00 µM
3.00 µM
10.00 µM
Nicotine
Average Distance Moved (cm/2min)
Minutes

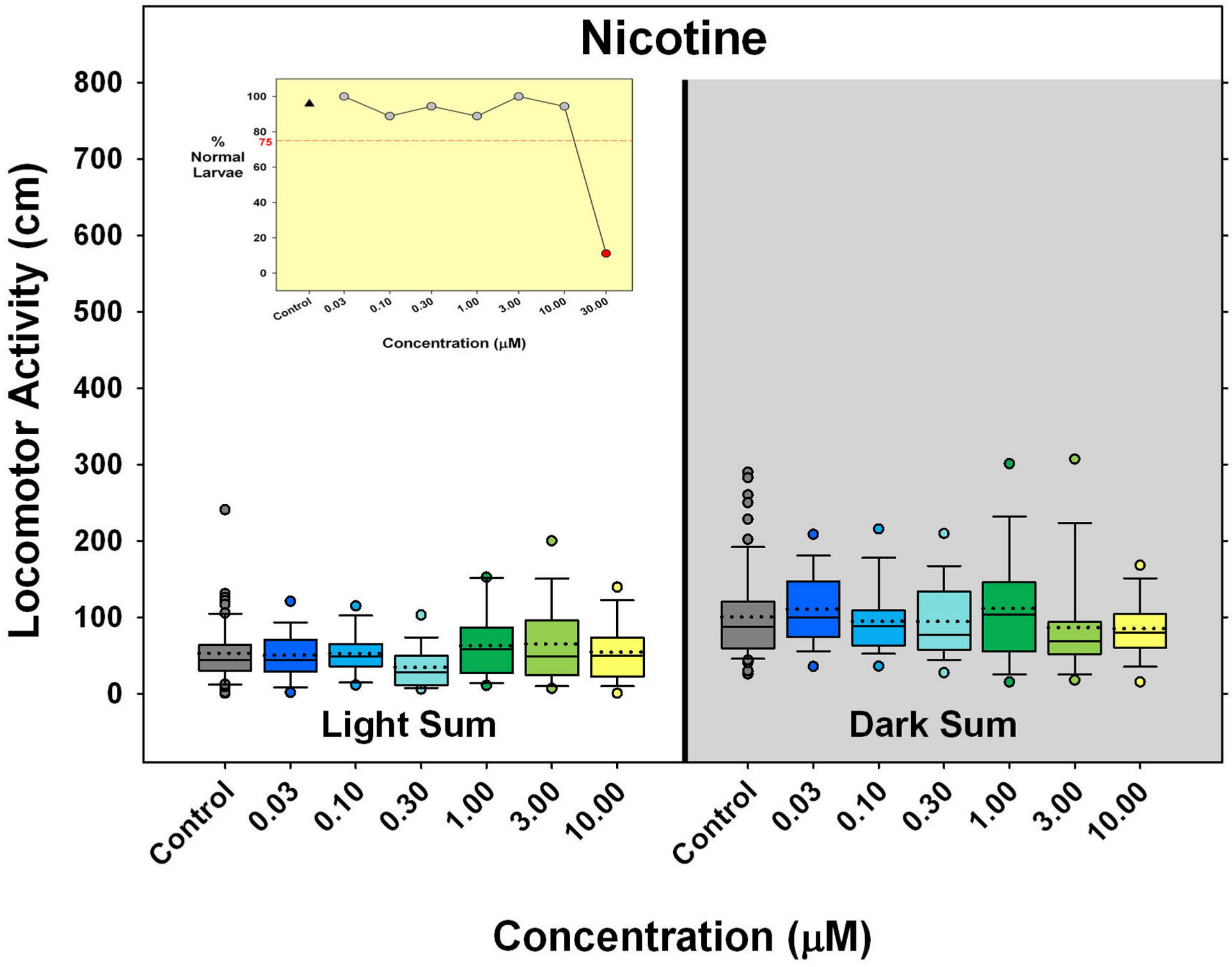

Nicotine
Locomotor Activity (cm)
% Normal Larvae
Concentration (µM)
Light Sum
Dark Sum
Control
0.03
0.10
0.30
1.00
3.00
10.00
30.00
Concentration (µM)

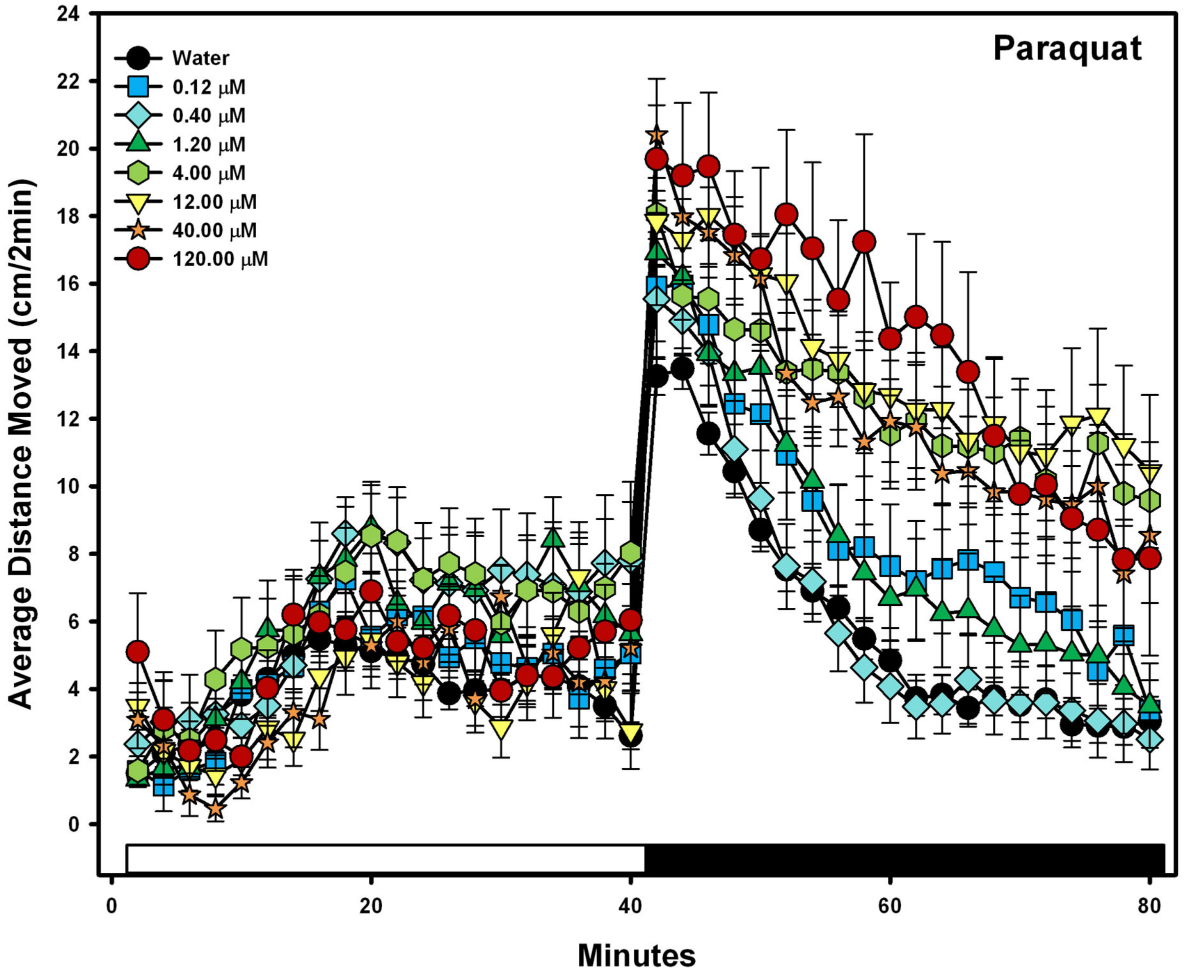
Paraquat
Water
0.12 µM
0.40 µM
1.20 µM
4.00 µM
12.00 µM
40.00 µM
120.00 µM
Average Distance Moved (cm/2min)
Minutes

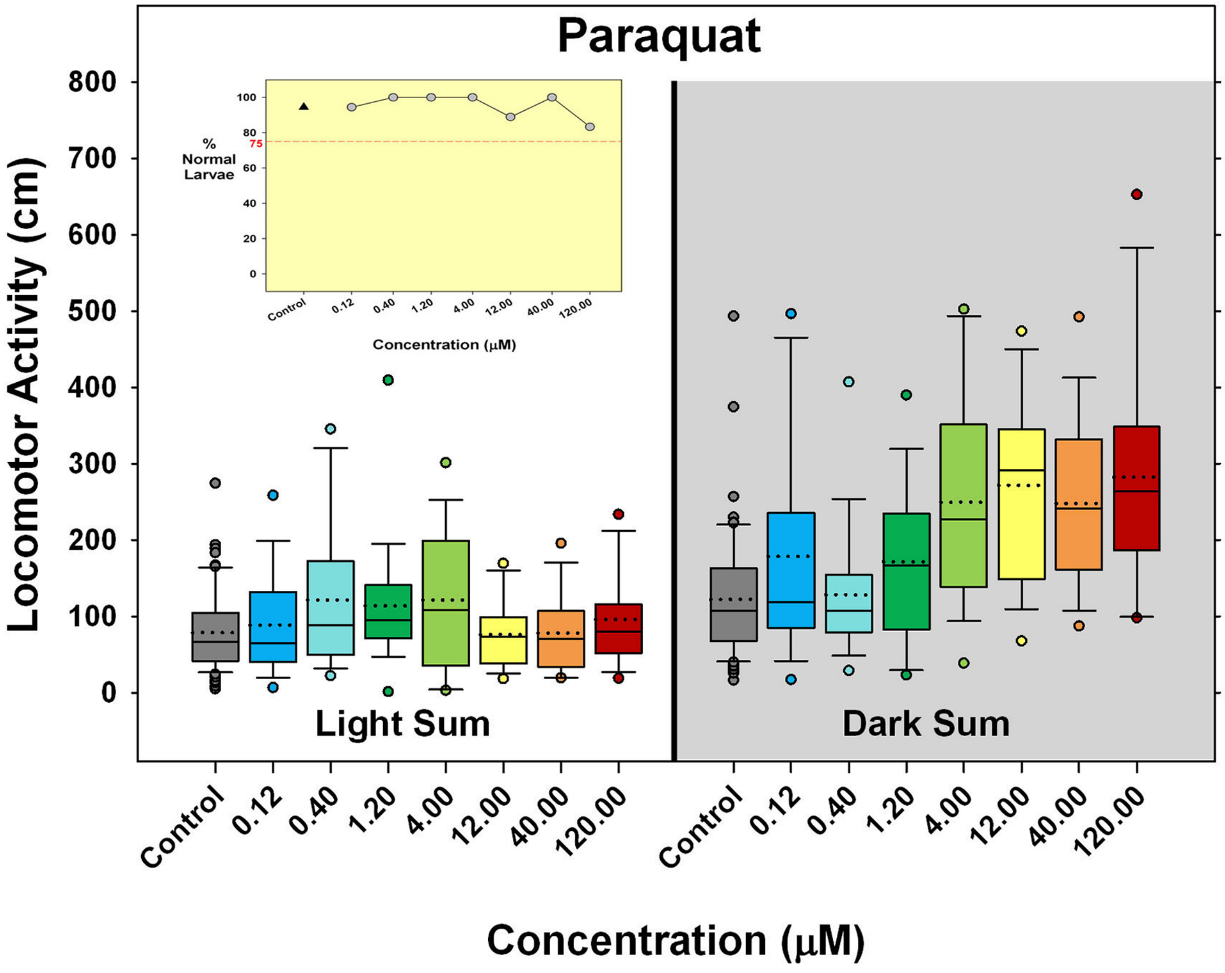
Paraquat
Locomotor Activity (cm)
% Normal Larvae
75
Concentration (µM)
Light Sum
Dark Sum
Control
0.12
0.40
1.20
4.00
12.00
40.00
120.00
Concentration (µM)

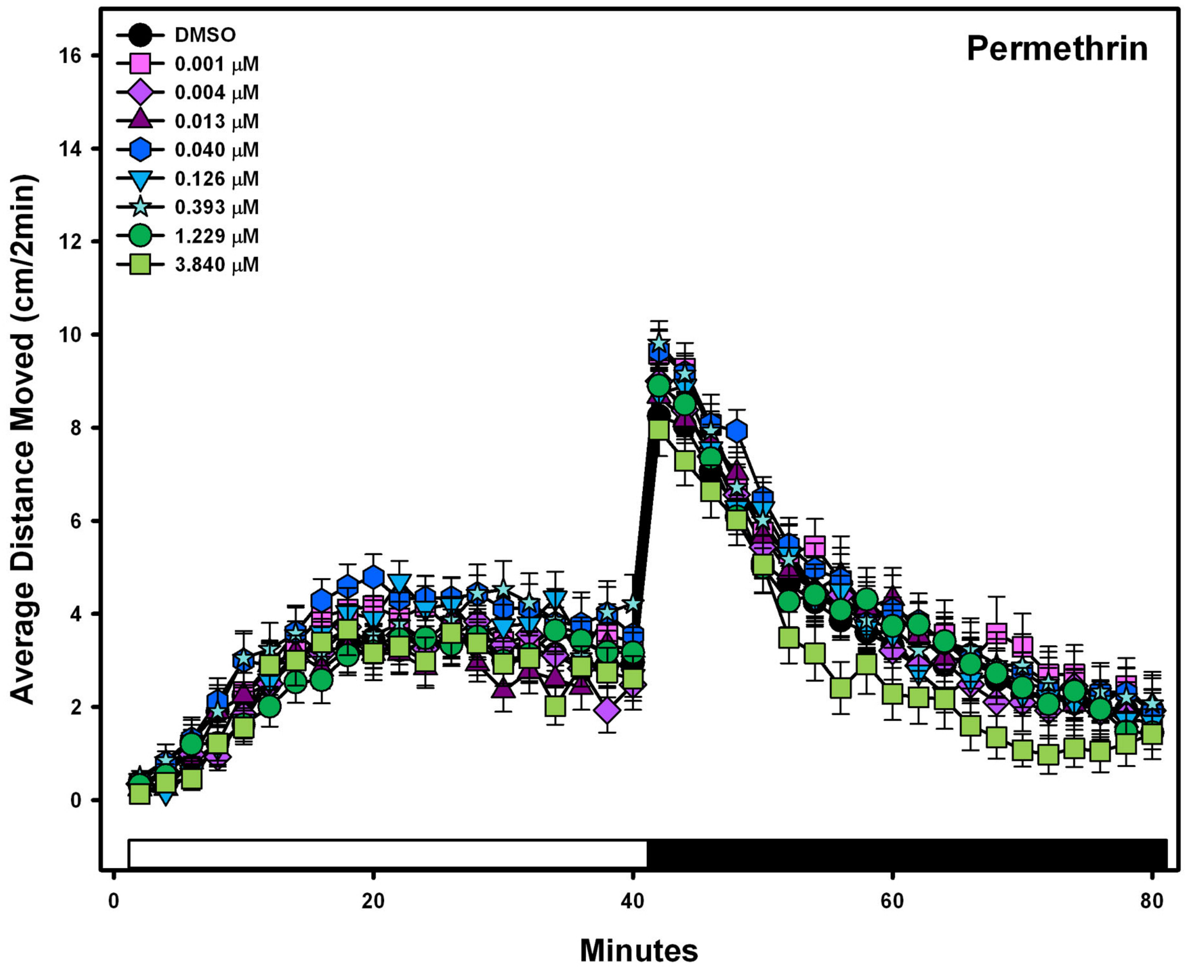
Permethrin
DMSO
0.001 µM
0.004 µM
0.013 µM
0.040 µM
0.126 µM
0.393 µM
1.229 µM
3.840 µM
Average Distance Moved (cm/2min)
Minutes

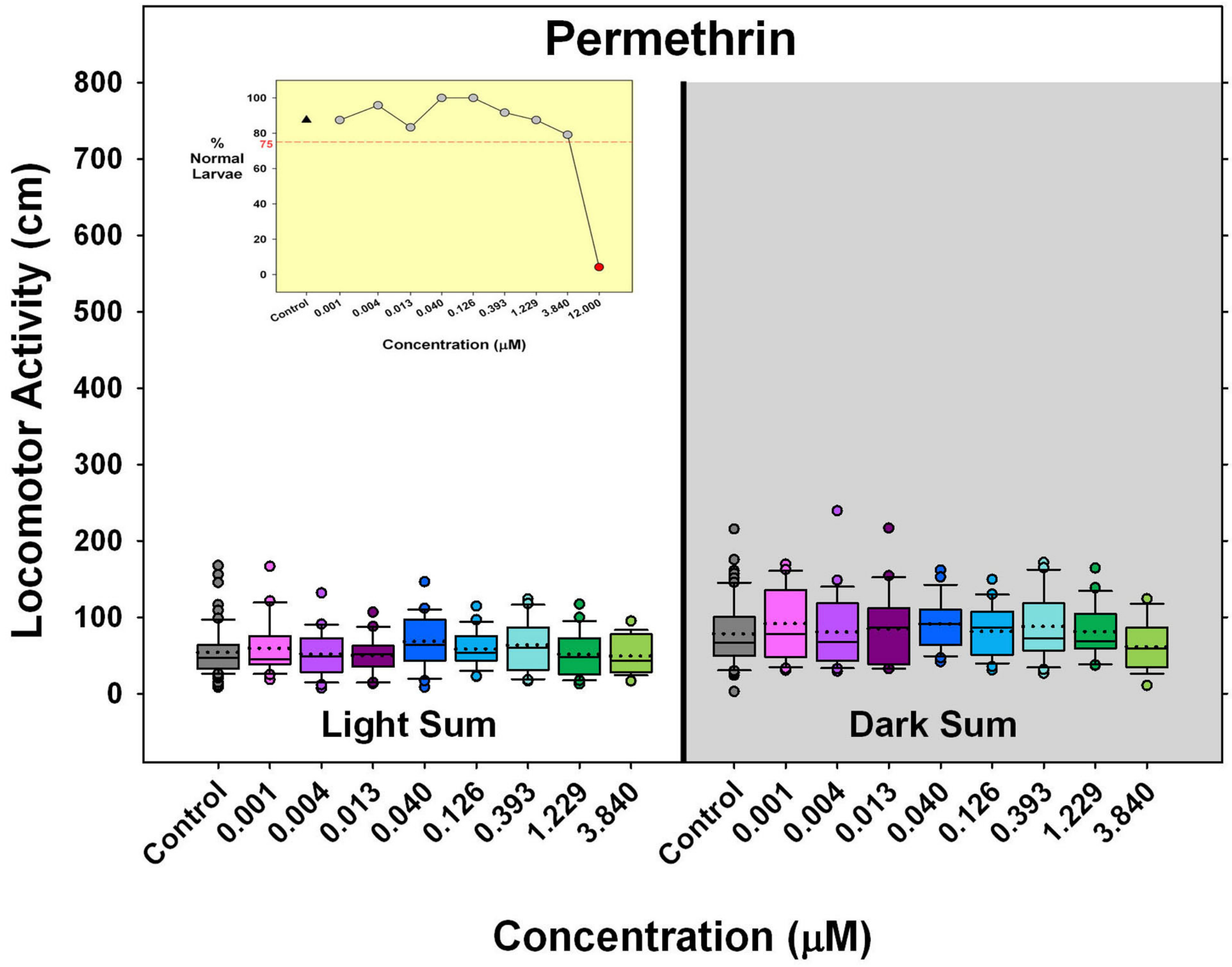
Permethrin
Locomotor Activity (cm)
% Normal Larvae
Concentration (µM)
Light Sum
Dark Sum
Control
0.001
0.004
0.013
0.040
0.126
0.393
1.229
3.840
12.000
Concentration (µM)

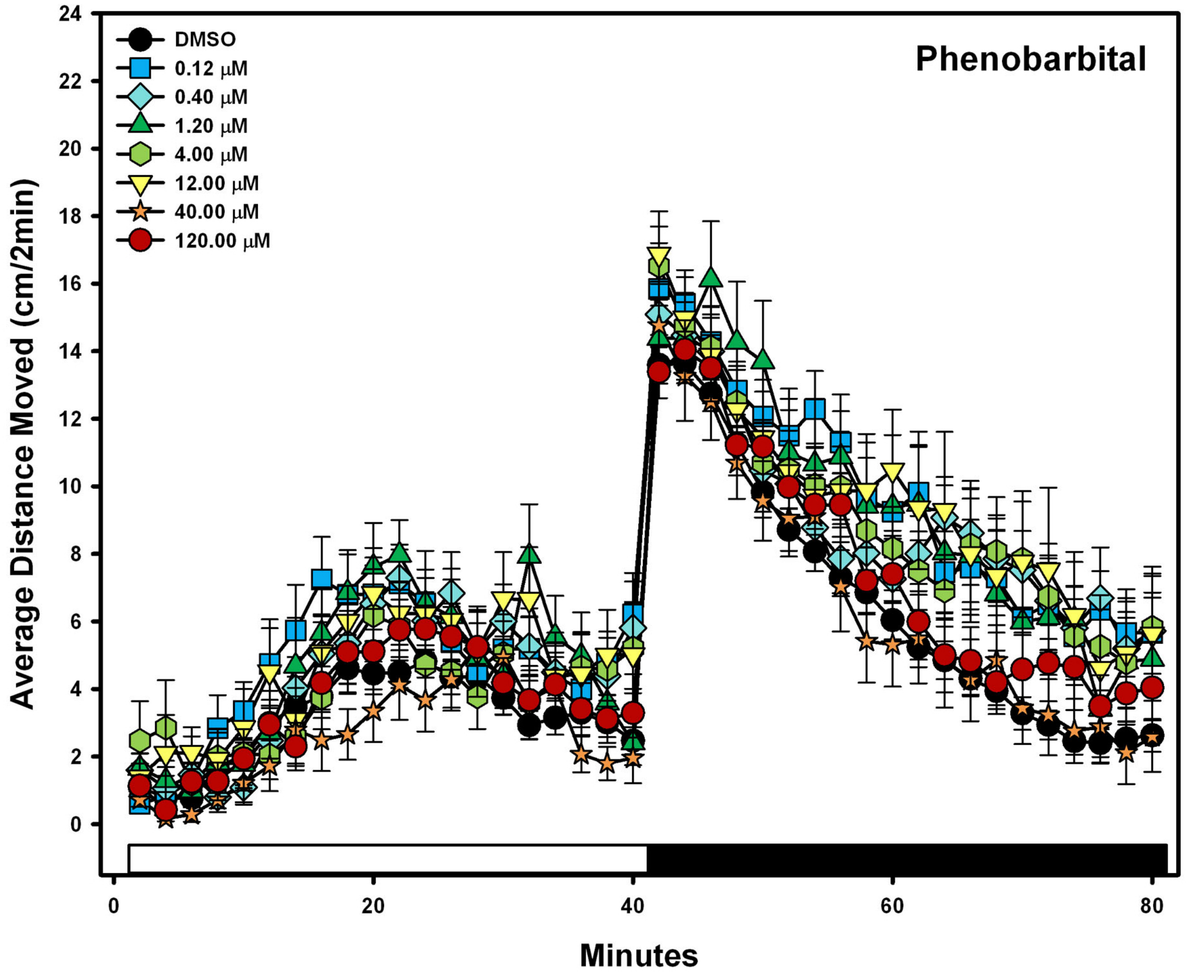
Phenobarbital
DMSO
0.12 μM
0.40 μM
1.20 μM
4.00 μM
12.00 μM
40.00 μM
120.00 μM
Average Distance Moved (cm/2min)
Minutes
0
20
40
60
80
0
2
4
6
8
10
12
14
16
18
20
22
24

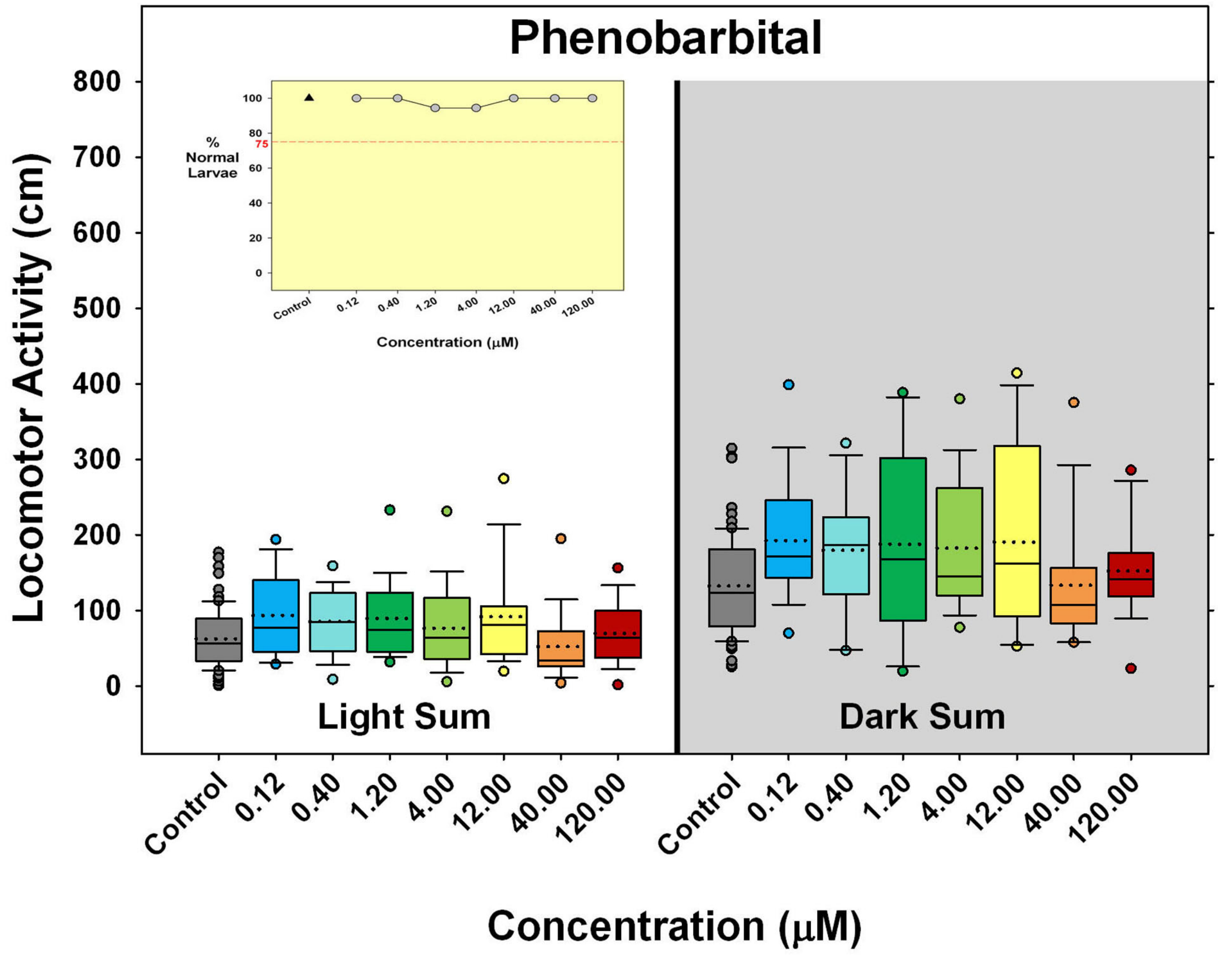
Phenobarbital
Locomotor Activity (cm)
0
100
200
300
400
500
600
700
800
% Normal Larvae
75
Concentration (μM)
Light Sum
Dark Sum
Control
0.12
0.40
1.20
4.00
12.00
40.00
120.00
Concentration (μM)

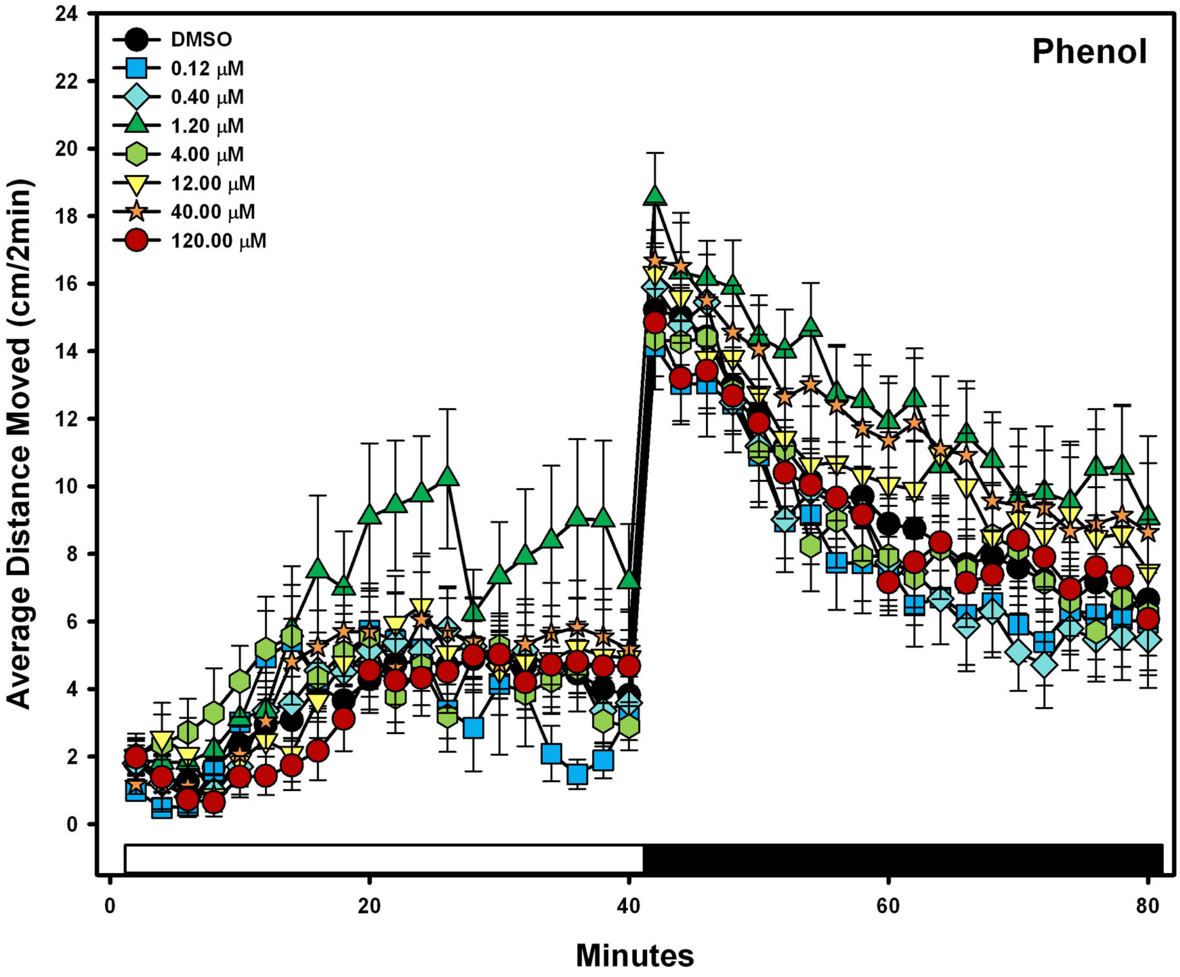

Phenol
DMSO
0.12 μM
0.40 μM
1.20 μM
4.00 μM
12.00 μM
40.00 μM
120.00 μM
Average Distance Moved (cm/2min)
Minutes

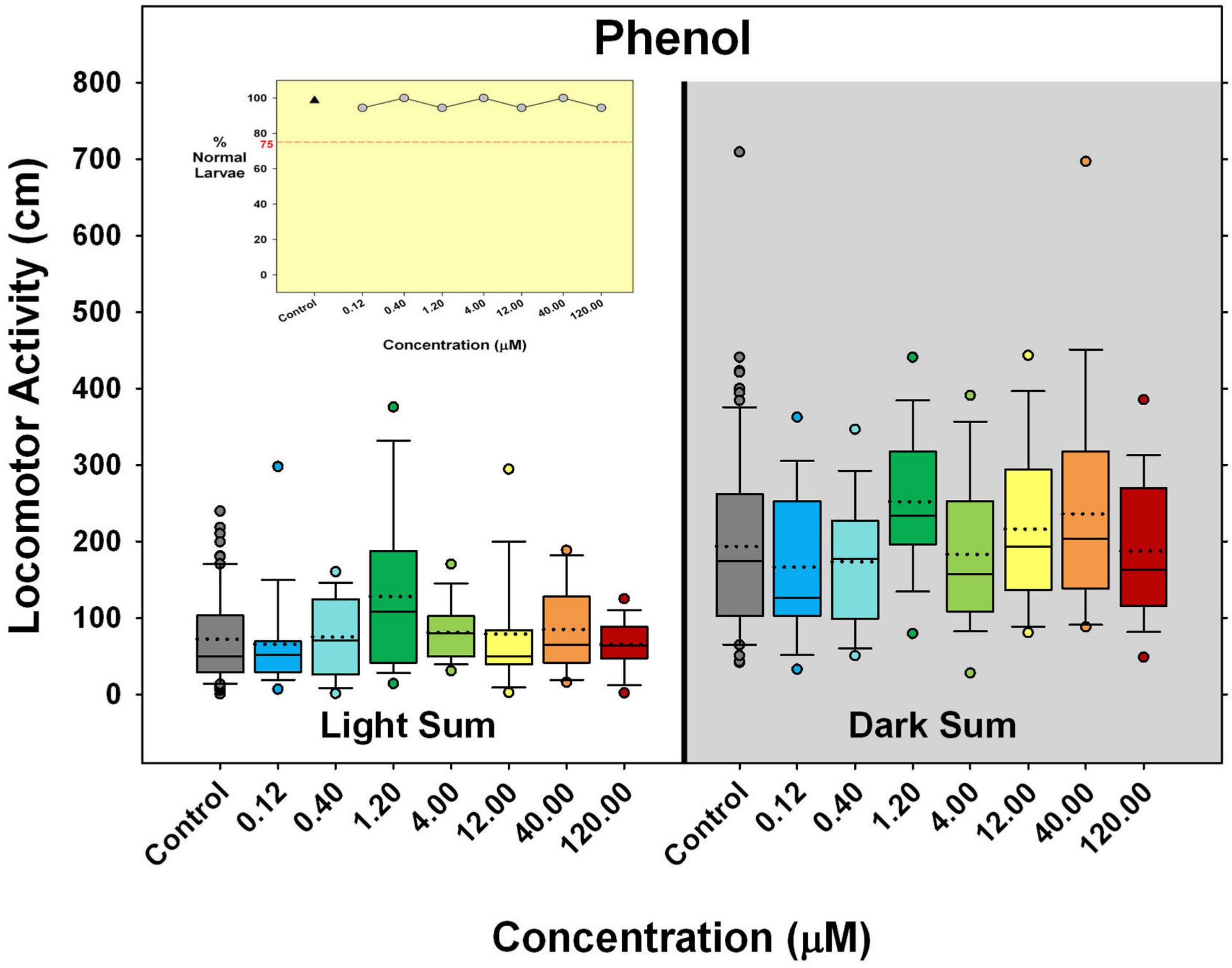

Phenol
Locomotor Activity (cm)
% Normal Larvae
75
Concentration (μM)
Light Sum
Dark Sum
Control
0.12
0.40
1.20
4.00
12.00
40.00
120.00
Concentration (μM)

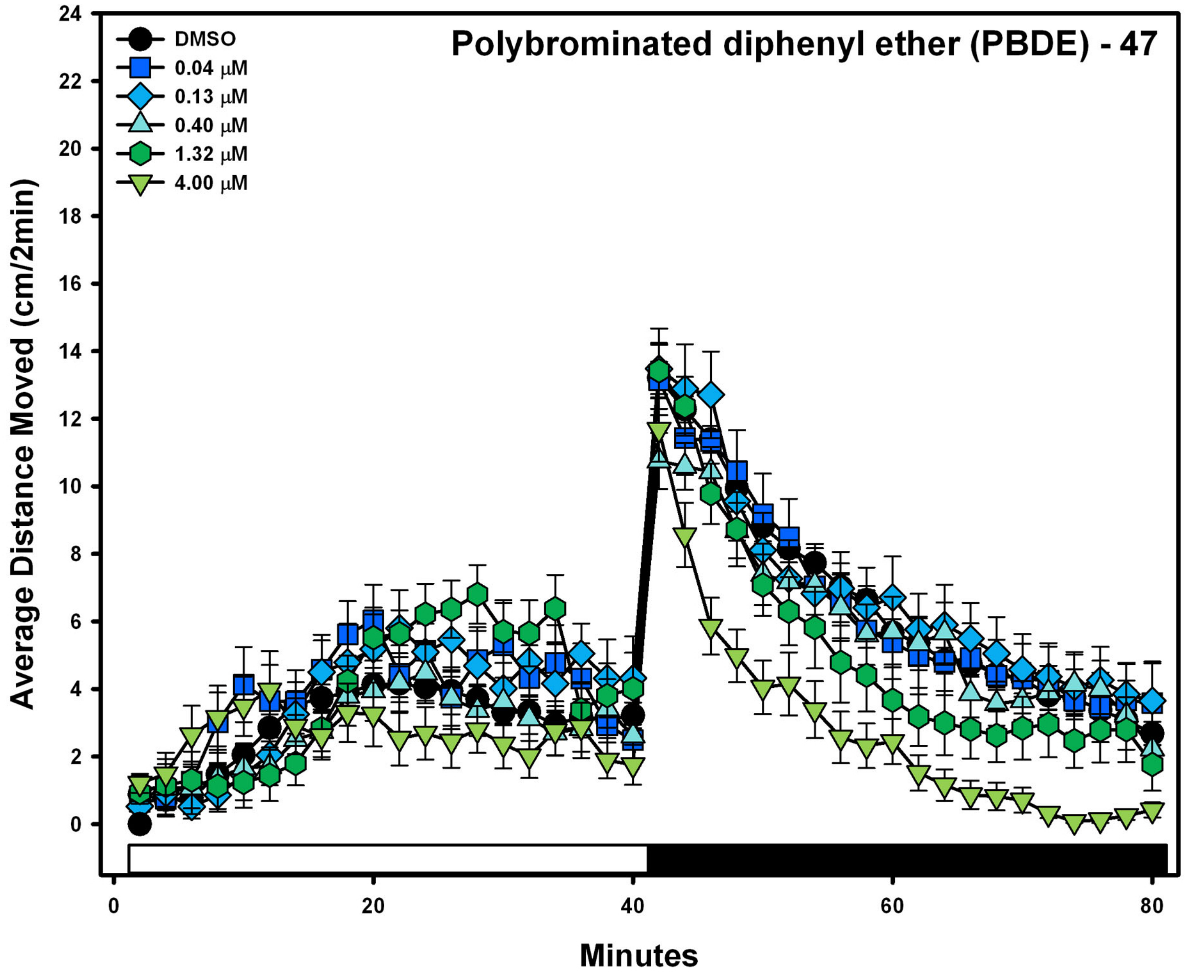
Polybrominated diphenyl ether (PBDE) - 47
DMSO
0.04 µM
0.13 µM
0.40 µM
1.32 µM
4.00 µM
Average Distance Moved (cm/2min)
Minutes

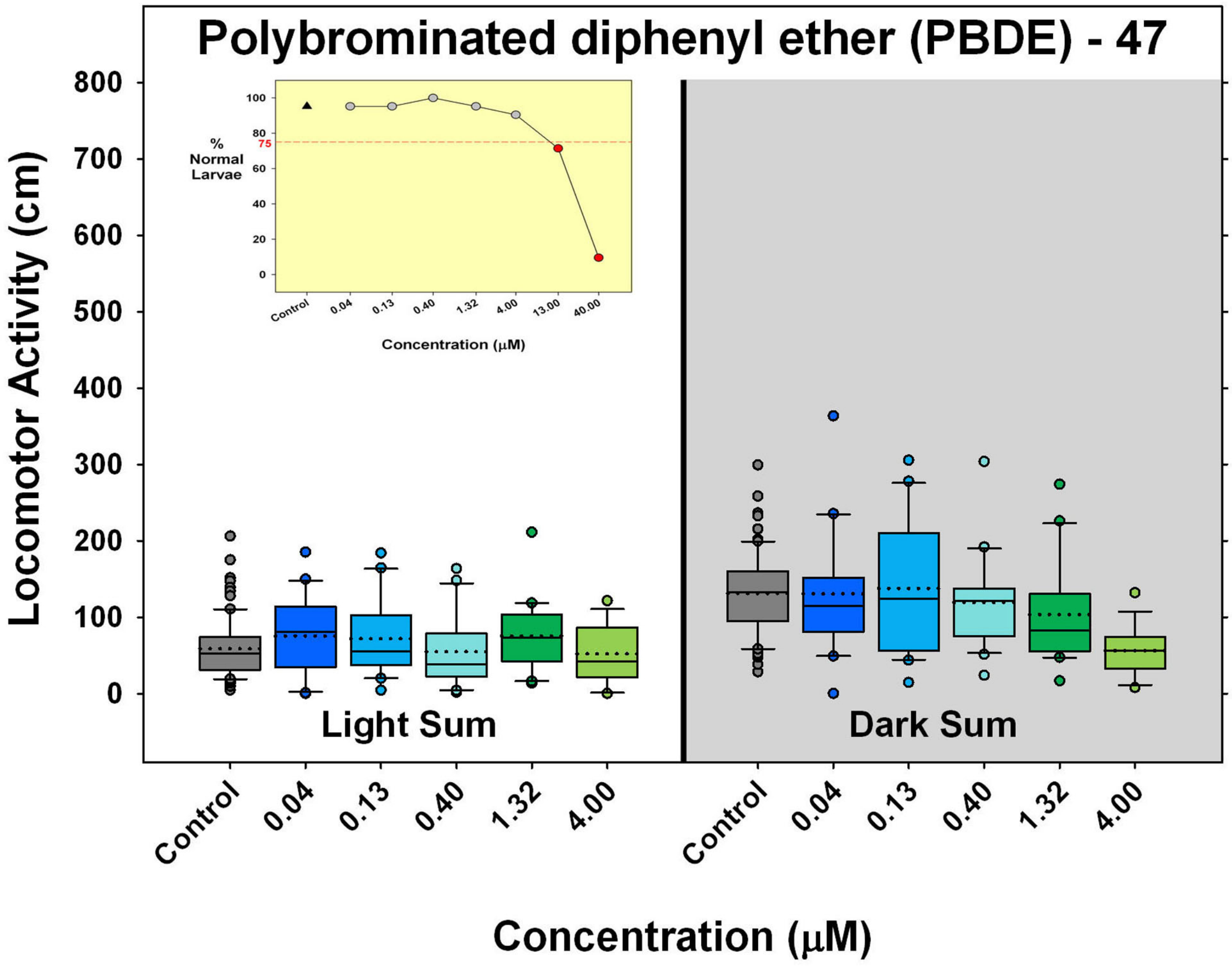
Polybrominated diphenyl ether (PBDE) - 47
Locomotor Activity (cm)
% Normal Larvae
Concentration (µM)
Light Sum
Dark Sum
Control
0.04
0.13
0.40
1.32
4.00
Concentration (µM)

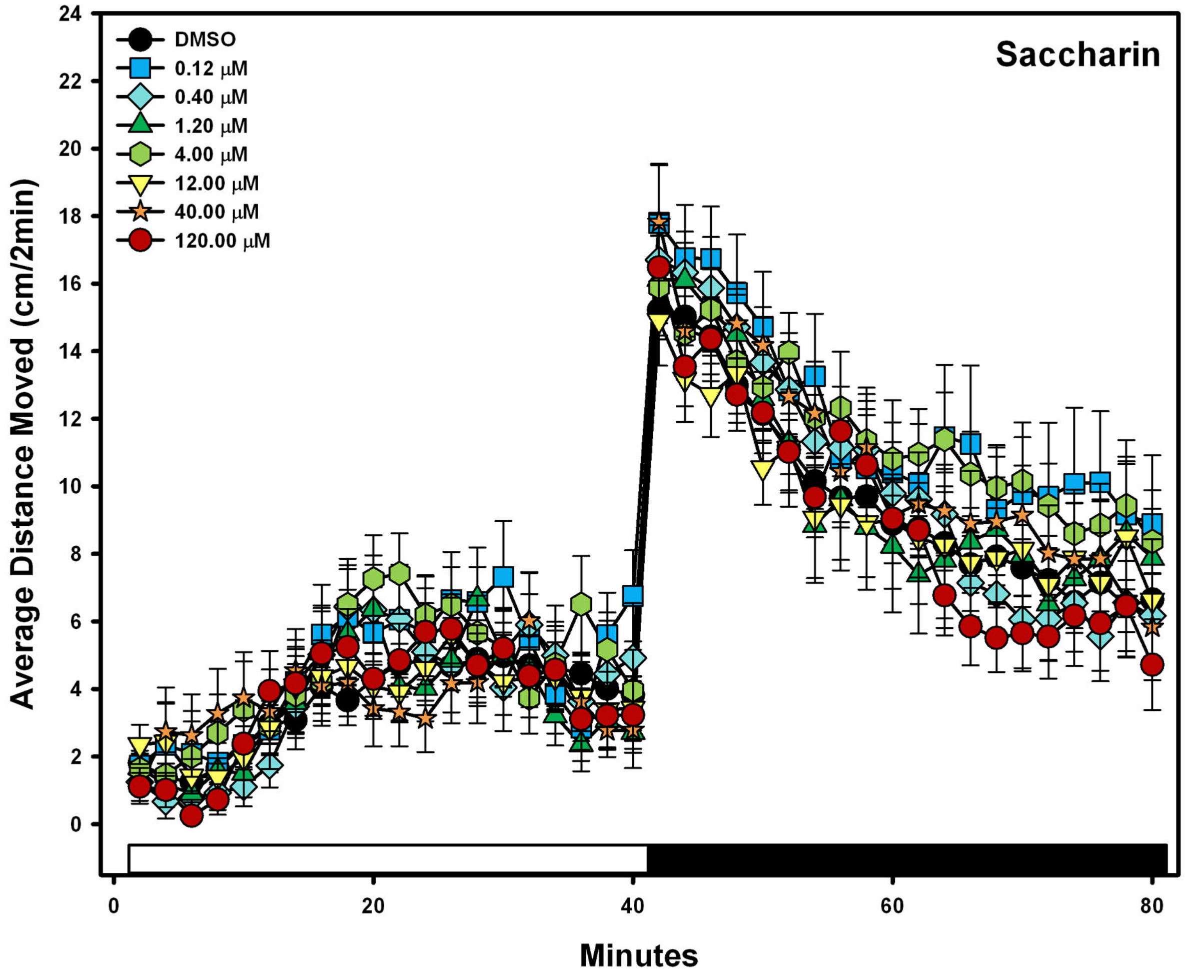

Saccharin
DMSO
0.12 µM
0.40 µM
1.20 µM
4.00 µM
12.00 µM
40.00 µM
120.00 µM
Average Distance Moved (cm/2min)
Minutes

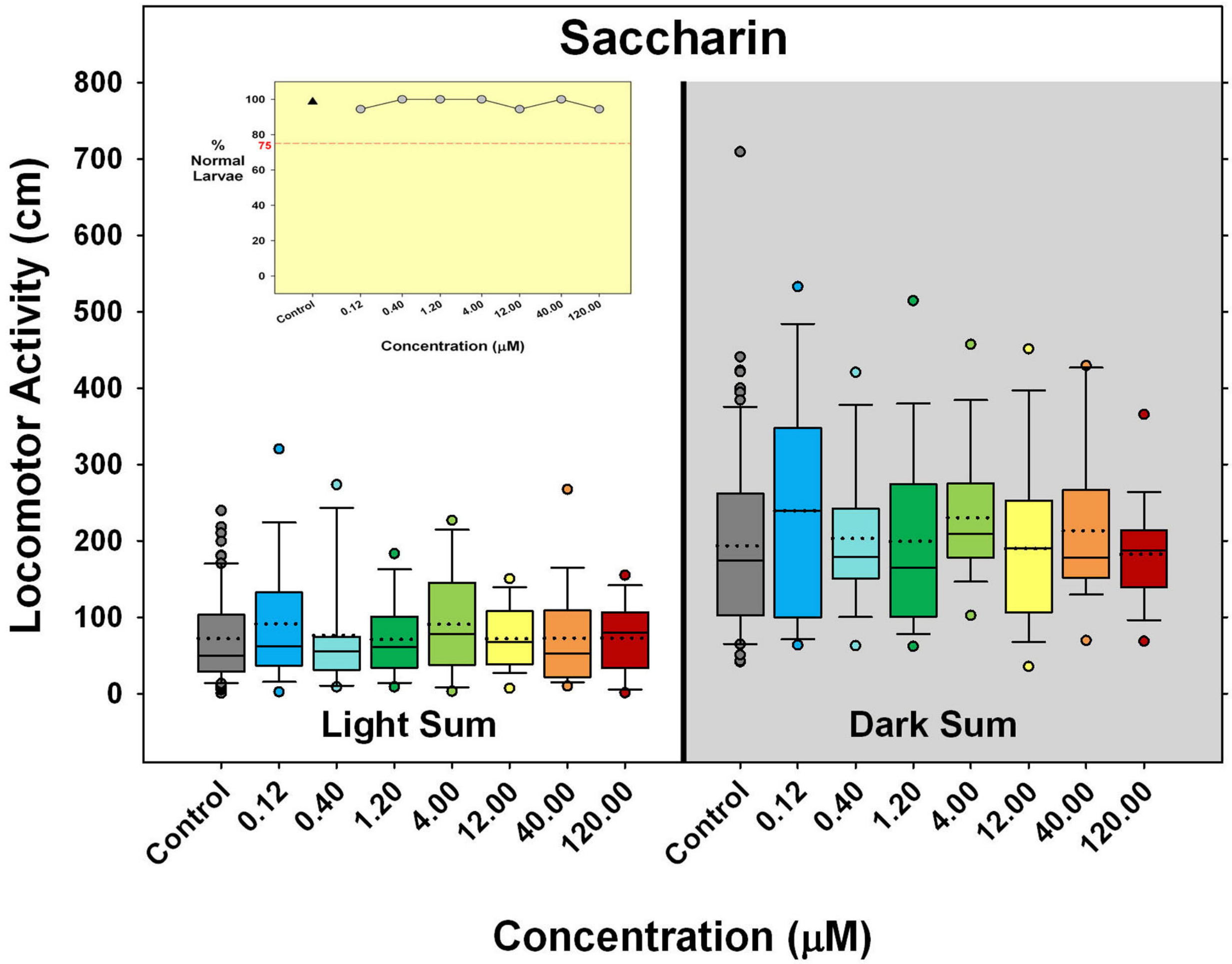

Saccharin
Locomotor Activity (cm)
% Normal Larvae
Concentration (µM)
Light Sum
Dark Sum
Control
0.12
0.40
1.20
4.00
12.00
40.00
120.00
Concentration (µM)

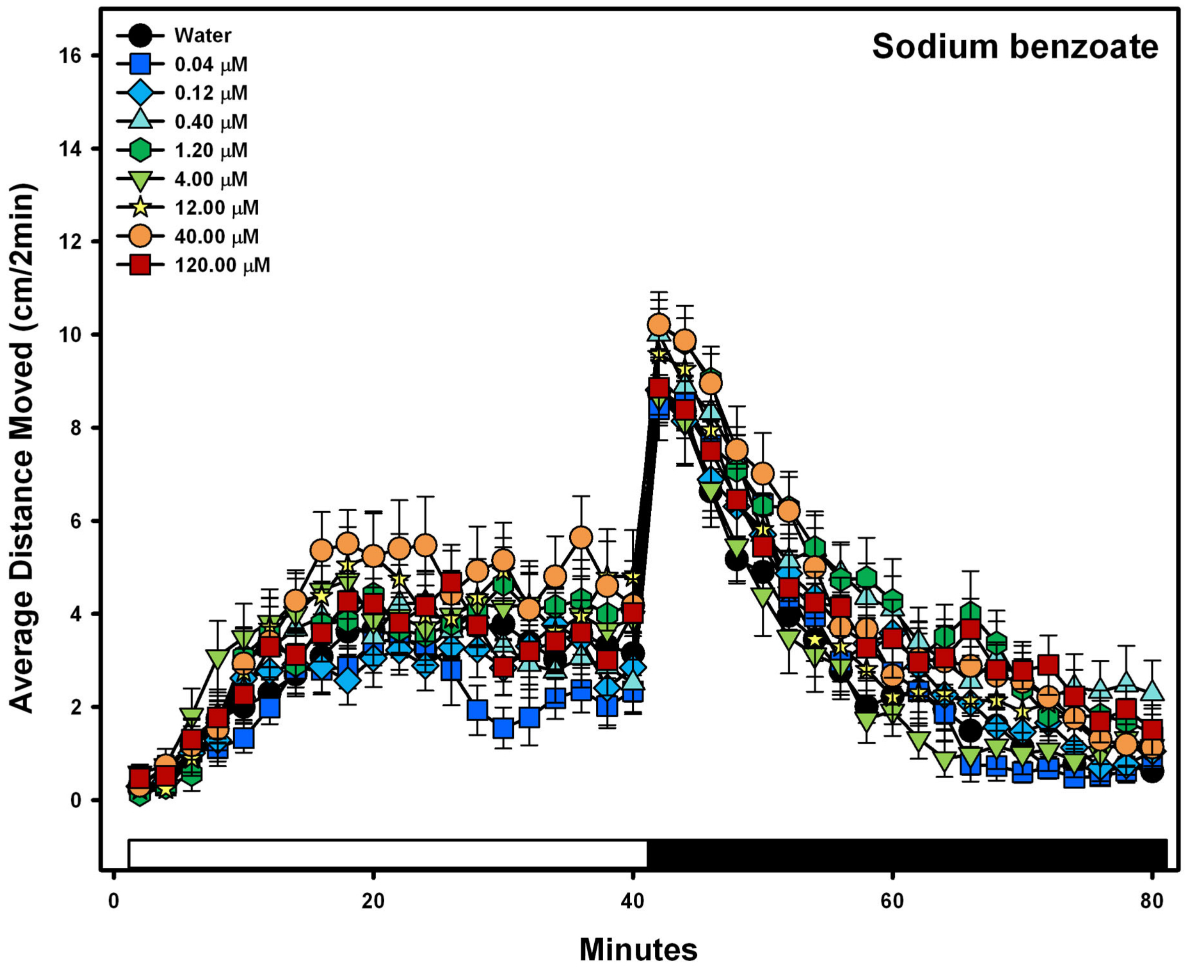

Water
0.04 µM
0.12 µM
0.40 µM
1.20 µM
4.00 µM
12.00 µM
40.00 µM
120.00 µM
Sodium benzoate
Average Distance Moved (cm/2min)
Minutes

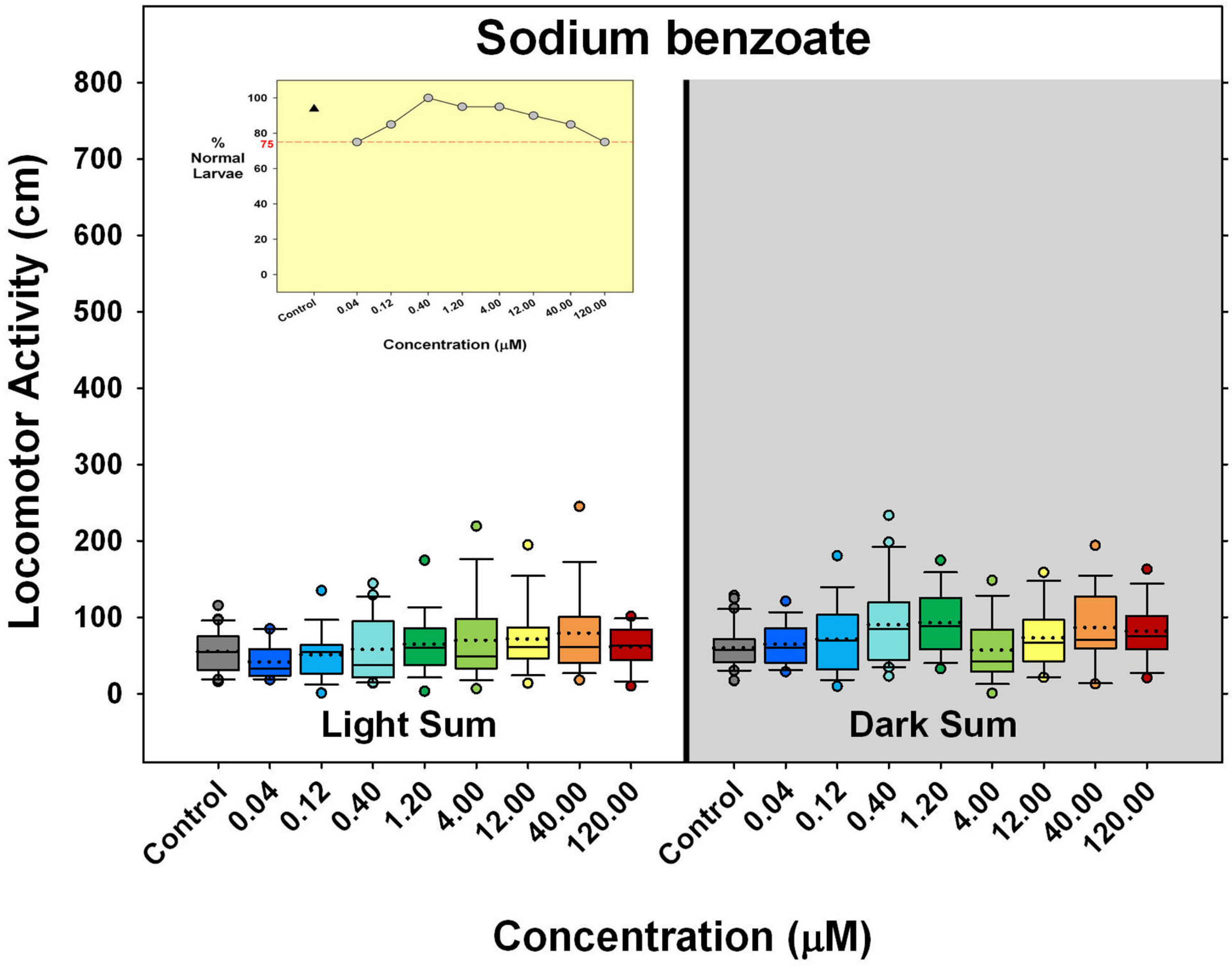

Sodium benzoate
Locomotor Activity (cm)
% Normal Larvae
75
Concentration (µM)
Light Sum
Dark Sum
Control 0.04 0.12 0.40 1.20 4.00 12.00 40.00 120.00
Concentration (µM)

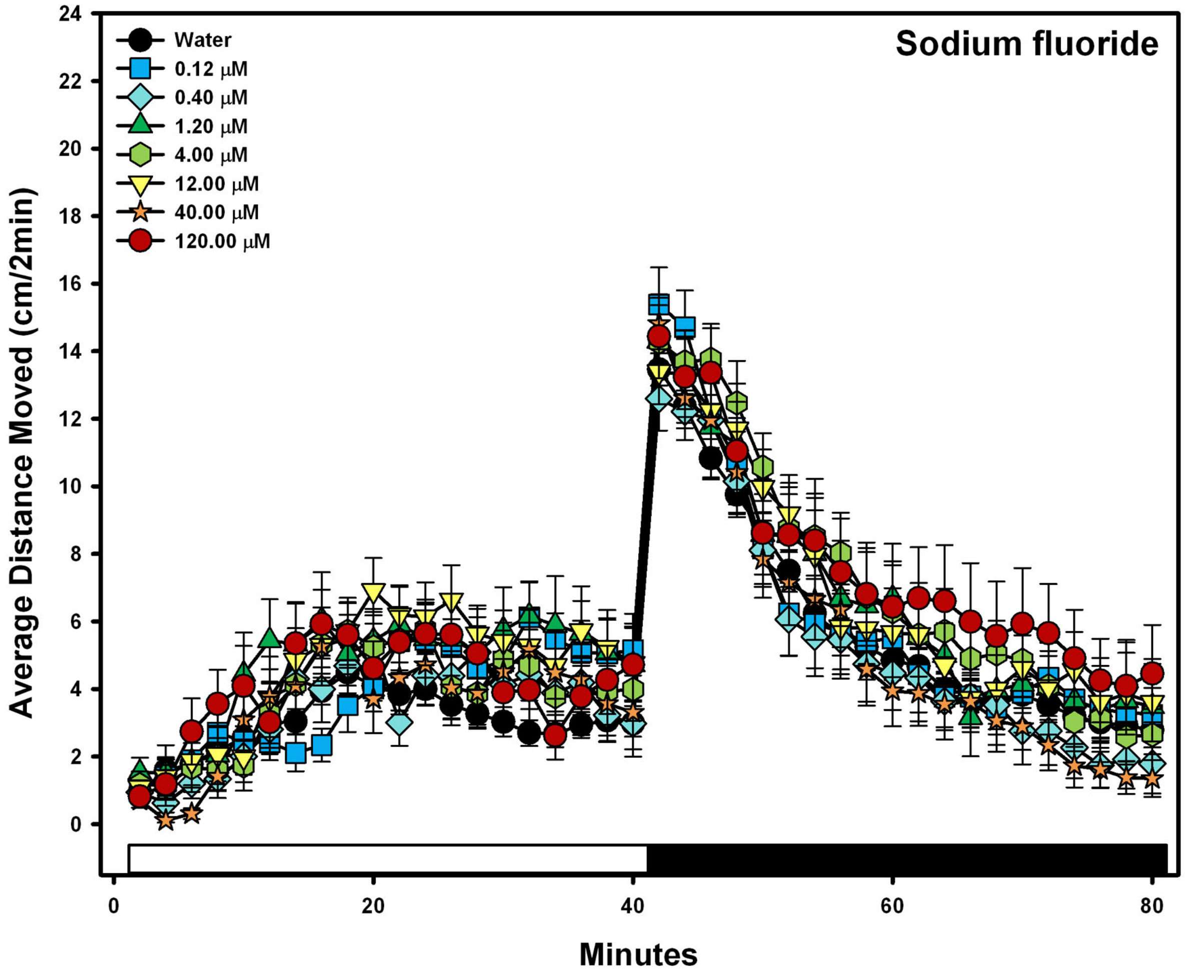
Sodium fluoride
Water
0.12 µM
0.40 µM
1.20 µM
4.00 µM
12.00 µM
40.00 µM
120.00 µM
Average Distance Moved (cm/2min)
Minutes
0
20
40
60
80

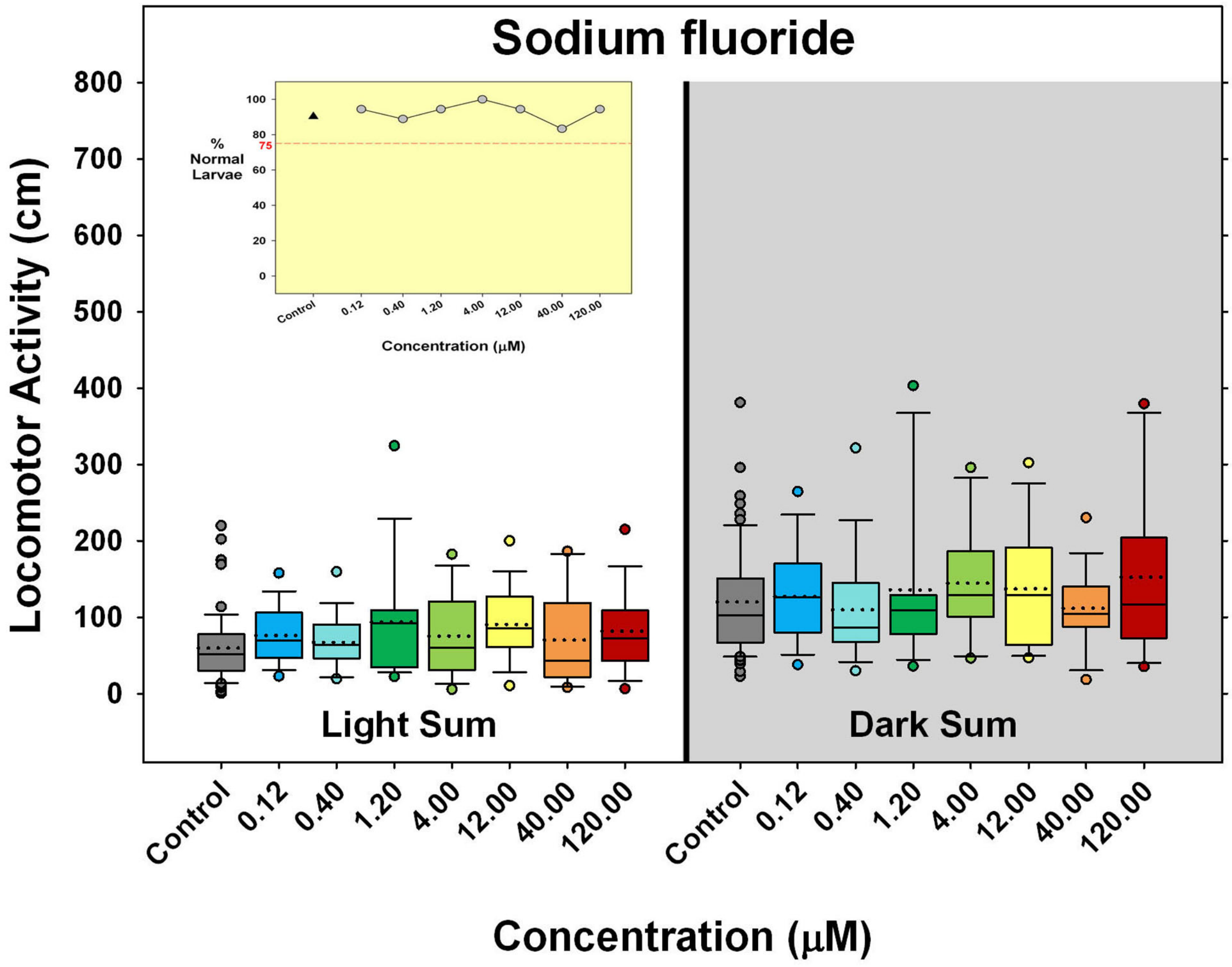
Sodium fluoride
Locomotor Activity (cm)
% Normal Larvae
75
Concentration (µM)
Light Sum
Dark Sum
Control
0.12
0.40
1.20
4.00
12.00
40.00
120.00
Concentration (µM)

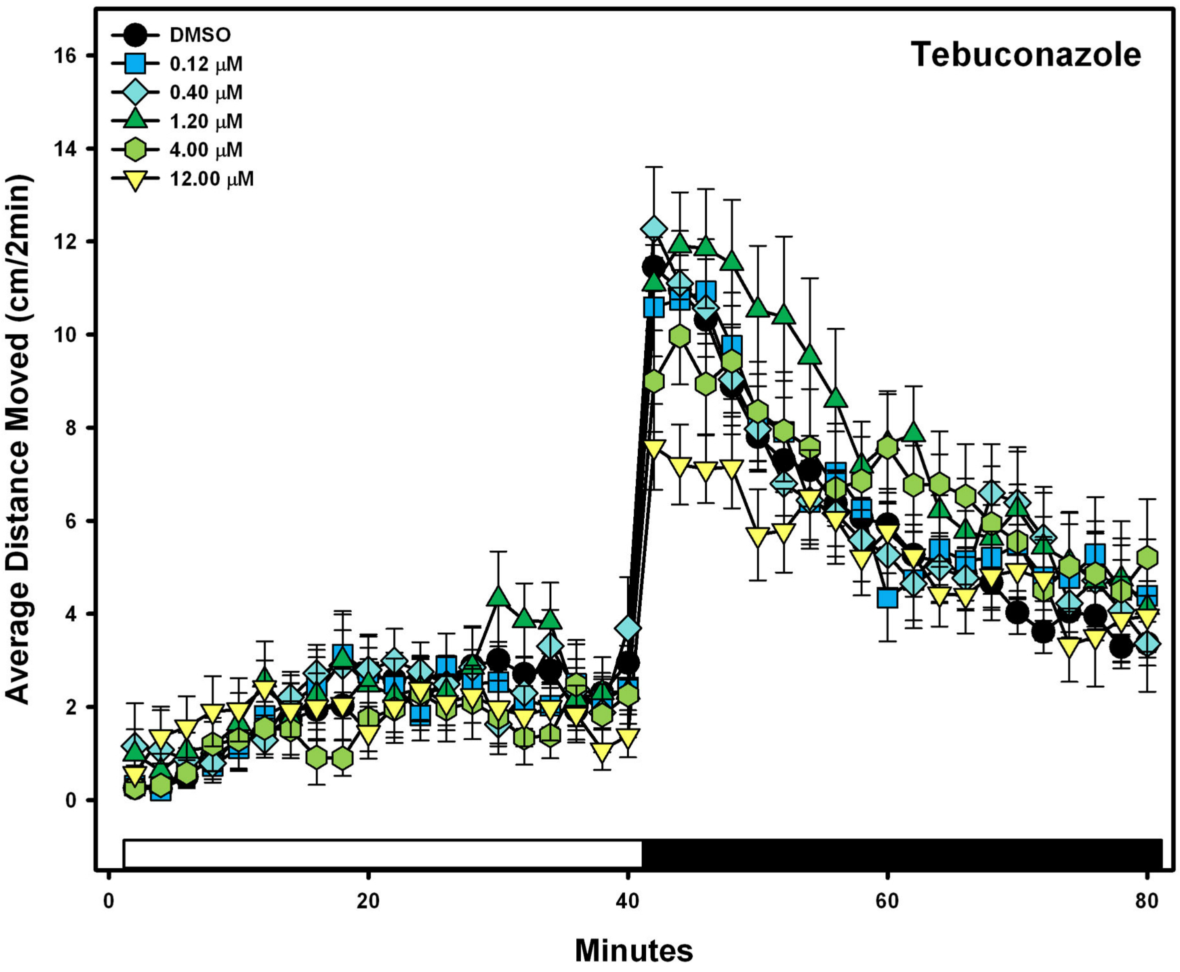

DMSO
0.12 μM
0.40 μM
1.20 μM
4.00 μM
12.00 μM
Tebuconazole
Average Distance Moved (cm/2min)
Minutes

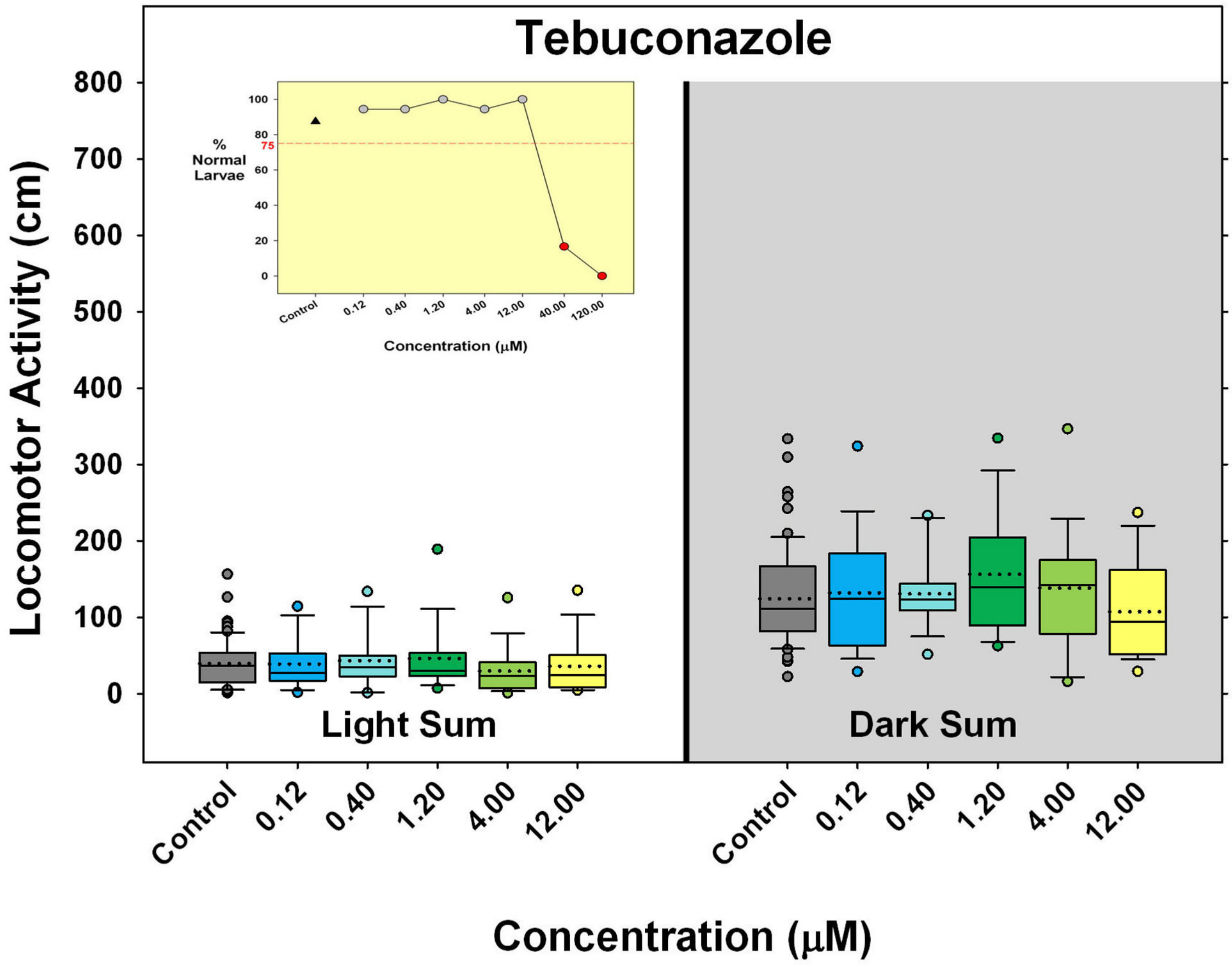

Tebuconazole
Locomotor Activity (cm)
% Normal Larvae
Concentration (μM)
Light Sum
Dark Sum
Control
0.12
0.40
1.20
4.00
12.00
40.00
120.00

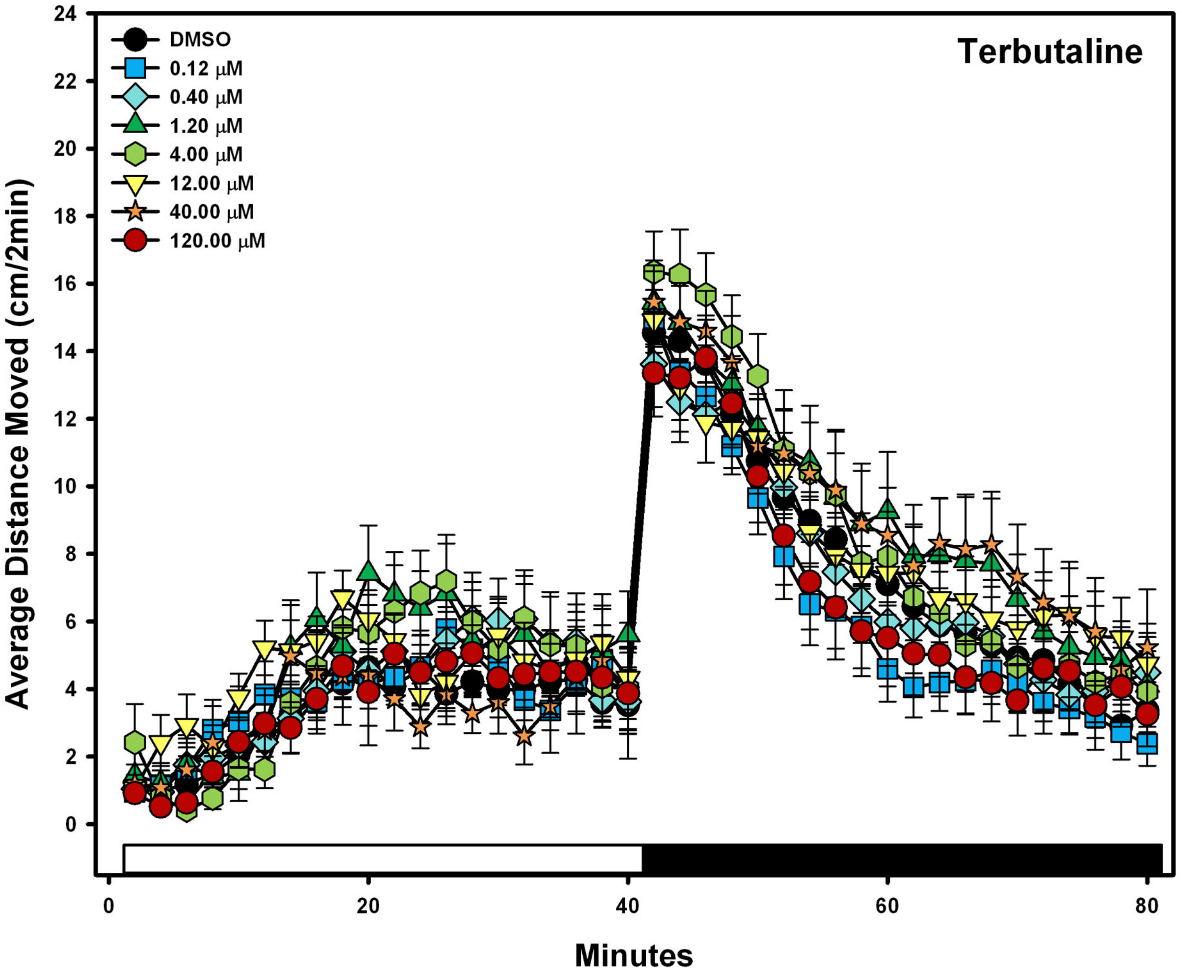

Terbutaline
DMSO
0.12 μM
0.40 μM
1.20 μM
4.00 μM
12.00 μM
40.00 μM
120.00 μM
Average Distance Moved (cm/2min)
Minutes

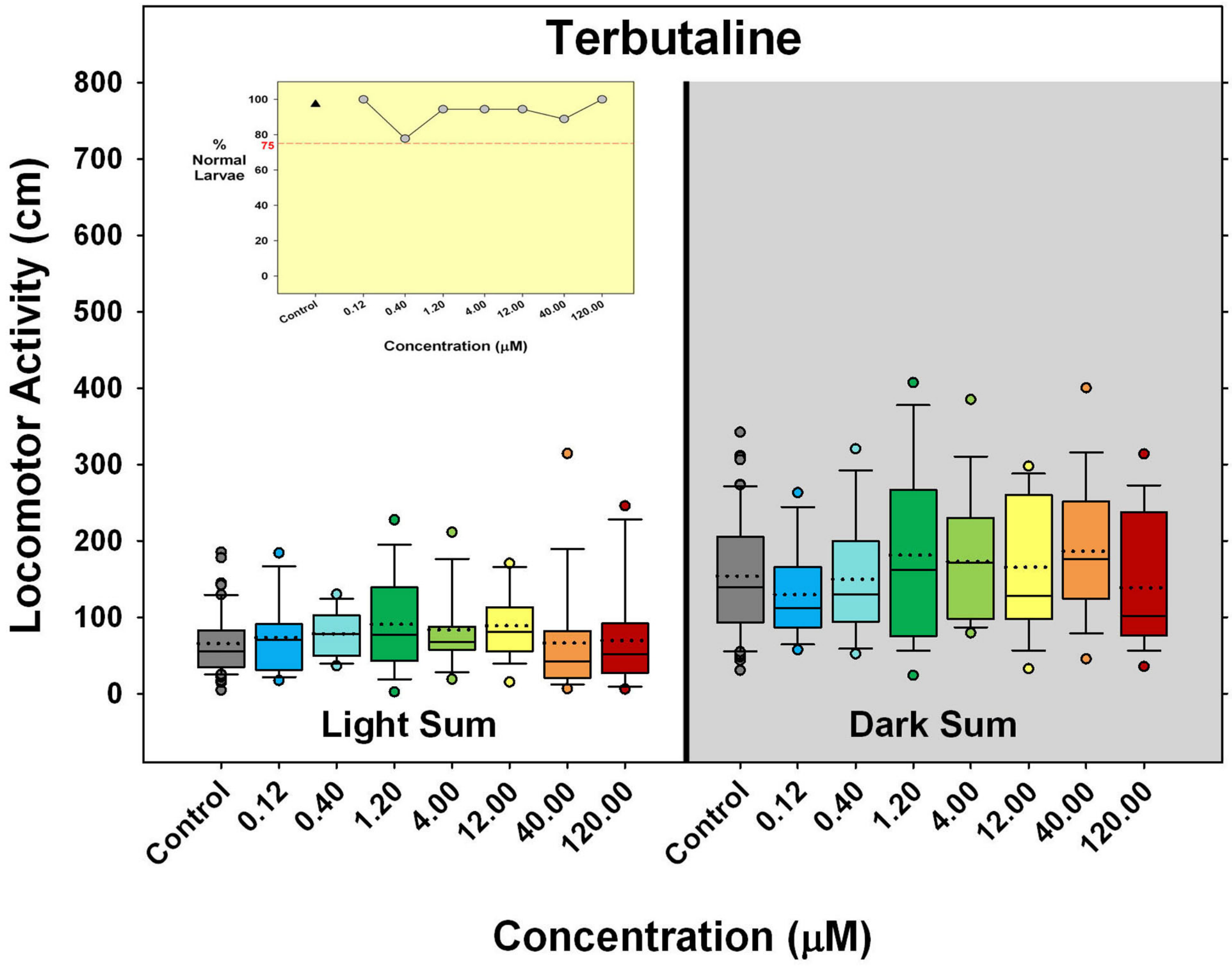

Terbutaline
Locomotor Activity (cm)
% Normal Larvae
75
Concentration (μM)
Light Sum
Dark Sum
Control
0.12
0.40
1.20
4.00
12.00
40.00
120.00
Concentration (μM)

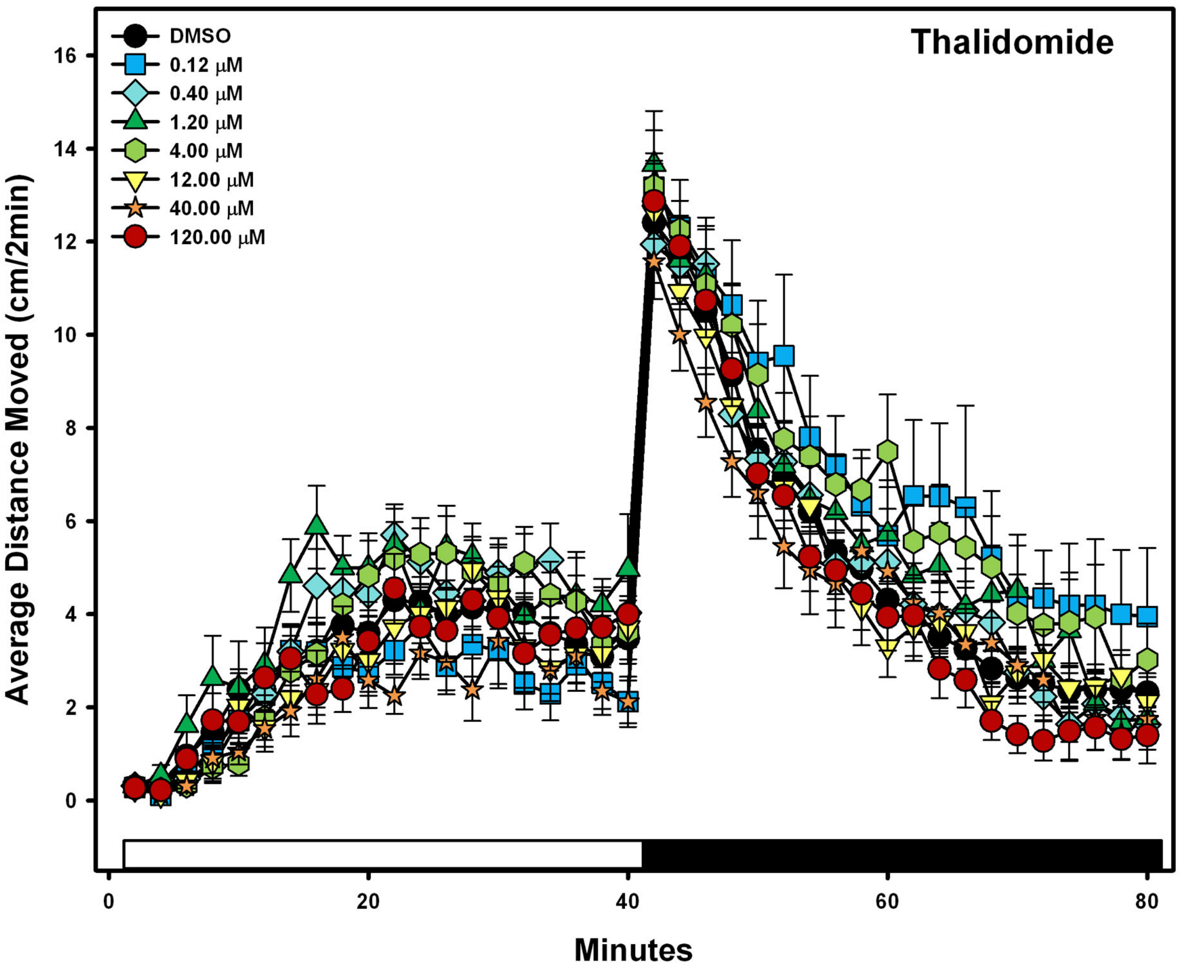
Thalidomide
DMSO
0.12 µM
0.40 µM
1.20 µM
4.00 µM
12.00 µM
40.00 µM
120.00 µM
Average Distance Moved (cm/2min)
Minutes

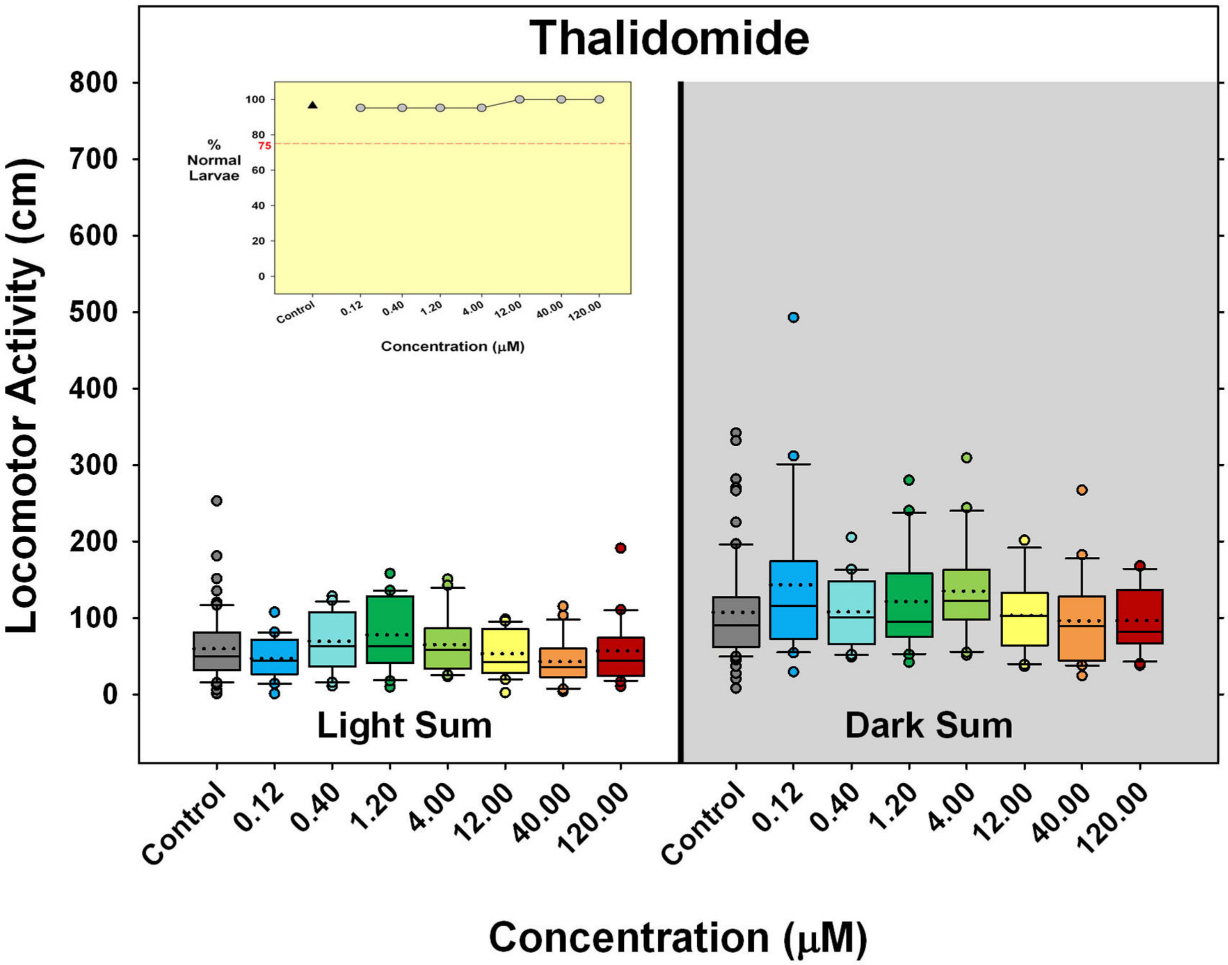
Thalidomide
Locomotor Activity (cm)
Light Sum
Dark Sum
% Normal Larvae
75
Concentration (µM)
Control
0.12
0.40
1.20
4.00
12.00
40.00
120.00
Concentration (µM)

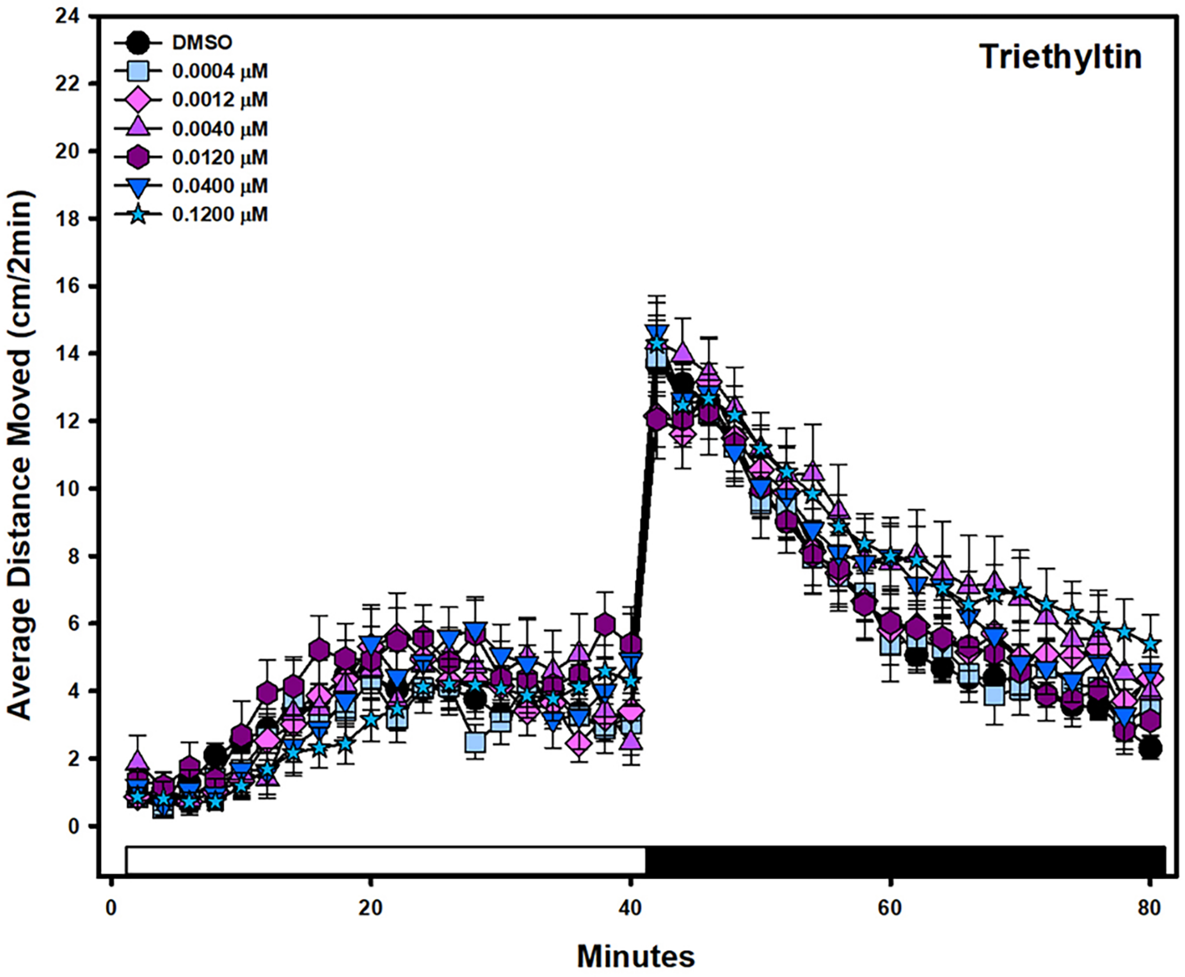
Triethyltin
DMSO
0.0004 μM
0.0012 μM
0.0040 μM
0.0120 μM
0.0400 μM
0.1200 μM
Average Distance Moved (cm/2min)
Minutes

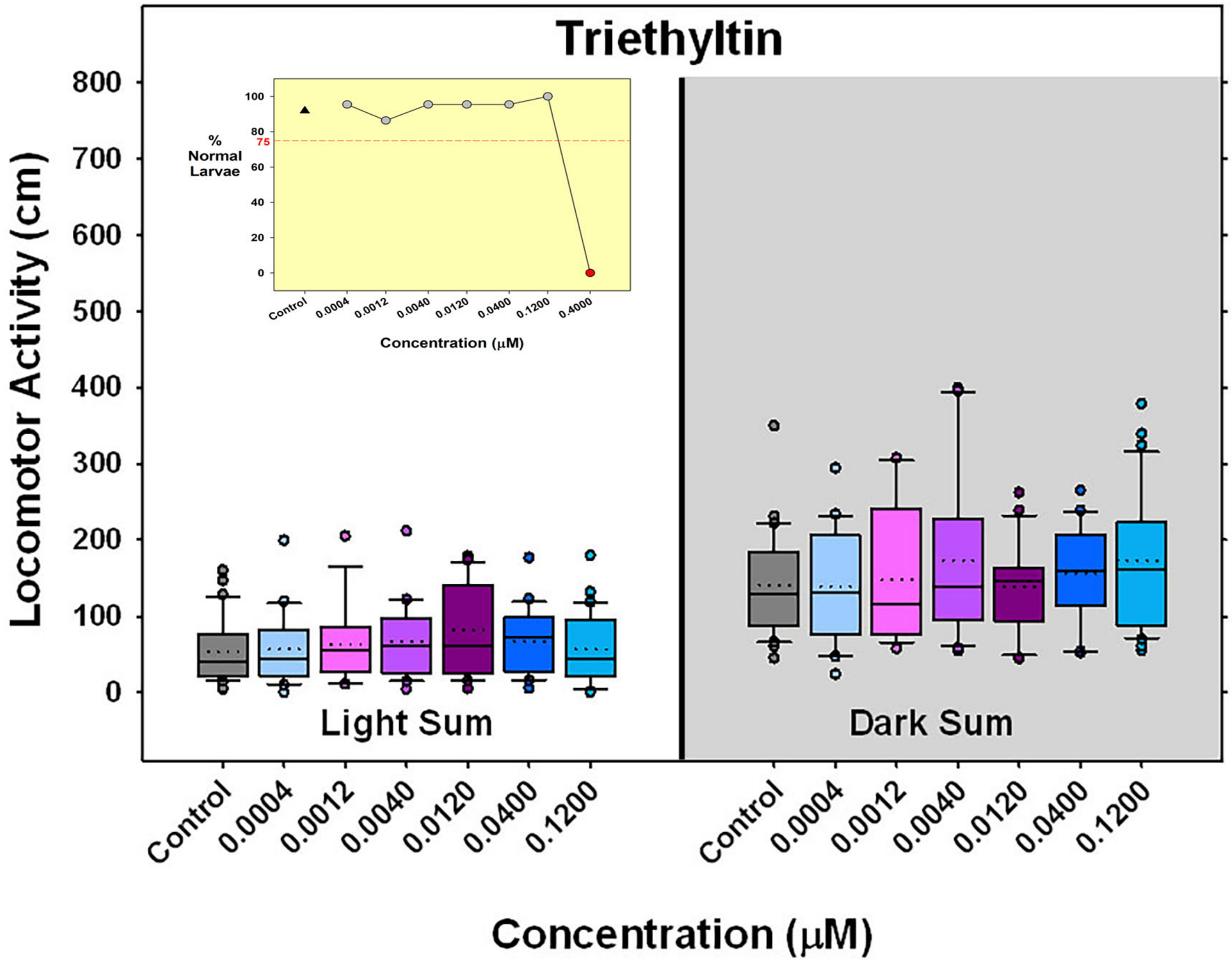
Triethyltin
Locomotor Activity (cm)
% Normal Larvae
75
Concentration (μM)
Light Sum
Dark Sum
Control
0.0004
0.0012
0.0040
0.0120
0.0400
0.1200
0.4000

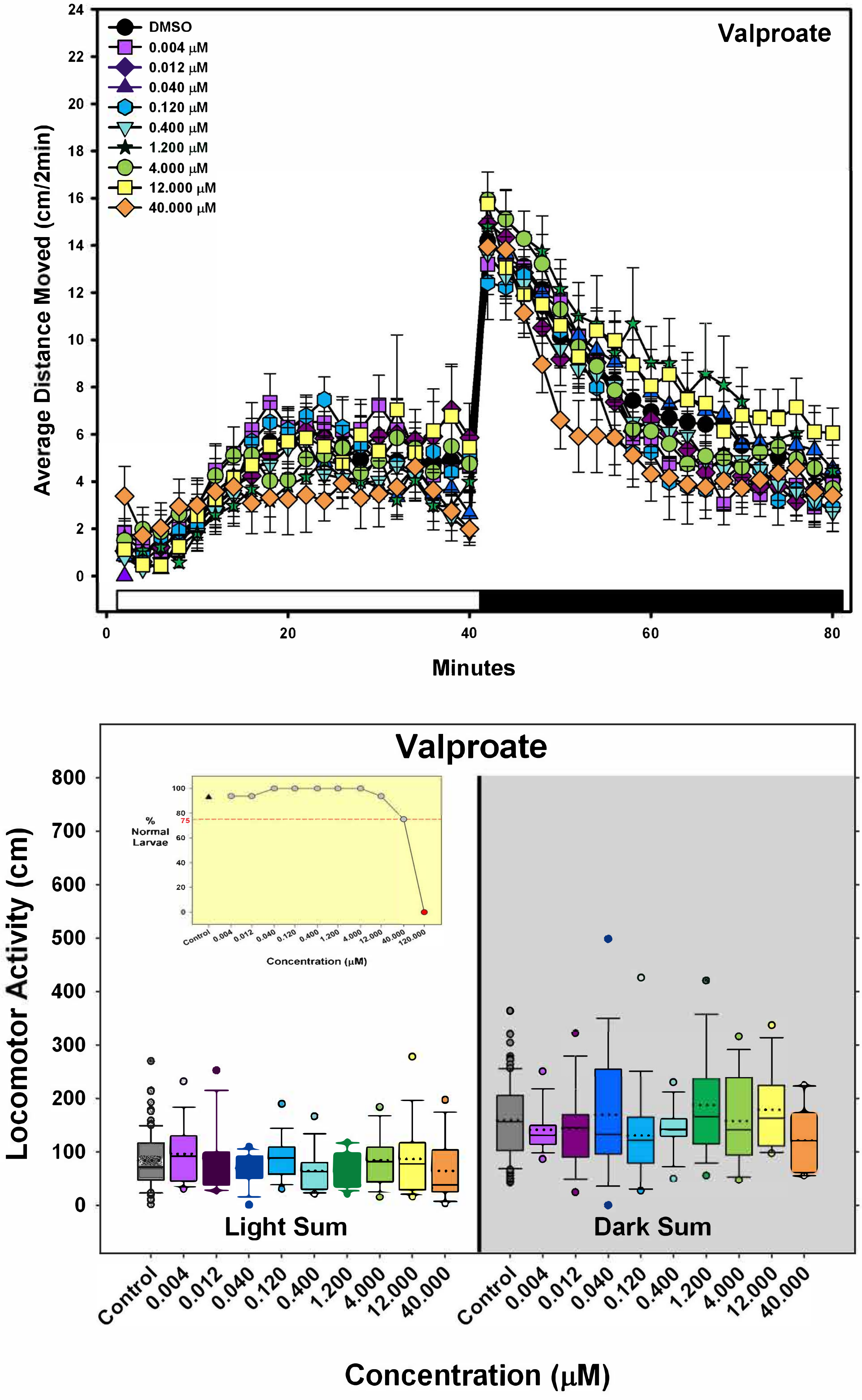

Valproate
DMSO
0.004 µM
0.012 µM
0.040 µM
0.120 µM
0.400 µM
1.200 µM
4.000 µM
12.000 µM
40.000 µM
Average Distance Moved (cm/2min)
Minutes
Valproate
Locomotor Activity (cm)
% Normal Larvae
Concentration (µM)
Light Sum
Dark Sum
Control 0.004 0.012 0.040 0.120 0.400 1.200 4.000 12.000 40.000
Concentration (µM)

Supplemental Figure S2—Time Course Behavioral Graph, Activity Box Plots and Developmental Toxicity for each Chemical. One page for each chemical presents the results as average locomotor activity (cm) ± SEM per 2 minutes with the white bar at the bottom indicating the Light period, and the dark bar indicating the Dark period in the top panel and behavioral data box plots with developmental toxicity as a line graph in the inset. Box plots show locomotor activity for both the Light and Dark (gray background) periods. The box represents the interquartile range (middle 50%), the top of the box to the top error bar is the upper quartile (75th percentile) while the bottom of the box to lower error bar is the lower quartile (25th percentile). The solid line in the box is the median and the dotted line in the box is the mean. The top whisker/error bar indicates the maximum and the bottom whisker/error bar indicates the minimum. The developmental toxicity inset shows the percent of normal larvae for control and each concentration. The dotted red line is the 75% line and concentrations that fall below are considered developmentally toxic and not included in behavioral analyses. The triangle represents control data, and the gray circles indicate results at each concentration. All concentrations are in micromolar (µM).
